# Conditional Risks of Biochemical Failure and Prostate Cancer–Specific Death in Patients Undergoing External Beam Radiotherapy: A Secondary Analysis of 2 Randomized Clinical Trials

**DOI:** 10.1001/jamanetworkopen.2023.35069

**Published:** 2023-09-26

**Authors:** Gregory S. Alexander, Rebecca F. Krc, James W. Assif, Kai Sun, Jason K. Molitoris, Phuoc Tran, Zaker Rana, Søren M. Bentzen, Mark V. Mishra

**Affiliations:** 1 Department of Radiation Oncology, Thomas Jefferson University, Philadelphia, Pennsylvania; 2 Department of Radiation Oncology, University of Maryland Medical Center, Baltimore; 3 Division of Biostatistics and Bioinformatics, University of Maryland Greenebaum Cancer Center, Department of Epidemiology and Public Health, University of Maryland School of Medicine, Baltimore; 4 Department of Radiation Oncology, University of Maryland School of Medicine, Baltimore

## Abstract

**Importance:**

As patients achieve years of survival after treatment for prostate cancer, the risk of biochemical failure (BF) or prostate cancer–specific death (PCSD) may evolve over time, with clinical relevance to both patients and clinicians.

**Objective:**

To determine conditional BF–free survival, PSCD, and overall survival estimates for patients with low- or intermediate-risk prostate cancer enrolled in the Radiation Therapy Oncology Group (RTOG) 0126 and RTOG 0415 clinical trials. A secondary objective was to determine whether prognostic factors at diagnosis remain relevant at later points in follow-up.

**Design, Setting, and Participants:**

A pooled secondary analysis of patients treated with external-beam radiotherapy alone and enrolled in the prospective randomized clinical trials RTOG 0126 and RTOG 0415 was performed. Patients included for analysis were enrolled between March 2002 and December 2009 with a median follow-up of 6.9 years. Overall survival was calculated using the Kaplan-Meier method at various survivorship time points. Cumulative incidence was used to calculate BF rates using the Phoenix definition, as well as PCSD. Risk factors such as Gleason score, tumor (T) stage, prostate-specific antigen level, and the equivalent dose in 2 Gy fractions of prescribed dose were analyzed at different time points using multivariable Cox proportional hazards modeling. Data were analyzed from November 2021 to February 2023.

**Main Outcomes and Measures:**

Conditional risks of BF and PCSD after completion of external-beam radiotherapy.

**Results:**

A total of 2591 patients (median [IQR] age, 69 [63-73] years) were included in the study with a mean (range) PSA level of 7.1 (4.7-8.9) ng/mL, 1334 patients (51.5%) with a Gleason score 6 disease, and 1706 patients (65.8%) with T1 disease. Rates of BF from time of treatment were 1.63% (95% CI, 1.20%-2.18%) at 1 year, 7.04% (95% CI, 6.09%-8.08%) at 3 years, 12.54% (95% CI, 11.28%-13.88%) at 5 years, and 22.32% (95% CI, 20.46%-24.24%) at 8 years. For patients surviving 1, 3, and 5 years without BF, the rates of BF in the next 5 years were 14.20% (95% CI, 12.80%-15.66%), 17.19% (95% CI, 15.34%-19.14%), and 18.85% (95% CI, 16.21%-21.64%), respectively. At the initial time point, the rate of PCSD in the next 5 years was 0.66% (95% CI, 0.39%-1.04%). For patients who achieved 1, 3, 5, and 8 years of survivorship, the rates of PCSD in the next 5 years were 1.16% (95% CI, 0.77-1.67) at 1 year, 2.42% (95% CI, 1.74%-3.27%) at 3 years, 2.88% (95% CI, 2.01%-3.99%) at 5 years, and 3.49% (95% CI, 0.98%-8.73%) at 8 years.

**Conclusions and Relevance:**

In this secondary analysis of 2 randomized clinical trials of patients undergoing external beam radiotherapy for prostate cancer, the conditional risks of BF and death from prostate cancer increased with time for patients with low- and intermediate-risk prostate cancer treated with radiotherapy alone. These results could inform optimal trial design and may be helpful information for patients evaluated in follow-up.

**Trial Registration:**

ClinicalTrials.gov Identifier: NCT00033631; NCT00331773

## Key Points

**Question:**

How do the risks of biochemical failure (BF) and prostate cancer–specific death (PCSD) probability evolve at later points of survivorship in patients with low- and intermediate-risk prostate cancer treated with external-beam radiotherapy alone?

**Findings:**

In this secondary analysis of 2591 patients from 2 randomized clinical trials, patients were found to be at an increasing risk of BF and PCSD in the years after completion of therapy.

**Meaning:**

Results suggest that patients with low- and intermediate-risk prostate cancer should be counseled on the long-term risks of BF and PCSD, and these data can aid in shared decision-making to guide frequency of long-term prostate-specific antigen surveillance.

## Teaser

This secondary analysis of 2 randomized clinical trials investigates how risks of biochemical failure and prostate cancer–specific death probability evolve in the years after completion of therapy in patients with low- and intermediate-risk prostate cancer treated with external-beam radiotherapy alone.

## Introduction

Conditional survival is defined as the future survival probability that is calculated after a particular length of survival time is achieved. The risk of disease recurrence or cancer-related death may evolve over time; thus, such estimates can help guide clinical follow-up as well as aid in prognostication for patients seen at later time points after completion of treatment. Conditional survival estimates have been published for patients with various malignant neoplasms, such as colon,^1^ rectal,^2^ bladder,^3^ pancreas,^4^ breast,^5^ and prostate cancer after prostatectomy.^6^ Differences in conditional survival may be quite dramatic at varying time points because the risk of cancer-related death generally decreases over time from completion of therapy. For example, the probability of dying from cancer-related causes for patients who have undergone complete resection for pancreatic cancer drops dramatically as they reach later end points of survivorship.^4^ For patients with prostate cancer treated with external-beam radiotherapy (EBRT), the interaction between years of survivorship achieved and risk of biochemical failure (BF) is unknown.

Patients with prostate cancer can survive for years and even decades after diagnosis and treatment. Given this long natural history, the relative risk of disease recurrence at later time points is of clinical relevance, particularly for younger patients. By providing BF-free survival probabilities, clinicians can better individualize follow-up regimens for patients by adjusting the frequency of prostate-specific antigen (PSA) monitoring. Such probabilities also can help researchers determine the ideal length of clinical trial follow-up needed for evaluation of meaningful end points. In addition, this would be a useful tool with which clinicians could educate their patients on the probability of experiencing BF when seen years after completion of definitive therapy. One retrospective analysis^6^ has demonstrated that for patients with high-risk disease who have undergone radical prostatectomy, the risk of BF decreases as the interval from treatment increases. Whether this relationship remains similar for patients with more favorable-risk disease treated with EBRT alone remains unknown. In addition, it is unclear whether disease-related factors, such as Gleason score (GS), PSA level, tumor (T) stage, and prescribed dose of radiotherapy continue to be relevant at later posttreatment time points.

In this study, we sought to determine the conditional survival probability and probability for prostate cancer cancer–specific death (PCSD) for patients with low- and intermediate-risk prostate cancer treated in prospective, randomized, Radiation Therapy Oncology Group (RTOG) clinical trials 0126 and 0415. We also sought to determine the effect of increasing years of BF-free survivorship on risk of BF in subsequent years. A secondary objective was to examine factors that were prognostic at the time of treatment initiation and determine whether these remain relevant at later time points.

## Methods

### Patients, Trial Design, and Procedures

This was a post hoc secondary analysis of RTOG 0126 and RTOG 0415 randomized clinical trials using data sets from the data archive of the National Clinical Trials Network/National Cancer Institute Community Oncology Research Program. Data were originally collected from clinical trials RTOG 0126 and RTOG 0415 (Supplement 1 and Supplement 2, respectively). The institutional review board of the University of Maryland declared this research as not involving human participants owing to the use of deidentified publicly available trial data. Therefore, informed consent was not required. Information on race and ethnicity was collected at the time of study entry in accordance with the National Institutes of Health Revitalization Act of 1993 and included the following race and ethnicity categories: Black, White, and other (included American Indian or Alaskan Native, Asian, Native Hawaiian or Other Pacific Islander, multiracial, or unknown). This study followed the Consolidated Standards of Reporting Trials (CONSORT) reporting guidelines.

**Figure 1.**
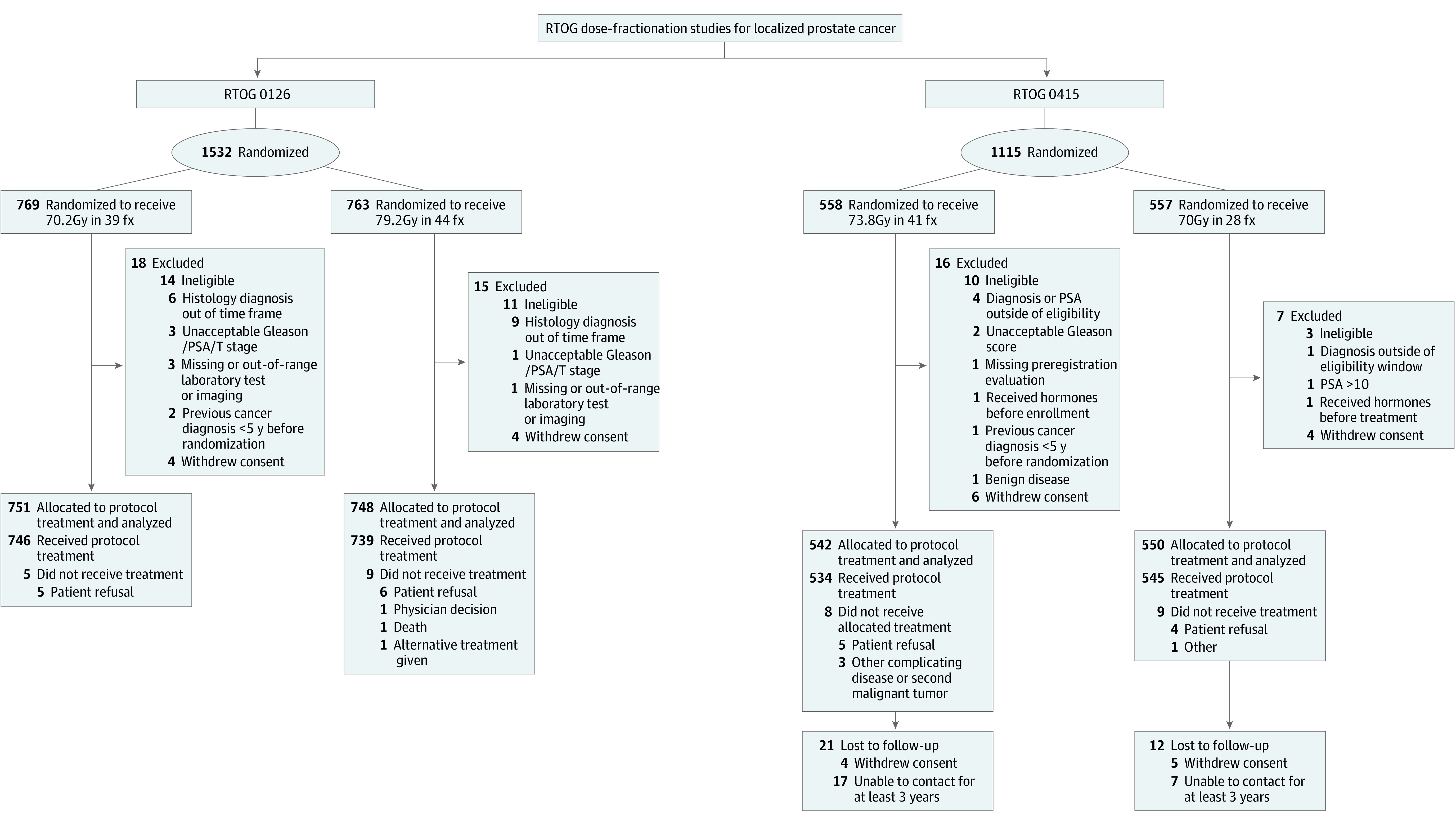
Study Flow Diagram Flow diagram of randomized clinical trials Radiation Therapy Oncology Group (RTOG) 0126 (A) and RTOG 0415 (B). Fx indicates fraction; PSA, prostate-specific antigen.

RTOG 0415 and RTOG 0126 were prospective trials that randomly assigned patients with low- and intermediate-risk prostate cancer, respectively, to receive various radiotherapy fractionation schedules without the addition of androgen deprivation. Randomization and patient numbers are included in Figure 1. RTOG 0126 included patients with T1b (incidental finding following procedure for benign condition) or T2b (palpable disease occupying more than 50% of 1 lobe) disease with either GS of 2 to 6 and PSA level greater than or equal to 10 ng/mL and less than 20 ng/mL (to convert to micrograms per liter, multiply by 1) or GS of 7 and PSA level less than 15 ng/mL. All eligible patients received conventionally fractionated radiotherapy in 1.8-Gy fractions and were randomly assigned to receive either 70.2 Gy or dose-escalated radiotherapy to a total dose of 79.2 Gy. RTOG 0415 randomly assigned eligible patients with low-risk disease (stage T1-2a with GS ≤6 and PSA level <10 ng/mL) to receive conventionally fractionated radiotherapy of 73.8 Gy in 41 fractions or moderately hypofractionated radiotherapy of 70 Gy in 28 fractions. For both trials, radiotherapy was delivered using either 3-dimensional conformal radiation therapy or intensity-modulated radiation therapy per institutional practice.

### Statistical Analysis

Overall survival was calculated using the Kaplan-Meier method at various survivorship time points. Cumulative incidence was used to calculate PCSD and BF rates, using the Phoenix definition with death as a competing risk. Risk factors of race, age, GS, T stage, PSA level, and the equivalent dose in 2 Gy fractions (EQD2) of prescribed dose were analyzed at different time points using multivariable Cox proportional hazard ratio (HR) modeling. The EQD2 of prescribed dose was calculated with the assumption of an α/β ratio of 2.7 and a dose-equivalent of proliferation of 0.24 Gy per day^7^ to account for differences in total treatment time as previously described.^8^ PSA level and EQD2 were analyzed as continuous variables. A 2-sided *P* value <.05 was considered statistically significant. Data were analyzed from November 2021 to February 2023 using SAS software, version 9.4 (SAS Institute).

## Results

### Study Patients

Patient demographics are detailed in Table 1. The combined analysis included a total of 2591 patients (median [IQR] age, 69 [63-73] years; range, 33-87 years) with a median follow-up of 6.9 years (range, 0-13 years; IQR, 5.11-8.66 years). The mean (IQR) PSA level was 7.1 (4.72-8.9) ng/mL. A total of 1334 patients (51.5%) had a GS of 6 or less, and 1706 patients (65.8%) had T1 disease. Participant data from the following race and ethnicity categories were included: 378 Black (14.6%), 2118 White (81.7%), and 95 other (3.7%; identifying as American Indian or Alaska Native, Asian, Native Hawaiian or Other Pacific Islander, multiracial, or unknown).

**Table 1.** Baseline Patient Characteristics

Characteristic	Patients, No. (%)		
	RTOG 0126 (n = 1499)	RTOG 0415 (n = 1092)	Combined (N = 2591)
Patient age, median (IQR), y	71 (65-74)	67 (62-72)	69 (63-73)
Race			
Black	188 (12.5)	190 (17.4)	378 (14.6)
White	1252 (83.5)	866 (79.3)	2118 (81.7)
Other/unknown^a^	59 (4)	36 (3.3)	95 (3.7)
Performance status			
0	1371 (91.5)	1012 (92.7)	2383 (92)
1	128 (8.5)	82 (7.5)	210 (8)
Gleason score			
≤6	242 (16.1)	1092 (100)	1334 (51.5)
7	1257 (83.9)	0 (0)	1257 (48.5)
T stage			
T1	853 (56.9)	853 (78.1)	1706 (65.8)
T2	646 (43.1)	239 (21.9)	885 (34.2)
PSA			
Mean (IQR), ng/mL	8.2 (5.3-10.8)	5.6 (4.2-7.14)	7.1 (4.72-8.9)
<10 ng/mL	1042 (69.5)	1092 (100%)	2134 (82.4%)
≥10 ng/mL	457 (30.5)	0 (0%)	457 (17.6%)

Abbreviations: PSA, prostate-specific antigen; RTOG, Radiation Therapy Oncology Group; T, tumor.

SI conversion factor: To convert PSA from nanograms per milliliter to micrograms per liter, multiply by 1.

^a^ Other/unknown includes American Indian or Alaskan Native, Asian, Native Hawaiian or Other Pacific Islander, more than 1 race, or unknown.

RTOG 0126 enrolled 1532 patients with intermediate-risk disease between March 2002 and August 2008, of whom 1499 (median [IQR] age, 71 [65-74] years) were eligible and included for analysis. The majority of patients had GS 7 disease (1257 [83.9%]), cT1 disease (853 [56.9%]), and a PSA level less than 10 ng/mL (1042 [69.5%]). RTOG 0415 enrolled 1114 patients with low-risk disease between April 2006 and December 2009, of whom 1092 (median [IQR] age, 67 [62-72] years) were eligible and included for analysis.

### Overall Survival and PCSD

**Figure 2.**
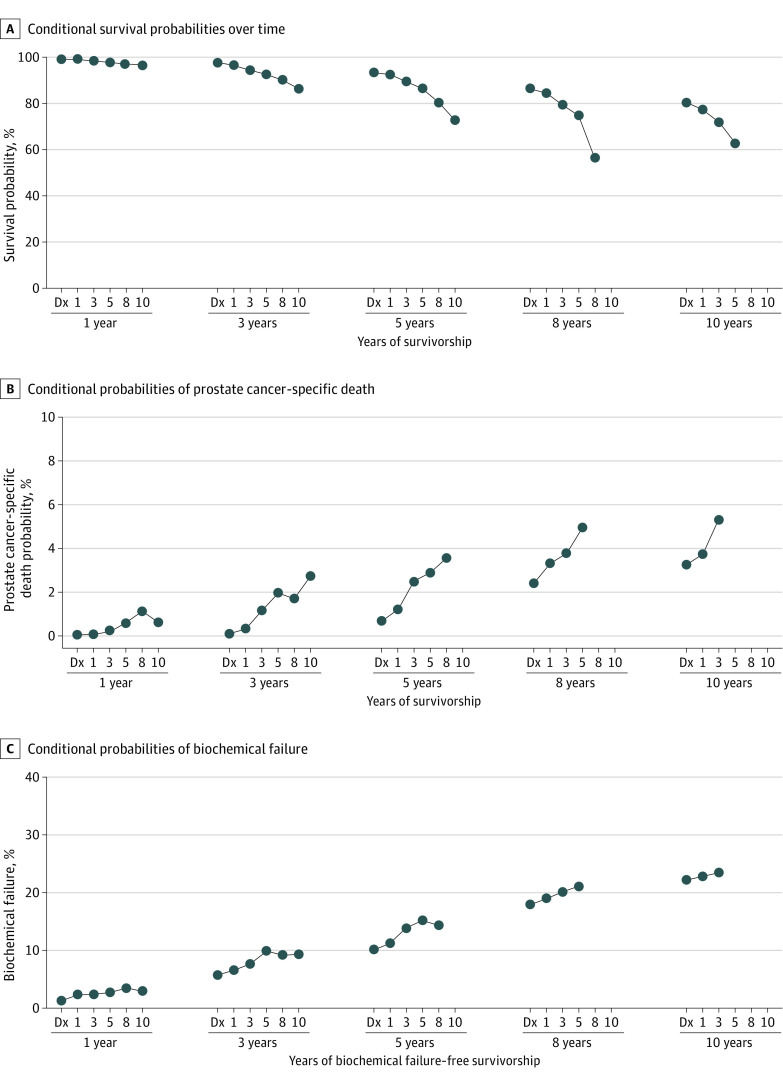
Conditional Probabilities Conditional probabilities of overall survival (A) and prostate cancer–specific death (B) with increasing years of survivorship. C, Conditional probability of biochemical failure with increasing years of biochemical failure–free survival. Dx indicates diagnosis.

Only 531 deaths were recorded at the time of last follow-up, with 79.5% of all patients (2060 of 2591) alive. Overall survival times after treatment were 98.5% at 1 year, 96.2% at 3 years, 89.2% at 5 years, and 41.0% at 8 years, with the majority of deaths being unrelated to prostate cancer. The rates of surviving an additional 5 years were 73.3% at 1-year survival, 42.6% at 3-year survival, and 20.8% at 5-year survival (Figure 2A). Multivariable analyses are listed in Table 2 and showed that at diagnosis, a GS of 7 (HR, 1.35; 95% CI, 1.04-1.07; *P* = .003), increasing age (HR, 1.05; 95% CI, 1.04-1.07; *P* < .001), and increasing PSA level (HR, 1.04; 95% CI, 1.01-1.06; *P* = .01) were associated with all-cause mortality. For those who achieved survivorship at 5 years, only age (HR, 1.07; 95% CI, 1.05-1.09; *P* < .001) was associated with mortality on multivariable analyses.

**Table 2.** Multivariable Cox Proportional Hazard Ratio Modeling for Clinical Parameters Associated With Overall Survival At Diagnosis and 5 Years of Survivorship

Variable	Diagnosis		5-y of Survivorship	
	HR (95% CI)	*P* value	HR (95% CI)	*P* value
Age	1.05 (1.04-1.07)	<.001	1.07 (1.05-1.09)	<.001
Gleason 7 vs 6	1.35 (1.11-1.65)	.003	1.28 (0.95-1.74)	.11
Stage T2 vs T1	0.90 (1.11-1.65)	.25	0.91 (0.71-1.15)	.42
PSA	1.04 (1.01-1.06)	.01	1.02 (0.98-1.06)	.35
EQD2	1.00 (0.97-1.02)	.84	0.99 (0.96-1.02)	.56

Abbreviations: EQD2, equivalent dose in 2 Gy fractions; HR, hazard ratio; PSA, prostate-specific antigen; T, tumor.

In total, prostate cancer accounted for 54 of all deaths (10.2%) that occurred during the follow-up period. The majority of these deaths (51 [94%]) occurred in those with intermediate-risk disease, with a median (IQR) time of 3.3 (2.3-5.0) years from BF. At the initial time point, the rate of PCSD in the subsequent 5 years was 0.66% (95% CI, 0.39%-1.04%). For patients who achieved 1, 3, 5, and 8 years of survivorship, the rates of PCSD in the next 5 years were 1.16% (95% CI, 0.77-1.67) at 1 year, 2.42% (95% CI, 1.74%-3.27%) at 3 years, 2.88% (95% CI, 2.01%-3.99%) at 5 years, and 3.49% (95% CI, 0.98%-8.73%) at 8 years (Figure 2B). At diagnosis, a GS of 7 (HR, 3.59; 95% CI, 1.59-8.11; *P* = .002), T stage (HR, 1.76; 95% CI, 1.01-3.06; *P* = .045), age (HR, 1.05; 95% CI, 1.00-1.01; *P* = .03), and PSA level (HR, 1.21; 95% CI, 1.12-1.31; *P* < .001) were all statistically significant in association with PCSD. The EQD2 of the prescribed radiation dose trended toward reduced risk of PCSD without reaching statistical significance (HR, 0.97; 95% CI, 0.94-1.01; *P* = .14). Only a GS of 7 (HR, 4.28; 95% CI, 1.44-12.76; *P* = .009) and PSA level (HR, 1.23; 95% CI, 1.11-1.35; *P* < .001) remained statistically significant associated with PCSD after 5 years of survivorship.

### BF

Rates of BF from time of treatment were 1.63% (95% CI, 1.20%-2.18%) at 1 year, 7.04% (95% CI, 6.09%-8.08%) at 3 years, 12.54% (95% CI, 11.28%-13.88%) at 5 years, and 22.32% (95% CI, 20.46%-24.24%) at 8 years. For patients surviving 1, 3, and 5 years without BF, the rates of BF in the next 5 years were 14.20% (95% CI ,12.80%-15.66%), 17.19% (95% CI, 15.34%-19.14%), and 18.85% (95% CI 16.21%-21.64%), respectively (Table 3 and Figure 2C). At initial trial enrollment, GS (HR, 1.91; 95% CI, 1.55-2.35; *P* < .001), T stage (HR, 1.38; 95% CI, 1.15-1.64; *P* < .001), pretreatment PSA level (HR, 1.11; 95% CI, 1.09-1.14; *P* < .001), and EQD2 of prescription dose (HR, 0.92; 95% CI, 0.90-0.95; *P* < .001) were all significant on multivariable analysis and associated with risk of BF. For those who survived 5 years without BF, all initially associated factors remained statistically significant on multivariable analysis: GS 7 (HR, 2.40; 95% CI, 1.63-3.55; *P* <.001), stage T2 (HR, 1.34; 95% CI, 1.01-1.78; *P* = .04), PSA level (HR, 1.11; 95% CI, 1.07-1.16; *P* < .001), and EQD2 of prescription dose (HR, 0.89; 95% CI, 0.85-0.93; *P* < .001) (Table 3).

**Table 3.** Multivariable Cox Proportional Hazard Ratio Modeling for Clinical Parameters Associated With Biochemical Failure at Diagnosis and 5 Years of BFFS

Variable	Diagnosis		5-y BFFS	
	HR (95% CI)	*P* value	HR (95% CI)	*P* value
Gleason 7 vs 6	1.91 (1.55-2.35)	<.001	2.40 (1.63-3.55)	<.001
Stage T2 vs T1	1.38 (1.15-1.64)	<.001	1.34 (1.01-1.78)	.04
PSA	1.11 (1.09-1.14)	<.001	1.11 (1.07-1.16)	<.001
EQD2	0.92 (0.90-0.95)	<.001	0.89 (0.85-0.93)	<.001

Abbreviations: BFFS, biochemical failure–free survival; EQD2, equivalent dose in 2 Gy fractions; HR, hazard ratio; PSA, prostate-specific antigen; T, tumor.

## Discussion

To our knowledge, we have performed the first prospective pooled analysis of conditional survival, conditional prostate cancer death, and conditional BF probability for longer-term follow-up in patients who have undergone radiotherapy for low- and intermediate-risk prostate cancer while enrolled in prospective phase 3 randomized clinical trials.

We found that patients had an increasing risk of PCSD as they achieved more years of survivorship. The cumulative incidence of prostate cancer death at 8 years from initial diagnosis was just 2.39%; however, for patients who had survived for 5 years, the rate of PCSD in the next 8 years increased to 4.95%. At the initial time point, GS 7, T stage, age, and pretreatment PSA level were all statistically significant in association with PCSD but not EQD2 of prescribed dose. Although there was a trend toward decreased risk of prostate cancer death with dose-escalated radiotherapy, it did not reach statistical significance, likely owing to a low event rate and lack of extended long-term follow-up.

Similarly, the longer patients lived without BF, their risk of BF actually increased. At the initial time point, the risk of BF in the first 5 years was 12.5%, but as patients achieved further survivorship without a BF, the rate of BF progressively increased. Of note, all initial prognostic factors for biochemical control (T stage, PSA level, GS, and EQD2 of prescribed dose) remained statistically significant at later time points. This finding supports the importance of dose-escalated radiotherapy for patients with prostate cancer. Large robust retrospective evidence suggests that the average lag time from BF to prostate cancer death in patients treated with dose-escalated EBRT is more than a decade.^9^ In our pooled analysis, the median time from BF to PCSD was only 3.3 years, which implies that only those who died of the most aggressive prostate cancer relapses were captured in the follow-up period and that many patients who experienced a BF toward the end of trial follow-up may have died of prostate cancer after the conclusion of the trial.

In the Scandinavian Prostatic Cancer Group 5 trial (which before the PSA screening era randomly assigned patients diagnosed with prostate cancer to either radical prostatectomy or watchful waiting), the benefits of prostatectomy became more dramatic as the duration of follow-up increased. With 10 years of follow-up, the number needed to treat to prevent 1 death was 20; at 18 years of follow-up, the number needed to treat had decreased to 8.^10^ Given that dose-escalated radiotherapy continued to remain important at later time points in this analysis, it is possible that with longer follow-up the benefits of dose escalation could have become more pronounced.

In addition, these findings are of clinical relevance and can help guide PSA monitoring for patients as they live longer after treatment. Because we found patients to be at increased risk of biochemical recurrence at later time points, clinicians should be discouraged from “graduating” patients who achieve a good performance status and long anticipated survival even at later time points, especially given the variety of salvage treatment options available. Patients should instead be counseled on the risks of long-term recurrence to discuss potential benefits and risks of PSA surveillance even years after treatment completion.

In terms of overall survival, our study found that as patients live longer beyond definitive treatment of their cancer, their risk of dying from any cause increases, which is expected. Because the median age at enrollment in the trials studied was 69 years and the majority of patients with low- and intermediate-risk prostate cancer will die of other causes, this result was expected and may lead to questions about whether intensification of local therapy is worthwhile. However, just as the probability of experiencing a BF or PCSD evolves with time, so does life expectancy. Although average life expectancy for a male is in the late 70s, once reaching 80 years old, life expectancy increases to 89 years.^11^ Because the majority of patients (79.5%) were alive at last follow-up when they would be at greatest risk for PCSD, our findings call into question the adequacy of follow-up duration for both of these important and well-run randomized clinical trials.

### Limitations

Although these findings are of importance to both clinicians and patients, there are important limitations to our findings. For patients with a GS of 7, we do not have data to categorize them as Gleason grade group 2 or 3. Since enrollment of the first patients in this analysis in 2002, the International Society of Urologic Pathology has published 2 separate consensus statements, which has dramatically changed the manner in which GS is reported. For example, GS 3 is now the lowest grade assignable score for all practical purposes.^12^ Additionally, stage migration through the increasing use of magnetic resonance imaging and magnetic resonance imaging–guided fusion biopsies further complicates comparison with patients treated in the modern era.^13^ However, given the long natural time course of prostate cancer, these limitations cannot be overcome, and it is likely that the conditional risk probabilities remain similar in the modern era.

## Conclusions

In this secondary analysis of 2 randomized clinical trials that treated patients with low- and intermediate-risk prostate cancer with EBRT alone, we found that conditional survival and conditional BF-free survival decreased over time and that the risk of PCSD increased. Initial risk factors for BF, such as EQD2 of prescribed dose, GS, T stage, and PSA level, remained relevant at later time points. Clinicians should be aware that patients are at increasing risk of failure farther out from treatment completion, which suggests that routine PSA screening should continue in routine follow-up, even at later time points, for patients with significant years of anticipated survival. This analysis also confirmed the clinical significance of dose-escalated radiotherapy, which remains important at later time points.

## Notes

Supplement 1. **Trial Protocol.** RTOG 0126

Supplement 2. **Trial Protocol.** RTOG 0415

Supplement 3.
Data Sharing Statement
