# Supplementary material for: Conditional Risks of Biochemical Failure and Prostate Cancer–Specific Death in Patients Undergoing External Beam Radiotherapy: A Secondary Analysis of 2 Randomized Clinical Trials
Source: JAMA Netw Open. 2023 Sep 26;6(9):e2335069. doi: 10.1001/jamanetworkopen.2023.35069 (PMC10523164; doi:10.1001/jamanetworkopen.2023.35069)
Supplement: Supplement 1. — Trial Protocol. RTOG 0126 [file jamanetwopen-e2335069-s001.pdf]

NRG Oncology

RTOG 0126

A PHASE III RANDOMIZED STUDY OF HIGH DOSE 3D-CRT/IMRT VERSUS STANDARD DOSE 3D-CRT/IMRT IN PATIENTS TREATED FOR LOCALIZED PROSTATE CANCER

**Study Chairs**	**6/12/14**
Radiation Oncology<br>Dept. of Radiation Oncology<br>Washington University<br>School of Medicine<br>4921 Parkview Place<br>Campus Box 8224<br>St. Louis, MO 63110<br>*(314)* 362-8566<br>FAX# *(314)* 362-8521<br>Michalski@radonc.wustl.edu	Jeff Michalski, M.D., M.B.A.
Physics and<br>Quality Assurance (4/18/06)	James Purdy, Ph.D.<br>*(916)* 734-3932<br>FAX# *(916)* 454-4614<br>james.purdy@ucdmc.ucdavis.edu
Quality of Life<br>and Outcomes (4/18/06)	Deborah Watkins Bruner, R.N., Ph.D.<br>*(404) 712-9695*<br>*FAX: (404) 727-8514*<br>deborah.w.bruner@emory.edu
Pathology (5/11/06)	Mahul Amin, M.D.<br>*(310)* 423-6631<br>FAX# *(310)* 423-0170<br>aminm@cshs.org

**Activation Date:**	**March 21, 2002**
**Closure Date:**	**August 20, 2008**
**Update Date:**	**May 1, 2014**
**Version Date:**	**June 12, 2014**
	**Includes Amendments 1-8**
	**(Broadcast Date: August 4, 2014)**

NRG Oncology
(215) 574-3189
1-800-227-5463, ext. 4189

This protocol was designed and developed by NRG Oncology. It is intended to be used only in conjunction with institution-specific IRB approval for study entry. No other use or reproduction is authorized by NRG Oncology nor does NRG Oncology assume any responsibility for unauthorized use of this protocol.

INDEX

Schema

Eligibility Check

1.0 Introduction

2.0 Objectives

3.0 Patient Selection

4.0 Pretreatment Evaluations

5.0 Registration Procedures

6.0 Radiation Therapy

7.0 Drug Therapy

8.0 Surgery

9.0 Other Therapy

10.0 Pathology

11.0 Patient Assessments

12.0 Data Collection

13.0 Statistical Considerations

References

Appendix I - Sample Study and Tissue Consent Forms
Appendix II - Performance Status Scoring
Appendix III - Staging System
Appendix IV - Toxicity Criteria
Appendix V - Adverse Event Reporting Guidelines
Appendix VI - 3D-CRT Quality Assurance Guidelines/Facility Questionnaire (9/18/03)
Appendix VII - IMRT Quality Assurance Guidelines/Facility Questionnaire (9/18/03) (9/17/04)
Appendix VIII - Specimen Plug Kit and Instructions (7/10/07)
Appendix IX - Blood Collection Kit Instructions (7/10/07)

NRG ONCOLOGY

RTOG 0126 (10/18/04)

A PHASE III RANDOMIZED STUDY OF HIGH DOSE 3D-CRT/IMRT VERSUS STANDARD DOSE 3D-CRT/IMRT IN PATIENTS TREATED FOR LOCALIZED PROSTATE CANCER (9/18/03)

SCHEMA (9/18/03)

**S**	<u>**Gleason Score/PSA**</u>	**R**	<u>**Arm 1**</u> ***(minimum PTV prescription)***
**T**	1. Gleason 2-6	**A**	3D-CRT or IMRT: 70.2 Gy in 39 fractions
**R**	and PSA ≥ 10 but < 20	**N**	
**A**		**D**	
**T**	2. Gleason 7	**O**	<u>**Arm 2**</u> ***(Minimum PTV prescription)***
**I**	and PSA < 15	**M**	3D-CRT or IMRT: 79.2 Gy in 44 fractions
**F**		**I**	
**Y**		**Z**	
	<u>**Radiation Modality**</u>	**E**	
	1. 3D-CRT		
	2. IMRT		

Treatment is prescribed as a minimum to the planning target volume (PTV) to be delivered at a rate of 1.8 Gy/daily fraction. The PTV includes with margin a clinical target volume that encompasses the prostate and *proximal* seminal vesicles (See section 6.0). A "cone-down" after 55.8 Gy to a planning target volume encompassing the prostate only will be optional for forward planned 3D-CRT. (9/18/03)

<u>**Eligibility**</u>**:** *(See Section 3.0 for details)* **(9/18/03)**
- Histologically confirmed prostate adenocarcinoma within 180 days of randomization
- Zubrod Performance Scale 0-1
- Prostatic biopsy tumor grading by the Gleason Score Classification
- One of the following combinations of factors:
    - Clinical stage T1b-T2b, Gleason score 2-6, and prostate-specific antigen ≥10 but < 20
    - Clinical stage T1b-T2b, Gleason score 7, and prostate-specific antigen < 15
- Clinically negative lymph nodes or histologically negative by nodal sampling or dissection
- No distant metastases *(M0)*
- No previous or concurrent invasive cancers, other than localized basal cell or squamous cell skin carcinoma, unless continually disease free for at least 5 years
- No prior pelvic irradiation, prostate brachytherapy, or bilateral orchiectomy
- No previous or concurrent cytotoxic chemotherapy for this cancer
- No previous hormonal treatment *(no finasteride or phytoestrogen preparation within 3 months prior to registration)*
- No radical surgery or cryosurgery for prostate cancer
- Pretreatment evaluations must be completed as specified in Section 4.1
- Patients must sign study-specific informed consent form prior to randomization.

Required Sample Size: 1520

Institution # _________

RTOG 0126 (10/18/04) **ELIGIBILITY CHECKLIST** ***(9/18/03) (4/18/06)***

Case # _________

_______(Y) 1. Is there histologically confirmed prostate adenocarcinoma within the past 180 days?

_______(2-7) 2. What is the combined Gleason Score Classification ?

______ (< 20) 3. What is the PSA level?

_______(NA/Y) 4. If PSA is >10 <u>and</u> Gleason score 7, was a bone scan done?

_______(T1b-T2b) 5. What is the T stage?

_______(N) 6. Is there evidence of nodal metastases?

_______(N) 7. Is there evidence of distant metastases?

_______(Y) 8. Is there the following combination of factors?

- Clinical stage T1b-T2b, Gleason score 2-6, and prostate-specific antigen ≥ 10 but < 20
- Clinical stage T1b-T2b, Gleason score 7 and prostate-specific antigen < 15

_______(0-1) 9. What is the Zubrod Performance Status?

_______(N) 10. Has the patient had prior pelvic radiation, prostate brachytherapy, bilateral orchiectomy, or cytotoxic chemotherapy for prostate cancer?

_______(N) 11. Has the patient had prior radical surgery or cryosurgery for prostate carcinoma?

_______(N) 12. Any previous hormonal therapy?

_______(Y) 13. If patient was receiving finasteride or the phytoestrogen preparation, "PC-SPES", has it been discontinued 90 days prior to randomization?

_______(N) 14. Has the patient had previous or concurrent invasive cancer within the past 5 years, other than localized basal cell or squamous cell skin carcinoma?

_______(Y) 15. Were pre-treatment evaluations completed per Section 4.1 of the protocol?

_______(N) 16. Are there any major medical or psychiatric illness, which would prevent completion of treatment and/or interference with follow up?

Institution # ______

RTOG 0126 (10/18/04) **ELIGIBILITY CHECKLIST** ***(9/18/03)***

Case # _________

The following questions will be asked at Study Registration:

________________ 1. Name of institutional person registering this case?

________________(Y) 2. Has the Eligibility Checklist *(above)* been completed?

________________(Y) 3. Is the patient eligible for this study?

________________ 4. Date the study-specific Consent Form was signed? *(must be prior to study entry)*

________________ 5. Patient's Initials *(Last, First)* [Initials only, effective 2/2002]

________________ 6. Verifying Physician

________________ 7. Patient's ID Number

________________ 8. Date of Birth

________________ 9. Ethnic Category *(Hispanic or Latino, Not Hispanic or Latino, Unknown)*

________________ 10. Race

________________ 11. Gender

________________ 12. Patient's Country of Residence

________________ 13. Zip Code *(U.S. Residents)*

________________ 14. Patient's Insurance Status

________________ 15. Will any component of the patient's care be given at a military or VA facility?

________________ 16. Treatment Start Date

____________(N/Y) 17. Tissue/blood kept for cancer research?

____________(N/Y) 18. Tissue/blood kept for medical research?

____________(N/Y) 19. Allow contact for future research?

________________ 20. Specify Gleason Score/PSA *(Gleason 2-6 and PSA ≥10 but < 20 or Gleason 7 and PSA < 15)*

(Continued on the next page)

Institution # _

RTOG 0126 (10/18/04) **<u>ELIGIBILITY CHECKLIST</u>** ***(9/18/03)***
Case # __

(page 3 of 3)

_______________ 21. Specify Radiation Modality *(3D-CRT <u>or</u> IMRT)*

_______________ 22. Treatment Assignment

The Eligibility Checklist must be completed in its entirety prior to calling RTOG. The completed, signed, and dated Checklist used at study entry must be retained in the patient's study file and will be evaluated during an institutional NCI/RTOG audit.

Completed by ____________________________________ Date ______________________________

1.0 INTRODUCTION

1.1 Safety Of Dose Escalation

Three-dimensional conformal radiation therapy *(3D-CRT)* offers therapeutic advantages in the management of prostate cancer. Several series have demonstrated a reduction in normal tissue toxicity, both acute and late, with the use of 3D-CRT. RTOG 94-06 has demonstrated that higher cumulative doses of radiation can be delivered with lower risks of late effects compared to historical controls.[1] Preliminary results at dose levels *(specified as a minimum tumor dose)* of 68.4 Gy and 73.8 Gy have been published. Acute toxicity was low, with 53–54% of patients treated to the prostate alone having either no toxicity or grade 1 toxicity. Sixty-two percent of patients treated to the prostate and seminal vesicles followed by a prostate boost had either no toxicity or grade 1 toxicity at either dose level. Few patients *(0–3%)* experienced a grade 3 acute bowel or bladder toxicity, and there were no grade 4 or 5 toxicities. Late toxicity, the primary endpoint on 94-06, has been very low in all patient groups. The majority *(81–85%)* had either no toxicity or mild *(grade 1)* late toxicity. A single late grade 3 bladder toxicity in a patient treated to 73.8 Gy was recorded. There were no grade 4 or 5 late effects in any patient. Compared to historical RTOG controls *(studies 75-06, 77-06)* at 68.4 Gy, no grade 3 or greater late effects were observed when nearly 15 were expected *($p < 0.05$).* At 73.8 Gy, there was a single grade 3 toxicity in a patient when nearly 25 were expected *($p < 0.0005$).*[1] This data was updated with further follow up and included patients with stage T3 disease who had received the study dose to both the prostate and seminal vesicles. The favorable low toxicity results in the first analysis persisted.[2] Patients treated to the next dose level, 79.2 Gy, were analyzed and reported in Spring 2001.[3] With a median follow-up of 27.6 months, only 3 *(1.8%)* patients experienced grade 3 late toxicity, 2 cases of which were related to bladder *(cystitis with gross hematuria)* and 1 related to rectum *(proctitis with rectal bleeding).* There were no grade 4 or 5 late complications. The inability to demonstrate a maximally tolerated dose *(MTD)* at 1.8 Gy fractions prompted the RTOG to continue the study at 2.0 Gy fractions with study arms at 74 Gy and 78 Gy. As of May 2001, follow up on these levels is too short to analyze for toxicity.

Similar dose escalation trials at Fox Chase Cancer Center *(FCCC)* and Memorial Sloan-Kettering Cancer Center *(MSKCC)* have demonstrated the ability to administer high dose image-guided external beam radiation therapy with acceptable toxicity.[4,5] Zelefsky reported toxicity on the dose escalation series from MSKCC. In that study, the planning target volume includes prostate and seminal vesicles for all patients.[6] For patients treated to a dose of 81 Gy with 3D-CRT, a separate boost plan was initiated after 72 Gy, which blocked the anterior rectal wall in all fields. The dose inhomogeneity within the PTV varied from 4% to 7%. Dose-volume histograms were used to assure that no more than 30% of the rectal wall and/or 50% of bladder wall received a maximum dose of 75.6 Gy. With IMRT, the part of the PTV that overlaps with the rectum is limited to 88% of the prescribed dose, and the part that overlaps the bladder is limited to 98% of the prescription. This prescription method is in contrast to the RTOG experience in which only patients with stage T3 received the full study dose to the seminal vesicles. The majority of RTOG 94-06 patients either was not treated to seminal vesicles or had a treatment volume reduction after 55.8 Gy. Bladder and rectal doses were not constrained prospectively due to the lack of data available at the time of protocol development and the desire to use the 94-06 data to define these normal tissue tolerance parameters.

In the series from MSKCC, the five-year actuarial likelihood of developing grade 2 and 3 late GI toxicities was 11% and 0.75%. A prescribed dose exceeding 75.6 Gy, a history of diabetes mellitus, and presence of acute GI symptoms during treatment were all associated with a greater risk of grade $\geq$ 2 late GI toxicities. The five-year actuarial likelihood of the development of grade 2 and 3 late GU toxicities was 10 percent and 3 percent. As in GI toxicity, dose exceeding 75.6 Gy and presence of acute GU symptoms were independent predictors of grade $\geq$ 2 late GU toxicity. It was concluded that dose escalation above 75.6 Gy should utilize intensity modulated radiotherapy in order to avoid grade 2 or greater toxicity.

Lee and colleagues at FCCC described an increase in rectal bleeding in their dose escalation study.[7] When doses to the central axis exceeded 76 Gy, the relative risk of rectal bleeding was 2.0. This prompted Schulteiss et al. to introduce a lateral rectal block on the last 10 Gy of the prostate boost fields. This additional block reduced the rate of grade 2-4 rectal complications.[8] There was an increased rate of GI and GU toxicity in patients receiving adjuvant hormone therapy, a result also described by Michalski in the first analysis of 94-06.[1]

1.2 Dose Response

Retrospective data from single institution series and the Patterns of Care analysis have suggested that prostate cancer is a dose responsive neoplasm: the higher the dose of radiation administered to the tumor, the more likely it is to attain local tumor control.[4, 9-11] With the availability of 3D-CRT, it is felt that the higher radiation doses achievable using this modality will improve local tumor control in patients with localized adenocarcinoma of the prostate. Local control is a critical endpoint in the management of patients with prostate cancer. Fuks, et al.[12] have demonstrated that local tumor progression serves as a nidus for the development of subsequent distant metastases and may ultimately compromise patient survival.

Higher doses of radiation have been known to increase cancer control in patients with adenocarcinoma of the prostate.[13-14] Unfortunately, high doses without careful conformal treatment planning leads to higher complication rates.[15] As described above, the RTOG and single institution studies have demonstrated that higher doses of radiation therapy can be safely administered using 3D-CRT.[5, 13-14, 16] In the single institution prospective phase I/II dose escalation trials, investigators at FCCC and MSKCC have reported that the higher radiation doses are leading to an improved biochemical disease-free survival in patients with localized prostate cancer.[5, 13] Doses in excess of 75.6 Gy to the prostate improve freedom from biochemical recurrence and local disease progression.[5] Radiation dose administered to the target volume was as important a prognostic factor in both of these series as was PSA, clinical stage, and Gleason score.

Recently, Pollack reported in a single-institution randomized trial that patients receiving 78 Gy to an ICRU reference point within the gland had a better *(79%)* biochemically disease-free survival *(BNED)* than patients receiving 70 Gy *(69%)*, p=0.058.[17] The patients with a presenting PSA > 10 had the most dramatic improvement in freedom from PSA failure with a 75% BNED at 78 Gy versus 48% BNED at 70Gy, p=0.011. The patients in this risk group demonstrated a reduction in the incidence of distant metastases *(98% metastasis free)* after receiving 78 Gy compared to patients receiving 70 Gy *(87% metastasis free)*, p=0.054. This provides supportive evidence of Fuks' hypothesis that inadequate local therapy will allow local tumor to become a source of metastatic disease.[12]

1.3 Trial Justification

This trial is felt to be important to prove that the higher doses achievable with 3D-CRT are justified by an improvement in local tumor control, BNED *(or freedom from PSA failure),* and overall survival. Data from single-arm phase II dose escalation studies have suggested an improvement in tumor control with higher doses, but this strategy has not yet been adopted by the community of radiation oncologists.[5, 13] The preliminary results from the MD Anderson Phase III trial *(that included only 301 evaluable patients)* may not show a sustainable advantage to high dose 3D-CRT, and it may not have been large enough to identify a survival advantage to higher doses *(as is proposed in this trial).* Furthermore, patients treated in the 70 Gy arm were not treated with 3D CRT techniques, high-dose arm patients had only a portion of their treatment given with 3D CRT, and the 78 Gy dose was prescribed to the isocenter. This prescription may represent a minimum dose to the prostate and margin of 74 Gy *(assuming just 5% heterogeneity in the PTV).* Thus, the results of this trial may not have fully utilized the advantages to 3D CRT, particularly because there was not a difference in the post-radiotherapy biopsy rate between the two dose arms.[17] The data from MSKCC suggests that microscopic disease persists until the prescribed minimum dose exceeds 75.6Gy.[5]

A randomized study is being conducted at Massachusetts General Hospital and Loma Linda using proton beam to treat patients to doses of 70.2 Gy vs 79.2 Gy. The primary endpoint of this study is local control. Although this study is important, the applicability of the results from the trial will be restricted due to the limited availability of proton therapy in US radiation oncology facilities, and the relatively small sample size may not be able to test the hypothesis that higher dose results in improved local tumor control and this in turn improves survivorship.

The Medical Research Council *(MRC)* in the United Kingdom is also conducting a randomized trial of high dose versus low dose conformal radiation therapy. In this trial, the conventional dose is 64 Gy, and the experimental arm is 74Gy; both arms prescribing dose to the isocenter *(personal communication, David Dearnaley).* Due to the lack of PSA screening in the UK, patients in this study are likely to have more advanced cancer than those we anticipate registering to this trial. Furthermore, even the high dose arm is not strikingly higher than our low dose control arm, after correction for prescription point and target volume heterogeneity.

The Netherlands Cancer Institute *(NKI)* is conducting a randomized dose study using 3D-CRT in both arms. Like the MD Anderson and MRC trials, the dose prescription is to the isocenter with 68 Gy as the standard dose arm and 78 Gy for the experimental arm *(personal communication, Joos Lebesque).* This study includes patients of all stages and risk factors of disease. The target accrual is 600 patients with four stratification groups.

1.4 Patient Selection

The dose escalation series from FCCC and MSKCC suggest that dose escalation is either not advantageous or minimally beneficial in patients with favorable prostate cancer. Zelefsky described the favorable population as being without adverse prognostic factors *(PSA ≤10, Gleason score 2 to 6, clinical stage T1-2).* Intermediate risk patients had a single adverse factor *(PSA >10, Gleason score ≥7, <u>or</u> clinical stage >T2),* and unfavorable risk patients had two or more adverse risk factors. In the MSKCC series, the intermediate risk patients had the largest improvement in four-year BNED outcome with doses ≥ 75.6 Gy *(79% versus 56%, p=0.04).* The unfavorable group also had a significant, but smaller, improvement *(58% vs 30%, p=0.03)* in four-year BNED.[5] Hanks reported a significant dose

response in patients presenting with PSA >10.[6] Patients presenting with PSA >20 only had a significant improvement when receiving doses >75.6 Gy if they had no other adverse risk factors.[18] This represented nearly 15% of their patients with initial PSA >20. Patients with PSA ≤10 demonstrated an improvement in BNED with higher doses if they had at least one adverse risk factor, including Gleason score ≥7, clinical stage T2b-T3, or perineural invasion.[19]

The MD Anderson randomized trial benefited patients presenting with PSA >10. No other subset demonstrated as significant improvement in BNED outcome as did these patients.

In summary, the dose escalation and randomized series demonstrate the most striking dose effect in patients with "intermediate risk" prostate cancer. Patients with more favorable disease may not benefit from high doses because the volume of disease at diagnosis is small enough that conventional doses are adequate to eradicate disease. On the other hand, high-risk patients may have competing mortality from occult metastatic disease at diagnosis, and although they may have a local control benefit, long-term BNED and disease-free survival rates are unaffected. The patient population selected for this study corresponds closely to the patients in Group 2 on RTOG 94-06. The risk of seminal vesicle invasion exceeds 15%, but the risk of lymph node metastases is low. The treatment volume in these patients will be prostate and seminal vesicles treated to a dose that is expected to control microscopic disease followed by a boost to the prostate alone to the dose to which the patient was randomized.

1.5 Dose Selection (9/18/03)

RTOG 94-06 had determination of the MTD for 3D-CRT of localized prostate cancer as its primary objective. Endpoints for this study are late effects, >18 months after completion of radiotherapy. Dose level III *(79.2 Gy, 44 fractions)* has adequate follow up to assess the safety of this prescription. Ryu reported these results at ASCO 2001.[3] As Michalski demonstrated at dose levels I and II, there was a significant reduction in ≥ grade 3 late GI and GU adverse effects, even at this higher dose level.[1, 2] Unfortunately, follow up on dose levels IV and V is inadequate to assess safety. Therefore, we have chosen to use a prescribed dose as administered on dose level III of RTOG 94-06. Due to a concern of rectal toxicity with minimum PTV doses exceeding 75 Gy, the RTOG chose to limit dose to the rectum on this arm by constraining the PTV dose to 73.8 Gy while keeping the minimum dose to the prostate at 79.2 Gy. Data from the RTOG 3D QA center database show that the 94-06 prescription, on average, delivers 82 Gy to the ICRU reference *(point close to isocenter)* and the minimum dose to > 95% of the PTV-HD was 78 Gy. To simplify the prescription for this protocol, the ICRU reference point will be used to specify the prescription dose. On the high dose arm of this study, patients will receive 82.28 Gy in 44 fractions of 1.87 Gy/day to the ICRU reference point. The PTV-HD minimum must be at least 78 Gy. On the low dose arm, patients will receive 72.93 Gy in 39 fractions of 1.87 Gy/day. In each arm, the seminal vesicles will be treated to a dose of 57.97 Gy in 31 fractions before being shielded for the remainder of the treatment course. Dose to the rectum and bladder will be constrained *(See Section 6.0)*. As in 94-06, no part of the rectum should receive more than the ICRU reference point prescription dose.

RTOG 94-06 has accrued patients to two dose levels following dose level III *(79.2 Gy)*. Dose levels IV and V treated patients to a minimum PTV dose of 74 Gy and 78 Gy, respectively. The dose level IV *(74 Gy)* did not represent an escalation *per se* as the prescription was similar to dose level II *(73.8 Gy)* and less than dose level III *(79.2 Gy),* albeit with a larger dose per fraction. Biologically, it is similar to dose level III. Dose level V *(78 Gy)*, on the other hand, represents a biologically more aggressive dose than dose level III *(79.2 Gy),* even though nominally it is slightly less than that level. The larger dose per fraction and shorter overall course of therapy may have more biological effect against both tumor and normal tissues. [20]Unfortunately, at the time of the development of this protocol, neither of those arms has mature follow up to determine whether they are "tolerable." Dose level V closed to accrual October 31, 2000, and the last patient did not complete therapy until January of 2001. With 18 months of follow up required to conduct an analysis, the tolerability of this level will not be known until early 2002.

The RTOG feels that it is appropriate to initiate this study with the 79.2 Gy dose of RTOG 94-06. First, it is not clear that dose level V is "tolerable." Indeed, dose level III *(79.2 Gy)* may represent the MTD. Secondly, accrual to this randomized trial is not likely to have significant patient numbers by the early 2002 given the lag to accrual that many cooperative group clinical trials experience while trials are undergoing initiation. At the time of analysis of the dose level V, the RTOG executive committee and the GU executive committee will decide if an amendment is required to change the prescription for the PTV-HD to reflect the dose delivered on dose level V of RTOG 9406.

The followup of dose level V on RTOG 9406 demonstrates no excess of grade 3 or greater complications compared to historical experience. However, the incidence of grade 2 or greater late effects is significantly higher than lower dose levels of RTOG 9406 or historical experience. For this reason, the current dose randomization will not be escalated further and the two arms will remain unchanged.

Because of the growing availability of Intensity Modulated Radiation Therapy (IMRT) and the desire to further reduce normal tissue radiation dose volumes, the RTOG GU, Medical Physics and Image-Guided Radiation Therapy committees have agreed that IMRT is an appropriate modality to be used on this clinical trial. Unique characteristics of IMRT planning have required modification of the dose prescription parameters. The study will now use a *minimum* dose prescription to the PTV with Arm 1 patients receiving 70.2 Gy in 39 x 1.8 Gy fractions and Arm 2 patients receiving 79.2 Gy in 44 x 1.8 Gy fractions.

For IMRT, the treatment will be delivered to a single clinical target volume (CTV) that encompasses the prostate and proximal seminal vesicles. This CTV modification for IMRT is being made to minimize the effort institutions must make to plan and perform quality assurance on multiple target volumes. Data from William Beaumont Hospital describes that of 344 patients with early stage prostate cancer, only 7% had SV involvement beyond 1.0 cm (Kestin LL, Goldstein NS, Vicini FA et al, Treatment Of Prostate Cancer With Radiotherapy: Should The Entire Seminal Vesicles Be Included In The Clinical Target Volume? Int J Rad Oncol Biol Phys 54 (3): 686-697, 2002). The clinical target volume for all patients will include the proximal 1.0 cm of seminal vesicles. As in RTOG 9406 and at this study's initiation, patients treated with 3D CRT may have a clinical target volume reduction after 55.8Gy to the prostate.

<u>1.6 Collection Of Tissue For Translational Research</u>

The RTOG has been collecting pretreatment diagnostic tissue from all of the prostate cancer protocols over the last ten years. A number of histologic, cell kinetic/proliferation, and molecular markers are under investigation, with several showing promise for the stratification of patients in future trials. This large randomized study presents an excellent opportunity for the collection of diagnostic biopsy specimens that will be assayed for various cytogenetic or gene expression abnormalities. Correlating these findings with clinical outcome in a group of men treated with radiation alone may help increase our understanding of radiation sensitivity or resistance. Along these lines, prospective and retrospective translational components will be applied to this randomized dose trial.

The prospective component will include measurements of serum testosterone, percent free PSA, prostate volume (obtained through planning CT-scans), percent of diagnostic biopsies involved, percent of cancer in the diagnostic biopsies, perineural invasion, mitotic index, and DNA-ploidy. The rationale for these markers is briefly provided.

A high pretreatment serum testosterone level has been associated with an increased risk of distant metastasis in prostate cancer patients treated with radiotherapy.[21] The percent free PSA is an important aid in diagnosis and has potential as a prognostic factor; although it has not been investigated previously. Prostate volume will be used to examine PSA density[22] and Vca[23], both of which have never been assessed prospectively in a large prostate cancer trial. The histopathologic parameters mentioned above have all shown promise in the past.[24] The intent here is to request the collaborating pathologists at participating RTOG institutions and affiliates to record these parameters, with the goal of comparing these data with parallel central review. DNA-nondiploidy has repeatedly been shown to be a correlate of poor outcome; however, there are few studies involving radiotherapy therapy-treated patients.[24] An application for funding will be made for the DNA-ploidy analysis.

Retrospective analyses of several tissue biomarkers will also be performed. The biomarkers currently under study include Ki67, p53, bcl2, Bax, p21, pRb, p16, Cox-2, EBFR, and VEGF *(See reference 23 for review).* All of these markers show promise for providing prognostic information that compliments the standard clinical parameters of PSA, Gleason score and stage. Since diagnostic tissue will be limited, a final selection of the most promising markers will be made upon the completion of the ongoing studies involving the completed protocols 85-31, 86-10, 92-02, and 94-13. Approximately 7 years will be required for the proposed protocol to mature and by that time, a clearer definition of the markers to be studied will be evident. The goal will be to analyze approximately 8 biomarkers from the pretreatment diagnostic material.

<u>1.6.1 Single Nucleotide Polymorphisms (SNPs) and Normal Tissue Toxicity</u> (7/10/07)

RT produces its biological effects mainly through the generation of short lived but highly reactive DNA radicals that evolve into stable/long-lived DNA lesions such as DSBs[25] or through interactions with the plasma membrane,[26] leading to cell death. The total number of gene products currently known to be involved in determining cellular radiosensitivity is well over 100 and growing.[27] Several groups have reported analysis of genetic variants of individual candidate genes potentially implicated in normal tissue radiosensitivity.[28-29] A more powerful search approach, in the post-genome era, would be to screen patients for a large number of genes that could impact on radiosensitivity. Variations in the sequence of the human genome can comprise repeating sequences such as variable number of tandem repeats (VNTRs), short tandem repeats (STRs) and SNPs.[30] Although the human genome is ~99.9% identical

among individuals, the ~0.1% variations (the vast majority of which are SNPs) tend to be heritable and stable.[31] It is postulated that these variations in the genome explain phenotypic differences between individuals and may also serve as a genetic blueprint for susceptibility to disease and cellular responses to pharmacologic agents.[32-33] SNP-types associated with a higher risk of radiation-induced normal tissue toxicity would comprise a predictive molecular signature of radiation injury, and would have broad applicability in patient selection for radical radiotherapy.

Several groups have reported preliminary results in their analysis of the association between candidate SNPs and late toxicity after RT for breast cancer.[34-39] An association between TGFB1 -509T and +869C alleles and fibrosis was found by Quarmby et al, while Andreassen et al found TGFB1 position -509 and codon 10 to be associated with fibrosis. The latter study also found associations between other DNA damage-related SNPs (SOD2 (codon 16), XRCC3 (codon 241), XRCC1 (codon 399)) and clinical late toxicity. Recently, in a different breast cancer patient cohort, Andreassen et al[35] found statistically significant associations between the TGFB1 codon 10 Pro allele (P=0.005) as well as the TGFB1 position -509 T allele (P=0.018) and increased risk of late breast fibrosis as indicated by breast appearance. The functional significance of either the TGFB1 codon 10 Pro allele or the TGFB1 position -509 T allele is currently unclear. However, recently Andreassen et al[40] failed to replicate these earlier associations in a study where DNA was obtained from formalin fixed paraffin embedded tissue samples in a different cohort of breast cancer patients. In order to avoid false positive associations, SNP-association studies should be validated in larger, well-defined cohorts of homogeneously treated patients.

The correlation of SNPs and pelvic normal tissue toxicity was reported by De Ruyck et al,[41-42] who examined SNPs in XRCC1, XRCC3, TGFB1 position -509, TGFB1 codon 10 and OGG1. Patients with three or more risk alleles in XRCC1 and XRCC3 had a significantly increased risk of developing late pelvic GI/GU toxicity (odds ratio 10.10, p = 0.001). Damaraju et al[43] analysed 53 SNPs in BRCA1, BRCA2, ESR1, XRCC1, XRCC2, XRCC3, NBS1, RAD51, RAD52, LIGIV, HAP1, ATM, BCL2, TGFβ-1, MSH6, XPD (ERCC2), XPF (ERCC4), GRL, CYP1A1, CYP2C19, CYP3A5, CYP2D6, CYP11B2, and CYP17 genes from a cohort of 83 men who had undergone 3-dimensional conformal RT for prostate cancer. Significant univariate associations with late rectal or bladder toxicity (grade 2+) were found for XRCC3 A>G 5' UTR NT 4541, LIGIV T>C Asp568Asp, MLH1 C>T, Val219Ile, CYP2D6*4 G>A splicing defect, mean rectal and bladder dose, dose to 30% of rectum or bladder, and age <60 years. In a Cox multivariate analysis, significant associations with toxicity were found for LIGIV T>C, Asp568Asp; XPD G>A, Asp711Asp; CYP2D6*4 G>A, splicing defect; mean bladder dose >60 Gy, and dose to 30% of rectal volume >75 Gy. These data suggest an association between candidate SNPs and late pelvic radiation toxicity.

1.6.2 Proposal for Banking of Buffy Coat Specimens for SNP Analysis (7/10/07)

In order to search for a genomic signature correlated with a higher propensity to normal tissue radiation damage, it is appropriate to propose a broad-based genetic (SNP) analysis for candidate genes. The working hypothesis is that toxicity (rectum and/or bladder in the case of pelvic sites; skin and subcutaneous tissue in the case of breast) will be correlated to a patient's genetic makeup measured as SNPs in a select group of candidate genes. The criteria for selecting SNPs should be based on published evidence for the various genes implicated or previously demonstrated to be involved in RT-induced tissue damage and repair pathways. Genomic DNA for SNP analysis can be most effectively isolated from buffy coat leukocytes using standard procedures. Banking of buffy coat leukocytes can be performed at any time in the patient's trajectory, whether before, during, or after treatment.

1.7 Quality Of Life (9/18/03) (7/10/07)

As important as survival and disease endpoints are in prostate cancer, increasing attention is being directed at quality of life endpoints. Local therapies have become increasingly aggressive for earlier stages of disease. Dose escalation, albeit with a lower rate of complications than historical experience, needs to be tempered with the increased expectations and desire of patients to have cancer control without negatively impacting their quality of life. Eventually, patients will demand a therapy not based on clinical disease endpoints alone, but on how the therapy will affect their day-to-day activities. The treatment options for prostate cancer currently include watchful waiting, brachytherapy, conformal radiation therapy, radical prostatectomy, or any combination of these, with or without hormone therapy. The side effects, cost, and emotional impact of each of these may be dramatically different despite clear and convincing data to argue the clinical advantage of any of them. This study offers an excellent opportunity to assess the impact of two 3D-CRT regimens *(high dose versus standard dose).* Furthermore, this study will stratify patients by whether they will have radiation treatment planning and delivery done by conventional 3D-CRT or

IMRT. The ability of IMRT to spare more normal tissue from higher doses may allow this study to compare impact of dosimetry differences on quality of life.

One significant quality of life concern that has received much attention after prostate cancer therapy is erectile dysfunction *(ED).* Although the specific mechanism by which radiation therapy reduces erections is uncertain, it has been suggested that radiation therapy does not damage the corporal nerves, but rather it causes vascular damage. This, in time, interferes with penile hemodynamics, which results in ED, even though desire and sexual sensations may be present.[44] The prognosis of erectile function has been found to be related to a number of factors including radiation dose to the bulb of the penis, patient age, and pretreatment sexual function.

With respect to 3D-CRT and sexual function, Roach analyzed the data from RTOG 94-06 to assess the impact of dose to the penile bulb on erectile function.[45] Using dose and volume data stored in the 3D QA center database, the dose to the penile bulb was computed retrospectively on 39 previously-potent patients treated to the 68.4 Gy and 73.8 Gy dose arms. There was a significantly higher rate of ED at 2 years *(p=0.048, hazard ratio ~ 5.0)* in patients if they received a mean dose of more than 52.5 Gy to the penile bulb. In this trial, the penile bulb structure will be defined prospectively. Dose to that structure will be recorded along with all other dosimetric parameters. Given the close proximity of the penile bulb to the prostate gland, the patients in the high dose arm may receive a slightly, yet significant, higher dose to the bulb.

In a retrospective study of 287 patients diagnosed with prostate cancer in clinical stages A to C and treated with 3D-CRT to 6200 to 7380 cGy, 29% experienced ED. For patients older than 70 years, 39% experienced ED after treatment. At months 1, 20, 40, and 60, actuarial ED rates after 3D-CRT were 4%, 25%, 41%, and 47%, respectively. Factors identified as significant predictors of post-3D-CRT ED included pre-treatment ED, diabetes, coronary artery disease, and anti-androgen medication usage.[46]

The importance of sexual functioning as a quality of life issue should not be underestimated. In a study of 413 impotent men and 109 controls, satisfaction with sexual life was found to be a powerful predictor of satisfaction with life as a whole.[47] Further, the importance of sexual functioning as a major issue in patient decision making regarding prostate cancer treatment has been demonstrated. Quality versus quantity of life trade-offs have been documented in up to two-thirds of men with prostate cancer who are willing to accept at least a 10% decrement in survival for a treatment that offered a better chance of preserving erectile function.[48] The long-term impact of cancer treatments also highlights the consequences of cure on quality of life. One study, which used a battery of quality-of-life instruments, concluded that cancer survivors enjoy quality of life similar to their neighbors in all but one aspect of daily life: sexual functioning.[49]

The current study will seek to minimize erectile dysfunction after 3D-CRT by minimizing dose to the bulb of the penis in both arms of this dose escalation study. However, even with specifying the contours of the bulb of the penis, the dose escalation arm may receive bulb of penis doses > 52.5 Gy. To assess the impact radiation dose has on erectile function, we will compare outcomes between the two treatment groups. Associations between sexual and global quality of life will be assessed. The global quality of life measure, the Spitzer Quality of Life Index, also provides data from which to calculate utilities to be used in quality adjusted survival analysis. If there is a difference in symptom control or toxicities, then quality-adjusted survival within the first five years should be affected. Therefore, differences in quality-adjusted survival will be examined between the two treatment arms.

In addition to an impact on sexual function, 3D-CRT affects bowel and bladder function. Incontinence after radiation therapy, documented using patient self-report, has been reported in 2% to 15% of men compared with 35% to 52% of men receiving radical prostatectomy,[50-52] although a much smaller risk of other side effects exist that mainly include bowel sequelae such as diarrhea, proctitis, bleeding, perforation after radiation therapy[53-54], or rectal injury requiring colostomy after surgery[55-56]. In our work at Fox Chase Cancer Center, we recently quantified symptoms that impact bowel and bladder quality of life *(QOL)* in prostate cancer patients treated with 3D-CRT alone to the prostate +/- seminal vesicles versus whole pelvis. Second, bowel and bladder QOL measures for these patients were compared to that of the normal population of men with a similar age distribution Two health status surveys evaluating bowel and bladder functioning, along with the AUA Symptom Problem Index and the BPH Impact Index were mailed to 195 prostate cancer patients treated with 3D-CRT between December 1992 and November 1995 at Fox Chase by a single clinician *(GH).* No patient received hormonal management as part of their treatment. Ninety-five patients had pre-treatment PSA levels <10 ng/ml, T1/T2AB tumors with Gleason Scores 2-6, and no perineural invasion. They were treated to the prostate +/- seminal vesicles and are referred to as Group I. The remaining 100 patients had one or more of the following characteristics: pre-treatment PSA levels ≥10 ng/ml, T2C/T3 tumors, Gleason Scores 7-10, or perineural invasion. These patients were treated to the whole pelvis and are referred to as Group II. Overall percentages were compared to those for equivalent measures reported by Litwin *(1999)* based on a normal population

of men with a mean age of 72 years *(range 47-86).* The mailing yielded a high response rate of 62% *(n=120, 60 in each group).* The mean age was 68 *(range 52-82),* and the median ICRU dose levels for Groups I and II were 73 and 76 Gy, respectively.

Table 1 displays comparisons between the two prognosis groups for bowel and bladder QOL symptoms observed to be statistically different. Table 2 compares bowel and bladder symptoms in our radiation therapy series to that of the normal population. Despite reported symptoms, when asked about overall satisfaction in bowel and bladder functioning, 80% of the prostate cancer patients responded favorably. There was no significant difference in the bother that bladder symptoms cause men treated with radiotherapy as compared to men without cancer. No patients reported bowel dysfunction bother as a big problem, but patients do tend to have more very-small to moderate bother from bowel dysfunction than the normal population *(59% versus 33%).* In summary, this study demonstrates that these men have QOL related to bladder function similar to that of the normal population. Patients report no major bother from bowel symptoms, but tend to have more very-small to moderate bother than the normal population. Treatment of prostate cancer patients to the whole pelvis may result in decreased QOL as defined by rectal urgency, total procedures performed, and satisfaction with bowel functioning. However, regardless of field size, men are generally satisfied with their bowel and bladder functioning long term. However, as we evaluate dose escalation with 3D-CRT and IMRT**,** further study of the impact on QOL is required to document areas that need increased surveillance and targeted interventions.

Table 1.		Group I	Group II	p-value
Rectal urgency	Yes<br>No	25%<br>75%	41%<br>58%	.05
Total procedures†	0<br>1<br>2	35%<br>37%<br>28%	45%<br>17%<br>38%	.05
Number of BM/day	1<br>2<br>3+	44%<br>39%<br>17%	37%<br>25%<br>38%	.04
Bowel functioning	Satisfied<br>Not Satisfied	81%<br>11%	74%<br>26%	.05
Pain in urination	Yes<br>No	10%<br>90%	0%<br>100%	.03

†Flexible sigmoidoscopy, colonoscopy, fulguration, observation, or other.
‡Controls based on Litwin Study, Journal of Urology, 161, April 1999.

Table 2.		PC pts	Controls ‡	p-value
Pads/day to control urinary leakage	None<br>1-2	98%<br>2%	98%<br>2%	.99
Total urinary control	Yes<br>No	70%<br>30%	69%<br>31%	.91
Bother from urinary incontinence	No problem<br>Very small problem<br>Small problem<br>Moderate-big problem	61%<br>25%<br>12%<br>2%	60%<br>34%<br>6%<br>2%	.13
Bother from bowel dysfunction	No problem<br>Very small-small problem<br>Moderate problem<br>Big problem	41%<br>37%<br>22%<br>0%	66%<br>28%<br>5%<br>1%	.001
Rectal urgency	Yes<br>No	33%<br>67%	31%<br>69%	.71

2.0 OBJECTIVES

2.1 Primary Objective (9/18/03)

2.1.1 Determine whether 3D-CRT/IMRT to 79.2 Gy in 44 fractions will lead to improved overall survival in patients treated for prostate cancer compared to a group of patients treated with 3D-CRT/ IMRT to 70.2 Gy in 39 fractions.

2.2 Secondary Objectives (7/10/07)

2.2.1 Determine freedom from PSA failure *(ASTRO consensus definition; See Endpoints, Section 13.0),* disease-specific survival, local progression, and distant metastases.

2.2.2 Collect dose/volume data to allow tumor control probability and normal tissue complication probability modeling for patients treated with radiation therapy for prostate cancer.

2.2.3 Determine the incidence of grade 2 or greater GU and GI acute and late toxicity in patients treated with each of the regimens described above.

2.2.4 Prospectively collect quality of life data, including sexual function, to compare outcomes between the two treatment groups.

2.2.5 Prospectively collect diagnostic biopsy samples to determine the influence of histopathologic or tumor-specific cytogenetic or chromosomal markers on cancer control outcomes following radiation.

2.2.6 To collect paraffin-embedded tissue blocks, serum, plasma, and buffy coat cells for future translational research analyses.

3.0 PATIENT SELECTION

3.1 Conditions for Patient Eligibility

3.1.1 Histologically confirmed prostate adenocarcinoma within 180 days of randomization;

3.1.2 Zubrod Performance Scale 0-1;

3.1.3 Prostatic biopsy tumor grading by the Gleason Score Classification;

3.1.4 One of the following combinations of factors:

- Clinical stage T1b-T2b, Gleason score 2-6, and prostate-specific antigen ≥ 10 but < 20;
- Clinical stage T1b-T2b, Gleason score 7 and prostate-specific antigen < 15;

3.1.5 Pretreatment evaluations must be completed as specified in Section 4.1; (9/18/03)

3.1.6 Patients must sign a study-specific informed consent form prior to randomization.

3.2 Conditions for Patient Ineligibility

3.2.1 Evidence of distant metastases;

3.2.2 Regional lymph node involvement;

3.2.3 Previous radical surgery *(prostatectomy)* or cryosurgery for prostate cancer;

3.2.4 Previous pelvic irradiation, prostate brachytherapy, or bilateral orchiectomy;

3.2.5 Previous hormonal therapy, such as LHRH agonists *(e.g. goserilin, leuprolide),* anti-androgens *(e.g. flutamide, bicalutamide),* estrogens *(e.g. DES),* or surgical castration *(orchiectomy);*

3.2.6 Use of 5-alpha-reductase (*finasteride/dutasteride [Proscar])* or the phytoestrogen preparation "PC-SPES" within 3 months prior to registration; (4/18/06)

3.2.7 Previous or concurrent cytotoxic chemotherapy for this cancer;

3.2.8 Previous or concurrent invasive cancers, other than localized basal cell or squamous cell skin carcinoma, unless continually disease free for at least 5 years;

3.2.9 Major medical or psychiatric illness that, in the investigator's opinion, would prevent completion of treatment and interfere with follow up.

4.0 PRETREATMENT EVALUATIONS

Protocol treatment must begin within 4 weeks after registration. (9/18/03)

4.1 Evaluations Required for Eligibility (9/18/03) (9/17/04) (4/18/06)

4.1.1 Complete history, physical examination, and evaluation of Zubrod Performance Scale;

4.1.2 Histological evaluation of prostate biopsy with assignment of a Gleason score to the biopsy material;

4.1.3 Digital rectal examination of prostate; (4/18/06)

4.1.4 Radionuclide bone scan must be done if PSA > 10 AND Gleason score 7.

4.2 Other Pretreatment Evaluations (7/10/07)

4.2.1 Lymph node assessment is recommended by pre-registration diagnostic pelvic CT scan or MRI and/or pelvic lymphadenectomy;

4.2.2 Urethrogram is strongly encouraged at the time of simulation or CT scan for treatment*;*

4.2.3 PA and lateral chest X-rays are optional;

4.2.4 Quality of life evaluations: International Index of Erectile Function Questionnaire *(IIEF)* [PQ]; Functional Alterations due to Changes in Elimination *(FACE)* [FA]; The Spitzer Quality of Life Index *(SQLI)* [SP].

4.2.5 Laboratory evaluations to include CBC, platelets, BUN, creatinine, testosterone, serum free PSA *(if available),* and prostatic specific antigen *(PSA);* PSA must be done a) within 120 days prior to registration and prior to biopsy or b) within 120 days prior to registration and at least 10 days after prostate biopsy. (Every effort should be made to obtain all serum PSA values obtained in the 1 year prior to treatment to allow for calculation of PSA kinetics) The type of PSA assay (e.g., Abbott) should be recorded on the data forms. (4/18/06)

4.2.6 Serum, plasma, buffy coat cells, and archival tissue (preferably in blocks; see Section 10.3.2) for banking: For patients who consent to this component of the study.

5.0 REGISTRATION PROCEDURES (9/18/03)

5.1 Only institutions that have met the technology requirements and that have provided the baseline physics information that are described in 3D-CRT Quality Assurance Guidelines *(See Appendix VI*) or the IMRT Quality Assurance Guidelines (see Appendix VII) may enter patients to this study.

5.1.1. The 3D Questionnaire *(one per institution, see Appendix VI)* is to be sent to the Washington University Image-Guided Therapy Center *(ITC)* for review prior to entering any cases. Upon review and successful completion of "Dry-Run" or "Benchmark" QA test *(See Appendix VI),* the ITC will notify both the registering institution and RTOG Headquarters that the institution is eligible to enter patients onto this study. Institutions that have previously enrolled patients on RTOG 94-06 may enroll patients on this study without further credentialing by the ITC.

5.1.2 Institutions or investigators anticipating the use of IMRT on this study must complete a new IMRT Facility Questionnaire. A copy of the IMRT Facility Questionnaire may be obtained only via the world-wide web at the Image-Guided Therapy Center (ITC) website *http://itc.wustl.edu*. The IMRT Facility Questionnaire requests information regarding the training and experience of the IMRT team; IMRT treatment planning and treatment equipment; and in-house QA procedures. In addition, all institutions must successfully complete an IMRT "dry-run" or benchmark case with the ITC. In addition, an IMRT phantom study with the RPC (see Appendix VII) must be successfully completed if the institution has not previously met this credentialing requirement on another RTOG IMRT study.

5.2 Patients can be registered only after pretreatment evaluation is completed and eligibility criteria are met. Patients are registered prior to any protocol therapy by calling RTOG headquarters at *(215)* 574-3191, Monday through Friday, 8:30 a.m. to 5:00 p.m. ET. The patient will be randomized to a treatment arm and a case number will be assigned and confirmed by mail. The Eligibility Checklist must be completed in its entirety prior to calling RTOG. The completed, signed, and dated Checklist used at study entry must be retained in the patient's study file and will be evaluated during an institutional NCI/RTOG audit.

5.3 After the patient is randomized to a treatment arm, RTOG will notify the ITC *(by FAX)* providing the following information:

- Case Number
- Institution Name
- Institution Number
- Date of Registration
- Treatment Option
- Stratification Group

5.4 After the patient is randomized to a treatment arm, the institution will submit the required data *(both hardcopy and digital)* to the ITC *(See Section 12.2)* and to the RTOG *(See Section 12.1).*

6.0 RADIATION THERAPY [IMRT is allowed (9/18/03)]

6.1 Dose Specification (9/18/03)

6.1.1 The prescription dose is the minimum dose to the PTV *(defined in Section 6.4).* The maximum dose *to a volume of no more than 2% of the PTV* should not exceed the prescription dose by more than 7% *(inhomogeneity ≤ 7%)* and will be scored as no variation: ≤ 7%; minor variation: > 7 to ≤ 10%; major variation: > 10%. It is expected that IMRT may result in more heterogeneity in dose coverage than forward planned 3D-CRT. Minor variations as described are acceptable.

6.1.2 **Forward planned 3D-CRT or IMRT (9/18/03)**

Prescription dose to the PTV shall be according to the following dose schema delivered in 1.8 Gy minimum dose fractions. All fields treated once daily, 5 fractions per week.

ARM 1: 70.2 Gy in 39 Fractions. No more than 2% of the PTV and none of the CTV may receive less than 70.2 Gy.

ARM 2: 79.2 Gy in 44 Fractions. No more than 2% of the PTV and none of the CTV may receive less than 79.2 Gy.

Patients treated with forward planned 3D-CRT may have a clinical target volume reduction after the first 31 fractions (55.8Gy) that encompasses the prostate with a PTV margin only.

Dose Goal (Prescription)	Minimum PTV dose (encompassing ≥ 98% of PTV)	Minimum CTV dose (encompassing ≥ 100% of CTV)	Maximum PTV dose to ≤ 2% of PTV[1] (No variation)	Maximum PTV dose to ≤ 2% of PTV[1] (Minor variation)	Maximum PTV dose to ≤ 2% of PTV[1] (Major variation)
ARM 1	70.2 Gy	70.2 Gy	75.1 Gy	77.2 Gy	>77.2 Gy
ARM 2	79.2 Gy	79.2Gy	84.7 Gy	87.1 Gy	>87.1 Gy

[1] The maximum dose must not be within an "Organ at Risk" such as the Rectum, Bladder, or Penile Bulb

GTV=Prostate CTV = Prostate + *proximal* BSV*
PTV = CTV + 0.5-1.0 cm

CTV_{boost} = Prostate (GTV) (optional for non-IMRT 3D patients, only)
PTV_{boost} = CTV + 0.5-1.0 cm (optional for non- IMRT, forward planned 3D-CRT cases)

*BSV = bilateral seminal vesicles. The proximal seminal vesicle is defined as the portion from its origin with the prostate and extending 1.0 cm superiorly.

6.1.2.1 The reported doses shall include the dose to the ICRU Reference Point *(Section 6.4.4)* as well as the maximum point dose, maximum dose to 2% of PTV, minimum point dose in both CTV and PTV, and mean dose to PTV. **(9/18/03)**

6.2 <u>External Beam Equipment</u> (9/18/03)

6.2.1 Megavoltage equipment is required with effective photon energies ≥ 6 MV

6.2.2 Credentialing requirements and QA guidelines for institutions planning to participate in this study using 3D-CRT can be found on the ITC website (***http://itc.wustl.edu***) and are given in *Appendix VI.*

6.2.3 Credentialing requirements and QA guidelines for institutions planning to participate in this study using IMRT can be found on the ITC website (***http://itc.wustl.edu***) and are given in *Appendix VII.*

6.3 <u>Treatment Planning Imaging and Localization Requirements</u> (11/4/04)

6.3.1 A treatment planning CT scan will be required to define tumor, clinical, and planning target volumes and the critical structures *(See Section 6.4.5).* The treatment planning CT will be acquired with the patient in the same position and with immobilization device and conditions as will be utilized for treatment. That is, if treatment is planned with a full bladder, the simulation CT should be performed with a full bladder. The rectum should be empty *(except for contrast material for its visualization).* Each patient will be positioned in the supine position in an individualized thermoplastic immobilization cast or molded foam cradle in the treatment position on a flat tabletop. The CT scan of the pelvis should start at or above the iliac crest down to the perineum. All tissues to be irradiated must be included in CT scan. CT scan thickness should be ≤ 0.5 cm through the region that contains the target volumes *(i.e., from the bottom of the sacroiliac joints down to the penile urethra).* The regions above and below the target volume region may be scanned with slice thickness ≤ 1.0 cm.
The GTV, CTV, and PTV *(see Section 6.4)*, and normal tissues must be outlined on all CT slices in which the structures exist. For patients receiving forward planned 3D-CRT, beam's eye view display must be used to design beam aperture.

6.4 <u>Volume and ICRU Reference Point Definitions</u> The definition of volumes will be in accordance with the ICRU Report #50: Prescribing, Recording, and Reporting Photon Beam Therapy.

6.4.1 *<u>The Gross Tumor Volume</u>* ***<u>(GTV)</u>*** is defined by the physician as all known disease as defined by the planning CT, urethrogram, and clinical information. If a urethrogram is used, the GTV will encompass a volume inferiorly 5mm superior to the tip of the dye and no less than the entire prostate. Prostate dimensions should be defined as visualized on CT scan.

6.4.2 *<u>The Clinical Target Volumes</u>* ***<u>(CTV)</u>*** are the GTV plus areas considered to contain microscopic disease, delineated by the treating physician, and is defined as follows:

CTV is the GTV (*prostate*) plus the proximal bilateral seminal vesicles. Only the first 1.0 centimeter of seminal vesicle tissue adjacent to the prostate shall be included in the clinical target volume. This 1.0 cm of seminal vesicles refers to both radial (in plane) and superior (out of plane) extent. If both prostate and seminal vesicle are visualized in the same CT slice, this seminal vesicle tissue will contribute to the 1.0 cm of tissue. (9/18/03)

6.4.3 *<u>The Planning Target Volume</u>* ***<u>(PTV)</u>*** will provide a margin around the CTV to compensate for the variability of treatment set up and internal organ motion. A minimum of 5 mm around the CTV is required to define each respective PTV. Superior and inferior margins *(capping)* should be 5-10 mm depending on the thickness and spacing of the planning CT scan. Careful consideration should be made when defining the 5-10 mm margin in three dimensions.

It is advised that extreme bladder or rectal filling not be present at the time of the planning CT scan. A distended bladder or rectum can introduce a systematic error that may increase the probability of missing the CTV. An enema before the planning CT scan and use of a hollow *(robnel)* catheter to evacuate flatus will empty the rectum, thereby allowing a narrow posterior PTV margin *(~5 mm)* to account mainly for set up errors.

6.4.4 *<u>The ICRU Reference Points</u>* are to be located in the central part of the PTV and, secondly, on or near the central axis of the beams. Typically these points should be located <u>on</u> the beam axes or <u>at the intersection</u> of the beam axes.

6.4.5 *<u>Critical Normal Structures</u>* (9/18/03)

The normal tissue volume to be contoured will include bladder, rectum, bilateral femora *(to the level of ischial tuberosity)*, penile bulb, and skin. The normal tissues will be contoured and considered as solid organs. The bladder should be contoured from its base to the dome, and the rectum from the anus (*at the level of the ischial tuberosities*) for a length of 15 cm or to the rectosigmoid flexure. This generally is below the bottom of the sacroiliac joints. The tissue within the skin and outside all other critical normal structures and PTV's is designated as unspecified tissue. See the ITC web site to view examples of target and normal tissue contours.

The following table summarizes the naming of organs for submission of data to the ITC

Standard Name	Description
**BLADDER**	Bladder
**CTV**	Clinical Target Volume
**FEMUR_LT**	Left Femur
**FEMUR_RT**	Right Femur
**GTV**	Gross Tumor Volume (Prostate)
**PENILE_BULB**	Penile Bulb
**PTV**	Planning Target Volume
**RECTUM**	Rectum
**SKIN**	External patient contour
**SEM_VES**	Seminal Vesicles

<u>6.5 Treatment Planning (9/18/03)</u>

6.5.1 *<u>PTV</u>*

Treatment will be given only to the PTV using three dimensional conformal fields shaped to exclude as much of the bladder and rectum as possible. Field arrangements will be determined by 3D planning to produce the optimal conformal plan in accordance with volume definitions. The treatment plan used for each patient will be based on an analysis of the volumetric dose including dose-volume histogram *(DVH)* analyses of the PTV and critical normal structures.

6.5.2 *<u>Critical Normal Structures</u>*

Custom shielding must be used in conjunction with conformal planning to restrict the dose to the normal structures. Dose-volume histograms *(DVHs)* must be generated for all critical normal structures and the unspecified tissues (*see Section 6.4.5).* Portions of the bladder and rectum will, by necessity, receive the full dose to the PTV; however, careful 3D planning must be performed to ensure that the volume of the bladder and rectum receiving the full dose is kept to a minimum.

Based upon a review of patient dosimetry on dose level 3 of RTOG 94-06, the following normal tissue guidelines should be followed:

Normal organ limit†	No more than 15% volume receives dose that exceeds	No more than 25% volume receives dose that exceeds	No more than 35% volume receives dose that exceeds	No more than 50% volume receives dose that exceeds
Bladder Constraint	80 Gy	75 Gy	70 Gy	65 Gy
Rectum Constraint	75 Gy	70 Gy	65 Gy	60 Gy
Penile Bulb	Mean dose less than or equal to 52.5 Gy			

†Normal organ limit refers to the volume of that organ that should not exceed the dose limit. No part of these normal organs shall receive more than 77.2Gy on Arm 1 or on 84.7Gy on Arm 2.

Roach has reported a lower incidence of erectile dysfunction in patients who received a mean dose of 52.5 Gy or less to the penile bulb on RTOG 94-06. This dose value represents a treatment planning guideline and not a clinical study constraint. Care should be taken not to shield the penile bulb at the expense of adequate coverage of the PTV in this study.

6.6 Treatment Verification (9/18/03)

First day port films or portal images of each field along with orthogonal isocenter verification films *(or images)* must be obtained. If modifications are made in field shaping or design, a port film of each modified field along with orthogonal isocenter verification films *(or images)* is required on the first day's treatment of that field. Thereafter, weekly verification films or images of **orthogonal isocenter** views *(anterior to posterior and lateral projection)* are required. The required accuracy of patient positioning and the use of multi-leaf collimator apertures suggests the daily use of on-line imaging.

For IMRT the intensity profiles of each beam must be independently verified and compared to the planned field intensity. Portal films are not required for IMRT but orthogonal verification films are required, just as for 3D- CRT.

Real-time ultrasound localization is an important complement to conventional port films or portal imaging; however, there is some reluctance in a cooperative group setting to rely solely upon ultrasound localization to verify patient positioning. Therefore, until more data suggests otherwise, weekly port filming is required in this study, in addition to the use of ultrasound localization in those centers using that device.

6.7 Quality Assurance of Target Volumes and Critical Structure Volumes (9/18/03)

The ITC will facilitate the review of GTV, CTV, PTV, and designated organs at risk (critical structures) on, as a minimum, the first five cases submitted by each institution *(unless previously submitted on RTOG 94-06)*. After an institution has demonstrated compliance with the protocol, future cases will be randomly selected for review.

6.8 Quality Assurance of Field Placement (9/18/03)

The ITC will archive in an ITC database all digital treatment prescription and verification images (hard copy films will be digitized by the ITC) for later review by the study chair of initial placement films submitted by each institution. At least one port film or pretreatment alignment film per field along with the digital reconstructed radiographs *(DRRs)* from the treatment planning program or, alternatively, a simulation verification radiograph shall be submitted for evaluation except where geometrically impractical.

6.9 Quality Assurance of Dose Distribution

6.9.1 The ITC will display, and compare with hard copies, isodose distributions for the axial, and coronal planes *(or multiple axial planes as outlined in QA Guidelines; See Appendices VI and VII)* through the planning target volume to verify correct digital submission and conversion. (9/18/03)

6.9.2 The ITC will compare the submitted DVHs for the PTV, designated critical structures, and unspecified tissues with DVHs calculated by the ITC.

6.9.3 *Protocol Deviation* (9/18/03)

- No variation *(total coverage)*; Prescription isodose surface covers ≥ 98% of the PTV
- Minor variation *(marginal coverage)*; Prescription isodose surface coverage between ≥ 95% to < 98% of the PTV
- Major variation *(miss)*; Prescription isodose surface coverage < 95% of the PTV, or less than 100% of CTV

6.9.4 *Dose Heterogeneity* (9/18/03)

Maximum dose to ≤ 2% of the PTV volume should not exceed the prescription dose by more than 7% *(no variation: ≤ 7%; minor variation: > 7 to ≤ 10%; major variation: > 10%).* **This maximum dose volume of the PTV must not be shared by an "Organ at Risk"**. The maximum point dose to critical normal structures outside the PTV including the unspecified tissue should not exceed the prescription dose. The treating physician must carefully consider the tolerance dose/volume to each critical normal structure and unspecified tissue.

6.10 RTOG 3D-CRT Summary of 1993 ICRU Report 50 on Recommendations for Prescribing, Recording, and Reporting External Beam Radiation Therapy

6.10.1 Complete descriptions of volumes to be treated have been included in the 3D-CRT protocols in order to minimize the institutional variation of tumor and target volume delineation for protocol cases. Please consult the ICRU 1993 document for complete descriptions of the various target volumes defined. The next paragraphs summarize the ICRU definitions that are relevant for this protocol:

6.10.2 The gross tumor volume *(GTV)* includes the known disease as determined by physical examination, imaging studies and other diagnostic information; more than one GTV can be defined.

6.10.3 The clinical target volume *(CTV)* includes the area of subclinical involvement around the GTV. The CTV is the GTV plus the margin for micro extensions of the tumor; more than one CTV can be defined.

6.10.4 The planning target volume *(PTV)* is the CTV plus a margin to ensure that the prescribed dose is actually delivered to the CTV. This margin accounts for variations in treatment delivery, including variations in set-up between treatments, patient motion during treatment, movement of the tissues that contain the CTV *(e.g. respiration)*, and size variations in the tissue containing the CTV. The PTV is a geometric concept; more than one PTV can be defined.

6.11 R.T. Quality Assurance Reviews (7/10/07)

The Principal Investigator, Jeff Michalski, M.D., M.B.A., will remotely perform RT Quality Assurance Review after complete data for the first 25 cases enrolled have been received by the ITC (see section 12.0). Dr. Michalski will perform remote reviews on subsequent blocks of 25 cases after complete data for these cases have been received by the ITC. The final cases will be reviewed within 3 months after this study has reached the target accrual or as soon as complete data for all cases enrolled have been received, whichever occurs first.

6.12 Radiation Toxicity (7/10/07) (3/29/10)

6.12.1 All patients will be seen weekly by their radiation oncologist during radiation therapy. Any observations regarding radiation reactions will be recorded and should include attention toward the following potential side effects:

6.12.1.1 Small bowel or rectal irritation manifesting as abdominal cramping, diarrhea, rectal urgency, proctitis, or hematochezia

6.12.1.2 Bladder complications including urinary frequency/urgency, dysuria, hematuria, urinary tract infection, and incontinence

6.12.1.3 Radiation dermatitis

6.12.2 Clinical discretion may be exercised to treat side effects from radiation therapy. Rectal side effects such as diarrhea may be treated with Diphenoxylate or Loperamide. Bladder or rectal spasms can be treated with anticholinergic or Tolterodine. Bladder irritation can be managed with Phenazopyridine. Erectile dysfunction can be treated with Sildenafil.

6.12.3 Acute toxicity monitoring: Acute *(≤ 90 days from RT start)* side effects of radiation therapy were documented using the NCI Common Toxicity Criteria version 2.0.

6.12.4 Late toxicity monitoring: Beginning April 1, 2010, renal and GU toxicities will be evaluated and graded according to the NCI Common Terminology Criteria for Adverse Events (CTCAE) version 4.0. All appropriate treatment areas should have access to a copy of the CTCAE version 4.0. A copy of the CTCAE version 4.0 can be downloaded from the CTEP website (http://ctep.cancer.gov/protocolDevelopment/electronic_applications/ctc.htm). All other late *(> 90 days from RT start)* side effects will be evaluated and graded according to the RTOG Late Radiation Morbidity Scoring Scale *(Appendix IV).*

6.13 Toxicity Reporting Guidelines (7/10/07)

6.13.1 All fatal toxicities (*grade 5*) resulting from protocol treatment must be reported by telephone to the Group Chairman, to RTOG Headquarters Data Management, and to the Study Chairman within 24 hours of discovery.

6.13.2 All life-threatening (*grade 4*) toxicities from protocol treatment must be reported by telephone to the Group Chairman, RTOG Headquarters Data Management Staff, and to the Study Chairman within 24 hours of discovery.

6.13.3 Appropriate data forms, and if requested a written report, must be submitted to RTOG Headquarters within 10 working days of the telephone report, FAX # *(215)* 928-0153.

7.0 DRUG THERAPY (8/23/02)

7.1 Neoadjuvant or Adjuvant Hormone Therapy

Neoadjuvant or adjuvant hormone therapy is NOT allowed on this randomized trial. The eligibility criteria for this study were chosen to exclude those "intermediate risk" patients that benefit from the use of hormone therapy in conjunction with radiation therapy. This trial is seeking to measure the effects of two dose levels of radiation therapy on cancer control and toxicity. Non-protocol use of hormone therapy will confound the effects related to the study question.

8.0 SURGERY

Not applicable to this study.

9.0 OTHER THERAPY (8/23/02)

9.1 Subsequent Disease Progression

Treatment of patients who have failed by criteria described in Sections 11.6 *(Criteria for Local Control)* or 11.7 *(Criteria for Nonlocal Failure)* may receive additional medical or surgical therapies. The selection of these therapies will be left to the discretion of the treating physician. Treatments may include local salvage surgery or brachytherapy in pathologically confirmed, isolated local failures. If salvage local therapy is not available or not medically appropriate, patients with local failure may be observed or treated with salvage hormone therapy *(LHRH agonists, LHRH antagonists, castration, anti-androgens, or combinations of these)* or other systemic treatments *(chemotherapy, other new agents).* Patients with biochemical relapse or other non-local failures may be observed or treated with salvage hormone therapy or other systemic treatments.

9.2 Non-Permitted Supportive Therapy (7/10/07)

The use of avodart (dutasteride) is not permitted at any time while on study.

10.0 PATHOLOGY (8/23/02)

(For Patients Who Have Consented to Participate in the Tissue Component of the Study; see Appendix IB)

10.1 Central Review

10.1.1 The investigators at the treating institutions are **strongly encouraged** to recruit patient participation in the central review component of this trial. Slides/blocks from the pre-treatment diagnostic prostatic biopsy will be reviewed to confirm Gleason score and to record other histopathologic features, such as the extent of tumor in the biopsies, the number of biopsies positive, and mitotic index.

10.2 Collection of Tissue For Translational Research (7/10/07)

10.2.1 Biomarker studies are being done on all RTOG prostatic cancer protocols using the original diagnostic material. The emphasis has been on proliferation markers *(e.g., ki-67 and ploidy),* apoptotic pathway markers *(e.g., p53, bcl-2, bax),* and angiogenesis markers *(e.g., Cox-2, VEGF)* [*See section 1.6*]. These markers have shown promise in predicting prostate cancer patient outcome after radiotherapy. All of these markers, with the exception of DNA ploidy, will be determined by quantitative immunohistochemistry, as has been done for RTOG 86-10. A final decision on which markers will be studied awaits the results of completed RTOG prostate cancer trials that have reached maturity *(e.g., 86-10, 92-02, 94-13).* The trial described here will not be ready for biomarker analysis for approximately 7 years. The goal is to measure approximately eight biomarkers using the archived pathologic material.

Because genomic DNA for SNP analysis can be most effectively isolated from buffy coat leukocytes, these specimens will also be banked.

10.3 RTOG Tissue Bank

10.3.1 *Central Pathology Review*

All patients must have a least one H & E slide from each positive biopsy site submitted to the Tissue Bank for central pathology review. The following must be provided:

10.3.1.1 One H &E stained slide per positive biopsy site;

10.3.1.2 A Pathology report documenting that submitted blocks, core, or slides contain tumor; the report must include the RTOG protocol number and the patient's case number. The patient's name and/or other identifying information should be removed from the report;

10.3.1.3 A Pathology Submission Form clearly stating that the tissue is being submitted for the central review; the form must include the RTOG protocol number and patient's case number.

10.3.2 *Tissue Banking for Biomarker Studies* **(7/10/07)**

The investigators at the treating institutions are **strongly encouraged** to recruit patient participation in the translational research component of this trial. The following must be provided in order for the case to be evaluable for the Tissue Bank:

10.3.2.1 At least one paraffin-embedded tissue block of the tumor *(containing the highest grade of tumor if multiple biopsy sites contain cancer)*, a 2 mm core of tumor from the block, obtained with a derm punch or similar device, or 15 unstained slides. Kits for punching blocks can be obtained free of charge from the Tissue Bank (See Appendix VIII). Blocks/core/slides must be clearly labeled with the pathology identification number that agrees with the pathology report;

10.3.2.2 A Pathology Report documenting that submitted blocks, core, or slides contain tumor; the report must include the RTOG protocol number and patient's case number. The patient's name or other identifying information should be removed from the report;

10.3.2.3 A Specimen Transmittal Form clearly stating that tissue is being submitted for the RTOG Tissue Bank; if for translational research, this should be stated on the form. The form must include the RTOG protocol number and patient's case number;

10.3.2.4 Serum, plasma, and buffy coat cells

See Appendix IX for the blood collection kits and instructions.

The following must be provided in order for the case to be evaluable for the Tissue Bank: A Specimen Transmittal Form documenting the date of collection of the serum, plasma, and buffy coat cells; the RTOG protocol number, the patient's case number, and method of storage, for example, stored at -20° C, must be included.

10.3.2.5 A copy of the patient's tissue consent form.

10.3.3 *Specimen Collection Summary* **(7/10/07) (10/25/07)**

**Specimens for Tissue Banking**			
**Specimens taken from patient:**	**Specimens collected when:**	**Submitted as:**	**Shipped:**
A paraffin-embedded tissue block of the primary tumor taken before initiation of treatment or a 2 mm diameter core of tissue, punched from the tissue block with a skin punch	From pretreatment biopsy	Paraffin-embedded tissue block or punch biopsy	Block or punch shipped ambient
5-10 mL of whole blood in red-top tube and centrifuge for serum	Pretreatment	Frozen serum samples containing a minimum of 0.5 mL per aliquot in 1 mL cryovials	Serum sent frozen on dry ice via overnight carrier
5-10 mL of anticoagulated whole blood in EDTA tubes (purple/lavender top) and centrifuge for plasma	Pretreatment	Frozen plasma samples containing a minimum of 0.5 mL per aliquot in 1 mL cryovials	Plasma sent frozen on dry ice via overnight carrier
5-10 mL of anticoagulated whole blood in EDTA tubes (purple/lavender top) and centrifuge for buffy coat	Pre-treatment	Frozen buffy coat samples in 1 mL cryovials	Buffy coat sent frozen on dry ice via overnight carrier
5-10 mL of anticoagulated whole blood in EDTA tubes (purple/lavender top) and centrifuge for buffy coat	*Mid RT treatment	Buffy coat samples into three (3) 1 mL cryovials.	Buffy coat sent frozen on dry ice via overnight carrier
5-10 mL of anticoagulated whole blood in EDTA tubes (purple/lavender top) and centrifuge for buffy coat	**Next follow-up treatment visit	Buffy coat samples into three (3) 1 mL cryovials.	Buffy coat sent frozen on dry ice via overnight carrier

*During Week 4 for Arm 1 patients; during Week 5 for Arm 2 patients.

**For patients enrolled on study prior to Amendment 6 (previous protocol Version Date April 18, 2006) and who have signed the consent form for blood banking.

10.3.4 Submit materials *(for central review or tissue banking)* to: (9/18/03) (9/17/04) (4/18/06) (7/10/07)

LDS Hospital
RTOG Tissue Bank, 1st Floor North
8th Ave & C Street
Salt Lake City, UT 84143
(801) 408-5626; (801) 408-2035
FAX (801) 408-5020
RTOG@intermountainmail.org

10.4 Reimbursement (7/10/07)

10.4.1 RTOG will reimburse submitting institutions $300 per specimen for serum, plasma, and buffy coat cells; $200 per case if a block or core of material is submitted; and $100 per case if unstained slides are submitted. After confirmation from the Tissue Bank that appropriate materials have been received, RTOG Administration will prepare the proper paperwork and send a check to the institution.

10.5 Confidentiality *(See RTOG Patient Tissue Consent Frequently Asked Questions, http://www.rtog.org/tissuebank/tissuefaq.html for further details)* (9/18/03)

10.5.1 Upon receipt, the specimen is labeled with the RTOG protocol number and the patient's case number only. The Tissue Bank database only includes the following information: the number of specimens received, the date the specimens were received, documentation of material sent to a qualified investigator, type of material sent, and the date the specimens were sent to the investigator. No clinical information is kept in the database.

10.5.2 The specimens will be stored for an indefinite period of time. If at any time the patient withdraws consent to store and use specimens, the material will be returned to the institution that submitted it.

11.0 PATIENT ASSESSMENTS (7/10/07)

11.1 Study Parameters (9/18/03) (4/18/06) (7/10/07)

				Follow up *(Interval in months)*							
Parameters	Pre-Entry	Weekly During RT	During Week 4 or 5 of RT	3	6	9	12	15	18	21	24[g]
History, Physical Exam	X	X		X	X	X	X	X	X	X	X
Zubrod Performance Scale	X	X		X	X	X	X	X	X	X	X
Prostate biopsy with Gleason score[j]	X										
CBC, platelets	X	X[f]									
BUN, creatinine	X										
Testosterone	X										X
PSA	X[a,]			X	X	X	X	X	X	X	X
Free PSA (if available)	X[e]						X				X
Lymph node assessment	X[b]										
Radionuclide bone scan[k]	X[c]										
Digital examination	X			X	X	X	X	X	X	X	X
Urethrogram	X[d]										
Toxicity Evaluation		X		X	X	X	X	X	X	X	X
PA and Lateral chest x-rays	X[e]										
Tissue for banking	Recommended										
Blood for banking	Recommended[l]		Recommended[l]								
International Index of Erectile Function Questionnaire **[PQ]**	X				X		X				X[i]
Functional Alterations due to Changes in Elimination **[FA]**	X			X	X	X	X		X		X[h]
Spitzer Quality of Life Index **[SP]**	X			X	X	X	X		X		X[h]

a. PSA must be done within 120 days prior to registration and prior to biopsy or at least 10 days after prostate biopsy; Every effort should be made to obtain all serum PSA values obtained in the 1 year prior to treatment to allow for calculation of PSA kinetics.

b. Recommended by pre-registration diagnostic pelvic CT scan or MRI and/or pelvic lymphadenectomy;
c. Must be done if PSA > 10 and Gleason Score 7; (9/18/03)
d. Strongly encouraged at the time of simulation or CT scan for treatment planning;
e. Optional
f. As indicated
g. Follow up will continue every 6 months for the next 3 years then annually thereafter.
h. Continue every six months through year 5
i. Continue annually through year 5
j. Biopsy also recommended at time of treatment failure *(See Sections 11.3.4, 11.6.2, and 11.6.3)*
k. Must be done if patient develops a PSA *(biochemical)* failure or when patient develops symptoms suggesting metastatic disease; (9/18/03)
l. For currently enrolled patients, these specimens may be collected at the next follow-up visit. (7/10/07). For new patients receiving Treatment 1, blood should be drawn during week 4; for new patients receiving Treatment 2, blood should be drawn during week 5.

11.2 Evaluation During Treatment

11.2.1 Patients will be seen and evaluated at least weekly during radiation therapy with documentation of tolerance, including acute reactions.

11.3 Evaluation Following Treatment

11.3.1 At each visit *(See Section 11.1)* the patient will have an interval history, complete physical examination *(including digital rectal examination)* and assessment of specific GU and GI morbidity. (9/18/03)

11.3.2 PSA will be drawn at each follow-up visit: 3 months after RT, then every 3 months for 2 years, then every 6 months for 3 years, then annually. Free PSA *(if available)* will be collected at 12 and 24 months after RT.

11.3.3 A bone scan will be performed if the patient develops a PSA *(biochemical)* failure, or when the patient develops symptoms suggesting the presence of metastatic disease.

11.3.4 A needle biopsy is encouraged — from the site of original tumor within the prostate and/or other site of original tumor identified by the transrectal ultrasound, as indicated for rising PSA or clinical failure.

11.4 Quality of Life and Utility Assessments

11.4.1 International Index of Erectile Function Questionnaire *(IIEF)* [PQ]

The IIEF[58] was developed as a measure of erectile function. Relevant cross-cultural domains of sexual function were identified via the literature and were reviewed and endorsed by an international panel of experts. The resulting fifteen-item questionnaire underwent linguistic validation in ten languages. Psychometric testing was conducted, and a principal components analysis identified five factors with eigenvalues greater than 1.0: erectile function, orgasmic function, sexual desire, intercourse satisfaction, and overall satisfaction. Internal consistency was high with Cronbach's alphas for the five domains ranging from .73 to .92 with an overall alpha of .91. Scale reliability was determined with high test-retest correlation coefficients ranging from $r = 0.64$ to $r = 0.84$ depending on the domain. Discriminant validity was demonstrated by the scales' ability to differentiate between patients with ED and age-matched controls. IIEF was positively correlated with clinical interviews of sexual function but not with measures of marital adjustment and social desirability, exhibiting acceptable convergent and divergent validity. Sensitivity and specificity were demonstrated with those patients responding to ED treatment over time showing significant change while patients who did not respond to treatment showed no change in IIEF scores.[58]

11.4.2 Functional Alterations due to Changes in Elimination *(FACE)* [FA]

FACE is a 15-item Likert-type self-rating scale designed to measure the construct of intrusion on daily functioning caused by changes in elimination as measured by two subscales. Dimensions contributing to intrusion include control, fear, anxiety, and interference with activities. The total score for FACE is reflected in a low score being better than a high score. FACE is an expansion of Changes in Urinary Function (CUF) developed by Bruner.[59] CUF was developed specifically for use in prostate and bladder cancer clinical trials. In addition to CUF, a second subscale labeled Changes in Bowel Function (CBF) has been developed. The first subscale has undergone preliminary psychometric testing in 33 patients with either prostate or bladder cancer treated on RTOG 91-16, a Quality of Life evaluation of patients treated for bladder cancer, and RTOG 90-20 a prostate cancer protocol. Internal consistency was demonstrated with a standardized Chronbach's alpha of 0.85 indicating the scale to be reliable. Factor analysis showed all items loaded on one factor with item-total correlations ranging from 0.66 to 0.87, demonstrating reliability and construct validity. Content validity was determined by a panel of experts. Convergent validity was evaluated by examining the association between scores on CUF and similar

measures on a second quality of life instrument measured at the same time, The Functional Assessment of Cancer Therapy – Bladder (FACT-BL). Pearson Correlation with FACT questions ranged from -0.35 to -0.79. A small subset of patients had CUF scores at three time points. The average CUF score for these patients, all of whom had prostate cancer and high Karnofsky scores (90-100), was 6.6 initially, 7.0 and 7.3 at 3 and 6 months respectively. Future testing with both subscales and a larger, more diverse sample is planned to evaluate responsiveness to change over time and to further corroborate validity.

11.4.3 **The Spitzer Quality of Life Index *(SQLI)* [SP]** is a five item categorical questionnaire with three item response options scored from 0-2 and summed in a Likert format with total scores ranging from 0-10. The reliability and validity have been established.[60] The SQLI has been criticized because it does not assess symptoms, which is the reason for pairing it with the patient self-assessment of skin reactions. The SQLI has been used in testicular patients,[61] gastric patients,[62] terminal patients,[63] glioma patients,[64-65] lung, ovary, and breast cancer patients[66] The SQLI has been applied as both a rater-assessed form and a patient self-assessment form. We will be using the SQLI as a patient self-assessment form. In addition to providing a global measure of quality of life, researchers have developed a conversion factor that uses the SQLI data to produce utilities.[67] The utilities derived from the SQLI will be used to analyze quality-adjusted survival within the first five years on study. SQLI is assessed every six months in order to document transitions in health states for quality-adjusted survival.

11.5 Criteria for Toxicity

11.5.1 *Acute toxicity monitoring*: Acute side effects *(≤ 90 days of treatment start)* will be documented using the using the NCI Common Toxicity Criteria version 2.0.

11.5.2 *Late toxicity monitoring*: Renal and GU toxicities will be evaluated and graded according to the NCI Common Toxicity Criteria version 2.0. All other late *(> 90 days from RT start)* side effects will be evaluated and graded according to the RTOG Late Radiation Morbidity Scoring Scale *(Appendix IV).*

11.6 Criteria for Local Control

11.6.1 *PSA failure* is defined as having 3 consecutive elevations of post-treatment PSA or starting hormones after one or more elevations in post-treatment PSA but before three consecutive elevations are documented. The failure day is the midpoint between last non-rising PSA and first PSA rise. The ASTRO definition[68] of rising PSA will be used. The presence of palpable disease must be recorded on the data collection forms for initial and follow-up evaluations of the patient. All PSA levels done during a follow-up interval will be recorded on the data forms.

11.6.2 *Clinical criteria* for local failure are progression (increase in palpable abnormality) at any time, failure of regression of the palpable tumor by two years, and redevelopment of a palpable abnormality after complete disappearance of previous abnormalities. Needle biopsy is recommended.

11.6.3 *Histologic criteria* for local failure are presence of prostatic carcinoma upon biopsy and positive biopsy of the palpably normal prostate more than two years after the start of treatment.

11.7 Criteria for Nonlocal Failure

Other types of failure will be documented as follows:

11.7.1 Distant metastasis will be documented if clinical or bone scan evidence is demonstrated. Ultrasound evaluation of the prostate with needle biopsy as indicated by the findings is recommended at the time distant metastasis is reported.

11.7.2 *Time to Distant Failure*: The time to distant failure will be measured from the date of randomization to the date of documented regional nodal recurrence or distant disease relapse. Patients with evidence of biochemical failure, but a negative prostate biopsy, will be considered as distant failure only.

11.7.3 *Disease-Specific Survival*: Disease-specific survival duration will be measured from the date of randomization to the date of death due to prostate cancer. Causes of death may require review by the study chair or their designee. Death due to prostate cancer will be defined as:

11.7.3.1 Primary cause of death certified as due to prostate cancer.

11.7.3.2 Death in association with any of the following conditions:

- Further clinical tumor progression occurring after initiation of "salvage" anti-tumor *(e.g., (androgen suppression)* therapy.
- A rise *(that exceeds 1.0 ng/ml)* in the serum PSA level on at least two consecutive occasions that occurs during or after "salvage" androgen suppression therapy.
- Disease progression in the absence of any anti-tumor therapy.

11.7.3.3 Death from a complication of therapy, irrespective of disease status.

11.7.4 *Overall Survival*: Survival duration will be measured from the date of randomization to the date of death from any cause. A post-mortem examination will be performed whenever possible and a copy of the final post-mortem report will be sent to RTOG Headquarters.

12.0 DATA COLLECTION (6/12/14)

(NRG Oncology, 1818 Market Street, Philadelphia, PA 19103 FAX# 215 928-0153) (9/17/04)

12.1 Summary of Data Submission (9/18/03) (4/18/06)

Item	Due
Demographic Form **(A5)**<br>Initial Evaluation Form **(I1)**<br>Pathology Report **(P1)**<br>Slides/Blocks **(P2)**<br>International Index of Erectile Function Questionnaire **[PQ]**<br>Functional Alterations due to Changes in Elimination **[FA]**<br>The Spitzer Quality of Life Index **[SP]**	Within 2 wks of study entry
<u>Preliminary Dosimetry Information:</u>	See Section 12.2
<u>Final Dosimetry Information:</u><br>Radiotherapy Form **(T1)**<br>***(Copy to RTOG HQ and ITC)***	See Section 12.2<br>Within 1 wk from end of RT
Initial Follow-up Form **(FS)**<br>Functional Alterations due to Changes in Elimination **[FA]**<br>The Spitzer Quality of Life Index **[SP]**	90 days from start of treatment
Follow-up Form **(F1)**	6, 9, 12 months in year 1; q 3 months in year 2; q 6 months x 3 years, then annually; Also at progression/relapse and at death.
International Index of Erectile Function Questionnaire **[PQ]**	6 and 12 months from start of treatment; then annually through year 5
Functional Alterations due to Changes in Elimination **[FA]**<br>The Spitzer Quality of Life Index **[SP]**	6, 9, 12, 18, and 24 months; then every 6 months through year 5
Autopsy Report **(D3)**	As applicable

12.2 Summary of RT QA Requirements *(submit to Washington University via ATC website at http://atc.wustl.edu)* (11/24/04)

**Preliminary Dosimetry Information:**<br>Digital Patient Submission Information Form **(T2)**<br>CT data, critical normal structures, all GTV, CTV and PTV contours<br>Simulation films and/or digital film images for all initial treatment fields and orthogonal set up pair<br>First day port films *(or digital images)* of all initial treatment fields and orthogonal set up pair<br>Digital beam geometry for initial and boost beam sets<br>Doses for initial and boost sets of concurrently treated beams	Within 1 week of start of RT

Digital DVH data for all required critical normal structures, GTV, CTV, and PTVs for total dose plan
Hard copy isodose distributions for total dose plan as described in the QA Guidelines

Final Dosimetry Information: — Within 1 week of RT end

Copy of Radiotherapy Form **(T1)**
Daily Treatment Record
Simulation films and/or digital film images for all *(or digital images)* of all boost treatment fields and orthogonal set up pair
First day port films of all boost treatment fields and orthogonal setup pair
Modified digital patient data as required through consultation with Image Guided Therapy QA Center

12.2.1 *For Mail or Federal Express* (9/18/03) (4/18/06) (7/10/07)

Roxana Haynes
QA Dosimetry Specialist
Image-Guided Therapy Center *(ITC)*
Washington University School of Medicine
4511 Forest Park, Suite 200
St. Louis, MO 63108
***(314)*747-5415; FAX *(314)* 747-5423**

12.2.2. *To send over Internet or using magnetic tape* (9/18/03)
Digital data submission may be accomplished using magnetic tape or the Internet. For network submission, the *ftp* account assigned to the submitting institution shall be used and *e-mail* identifying the data set*(s)* being submitted shall be sent to:

itc@castor.wustl.edu

For tape submission, please contact the ITC about acceptable tape types and formats.

13.0 STATISTICAL CONSIDERATIONS (7/10/07)

13.1 Study Endpoints

13.1.1 *Primary Endpoint*

- Overall survival *(Failure: death from any cause; See Section 11.7.4)*

13.1.2 *Secondary Endpoints*

- PSA Failure[68]: Failure is defined as having 3 consecutive elevations of post-treatment PSA or starting hormones after one or more elevations in post-treatment PSA but before three consecutive elevations are documented. The failure day is the midpoint between last non-rising PSA and first PSA rise *(See Section 11.6.1)*.
- Disease-Specific Survival (*Failure: a competing risk cause of death not necessarily due to disease-specific cause; See Section 11.7.3*)
- Local Progression *(See Section 11.6)*
- Distant Metastases *(See Section 11.7)*
- Incidence of grade 2 or greater GU and GI toxicity
- Tumor Control Probability
- Normal Tissue Complication Probability
- Erectile function
- Global quality of life
- Quality-adjusted survival
- Correlation of biomarkers with PSA failure
- Correlation of biomarkers with overall survival

- To collect paraffin-embedded tissue block, serum, plasma, and buffy coat cells for future translational research analyses (7/10/07)

13.2 Sample Size

13.2.1 *Stratification*: Patients will be stratified before randomization with respect to PSA and Gleason score: Gleason score 2-6 and PSA ≥ 10 but < 20 vs. Gleason score 7 and PSA <15, and Radiation Modality: 3D-CRT vs. IMRT. The treatment allocation scheme described by Zelen[69] will be used because it balances patient factors other than institution. Patients will be randomized to MTD 3D-CRT or IMRT dose *(79.2 Gy)* vs. standard dose 3D-CRT or IMRT *(70.2 Gy).* (9/18/03)

13.2.2 *Sample Size Derivation*: The sample size calculations will address the specific primary hypothesis that the maximally tolerated dose of 3D-CRT/IMRT delivered to patients with localized adenocarcinoma of the prostate will result in a hazard reduction of 23% in the mortality rate. Roach et al.[70] identified four prognostic risk groups using independent predictors of death: combined Gleason score (*centrally reviewed*), T stage, and pathologic lymph node status. These groups were defined by observing the correlation between these factors and disease-specific survival. The five, ten and fifteen-year overall survival rates by risk group are presented in Table 1 below. Based on these risk groups, the probability of surviving in a five-year interval *(1-5, 6-10, 11-15)* is shown in Table 2. A preliminary analysis of RTOG 94-08, which is a two-arm study of endocrine therapy used as a cytoreductive and cytostatic agent prior to radiation therapy *(RT)* in locally confined adenocarcinoma of the prostate, showed a five-year overall survival rate of 74% for patients with clinical stage T1b-T2b, combined Gleason scores 2-6 and PSA <20 treated with radiation therapy alone. Forty-four percent of the men entered on RTOG 94-08 had a combined Gleason score between 2-6 and a PSA value ≥ 10 but < 20 while 56% had a Gleason of 7 and PSA < 15. In light of these data, the survival estimates based on Roach et al[70] risk group two were used for the sample size calculations. The five and ten-year overall survival rates used were 80% and 48%, respectively. The hazard rates of the control arm are assumed to be constant within each five-year interval (*1-5, 6-10, and 11-15*) and they are 0.0446, 0.1022, and 0.1386, respectively. Five interim significance tests and a final test are planned. Early testing will employ a nominal significance level of 0.001 while a level of 0.020 will be used at the final analysis to preserve a 0.025 level for the study[71]. Using Lakatos' method[72] for time-dependent mortality rates, 715 deaths are required to detect a survival benefit translating into a hazard ratio (Δ) of 1.3 with 90% statistical power using a one-sided test at a level of significance of 0.025. This level of 0.025 is the same as a level of significance that would be required for a two-sided test of significance at 0.05 to show the experimental arm is better than the control arm. Using the projected hazard rates for each five-year interval, we will require a total sample size of 1410 to be accrued uniformly over five years with an additional eight years of follow-up. Guarding against an ineligibility or lack-of-data rate up to 8%, **the final targeted accrual for this study will be 1520 cases**. Some of the secondary endpoints that will be evaluated are PSA failure, disease-specific survival, and quality-adjusted survival. The planned PSA failure analysis after the 7th year is discussed in more detail in section 13.4.3.2. (9/18/03)

Table 1. Overall survival rates by risk group

Risk Group	5-year	10-year	15-year
1	85%	59%	39%
2	82%	50%	24%
3	68%	32%	16%
4	52%	19%	12%*

* Extrapolated from 13-year results

Table 2. Probability of surviving during five-year interval by risk group

Risk Group	1-5	6-10	11-15
1	.85	.69	.66
2	.82	.61	.48
3	.68	.47	.50
4	.52	.37	.63*

13.2.3 *Erectile Function and Quality of Life*

The International Index of Erectile Function Questionnaire *(IIEF)* is the primary measure for erectile function *(ED).* IIEF question number 1 *("How often were you able to get an erection during sexual activity?")* is scored from: none/almost never *(response 0-1)* or ≤ half the time *(response 2-3)* to most times/almost always/always *(response 4-5).* A response of 0 to 3 on question number 1 of the IIEF will be considered erectile dysfunction. If the expected rate of ED at 12 months is 29%, then 688 patients per arm is sufficient for 90% statistical power and a two-sided significance level *(0.05)* to detect a reduction in ED to 19%. This assumes that 26% of the patients will have ED prior to the start of therapy and 80% compliance at 1 year. Only patients that respond 4-5 on question number 1 of the IIEF will be considered for this endpoint. The subset of patients age 70 and above will also be examined. Assuming 39% will have ED at 12 months, then this study will have the same statistical parameters to detect a reduction in ED to 25%, assuming that 50% of the sample is age 70 and above.

Quality of life will be measured using the Spitzer Quality of Life Index *(SQLI).* A 0.5 point difference in SQLI scores has been shown to be clinically meaningful,[73] assuming a standard deviation of 0.54 for SQLI. If ED reduces quality of life, then the difference in quality of life between the arms only may occur in this subgroup. In addition, ED may cause a reduction in quality of life greater than the minimally important difference; assuming the effect is 50% greater, then a 0.75 difference is expected. However, only those patients with ED may substantially impair quality of life. Therefore, the proportion of patients with ED may reduce the observable difference in quality of life. Using the above estimates of ED and sample size, this study will have 90% statistical power to find an observable difference of 0.12 in SQLI between the two arms with a significance level of 0.05 *(two-sided).*

13.2.4 *Quality-Adjusted Survival*

The SQLI generates a maximum of 125 health states. The utilities for these health states has been obtained by Weeks et al.[67] The SQLI will be assessed prior to the start of therapy, at 6, 9, 12, 18, and 24 months, then every six months through year five. The utility will then be multiplied by the duration in a health state, which will produce the quality-adjusted survival over the first five years. Patients will be assumed to be in a health state until a change in status is noted. An effect size of 0.20 will be examined with 90% statistical power and 0.05 *(two-sided)* level of significance.

13.3 Patient Accrual

This study will require a five-year accrual period with approximately 304 entries per year. The total duration of the study is expected to be thirteen years from the time the first patient is entered to the "final" overall survival analysis. If the average yearly accrual falls below 200 cases after 18 months, the feasibility of continuing the study will be discussed at the RTOG Data Monitoring Committee (*DMC*).

13.3.1 *Feasibility*: RTOG has an excellent history for successfully completing major Phase III prostate trials. One recently completed trial of significance is RTOG 94-13. It was a four-arm Phase III trial comparing definitive whole pelvic irradiation to irradiation to the prostate only and comparing neoadjuvant to adjuvant Total Androgen Suppression *(TAS).* Some of the eligibility requirements for participation in the study were: confirmed localized adenocarcinoma of prostate with an elevated PSA, estimated risk of lymph node involvement > 15%, no involved common iliac or para-aortic nodes, pathological lymph-node-positive patients are ineligible, PSA ≤ 100 and no prior or concurrent hormonal therapy, radiation or chemotherapy. RTOG 94-13 opened for accrual on April 4, 1995 and closed to accrual on June 1, 1999. During that time period, 1323 men were entered with an accrual rate of 26.5 patients per month.

RTOG 94-08 is a two-arm study of endocrine therapy used as a cytoreductive and cytostatic agent prior to radiation therapy *(RT)* in locally confined adenocarcinoma of the prostate. Patients were randomized to receive either neoadjuvant TAS two months before or during RT, or to radiation therapy alone. Some of the eligibility requirements are: confirmed localized adenocarcinoma of prostate, clinical stage T1b-2b, no involved nodes, PSA ≤ 20 and no prior antiandrogen therapy, radiation, or chemotherapy, and no radical surgery or cryosurgery for the prostate. RTOG 94-08 was opened for accrual on October 31, 1994 and closed on April 30, 2001. As of April 30, 2001, there were 2028 patients entered with an accrual rate of 26 patients per month.

RTOG 94-06 was a dose escalation study of 3D conformal radiation therapy for prostate cancer from which the total dose for this proposed trial was to be chosen. Thirty-four institutions were credentialed by the Image-Guided Therapy *(formerly 3DQA)* Center *(ITC)* in St. Louis to participate in this trial and 28 of

these participated in the last two dose levels of RTOG 94-06. The average monthly accrual to RTOG 94-06, for patients whose eligibility is compatible with this proposed study, was 16.2 per month for dose level IV and 25.8 per month for dose level V. As 3D conformal radiation therapy becomes more widely used, more RTOG institutions will become credentialed by the ITC, and therefore, this proposed Phase III trial will have no problem reaching its targeted monthly accrual of 26 men.

13.4 Analysis Plan (6/12/14)

13.4.1 *Statistical Methods*: Gelman[74] and Gaynor[75] have shown that the Kaplan Meier method tends to overestimate cumulative failure probabilities for events with competing risks, such as time to PSA failure, disease-specific survival and time to distant metastases. Thus, the cumulative incidence approach will be used to estimate these endpoints as a function of time, since this approach specifically accounts for competing risks such as dying without a recurrence from prostate cancer. The distributions between the two arms will be compared by a method developed by Gray.[76] Overall survival probabilities will be estimated by the usual Kaplan-Meier method. The survival distributions between the two arms will be compared using the log rank test. Factors associated with time to failure endpoints will be analyzed using the Cox regression model. Tumor control probability and normal tissue complication probability will be examined by categorical modeling techniques.

13.4.2 *Interim Analysis to Monitor the Study Progress*: Interim reports with statistical analyses will be prepared twice a year until the initial paper reporting the treatment results has been submitted.

In general, the interim reports will contain information about the patient accrual rate with a projected completion date for the accrual phase; data quality; compliance rate of treatment delivery with the distributions of important prognostic baseline variables; and the frequencies and severity of the toxicities by treatment arm. The interim reports will not contain the results from the treatment comparisons with respect to efficacy endpoints such as overall survival or PSA failure rate.

13.4.3 *Significance Testing for Early Termination and Reporting:*

13.4.3.1 *Primary Endpoint: Overall Survival*

Five interim significance tests of treatment difference are planned. The first interim analysis will be performed for the first RTOG semi-annual meeting after the 4th year of accrual. After the 5th year, it is projected that 100% of the target sample size has been achieved. The second interim analysis will be performed for the first RTOG semi-annual meeting one year after the last patient has been randomized. The third, fourth, and fifth interim analysis will be performed approximately eight, ten, and twelve years, respectively, from the start of the trial. The results will be reported to the NRG Oncology Data Monitoring Committee (DMC) with the treatment arms blinded.

The maximum number of deaths required for the study is 715. Under the alternative hypothesis given above, the projected numbers of deaths at the time of these five interim analyses are approximately 85, 186, 320, 478, and 640. The corresponding nominal significance level for each interim analysis will be 0.001. If the difference is significant at that level, the study statistician will recommend to the DMC that the randomization be discontinued *(if applicable)* and the study be considered for early publication.

At each planned interim analysis, the p-value from the log-rank test for assessing treatment efficacy, and the conditional power[77] for the alternative hypothesis given the observed data will be reported to the NRG Oncology DMC. A low conditional power indicates a small probability of a significant treatment effect if future follow-up events are assumed to follow the same distribution under the alternative hypothesis. The responsible statistician may recommend early reporting of the results and/or stopping the trial if the treatment effect, with respect to overall survival, is highly significant, i.e. the p-value is less than the nominal value specified in a sequential design, or if the conditional power is less than 10%. Before making such a recommendation, the accrual rate, treatment compliance, safety of the treatments, and the importance of the study are also taken into consideration with the p-value and conditional power. The DMC will then make a recommendation about the trial to the group chair.

13.4.3.2 *Secondary Endpoint: PSA Failure*

PSA failure will be a secondary endpoint of interest and will be reported early before the final overall survival result. The analysis of PSA failure will take place after the 7th year, approximately two years after accrual is completed. Pollack and his colleagues[10] reported a five-year PSA-free failure rate of 69% for standard 70 Gy dose as compared to 79% for 78 Gy 3D-CRT. These results in the appropriate prognostic groups have been substantiated by Zelefsky and Hanks.[2, 3] The range of differences has varied from 10% to 25%. In light of Pollack's study, power was calculated for different hazard ratios for the analysis of PSA failure using the estimated sample size. The baseline PSA failure rate was

0.074 per year and was assumed to be constant over time. It was also assumed that the frequency of deaths without a PSA failure reported was 2% per year and was incorporated into Lakatos' method when calculating the number of PSA failures and statistical power. Table 3 below lists the number of PSA failures and power at the 7th year analysis time point for each hazard ratio (Δ=*1.3, 1.4, 1.45, 1.5, and 1.57*). At the time of the PSA failure analysis, there will be at least 80% power to detect a hazard ratio greater than or equal to 1.4. The nominal significance level at this analysis is 0.025 The PSA results will be reported following this analysis. Treatment toxicity and quality of life (QOL) results will also be reported.

Table 3. Power of significance testing for early reporting of PSA Failure

Δ	PSA Failures	Power
1.30	338	67%
1.40	331	83%
1.45	326	90%
1.50	321	95%
1.57	316	98%

†Hazard ratio based on Pollack's results: 0.074 failure rate per year with standard dose compared to a 0.047 failure rate per year for 78.0 Gy 3D-CRT dose.

13.4.4 *Analysis For Reporting the Initial Treatment Results*: **(9/18/03)** This major analysis will occur after each patient has been potentially followed for a minimum of eight years for the primary endpoint, overall survival, and for a minimum of five years for the secondary endpoint, PSA failure, unless the early stopping rule is satisfied. It will include tabulation of all cases entered and those excluded from the analyses with the reasons for such given; the distribution of the important prognostic baseline variables; and observed results with respect to all the above mentioned endpoints. The primary hypothesis for the study is whether the higher total dose of 3D-CRT/IMRT will improve overall survival. All eligible patients randomized will be included in the comparison and will be grouped by assigned treatment in the analysis. The primary hypothesis of MTD 3D-CRT/IMRT treatment benefit will be tested using the Cox proportional hazard model with the major stratification factors included as fixed covariates. Additional analyses of treatment effect will include modifying factors such as age, race, and other patient characteristics. These analyses will also use the Cox proportional hazards model. The treatment comparison of time to PSA failure and disease-specific survival will be analyzed in a similar fashion. The treatment comparison of 2+ grade toxicity rates will use the z-statistic for testing binomial proportions. Also, where feasible, treatment comparisons with respect to time to PSA failure and overall survival will be compared within each ethnic category. Secondary analyses of the primary and secondary endpoints will be performed using the available Gleason scores from central review. Comparison of the proportion of patients with ED at one year by treatment arm will be performed using a chi-square test. Other comparisons will be made for subsets defined by the dose to the penis bulb and by age (*<70 vs. ≥ 70 years old*) respectively. Quality of life change scores from pretreatment to one year will be compared between treatment arms using a t-test. Quality of life will be correlated with responses on the IIEF. The z-test will be used to compare between treatment arms the average quality-adjusted survival within the first five years. An area under the curve analysis will be performed to compare the bowel and bladder symptoms assessed using FACE over the first five years.

13.4.5 *Revised Interim Futility Analysis Plan* **(6/12/14)**

As described in Section 13.4.3.1, there will be five interim analyses of the primary hypotheses, at the increments of the requisite events for definitive analysis indicated. The interim futility analysis plan calls for evaluation of whether conditional power falls below 10% at each of those interim analyses. The following revised futility monitoring plan will be carried out for remaining interim analyses.

Interim futility analysis will be based on the lower inefficacy boundary (LIB) rule of Freidlin, Korn, and Gray[78]. This rule provides the opportunity to terminate early for evidence that the experimental arm will not prove superior, but protects against aggressive early termination for treatment effect sizes smaller than planned. For implementation, we chose the LIB40 rule as suggested for a moderately aggressive futility stopping criterion in late follow-up. Note that futility monitoring does not apply before the time to commence such monitoring derived from the formula given in Freidlin, Korn and Gray, and in any case, those interim analyses have already been completed.

The following table summarizes the interim futility monitoring schedule:

Analysis	Prop. Total events	Cumulative Total Events (Both Arms)	Futility boundary: stop if		
			**Z<**	**P>**	**HR (Exp/Control) >**
Interim 1	0.12	85	NA	NA	NA
Interim 2	0.26	186	NA	NA	NA
Interim 3	0.45	320	0.126	0.450	0.986
Interim 4	0.67	478	0.564	0.286	0.950
Interim 5	0.90	640	1.124	0.131	0.915
final	1.00	715	-	-	-

13.5 Inclusion of Minorities

In conformance with the national Institutes of Health *(NIH)* Revitalization Act of 1993 with regard to inclusion of women and minorities in clinical research, we address this issue here, as we will also analyze treatment differences in this male cohort by ethnicity. Based on previous RTOG prostate protocol data the minority enrollment for this study is expected as follows:

Ethnic Group	Number	Percent (%)
American Indian or Alaskan Native	3	0.2
Asian	8	0.5
Black or African American	228	15.0
Hispanic or Latino	46	3.0
Native Hawaiian or other Pacific Islander	4	0.3
White	1216	80.0
Other/Unknown	15	1.0
**Total**	**1520**	**100**

Obviously with 80% of the available sample being white, univariate comparisons by treatment will not yield sufficient power in most cases. We will, however, also include the ethnicity variable in all regressions including the Cox model.

13.6 Tumor Marker Evaluation In addition to the clinical endpoints, this study also will seek to answer translational questions with the idea of generation hypotheses for further testing. Each marker will be evaluated with respect to its prognostic value on PSA failure and overall patient survival. There will be 2 separate analyses, the first occurring when the PSA failure data from the clinical study is mature and the second when the survival data is mature.

13.6.1 *Correlation of Biomarkers with PSA Failure*

We wish to determine the prognostic impact of each marker on time to PSA failure. This analysis will take place when each patient potentially has been followed for a minimum of 5 years, to correspond with the major clinical analysis of PSA failure. Multivariate Cox models will be used to evaluate the prognostic significance of each marker. The model will contain treatment and the stratifying variables (*combined Gleason score/PSA and Radiation Modality*) as fixed covariates. (9/18/03)

13.6.2 *Correlation of Biomarkers with Overall Survival*

We wish to determine the prognostic impact of each marker on time to overall survival. This analysis will take place when each patient has been potentially followed for a minimum of 8 years, to correspond with the major clinical analysis of the primary endpoint, overall survival. Multivariate Cox models will be used to evaluate the prognostic significance of each marker. The model will contain treatment and the stratifying variables *(combined Gleason score/PSA and Radiation Modality)* as fixed covariates. (9/18/03)

REFERENCES <u>(6/12/14)</u>

1. Michalski JM, Purdy JA, Winter K, Roach III M, Vijayakumar S, Sandler HM, Markoe AM, Ritter MA, Russell KJ, Sailer S, Harms WB, Perez CA, Wilder RB, Hanks GE, Cox JD, Preliminary Report of Toxicity Following 3D Radiation Therapy for Prostate Cancer on 3DOG/RTOG 94-06. *Int J Rad Onc Biol Phys*. 46 (2): 391–402, 2000.
2. Michalski JM, Winter K, Purdy JA, et al. Update of toxicity following 3D radiation therapy for prostate cancer on RTOG 94-06. *Int J Rad Onc Biol Phys.* 48: 228 (Supplement 1), 2000.
3. Ryu, J., K. Winter, J. Michalski, et al. Interim Report of Toxicity from 3D Conformal Radiation Therapy *(3DCRT)* for Prostate Cancer on 3DOG/RTOG 9406, Level III *(79.2 GY)*. Proceeding from Am Soc Clin Oncol *(ASCO),* San Francisco, CA, Am Soc Clin Oncol, 2001.
4. Hanks GE. Leibel SA. Krall JM. Kramer S. Patterns of care studies: dose-response observations for local control of adenocarcinoma of the prostate. *Int J Rad Onc Biol Phys.* 11(1):153-7, 1985.
5. Zelefsky MJ, Leibel SA, et al. Dose escalation with three-dimensional conformal radiation therapy affects the outcome of prostate cancer. *Int J Rad Onc Biol Phys*. (41) 3: 491-500, 1998.
6. Zelefsky MJ, Cowen D, Fuks Z, et al. Long term tolerance of high dose three-dimensional conformal radiotherapy in patients with localized prostate carcinoma. *Cancer*. 85: 2460-2468, 199
7. Lee WR, Hanks GE, Hanlon AL, Schultheiss TE, Hunt MA. Lateral rectal shielding reduces late rectal morbidity following high dose three-dimensional conformal radiation therapy for clinically localized prostate cancer: Further evidence for a significant dose effect. *Int J Rad Onc Biol Phys*. 35(2): 251-7, 1996.
8. Schulteiss TE, Lee WR, Hunt MA, Hanlon AL, Peter RS, Hanks GE. Late GI and GU complications in the treatment of prostate cancer. *Int J Rad Onc Biol Phys*. 37(1): 3-11, 1997.
9. Perez CA, Pilepich MV, Zivnuska F. Tumor control in definitive irradiation of localized carcinoma of the prostate. *Int J Rad Onc Biol Phys.* 12:523–531, 1986.
10. Pollack A, Zagars GK. External beam radiotherapy dose response of prostate cancer. *Int J Rad Onc Biol Phys*. (39) 5: 1011-1018, 1997.
11. Kupelian PA, Mohan DS, Lyons J, Klein EA, Reddy CA. Higher than standard radiation doses (>72 Gy) with or without androgen deprivation in the treatment of localized prostate cancer. *Int J Rad Onc Biol Phys*., 46 (3), 567–574, 2000.
12. Fuks Z, Leibel SA, Wallner KE, et al. The effect of local control on metastatic dissemination in carcinoma of the prostate: Long-term results in patients treated with 125-I implantation. *Int J Rad Onc Biol Phys*. 21:537-547, 1991.
13. Hanks GE, Hanlon AL, Schultheiss TE, Pinover WH, Movsas B, Eptstein BE, Hunt MS. Dose escalation with 3D conformal treatment: Five-year outcomes, treatment optimization, and future directions. *Int J Rad Onc Biol Phys*. 41:501-510, 1998.
14. Perez CA, Michalski JM, Purdy JA, Wasserman TH, Williams K, Lockett MA, Three-Dimensional Conformal Therapy or Standard Irradiation in Localized Carcinoma of Prostate: Preliminary Results of a Nonrandomized Comparison, *Int J Rad Onc Biol Phys*. 47, (3): 629–637, 2000.
15. Smit WG. Helle PA. van Putten WL. Wijnmaalen AJ. Seldenrath JJ. van der Werf-Messing BH. Late radiation damage in prostate cancer patients treated by high dose external radiotherapy in relation to rectal dose *Int J Rad Onc Biol Phys.* 18(1):23-9, 1990.
16. Sandler HM, McLaughlin WP, Ten Haken RK, *et al.* Three dimensional conformal radiotherapy for the treatment of prostate cancer: Low risk of chronic rectal morbidity observed in a large series of patients. *Int J Radiat Onc Biol Phys* 33: 797– 801, 1995
17. Pollack A, Zagars GK, Smith LG, Lee JJ, von Eschenbach AC, Antolak JA, Starkschall G, Rosen I, Preliminary Results of a Randomized Radiotherapy Dose-Escalation Study Comparing 70 Gy With 78 Gy for Prostate Cancer, *J Clin Oncol*, 18 (23): 3904-3911, 2000.

18. Algan O, Pinover WH, Hanlon AL, Al-Saleem TI, Hanks GE. Is there a subset of patients with PSA > or = 20 ng/ml who do well after conformal beam radiotherapy? *Rad Onc Investigations*. 7(2):106-10, 1999.

19. Pinover WH, Hanlon AL, Horwitz EM, Hanks GE Defining the appropriate radiation dose for pretreatment PSA ≤10 ng/mL prostate cancer. *Int J Rad Onl Biol Phys*. 47:649-54, 2000.

20. Fowler J, Chappell R, Ritter M. Is alpha/beta for prostate tumors really low? *Inr J Rad Onc Biol Phys.* 50 (4):1021-1031, 2001.

21. Zagars GK, Pollack A, von Eschenbach AC. Serum testosterone—A significant determinant of metastatic relapse for irradiated localized prostate cancer. *Urology*. 49:327-334, 1997.

22. D'Amico AV, Propert KJ. Prostate cancer volume adds significantly to prostate-specific antigen in the prediction of early biochemical failure after external beam radiation therapy. *Int J Radiat Oncol Biol Phys.* 35:273-279, 1996.

23. Pollack A, Lankford S, Zagars GK, Babaian RJ. PSA density as a prognostic factor in prostate cancer patients treated with radiotherapy. *Cancer*. 77:1515-1523, 1996.

24. Pollack A, Zagars GK. In: Greco C, Zelefsky MJ, eds. *Radiotherapy of Prostate Cancer.* Singapore: Harwood Academic Publishers; 2000: 113-128.

25. Leadon SA Repair of DNA damage produced by ionizing radiation: a mini review. Semin Radiat Oncol 6, 295-305, 1996.

26. Haimovitz-Friedman A Radiation-induced signal transduction and stress response. Radiat Res 150, S102, 1998.

27. Murray D, Begg AD. DNA repair genes and radiosensitivity. In: LC Panasci and MA Alaoui-Jamali (eds.), DNA Repair in Cancer Therapy, Humana Press, Totawa, NJ, pp. 211-256, 2004.

28. Green H et al. Variation in the manganese superoxide dismutase gene (SOD2) is not a major cause of radiotherapy complications in breast cancer patients. Radiother Oncol 63, 213-216, 2002.

29. Severin DM et al. Novel DNA sequence variants in the hHR21 DNA repair gene in radiosensitive cancer patients. Int J Radiat Oncol Biol Phys 50, 1323-1331, 2001.

30. Brookes AJ. The essence of SNPs. Gene, 234, 177-186, 1999.

31. Stoneking M. Single nucleotide polymorphisms. From the evolutionary past. Nature 409, 821-822, 2001.

32. Damaraju S et al. Genomic approaches to clinical drug resistance. In: B. Anderson and D. Murray, (eds.) Clinically Relevant Resistance in Cancer Chemotherapy. pp.347-372, 2002.

33. Riley JH et al. The use of single nucleotide polymorphisms in the isolation of common disease genes. Pharmacogenomics 1, 39-47, 2000.

34. Quarmby S, et al. Association of transforming growth factor beta-1 single nucleotide polymorphisms with radiation-induced damage to normal tissues in breast cancer patients. Int J Radiat Biol 79(2):137-43, 2003.

35. Andreassen CN et al. Prediction of normal tissue radiosensitivity from polymorphisms in candidate genes. Radiother Oncol 69(2):127-35, 2003.

36. Andreassen CN et al. TGFβ1 polymorphisms are associated with risk of late normal tissue complications in the breast after radiotherapy for early breast cancer. Radiother Oncol 75:18-21, 2005.

37. Cesaretti JA, Stock RG, Lehrer S, Atencio DA, Bernstein JL, Stone NN, Wallenstein S, Green S, Loeb K, Kollmeier M, Smith M, Rosenstein BS. ATM sequence variants are predictive of adverse radiotherapy response among patients treated for prostate cancer. Int J Radiat Oncol Biol Phys 2005 Jan 1;61(1):196-202.
38. Andreassen CN, Alsner J, Overgaard J, Herskind C, Haviland J, Owen R, Homewood J, Bliss J, Yarnold J. TGFB1 polymorphisms are associated with risk of late normal tissue complications in the breast after radiotherapy for early breast cancer. Radiother Oncol 2005 Apr;75(1):18-21.

39. Andreassen CN, Overgaard J, Alsner J, Overgaard M, Herskind C, Cesaretti JA, Atencio DP, Green S, Formenti SC, Stock RG, Rosenstein BS. ATM sequence variants and risk of radiation-induced subcutaneous fibrosis after postmastectomy radiotherapy. Int J Radiat Oncol Biol Phys 2006 Mar 1;64(3):776-83.

40. Andreassen CN, Alsner J, Overgaard M, Sorensen FB, Overgaard J. Risk of radiation-induced subcutaneous fibrosis in relation to single nucleotide polymorphisms in TGFB1, SOD2, XRCC1, XRCC3, APEX and ATM--a study based on DNA from formalin fixed paraffin embedded tissue samples. Int J Radiat Biol 2006 Aug;82(8):577-86.

41. De Ruyck K, Van Eijkeren M, Claes K, Morthier R, De Paepe A, Vral A, De Ridder L, Thierens H. Radiation-induced damage to normal tissues after radiotherapy in patients treated for gynecologic tumors: association with single nucleotide polymorphisms in XRCC1, XRCC3, and OGG1 genes and in vitro chromosomal radiosensitivity in lymphocytes. Int J Radiat Oncol Biol Phys 2005 Jul 15;62(4):1140-9.

42. De Ruyck K, Van Eijkeren M, Claes K, Bacher K, Vral A, De Neve W, Thierens H. TGFbeta1 polymorphisms and late clinical radiosensitivity in patients treated for gynecologic tumors. Int J Radiat Oncol Biol Phys 2006 Jul 15;65(4):1240-8.

43. Damaraju S, Murray D, Dufour J, Carandang D, Myrehaug S, Fallone G, Field C, Greiner R, Hanson J, Cass CE, Parliament M. Association of DNA repair and steroid metabolism gene polymorphisms with clinical late toxicity in patients treated with conformal radiotherapy for prostate cancer. Clin Cancer Res 2006 Apr 15;12(8):2545-54.

44. Goldstein I, et al. Radiation associated impotence: A clinical study of its mechanism. *JAMA.* 251: 903-910, 1984.

45. Roach M, Winter K, Michalski J, Bosch W, Lin X. Mean dose of radiation to the bulb of the penis correlates with risk of erectile dysfunction at 24 months: Preliminary analysis of Radiation Therapy Oncology Group *(RTOG)* phase I / II dose escalation trial 94-06. *J Rad Onc Biol Phys*. 48:3, Supplement 1, 2000.

46. Mantz CA, et al. Potency preservation following conformal radiotherapy for localized prostate cancer: Impact of neoadjuvant androgen blockade, treatment technique, and patient-related factors. *Cancer J Scientific America.* 5(4): p. 230-236, 1999.

47. Fugl-Meyer AR, et al. On life satisfaction in male erectile dysfunction. *International J Impotence Res.* 9(3): 141-148, 1997.

48. Singer PA, et al. Sex or survival: Trade-offs between quality and quantity of life. *J Clin Onc.* 9(2): 328-334, 1991.

49. Olweny CLM. et al. Long-term effects of cancer treatment and consequences of cure: Cancer survivors enjoy quality of life similar to their neighbors. *Euro J Cancer*. 29A(6): p. 826-830, 1993.

50. Schrader-Bogen CL, Kjellberg J L, McPherson C P, Murray C L. Quality of life and treatment outcomes: Prostate carcinoma patients' perspectives after prostatectomy or radiation. *Cancer*. 79(10): 1977-1986, 1997.

51. Talcott J A, Rieker P, Clark J A, et al. Patient-reported symptoms after primary therapy for early prostate cancer: Results of a prospective cohort study. *J Clin Onc*. 16(1): 275-283, 1998.

52. Yarbro CH, Ferrans CE. Quality of life of patients with prostate cancer treated with surgery or radiation therapy. *Onc Nurs Forum.* 25(4): 685-693, 1998.

53. Lawton CA, Won M, Pilepich MV, et al. Long-term treatment sequelae following external beam irradiation for adenocarcinoma of the prostate: Analysis of RTOG studies 75-06 and 77-06. *Int J Rad Onc Biol Phys*. 21(4): 935-939, 1991.

54. Mameghan H, Fisher R, Mameghan J, Watt WH, Tynan A. Bowel complications after radiotherapy for carcinoma of the prostate: The volume effect. *Int J Rad Onc Biol Phys*. 18: 315-320, 1990.

55. Gibbons RP, Correa RJ, Brannen GE, Weissman, RM. Total prostatectomy for clinically localized prostatic cancer: Long-term results. *Urology*.141(3): 564-566, 1989.

56. Middleton RG, Smith Jr JA, Melzer RB, Hamilton PE. Patient survival and local recurrence rate following radical prostatectomy for prostatic carcinoma. *Urology*. 136(2): 422-424, 1986.

57. Hanlon A, Bruner DW, Hanks G. Long term quality of life study in prostate cancer patients treated with three dimensional conformal radiation therapy: Comparing bowel and bladder quality of life symptoms to that of the normal population. *Int J Rad Onc Biol Phys*. 49(1): 51-59, 2001.

58. Rosen, RC, et. al. The International Index of Erectile Function (IIEF): A multidimensional scale for assessment of erectile dysfunction. *Urology.* 49(6): 822-830, 1997.

59. Bruner DW, Scott C, Byhardt R, Coughlin C, Friedland J, DelRowe J. Changes in urinary function (CUF): Pilot data on the development and validation of a measure of the intrusion on quality of life caused by changes in urinary function after cancer therapy, (RTOG 91-16, 90-20). Presented at the Drug Information Association 2nd Symposium on Quality of Life Evaluation, Charleston, SC, 1994.

60. Spitzer WO, Dobson AJ, Hall J, Chesterman E, Levi J, Shepherd R, Battista RN, Catchlove BR. Measuring the quality of life of cancer patients:A concise QL-index for use by physicians. *J Chronic Dis.* 34(12): 585-97, 1981.

61. Tamburini M, Filiberti A, Barbieri A, Zanoni F, Pizzocaro G, Barletta L, Ventafridda V. Psychological aspects of testis cancer therapy: a prospective study. *J Urol*. 142(6): 1487-9, 1989.

62. Buhl K, P Schlag, C Herfarth. Quality of life and functional results following different types of resection for gastric carcinoma. *Eur J Surg Oncol*. 16:404-409, 1990.

63. Addington-Hall JM, MacDonald LD, Anderson HR. Can the Spitzer Quality of Life Index help to reduce prognostic uncertainty in terminal care? *Br Jcancer*. 62(4):695-9, 1990.

64. Awwad S, Cull A, Gregor A. Long-term survival in adult hemispheric glioma: prognostic factors and quality of outcome. *Clin Oncol* (R Coll Radiol). 2(6):343-6, 1990.

65. Bauman GS, Gaspar LE, Fisher BJ, Halperin EC, Macdonald DR, CairncrossJG. A prospective study of short- course radiotherapy in poor prognosis glioblastoma multiforme. *Int J Radiat Oncol Biol Phys*. 29:835-839, 1994.

66. Ovesen L, Allingstrup L, Hannibal J, Mortensen EL, Hansen OP. Effect of dietary counseling on food intake, body weight, response rate, survival, and quality of life in cancer patients undergoing chemotherapy: A prospective, randomized study. *J Clin Onc*. 11:2043-2049, 1993.

67. Weeks JC, O'Leary J, Fairclough D, Paltield Weinsten. The "Q-tility Index": A new tool for assessing health related quality of life and utilities in clinical trials and clinical practice. *Proc ASCO.* 13:436, 1994.

68. ASTRO consensus statement: Guidelines for PSA following radiation therapy. *Int J Radiat Oncol Biol Phys*. 37:1035-1041, 1997.

69. Zelen M. The randomization and stratification of patients to clinical trials. *J Chron Dis*. 27: 365-375, 1974.

70. Roach M, Lu J, Pilepich MV, et al. Four prognostic groups predict long-term survival form prostate cancer following radiotherapy alone on Radiation Therapy Oncology Group clinical trials. *Int J Radiat Oncol Biol Phys*. 47:609-615, 2000.

71. Freidlin B, Korn EL, George SL. Data monitoring committees and interim monitoring guidelines. *Control Clin Trials.* 20:395-407, 1999.

72. Lakatos, E. Sample size determination in clinical trials with time-dependent rates of losses and noncompliance. *Controlled Clin Trials*. 7:189-199, 1986.

73. Scott C, Paulus R, Stevens R, Marconi B, Champion L, Freedman G, et al. Is radiation-induced skin toxicity of the breast associated with reduced quality of life: Report of RTOG 97-13. Proc from Int Soc Qual of Life Research. 8:572, 1999.

74. Gelman R, Gelber R, Henderson IC, et al. Improved methodology for analyzing local and distant recurrence. *J Clin Oncol*. 8: 548-555, 1990.

75. Gaynor JJ, Feuer EJ, Tan CC, et al. On the use of cause specific failure and conditional failure probabilities: Examples from clinical oncology data. *J Amer Statist Assoc*. 88: 400-409, 1993.

76. Gray RJ. A class of K-sample tests for comparing the cumulative incidence of a competing risk. *Ann Statist*. 16: 1141-1154, 1988.

77. Andersen PK. Conditional power calculations as an aid in the decision whether to continue a clinical trial. *Control Clin Trials.* 8:67-74, 1987.

78. Freidlin B, Korn EL, Gray R. A general inefficacy interim monitoring rule for randomized clinical trials. *Clin Trials*. 2010;7(3):197-208.

APPENDIX IA

RTOG 0126 (10/18/04)

SAMPLE CONSENT FOR RESEARCH STUDY

STUDY TITLE (9/18/03)

A PHASE III RANDOMIZED STUDY OF HIGH DOSE 3D-CRT/IMRT VERSUS STANDARD DOSE 3D-CRT/IMRT IN PATIENTS TREATED FOR LOCALIZED PROSTATE CANCER

This is a clinical trial (a type of research study). Clinical trials include only patients who choose to take part. Please take your time to make your decision. Discuss it with your friends and family. The National Cancer Institute (NCI) booklet, "Taking Part in Clinical Trials: What Cancer Patients Need To Know," is available from your doctor.

You are being asked to take part in this study because you have prostate cancer.

WHY IS THIS STUDY BEING DONE? (9/18/03)

One of the standard treatment options for your stage and type of prostate cancer is external beam radiation therapy. Modern radiation therapy planning methods with 3-dimensional therapy or Intensity Modulated Radiation Therapy (IMRT) allow safer delivery of higher than conventional doses of radiation. The purpose of this study is to compare the effects (good and bad) on you and your cancer of the standard dose of radiation therapy (39 treatments) with a higher dose of radiation (44 treatments) to see which treatment is better.

Both three-dimensional radiation therapy and IMRT allow the radiation beam to treat an area that is shaped like your tumor and also to penetrate as deeply as your tumor is located. By treating this way, the dose of radiation to the healthy areas near your tumor is minimized, and the dose to your tumor is maximized. This research is being done because doses higher than the standard dose of radiation therapy may better control your cancer without increased toxicity.

This study also will study biologic factors that may help to predict and treat prostate cancer. In addition, this study will gather information about the effects of radiation therapy on your sexual functioning and on your overall quality of life.

HOW MANY PEOPLE WILL TAKE PART IN THE STUDY

About 1520 people will take part in this study.

WHAT IS INVOLVED IN THE STUDY?

You will be "randomized" into one of the study groups described below. Randomization means that you are put into a group by chance. It is like flipping a coin. A computer will determine into which group you are placed. You will have approximately an equal chance of being placed in one of the following groups:

Treatment 1 (9/18/03)
If you are randomized to this treatment, you will receive the standard dose of 3D or IMRT radiation. You will receive radiation therapy once daily, five days a week, Monday through Friday, for a total of 39 treatments.

Treatment 2 (9/18/03)
If you are randomized to this treatment, you will receive a higher dose of 3D or IMRT radiation. You will receive radiation therapy once daily, five days a week, Monday through Friday, for a total of 44 treatments.

Each radiation treatment will take 30-60 minutes.

If you take part in this study, you also will have the following tests and procedures:

- A physical examination, including a digital rectal exam, prior to beginning treatment, every 3 months for the first two years following treatment, every 6 months until the fifth year, and then annually. The follow-up visits generally take 15 to 30 minutes.
- Blood tests prior to beginning treatment, weekly during radiation therapy, if your doctor feels these tests are needed, and at each follow-up visit as described above.
- CT scan or MRI (Magnetic Resonance Imaging) of the pelvis prior to treatment and an x-ray of the urethra (the canal from the bladder that discharges urine) prior to treatment, if your doctor feels this x-ray is indicated
- Bone scan, if indicated, prior to treatment
- Removal and biopsy of pelvic lymph glands, if indicated, to evaluate your cancer prior to treatment
- If your disease progresses, your physician may request a needle biopsy of your prostate to microscopically evaluate response to treatment.
- You will be asked to complete three brief questionnaires about your sexual and urinary functioning, bowel habits, and overall quality of life according to the following schedule (9/18/03):

Questionnaire	Before Treatment							
		3	6	9	12	15	18	24
Number 1- sexual	X		X		X			X
Number 2- urinary	X	X	X	X	X		X	X
Number 3- quality of life	X	X	X	X	X		X	X

After two years, you will be asked to complete questionnaire number 1 every year for 3 years and questionnaires numbers 2 and 3 every six months for 3 years. The questionnaires each take 10-15 minutes to complete.

HOW LONG WILL I BE IN THE STUDY?

If you participate in this study, you will receive radiation therapy for 8 to 9 weeks. Follow up will continue indefinitely, for as long as 13 years or longer.

The researcher may decide to take you off this study if your doctor decides it is in your best interest, if side effects become very severe or your condition worsens, or if new information becomes available that indicates it is in your best interest.

You can stop participating at any time. However, if you decide to stop participating in the study, we encourage you to talk to the researcher and your regular doctor first.

WHAT ARE THE RISKS OF THE STUDY?

While on the study, you are at risk for these side effects. You should discuss these with the researcher and/or your regular doctor. There also may be other side effects that we cannot predict. Other drugs will be given to make side effects less serious and uncomfortable, such as medication to reduce irritation of the bowel, rectum, or bladder. Inability to achieve an erection also can be successfully treated with medication in many circumstances. Many side effects go away shortly after the radiation therapy is stopped, but in some cases side effects can be serious or long-lasting or permanent. Some side effects do not become apparent for months or years after all treatment has been delivered.(9/18/03)

Risks and side effects related to the radiation therapy we are studying include:

Very Likely

Tanning or redness of skin in treatment area
Rash, itching or peeling of skin

Temporary hair loss in the treatment area
Temporary fatigue, nausea or diarrhea
Abdominal cramps
Bladder irritation with a stinging sensation
Frequency or urgency of urination
Rectal irritation with more frequent bowel movements

Less Likely, But Serious
Injury to the bladder, urethra, bowel, or other tissues in the pelvis or abdomen
Intestinal or urinary obstruction
Inability to achieve an erection
Rarely, rectal bleeding that requires medication or burning/cutting of tissues to stop

Risks and side effects related to the optional post-treatment needle biopsy include bleeding, pain, possible infection, and rarely, creation of an abnormal opening or passage.

Reproductive risks: Because the radiation in this study can affect an unborn baby, you should not father a baby while on this study. Ask about counseling and more information about preventing pregnancy.

ARE THERE BENEFITS TO TAKING PART IN THE STUDY?

If you agree to take part in this study, there may or may not be direct medical benefit to you. It is not known whether the higher dose of three-dimensional radiation therapy or IMRT you could receive will help your condition more than the standard dose. A possible benefit of this study may be a decrease in the size of your tumor and longer survival, but these benefits are not certain or guaranteed. We hope the information learned from this study will benefit other patients with prostate cancer in the future. (9/18/03)

WHAT OTHER OPTIONS ARE THERE?

You may choose to not participate in this study. Other treatments that could be considered for your condition may include the following: *(1)* external (non-3D) radiation therapy; *(2)* internal radiation *(seed implants or brachytherapy)*; *(3)* surgery; *(4)* hormone therapy; or *(5)* no treatment except medications to make you feel better. With the latter choice, your tumor could continue to grow and your disease could spread. These treatments could be given either alone or in combination with each other. If you decide not to participate in this study, you still

could receive 3D radiation therapy or IMRT similar to the therapy described above. (9/18/03)

Your doctor can tell you more about your condition and the possible benefits of the different available treatments.

Please talk to your regular doctor about these and other options.

WHAT ABOUT CONFIDENTIALITY?

Efforts will be made to keep your personal information confidential. We cannot guarantee absolute confidentiality. Records of your progress while on the study will be kept in a confidential form at this institution and in a computer file at the headquarters of the Radiation Therapy Oncology Group *(RTOG).* Your personal information may be disclosed if required by law.

Organizations that may inspect and/or copy your research records for quality assurance and data analysis include groups such as the Food and Drug Administration *(FDA),* the National Cancer Institute *(NCI)* or its authorized representatives, and other groups or organizations that have a role in this study.

WHAT ARE THE COSTS?

Radiation therapy is a standard treatment for your prostate cancer, and health insurers generally cover its costs. The higher dose administered on Arm 2 may result in higher costs. Therefore, taking part in this study may lead to added costs to you or your insurance company. Please ask about any expected added costs or insurance problems. (9/18/03)

In the case of injury or illness resulting from this study, emergency medical treatment is available but will be provided at the usual charge. No funds have been set aside to compensate you in the event of injury.

You or your insurance company will be charged for continuing medical care and/or hospitalization. Medicare should be considered a health insurance provider.

You will receive no payment for taking part in this study.

WHAT ARE MY RIGHTS AS A PARTICIPANT?

Taking part in this study is voluntary. You may choose not to take part or may leave the study at any time. Leaving the study will not result in any penalty or loss of benefits to which you are entitled.

We will tell you about new information that may affect your health, welfare, or willingness to stay in this study.

A Data Safety and Monitoring Board, an independent group of experts, will be reviewing the data from this research throughout the study. We will tell you about the new information from this or other studies that may affect your health, welfare, or willingness to stay in this study.

WHOM DO I CALL IF I HAVE QUESTIONS OR PROBLEMS?

(This section must be completed)

For information about your disease and research-related injury, you may contact:

____________________	____________________
Name	Telephone Number

For information about this study, you may contact:

____________________	____________________
Name	Telephone Number

For information about your rights as a research subject, you may contact:
(OHRP suggests that this person not be the investigator or anyone else directly involved with the research)

____________________	____________________
Name	Telephone Number

You also may call the Project Office of the NCI Central Institutional Review Board *(CIRB)* at 888-549-0715 *(from the continental U.S. only)* or 800-937-8281, ext. 4445 *(from sites outside the continental U.S.).*

WHERE CAN I GET MORE INFORMATION?

You may call the NCI's Cancer Information Service at
1–800–4–CANCER (1–800–422–6237) or **TTY: 1–800–332–8615**

Visit the NCI's Web sites for comprehensive clinical trials information **http://cancertrials.nci.nih.gov** or for accurate cancer information including PDQ **http://cancernet.nci.nih.gov.**

CancerFax: Includes NCI information about cancer treatment, screening, prevention, and supportive care. To obtain a contents list, dial 301-402-5874 or 800-624-2511 from a fax machine handset and follow the recorded instructions.

<u>SIGNATURE</u> (8/23/02)

I have read all the above, asked questions, and received answers concerning areas I did not understand. I have had the opportunity to take this consent form home for review or discussion.

I willingly give my consent to participate in this program. Upon signing this form I will receive a copy. I may also request a copy of the protocol *(full study plan)*.

______________________	______________________	___________
Patient's Name	Signature	Date
______________________	___________________	___________
Name of Person Obtaining Consent	Signature	Date

APPENDIX IB (8/23/02)

RTOG 0126 (10/18/04)

CONSENT FORM FOR USE OF TISSUE AND BLOOD FOR RESEARCH

ABOUT USING TISSUE AND BLOOD FOR RESEARCH (7/10/07)

You have had or will have a biopsy (or surgery) to see if you have cancer. Your doctor has removed or will remove some body tissue to do some tests. The results of these tests will be given to you by your doctor and will used to plan your care.

We would like to keep some of the tissue that is left over for future research. If you agree, this tissue will be kept and may be used in research to learn more about cancer and other diseases.

In addition, you will have blood tests before you start treatment. We would like to keep about four teaspoons of blood for future research as well. If you agree, this blood will be kept and may be used in research to learn more about cancer and other diseases. One specific test will analyze whether your blood contains certain genes and if the side effects you had on radiation are related to these genes. We will then try to see if these genes can help us learn about why some people get worse side effects than others.

Your tissue and blood may be helpful for research whether you do or do not have cancer. The research that may be done with your tissue and blood is not designed specifically to help you. It might help people who have cancer and other diseases in the future.

Reports about research done with your tissue and blood will not be given to you or your doctor. These reports will not be put in your health record. The research will not have an affect on your care.

THINGS TO THINK ABOUT (7/10/07)

The choice to let us keep the left over tissue and blood for future research is up to you. **No matter what you decide to do, it will not affect your care.**

If you decide now that your tissue and blood can be kept for research, you can change your mind at any time. Just contact us and let us know that you do not want us to use your tissue and blood and then any tissue and blood that remains will no longer be used for research; or, you may request that your tissue be returned to you or your designee.

In the future, people who do research may need to know more about your health. While the [treating institution/treating physician] may give them reports about

your health, it will not give them your name, address, phone number, or any other information that will let the researchers know who you are.

Sometimes tissue and blood are used for genetic research (about diseases that are passed on in families). Even if your tissue and blood is used for this kind of research, the results will not be put in your health records.

Your tissue and blood will be used only for research and will not be sold. The research done with your tissue and blood may help to develop new products in the future.

BENEFITS (7/10/07)

The benefits of research using tissue and blood include learning more about what causes cancer and other diseases, how to prevent them, and how to treat them.

RISKS

The greatest risk to you is the release of information from your health records. The [treating institution/treating physician] will protect your records so that your name, address, and phone number will be kept private. The chance that this information will be given to someone else is very small.

MAKING YOUR CHOICE (7/10/07)

Please read each sentence below and think about your choice. After reading each sentence, circle "Yes" or "No". **No matter what you decide to do, it will not affect your care.** If you have any questions, please talk to your doctor or nurse, or call our research review board at [IRB's phone number].

1. My tissue/blood may be used for the research in the current study.

   Yes No

2. My tissue/blood may be kept for use in research to learn about, prevent or treat cancer.

   Yes No

3. My tissue/blood may be kept for use in research to learn about, prevent or treat other health problems (for example: diabetes, Alzheimer's disease, or heart disease).

   Yes No

4. My blood may be kept for use in future research to learn about the correlation between genes and radiation side effects.

   Yes No

5. Someone from [treating institution/treating physician] may contact me in the future to ask me to take part in more research.

Yes No

Participant statement:
I have read and received a copy of this consent form. I have been given an opportunity discuss the information with my doctor/nurse, and all of my questions/concerns have been answered to my satisfaction. My answers above and my signature below indicate my voluntary participation in this research.

______________________________ ________________ ______________
Patient's Name Signature Date

Witness statement:
I have explained the information in this consent form to the patient and have answered any questions raised. I have witnessed the patient's signature.

________________________________ __________________ __________
Name of Person Obtaining Consent Signature Date

APPENDIX II

KARNOFSKY PERFORMANCE SCALE

100	Normal; no complaints; no evidence of disease
90	Able to carry on normal activity; minor signs or symptoms of disease
80	Normal activity with effort; some sign or symptoms of disease
70	Cares for self; unable to carry on normal activity or do active work
60	Requires occasional assistance, but is able to care for most personal needs
50	Requires considerable assistance and frequent medical care
40	Disabled; requires special care and assistance
30	Severely disabled; hospitalization is indicated, although death not imminent
20	Very sick; hospitalization necessary; active support treatment is necessary
10	Moribund; fatal processes progressing rapidly
0	Dead

ZUBROD PERFORMANCE SCALE

0 Fully active, able to carry on all predisease activities without restriction *(Karnofsky 90-100)*.

1 Restricted in physically strenuous activity but ambulatory and able to carry out work of a light or sedentary nature. For example, light housework, office work *(Karnofsky 70-80)*.

2 Ambulatory and capable of all self-care but unable to carry out any work activities. Up and about more than 50% of waking hours *(Karnofsky 50-60)*.

3 Capable of only limited self-care, confined to bed or chair 50% or more of waking hours *(Karnofsky 30-40)*.

4 Completely disabled. Cannot carry on any self-care. Totally confined to bed or chair *(Karnofsky 10-20)*.

APPENDIX III

AJCC STAGING SYSTEM
PROSTATE, 5th Edition

DEFINITION OF TNM

Primary Tumor, Clinical (T)

TX	Primary tumor cannot be assessed	
T0	No evidence of primary tumor	
T1	Clinically inapparent tumor not palpable or visible by imaging	
	T1a	Tumor incidental histologic finding in 5% or less of tissue resected
	T1b	Tumor incidental histologic finding in more than 5% of tissue resected
	T1c	Tumor identified by needle biopsy *(e.g., because of elevated PSA)*
T2	Tumor confined with prostate*	
	T2a	Tumor involves one lobe
	T2b	Tumor involves both lobes
T3	Tumor extends through prostate capsule**	
	T3a	Extracapsular extension *(unilateral or bilateral)*
	T3b	Tumor involves the seminal vesicle*(s)*
T4	Tumor is fixed or invades adjacent structures other than the seminal vesicles: bladder neck, external sphincter, rectum, levator muscles and/or pelvic wall	

*Note: Tumor found in one or both lobes by needle biopsy, but not palpable or reliably visible by imaging, is classified as T1c

**Note: Invasion into the prostatic apex or into *(but not beyond)* the prostatic capsule is not classified as T3, but as T2.

Regional Lymph Nodes (N)

NX	Regional lymph nodes cannot be assessed
N0	No regional lymph node metastasis
N1	Metastasis in regional lymph node or nodes

Primary Tumor, Pathologic (pT)

pT2***	Organ confined	
	pT2a	Unilateral
	pT2b	Bilateral
pT3	Extraprostatic extension	
	pT3a	Extraprostatic extension
	pT3b	Seminal vesicle invasion
pT4	Invasion of bladder, rectum	

***Note: There is no pathologic T1 classification

Distant Metastasis* (M)***

MX	Presence of distant metastasis cannot be assessed
M0	No distant metastasis

APPENDIX III ***(continued)***

AJCC STAGING SYSTEM
PROSTATE, 5th Edition

M1 Distant metastasis
- M1a Non regional lymph node*(s)*
- M1b Bone*(s)*
- M1c Other site*(s)*

****Note: When more than one site of metastasis is present, the most advanced category is used. pM1c is most advanced

Histopathologic Grade (G)

GX	Grade cannot be assessed
G1	Well-differentiated *(slight anaplasia)*
G2	Moderately differentiated *(moderate anaplasia)*
G3-4	Poorly undifferentiated or undifferentiated *(marked anaplasia)*

Stage Grouping

Stage I	T1a	N0	M0	G1
Stage II	T1a	N0	M0	G2, G3-4
	T1b	N0	M0	Any G
	T1c	N0	M0	Any G
	T1	N0	N0	Any G
	T2	N0	M0	Any G
Stage III	T3	N0	M0	Any G
Stage IV	T4	N0	M0	Any G
	Any T	N1,	M0	Any G
	Any T	Any N	M1	Any G

RTOG/EORTC Late Radiation Morbidity Scoring Scheme — APPENDIX IV

ORGAN TISSUE	0	*GRADE 1*	*GRADE 2*	*GRADE 3*	*GRADE 4*	5
**SKIN**	None	Slight atrophy; Pigmentation change; Some hair loss	Patch atrophy; Moderate telangiectasia; Total hair loss	Marked atrophy; Gross telangiectasia	Ulceration	**DEATH DIRECTLY RELATED TO RADIATION EFFECTS**
**SUBCUTANEOUS TISSUE**	None	Slight induration (fibrosis) and loss of subcutaneous fat	Moderate fibrosis but asymptomatic; Slight field contracture; <10% linear reduction	Severe induration and loss of subcutaneous tissue; Field contracture > 10% linear measurement	Necrosis	
**MUCOUS MEMBRANE**	None	Slight atrophy and dryness	Moderate atrophy and telangiectasia; Little mucous	Marked atrophy with complete dryness; Severe telangiectasia	Ulceration	
**SALIVARY GLANDS**	None	Slight dryness of mouth; Good response on stimulation	Moderate dryness of mouth; Poor response on stimulation	Complete dryness of mouth; No response on stimulation	Fibrosis	
**SPINAL CORD**	None	Mild L'Hermitte's syndrome	Severe L'Hermitte's syndrome	Objective neurological findings at or below cord level treated	Mono, para quadriplegia	
**BRAIN**	None	Mild headache; Slight lethargy	Moderate headache; Great lethargy	Severe headaches; Severe CNS dysfunction (partial loss of power or dyskinesia)	Seizures or paralysis; Coma	
**EYE**	None	Asymptomatic cataract; Minor corneal ulceration or keratitis	Symptomatic cataract; Moderate corneal ulceration; Minor retinopathy or glaucoma	Severe keratitis; Severe retinopathy or detachment Severe glaucoma	Panophthalmitis/Blindness	
**LARYNX**	None	Hoarseness; Slight arytenoid edema	Moderate arytenoid edema; Chondritis	Severe edema; Severe chondritis	Necrosis	
**LUNG**	None	Asymptomatic or mild symptoms (dry cough); Slight radiographic appearances	Moderate symptomatic fibrosis or pneumonitis (severe cough); Low grade fever; Patchy radiographic appearances	Severe symptomatic fibrosis or pneumonitis; Dense radiographic changes	Severe respiratory insufficiency/continuous O2/Assisted ventilation	
**HEART**	None	Asymptomatic or mild symptoms; Transient T wave inversion & ST Changes; Sinus tachycardia >110 (at rest)	Moderate angina on effort; Mild pericarditis; Normal heart size; Persistent abnormal T wave and ST changes ; Low ORS	Severe angina; Pericardial effusion; Constrictive pericarditis; Moderate heart failure; Cardiac enlargement; EKG abnormalities	Tamponade/Severe heart failure/Severe constrictive pericarditis	
**ESOPHAGUS**	None	Mild fibrosis; Slight difficulty in swallowing solids; No pain on swallowing	Unable to take solid food normally; Swallowing semi-solid food; Dilation may be indicated	Severe fibrosis; Able to swallow only liquids; May have pain on swallowing Dilation required	Necrosis/Perforation Fistula	
**SMALL/LARGE INTESTINE**	None	Mild diarrhea; Mild cramping; Bowel movement 5 times daily Slight rectal discharge or bleeding	Moderate diarrhea and colic; Bowel movement >5 times daily; Excessive rectal mucus or intermittent bleeding	Obstruction or bleeding, requiring surgery	Necrosis/Perforation Fistula	
**LIVER**	None	Mild lassitude; Nausea, dyspepsia; Slightly abnormal liver function	Moderate symptoms; Some abnormal liver; function tests; Serum albumin normal	Disabling hepatitic insufficiency; Liver function tests grossly abnormal; Low albumin; Edema or ascites	Necrosis/Hepatic coma or encephalopathy	
**KIDNEY**	None	Transient albuminuria; No hypertension; Mild impairment of renal function; Urea 25-35 mg%; Creatinine 1.5-2.0 mg%; Creatinine clearance > 75%	Persistent moderate albuminuria (2+); Mild hypertension; No related anemia; Moderate impairment of renal function; Urea > 36-60mg% Creatinine clearance (50-74%)	Severe albuminuria; Severe hypertension Persistent anemia (< 10%); Severe renal failure; Urea >60 mg% Creatinine >4.0 mg% Creatinine clearance < 50%	Malignant hypotension; Uremic coma/Urea > 100%	
**BLADDER**	None	Slight epithelial atrophy; Minor telangiectasia (microscopic hematuria)	Moderate frequency; Generalized telangiectasia; Intermittent macroscopic hematuria	Severe frequency & dysuria Severe generalized Telangiectasia (often with petechiae); Frequent hematuria; Reduction in bladder capacity (< 150 cc)	Necrosis/Contracted bladder (capacity < 100 cc); Severe hemorrhagic cystitis	
**BONE**	None	Asymptomatic; No growth retardation; Reduced bone Density	Moderate pain or tenderness; Growth retardation; Irregular bone sclerosis	Severe pain or tenderness; Complete arrest of bone growth; Dense bone sclerosis	Necrosis/Spontaneous fracture	
**JOINT**	None	Mild joint stiffness; Slight limitation of movement	Moderate stiffness; Intermittent or moderate joint pain; Moderate limitation of movement	Severe joint stiffness; Pain with severe limitation of movement	Necrosis/Complete fixation	

APPENDIX V (3/29/10) (5/1/14)

ADVERSE EVENT REPORTING GUIDELINES

Federal Regulations require that investigators report adverse events and reactions in a timely manner. This reporting improves patient care and scientific communication by providing information to the National Cancer Institute (NCI) whereby new findings can be more widely disseminated to investigators and scientists.

A. Definitions and Terminology

An adverse event is defined as an undesirable, unfavorable or unintended sign (including an abnormal laboratory finding), symptom or disease associated with the use of a medical treatment or procedure regardless of whether it is considered related to the medical treatment or procedure. This may be a new event that was not pre-existing at initiation of treatment, a pre-existing event that recurs with increased intensity or frequency subsequent to commencement of treatment or an event, though present at the commencement of treatment, becomes more severe following initiation of treatment. These undesirable effects may be classified as "known or expected" or "unknown or unexpected".

Known/expected events are those that have been previously identified as having resulted from administration of the agent or treatment. They may be identified in the literature, the protocol, the consent form, or noted in the drug insert.

Unknown/unexpected events are those thought to have resulted from the agent, e.g. temporal relationship but not previously identified as a known effect.

Assessment of Attribution

In evaluating whether an adverse event is related to a procedure or treatment, the following attribution categories are utilized:

**Definite:**	The adverse event is *clearly related* to the treatment/procedure.
**Probable:**	The adverse event is *likely related* to the treatment/procedure.
**Possible:**	The adverse event *may be related* to the treatment/procedure.
**Unlikely:**	The adverse event is *doubtfully related* to the treatment/procedure.
**Unrelated:**	The adverse event is *clearly NOT related* to the treatment/procedure.

B. Grading of Adverse Events (3/29/10)

Unless specified otherwise, the NCI Common Terminology Criteria for Adverse Events (CTCAE) version 4.0 will be used to grade severity of adverse events, beginning April 1, 2010. The CTCAE version 4.0 is located on the CTEP website at (http://ctep.cancer.gov/protocolDevelopment/electronic_applications/ctc.htm). All appropriate treatment areas should have access to a copy of the CTCAE version 4.0.

C. General Guidelines

In order to assure prompt and complete reporting of adverse events and toxicity, the following general guidelines must be observed. The guidelines apply to all RTOG studies. **When protocol-specific guidelines indicate more intense monitoring than the standard guidelines, the study-specific reporting procedures supercede the General Guidelines.** A protocol may stipulate that specific grade 4 events attributable to treatment are expected and therefore may not require the standard reporting; however, exceptions to standard reporting must be specified in the text of the protocol.

1. The Principal Investigator will report to the RTOG Group Chair, to the Headquarters Data Management Staff (215/574-3214) and to the Study Chair within 24 hours of discovery, the details of all unexpected severe, life-threatening (grade 4) and fatal (grade 5) adverse events if there is reasonable suspicion that the event was definitely, probably, or possibly related to protocol treatment.

2. All deaths during protocol treatment or within 30 days of completion or termination of protocol treatment regardless of attribution require telephone notification within 24 hours of discovery.

3. A written report, including all relevant clinical information and all study forms due up to and including the date of the event, will be sent by mail or FAX (215/928-0153) to RTOG Headquarters within 10 working days of the telephone report (unless specified otherwise within the protocol). The material must be labeled: ATTENTION: Adverse Event Reporting.

a. The Group Chair in consultation with the Study Chair will take appropriate and prompt action to inform the membership and statistical personnel of any protocol modifications and/or precautionary measures, if this is warranted.

b. For events that require telephone reporting to the National Cancer Institute (NCI), Investigational Drug Branch (IDB), the Food and Drug Administration (FDA), to another co-operative group or to the study sponsor, the investigator may first call RTOG (as outlined above) unless this will unduly delay the required notification process.

A copy of all correspondence sent to recipients of the call, e.g. NCI, IDB, another cooperative group office (non-RTOG coordinated studies) must be submitted to RTOG Headquarters. **Copies must include the RTOG study and case numbers.**

4. When participating in non-RTOG coordinated intergroup studies or in RTOG sponsored pharmaceutical studies, the investigator must comply with the reporting specification required in the protocol.

5. Institutions must comply with their individual Institutional Review Board policy regarding submission of documentation of adverse events. All "expedited" adverse event reports should be sent to the local Institutional Review Board (IRB).

6. Failure to comply with reporting requirements in a timely manner may result in suspension of patient registration.

7. When submitting reports and supporting documentation for reports to RTOG on an RTOG protocol patient, **the study number and the case number must be recorded** so that the case may be associated with the appropriate study file. This includes submission of copies of FDA Form 3500 (MedWatch).

8. All data collection forms through the date of the reported event and the applicable reporting form are submitted to RTOG Headquarters data management department (Attention: Adverse Event) **within 10 working days** of the telephone report or sooner if specified by the protocol. Documentation must include an assessment of attribution by the investigator as previously described in section A.

9. MedWatch Forms (FDA 3500) submitted on RTOG protocol patients must be signed by the Principal Investigator.

10. All neuro-toxicity ($\geq$ grade 3) from radiosensitizer or radioprotector drugs are to be reported to RTOG Headquarters Data Management, to the Group Chair, and to the Study Chair within 10 days of discovery.

D. Adverse Event Reporting Related to Radiation Therapy (3/29/10)

1. All fatal events resulting from protocol radiation therapy must be reported by telephone to the Group Chair, to RTOG Headquarters Data Management department and to the radiation therapy protocol Study Chair within 24 hours of discovery.

2. All grade 4, (NCI Common Terminology Criteria for Adverse Events [CTCAE] version 4.0—beginning April 1, 2010—and RTOG/EORTC Late Radiation Morbidity Scoring Scheme Criteria) and life-threatening events (an event, which in view of the investigator, places the patient at immediate risk of death from the reaction) and grade 4 toxicity that is related, possibly related or probably related to protocol treatment using non-standard fractionated radiation therapy, brachytherapy, radiopharmaceuticals, high LET radiation, and radiosurgery must be reported by telephone to the Group Chair, to RTOG Headquarters Data Management and to the radiation therapy Study Chair within 24 hours of discovery. Expected grade 4 adverse events may be excluded from telephone reporting if specifically stated in the protocol.

3. All applicable data forms and if requested, a written report, must be submitted to RTOG Headquarters within 10 working days of the telephone call.

E. Adverse Event Reporting Related to Systemic Anticancer Agents (3/29/10) (5/1/14)

Adverse drug reactions (ADRs) are adverse events that are related to an anticancer agent and meet certain criteria: are unexpected effects of the drug or agent, or are severe (grade 3), life-threatening (grade 4), or fatal (grade 5), even if the type of event has been previously noted to have occurred with the agent.

1. Commercial Agents/Non-Investigational Agents

	Grade 4 or 5 Unexpected with Attribution of Possible, Probable, or Definite	Increased Incidence of an Expected AE[1]	Hospitalization During Treatment[2]	Secondary AML/MDS[3]
FDA Form 3500[4,5] within 10 days	X	X	X	
NCI/CTEP Secondary AML/MDS Form within 10 days of diagnosis [4,5]				X
Call RTOG within 24 hrs of event[7]	X[6]			

1. Any increased incidence of a known AE.
2. Inpatient hospitalizations or prolongation of existing hospitalization for medical events equivalent to the NCI Common Terminology Criteria for Adverse Events (CTCAE) version 4.0 (beginning April 1, 2010) Grade 3-5 which precipitated hospitalization must be reported regardless of the requirements or phase of study, expected or unexpected and attribution.
3. Reporting required during or subsequent to protocol treatment.
4. Submitted to Investigational Drug Branch, PO Box 30012, Bethesda, MD 20924-0012.
5. Copy to RTOG Data Management labeled: Attention: Adverse Event Report.
6. All grade 5 known toxicity.
7. Call RTOG Data Management (215) 574-3214. To leave a voice mail message when the office is closed, announce that you're reporting an "adverse event", provide your name, institution number, and a telephone number where you may be contacted.

2. Investigational Agents

An investigational agent is one sponsored under an Investigational New Drug Application (IND). Reporting requirements and timing are dependent on the phase of the trial, grade, attribution and whether the event is expected or unexpected as determined by the NCI Agent Specific Expected Adverse Event List, protocol and/or Investigator's Brochure. An expedited adverse event report requires submission to CTEP via CTEP-AERS (CTEP Adverse Event Reporting System). See the CTEP Home Page, http://ctep.cancer.gov for complete details and copies of the report forms.

a. CTEP-AERS (CTEP Adverse Event Reporting System)

The CTEP Adverse Event Reporting System (CTEP-AERS) was implemented for all protocols for which NCI is the supplier of an investigational agent.

Attribution: An expedited report is required for all unexpected and expected Grade 4 and Grade 5 adverse events regardless of attribution for any phase of trial. An expedited report is required for unexpected Grade 2 and Grade 3 adverse events with an attribution of possible, probable or definite for any phase of trial. An expedited report is not required for unexpected or expected Grade 1 adverse events for any phase of the trial.

RTOG uses "decentralized" notification. This means that all reportable events will be directly reported to NCI, just as has been done with paper-based reporting. CTEP-AERS is an electronic reporting system; therefore, all events that meet the criteria must be reported through the CTEP-AERS web application. Once the report is filed with CTEP-AERS, the institution need not send notification to RTOG, as the CTEP-AERS system will notify the Group Office. Institutions that utilize this application are able to print the report for local distribution, i.e., IRB, etc.

For institutions without Internet access, if RTOG is the coordinating group for the study, contact RTOG Data Management (215-574-3214) to arrange for CTEP-AERS reporting. In these instances, the appropriate Adverse Event Expedited Report template (Single or Multiple Agents) must be completed. The template must be fully completed and in compliance with the instruction manual; i.e., all mandatory sections must be completed including coding of relevant list of value (LOV) fields before sending to RTOG. Incomplete or improperly completed templates will be returned to the investigator. This will delay submission and will reflect on the timeliness of the investigators' reporting. A copy of the form sent to RTOG must be kept at the site if local distribution is required. Do not send the template without first calling the number noted above.

Templates for Single or Multiple Agents may be printed from the CTEP web page or will be supplied from the RTOG Registrar upon faxed request (FAX) (215) 574-0300.

When reporting an event on a patient in an RTOG-coordinated study, you must record the RTOG case number in the Patient ID field.

CTEP-AERS reporting does not replace or obviate any of the required telephone reporting procedures.
Investigational Agent(s) used in a Clinical Trial Involving a Commercial Agent(s) on separate arms: **An expedited adverse event report should be submitted for an investigational agent(s) used in a clinical trial involving a commercial agent(s) on a separate arm only if the event is specifically associated with the investigational agent(s).**
Investigational Agent(s) used in a Clinical Trial in Combination with a Commercial Agent(s): **When an investigational agent(s) supplied under an NCI-sponsored IND is used in combination with a commercial agent(s), the combination should be considered investigational and reporting should follow the guidelines for investigational agents.**

b. Expedited Reporting for Phase 1 Studies

**Unexpected Event**		**Expected Event**	
**Grades 2-3 Attribution: Possible, Probable or Definite**	**Grades 4 & 5 Regardless of Attribution**	**Grades 1 - 3**	**Grades 4 & 5 Regardless of Attribution**
Grade 2: Expedited report within 10 working days.<br><br>Grade 3: Report by phone to IDB[1,2] within 24 hrs. Expedited report to follow within 10 working days.<br><br>Grade 1: Adverse Event Expedited Reporting **NOT** required.	Report by phone to IDB[1,2] within 24 hrs. Expedited report to follow within 10 working days.<br><br>This includes deaths within 30 days of last dose of treatment with an investigational agent.	Adverse Event Expedited Reporting **NOT** required.	Report by phone to IDB[1,2] within 24 hrs.<br><br>Expedited report to follow within 10 working days.<br><br>This includes deaths within 30 days of the last dose of treatment with an investigational agent.

1. Report by telephone to RTOG Data Management (215) 574-3214, to the Group Chair and to the Study Chair. To leave a voice mail message with RTOG when the office is closed, announce that you're reporting an "adverse event", provide your name, institution number and a telephone number where you may be contacted.
2. Telephone reports to IDB (301) 230-2330 available 24 hours a day (recorder after 5 PM to 9 AM ET).

c. Expedited Reporting for Phase 2 and Phase 3 Studies

Unexpected Event		Expected Event	
**Grades 2-3 Attribution: Possible, Probable or Definite**	**Grades 4 & 5 Regardless of Attribution**	**Grades 1 - 3**	**Grades 4 & 5 Regardless of Attribution**
Expedited report within 10 working days.<br><br>Grade 1: Adverse Event Expedited Reporting **NOT** required.	Report by phone to IDB[1,2] within 24 hrs. Expedited report to follow within 10 working days.	Adverse Event Expedited Reporting **NOT** required.	Expedited including Grade 5 aplasia in leukemia patients within 10 working days. Grade 4 myelosuppression not to be reported, but should be submitted as part of study results. Other Grade 4 events that do not require expedited reporting would be specified in the protocol.

1. Report by telephone to RTOG Data Management (215) 574-3214, to the Group Chair and to the Study Chair. To leave a voice mail message with RTOG when the office is closed, announce that you're reporting an "adverse event", provide your name, institution number and a telephone number where you may be contacted.

2. Telephone reports to IDB (301) 230-2330 available 24 hours a day (recorder after 5 PM to 9 AM ET).

Appendix VIA (9/18/03) (10/18/04)
(Posted on Image-Guided Therapy Center [ITC] Web Site)
3DCRT Quality Assurance Guidelines
for RTOG 0126 (10/18/04)

Current Edition: 19 June 2003

I. Purpose

To establish credentialing requirements and quality assurance (QA) guidelines for institutions planning to participate in ATC supported protocols allowing 3-D conformal radiation therapy (3DCRT).

II. A partial list of references that provide background information regarding 3DCRT are listed below.

1. Report of the Collaborative Working Group on the Evaluation of Treatment Planning for External Photon Beam Radiotherapy, A.L. Smith and J.A. Purdy - guest editors, Int. J. Radiat. Oncl. Biol. Phys. 21(l), 1991.
2. ICRU Report 50: Prescribing, Recording, and Reporting Photon Beam Therapy. Bethesda, MD: International Commission on Radiation Units and Measurements, 1993.
3. ICRU Report 62: Prescribing, Recording, and Reporting Photon Beam Therapy (Supplement to ICRU Report 50). Bethesda, MD: International Commission on Radiation Units and Measurements, 1999.
4. Implementation of Three Dimensional Conformal Radiotherapy, A.L. Smith and C. Clifton Ling - guest editors, Int. J. Radiat. Oncol. Biol. Phys. 33(5), 1995.
5. A Practical Guide to 3-D Planning and Conformal Radiation Therapy. Edited by J.A Purdy and George Starkschall, Advanced Medical Publishing, Madison, WI. 369 pp. May, 1999.
6. 3-D Conformal and Intensity Modulated Radiation Therapy. Edited by J.A. Purdy, W.H. Grant III, J.R. Palta, E.B. Butler, and C.A. Perez, Advanced Medical Physics Publishing, Madison, WI. (2001)

III. Credentialing Requirements for Participating Institutions

A. The following items are required before you can enter cases on each ATC supported protocols allowing 3DCRT:

1. Submit a completed 3DCRT Facility Questionnaire specific to the 3DCRT protocol

   Image-guided Therapy Center
   4511 Forest Park Ave., Suite 200
   St. Louis, MO 63108
   E-mail: itc@castor.wustl.edu
   Phone: 314-747-5414
   FAX: 314-747-5423

2. Contact the ITC (itc@castor.wustl.edu) and request an FTP account for digital data submission (unless your institution already has been issued a FTP account for a different protocol).
3. Submit and successfully complete a protocol specific Dry-Run test

B. Facility Questionnaire: A current copy of the Facility Questionnaire may only be obtained via the world-wide web at *http://itc.wustl.edu*. The Facility Questionnaire provides information regarding the training and experience of the 3DCRT team; 3DCRT treatment planning and treatment equipment; and in-house QA procedures.

1. Computer planning system: Documentation of 3DCRT system to be used. To participate in ATC supported protocols allowing3DCRT, the institution's planning system must have the capability of digital data exchange with the ATC for all digital data required by the specific protocol. This digital data must comply with one of two possible formats:
   - RTOG Specification for Tape/Network Format for Exchange of Treatment planning Data, Version 3.20, or later; or
   - DICOM 3.0 in compliance with the ATC's DICOM 3.0 Conformance Statement.
2. Treatment Verification Procedures: Documentation of the 3DCRT planning and delivery process as well as the routine QA tests performed to insure proper functioning. The method used to conduct a check of the dose and

monitor unit calculations performed by the 3DCRT planning system must be provided.

C. Dry Run (Benchmark) Test: A complete patient data set as specified by the treatment protocol is to be submitted to the ATC to demonstrate compliance with 3D technical requirements (see Dry Run Guidelines at http://itc.wustl.edu/).

1. No port films are required for the Dry Run test other than DRRs, as the patient's treatment is not required to be per protocol. However, if you plan on submitting your treatment verification images in digital format, you must prove that you have a compliant method of submitting these images as part of the Dry Run test.
2. **NOTE:** There is no requirement that the patient whose data is used for the Dry Run test be treated according to the protocol. This test set can be from a data set for a patient who was previously seen and/or treated (in some other fashion). The only requirement is that the CT scan be close to protocol compliant and the tumor/target volumes and critical normal structure contours be defined in compliance with the protocol and that protocol compliant treatment plans be generated and the appropriate data submitted to the ATC. Any protocol immobilization device requirement is waived for this test data set. All patient identifying data for the Dry Run test data must be removed before submission to protect patient confidentiality.

IV. Protocol Requirements

A. Protocols permitting 3DCRT treatment delivery must be written using the nomenclature defined in the International Commission on Radiation Units and Measurements (ICRU) Reports 50 and 62 for specifying the volumes of known tumor, i.e., Gross Tumor Volume (GTV), the volumes of suspected microscopic spread, i.e., Clinical Target Volume (CTV), and the marginal volumes necessary to account for setup variations and organ and patient motion, i.e., Planning Target Volume (PTV). Report 62 introduced the concept of the Planning Organ at Risk Volume (PRV), in which a margin is added around the critical structure to compensate for that organ's geometric uncertainties. The PRV margin around the critical structure is analogous to the PTV margin around the CTV.

B. The protocol must provide a clear definition of the GTV, CTV, and the PTV.

C. The protocol must provide a clear definition of the prescription dose and dose heterogeneity allowed throughout the PTV.

D. The protocol must require that a volumetric treatment planning CT study be used to define the GTV.

E. The protocol must clearly define the organs-at-risk that are required to be contoured and provide clear guidelines for contouring each organ-at-risk defined in the study. Dose constraints for each organ-at-risk in the irradiated volume must be defined. This should include a reasonable definition of major and minor deviation for each item of interest.

F. The protocol must require that specific procedures be in place to insure correct, reproducible positioning of the patient. As a minimum, orthogonal (AP and lateral) DRRs and corresponding orthogonal portal images (film or electronic) are to be required.

G. The treatment machine monitor units generated using the 3DCRT planning system must be independently checked prior to the patient's first treatment.

V. Protocol Data and Quality Assessment Parameters

A. Patient Data Submission: The following information is to be submitted to the ATC for each protocol patient at times specified in the protocol:

1. T2 Form: Digital Patient Information Submission Form (obtain from ATC website).

2. 3DCRT digital dosimetry and imaging data.
   a. Protocol compliant images (e.g. CT or MRI scan series);

   b. Protocol compliant contours using required standard names (standard structure names can be found on the ATC website) for all GTV, CTV and PTVs, and for all specified critical normal structures. They must be contoured on all slices in which each structure exists or as defined by the protocol and includes skin on ALL

CT cuts;

c. Beam geometry specifications including ICRU 50 reference point doses (for the purposes of this protocol, the isocenter dose should suffice) in absolute dose units (all fraction groups required with initial submission).

d. Volumetric 3-D dose distribution (see protocol for requirements regarding no heterogeneity and/or heterogeneity corrections) data in absolute dose for each fraction group used to deliver a protocol compliant dose. Note, a Fraction Group represents the beams and doses for a concurrently treated set of beams;

e. DVH's computed for the total dose of all dose distributions submitted for item d (summed fraction groups from item d) for all PTVs and all critical normal structures (excluding Unspecified Tissue);

f. Any corrections to previously submitted digital data should be discussed with the ATC prior to such submission.

3. Color hardcopy isodose distribution for the axial, sagittal and coronal planes through the isocenter for the total dose plan must be submitted. If sagittal and coronal hard copy can not be generated, five axial distributions may be substituted for them (two cuts which are 2 slices superior and inferior of the superior and inferior slices containing the high dose PTV, the superior and inferior cuts containing the high dose PTV, and one through the center of the boost PTV. These dose distributions must include:

   a. A reasonable number of isodose lines should be shown which can be used to determine that the digital dose and anatomy data are properly aligned relative to each other. The prescription dose for the high-dose PTV should be displayed. If the hard copy isodose lines are in percentage, the conversion factor to convert them to absolute dose (Gy or cGy) for all delivered fractions must be indicated.

   b. The above isodoses shall be superimposed over the treatment planning CT images or reconstructed planes of the planning CT images and should be in color.

4. Treatment prescription and verification images:

   a. The ATC will archive in an ATC database all digital treatment prescription and verification images (hard copy films are to be digitized) for later review by the study chair of initial placement films submitted by each institution. At least one port film or pretreatment alignment film per field along with the DRR from the treatment planning program or, alternatively, a simulation verification radiograph shall be submitted for evaluation except where geometrically impractical.

VI. QA Review

A. Quality Assurance of the CT Scan Data and Digital Planning Data Format

1. The CT scan data set will be reviewed to ensure protocol compliance with regard to both inter-slice spacing as well as the superior/inferior extents of the scan region.
2. The format of the digital treatment planning data submitted will be reviewed for compliance with the appropriate data exchange specification version. Deviations from compliance will be noted and, depending upon the severity of the deviation, may require a complete resubmission of the digital data set.

B. Quality Assurance of Target Volumes and Organs at Risk Volumes

1. The ATC will facilitate the review of GTV, CTV, and PTV on, at a minimum, the first 5 cases submitted by each institution. After the institution has demonstrated compliance with the protocol, future cases may be spot checked only.

2. The ATC will facilitate the review of all designated critical structures on, at a minimum, the first 5 cases submitted by each institution. After the institution has demonstrated compliance with protocol, future cases may be spot checked only.

C. Quality Assurance of Dose Distribution

1. The ATC will display, and compare with hardcopies, isodose distributions for the planes submitted to verify correct interpretation and conversion of the digital patient and dose data.
2. The ATC will calculate DVH's for the sum of all dose distributions submitted (each submitted distribution is for one set of concurrently treated beams) and may compare them with the digitally submitted dose-volume histograms for the PTV, designated critical structures, and unspecified tissue.
   a. There should be reasonable agreement between an individual participating institution's DVH computations and those of the ATC. Therefore, any discrepancy between the submitting institution's DVHs and those computed by the ATC in excess of $\pm$5% (or 3 cc for small structures) in total volume or $\pm$5% (relative to the absolute structure volume) of the volume calculated to be at or above the appropriate TD 5/5 dose for the particular structure will need to be resolved prior to successfully completing the Dry Run Test.

D. **Dose QA Score Assignment:** Each protocol must have established criteria for evaluating the submitted treatment plan. The criteria will be published on the ATC website. An overall score will be assigned to each plan. The items involved in the scoring are the coverage and overdose of each PTV and the level of specified organ(s)-at-risk sparing. ***The largest variation encountered (None, Minor or Major) will be the overall score assigned to the plan.*** No credentialing plan (dry run) will be approved that results in a Major Variation. Plans with No Variation or Minor Variations will be approved (assuming no other significant areas of protocol non-compliance).

1. **Compliance with Prescription Dose Coverage:** A (1) No variation, (2) Minor variation (marginal coverage), and (3) Major variation (miss) criteria similar to that posted for RTOG 0126 will be used to evaluate plan compliance. (10/18/04)

2. **Compliance with Dose Heterogeneity**: A (1) No variation, (2) Minor variation, and (3) Major variation criteria similar to that posted for RTOG 0126 will be used to evaluate plan compliance. (10/18/04)

   The maximum point dose (to a volume of specified cc's) to organs at risk (and unspecified tissue) outside the $PTV_{High\ Dose}$ volume should not exceed the protocol specified allowable dose. The treating physician must carefully consider the tolerance dose/volume to each organ at risk and unspecified tissue.

Last modified: 06/19/2003 17:30:44

Appendix VIB (9/18/03)

3DCRT Facility Questionnaire

Please type this form.

The following items are required before you can enter cases on each RTOG 3DCRT protocol supported by the Image-Guided Therapy QA Center (ITC):

Submit this completed Facility Questionnaire for the 3DCRT protocol to the:

Image-guided Therapy Center
4511 Forest Park Ave., Suite 200
St. Louis, MO 63108

E-mail: itc@castor.wustl.edu
Phone: 314-747-5414
FAX: 314-747-5423

1. Contact the ITC (itc@castor.wustl.edu) and request an FTP account for digital data submission

2. Submit and successfully complete a protocol specific Dry-Run test

RTOG Protocol #:______________________________**RTOG Institution #:**__________________________

Institution Name:____

If Affiliate, Name of Member Institution:____

Date Questionnaire Submitted:_____/_____/_______

Physicist:__________________________________e-mail:____________________________________
Address:____

____

____

____

Telephone:__________________________ Fax:_________________________
Research Associate:__________________________e-mail:____________________________________
Telephone:__________________________
Fax:_________________________
Dosimetrist:_________________________________e-mail:____________________________________
Telephone:__________________________
Fax:_________________________
Responsible Radiation Oncologist(s) ____
Telephone:__________________________ e-mail:___________________________________

EXPERIENCE OF PERSONNEL

A. For the Radiation Oncologist named above, how many total 3DCRT treatment have been planned and delivered?

Approximately how many 3DCRT treatments have been planned and delivered in the past 6 months?

In the past 12 months?

Have this physician's skills previously been verified through either the ITC or QARC?

If yes, indicate QA Review Center and date of completion:

☐ QARC Date:	☐ ITC Date:

B. For the Physicist named above, how many total 3DCRT treatment have been planned and delivered? ☐

Approximately how many 3DCRT treatments have been planned and delivered in the past 6 months? ☐

In the past 12 months? ☐

Have this physicist's skills previously been verified through either the ITC or QARC? ☐

If yes, indicate Review Center and date of completion:

☐ QARC Date:	☐ ITC Date:

<u>EQUIPMENT TO BE USED FOR 3DCRT</u>

A. Treatment Unit

Manufacturer And Machine Model	Nominal Energy (MV)	Nominal SSD/SAD (cm)	***Field Shaping*** **(check all that apply)**
			☐ MLC<br>☐ Custom Blocks
			☐ MLC<br>☐ Custom Blocks
			☐ MLC<br>☐ Custom Blocks
			☐ MLC<br>☐ Custom Blocks
			☐ MLC<br>☐ Custom Blocks
			☐ MLC<br>☐ Custom Blocks

B.CT Simulator (CT Scanner)

**<u>Manufacturer</u>**	**<u>Model</u>**
______________________	______________________

C. For prostate 3DCRT protocols only: How are prostate patients immobilized for IMRT (mark all that apply)

☐ Knee sponge only

☐ Thermoplastic cast

☐ Knee sponge and foot holder

☐ Foam-immobilization mold

☐ Other: ______________________

The RTOG 0126 protocol as written does require… "the patient to be positioned in the supine position in an individualized thermoplastic cast or molded foam cradle in the treatment position on a flat tabletop." If the institution wishes to use a different type of immobilization system or "no immobilization", they must perform an internal study documenting that the combined range of setup error and internal organ motion is within the 5-10 mm range, and submit the report for Study Chair review and approval. (10/18/04)

D. 3D Treatment Planning System(s)

	**Manufacturer**	**Version**
1.	______________________	______________________
2.	______________________	______________________
3.	______________________	______________________

E. For prostate 3DCRT protocols only: Describe the margins you typically use for 3DCRT of prostate cancer.

Target	Anterior (cm)	Posterior (cm)	Superior (cm)	Inferior (cm)	Left (cm)	Right (cm)
Seminal Vesicles						
Prostate						

TREATMENT VERIFICATION USED FOR 3DCRT

A. How do you verify field positioning relative to the patient's anatomy (check all that apply)?

☐ port film ☐ orthogonal port films ☐ BAT ultrasound

☐ Other: ______________________

B. How often is positioning verification done?

☐ first treatment only ☐ daily ☐ weekly

☐ Other: ______________________

C. Describe the method used to conduct a check of the dose and monitor unit calculations generated by the 3DRTP system.

____

____

____

____

D. Are your 3DCRT treatments monitored by a record and verify system?

Manufacturer & Model:

E. Treatment Machine Calibration and Reviews

1. How is the calibration of this machine traced to NIST?

____

____

2. When was your last RPC site visit (m/d/yy)?

3. When was your last set of RPC TLDs performed (m/d/yy)?

Appendix VIIA (9/18/03) (10/18/04)
(Posted on Image-Guided Therapy Center [ITC] Web Site)
IMRT Quality Assurance Guidelines
for RTOG 0126 (10/18/04)

Current Edition: 19 June 2003

I. Purpose

To establish credentialing requirements and quality assurance (QA) guidelines for institutions planning to participate in ATC supported protocols allowing intensity modulated radiation therapy (IMRT).

II. A partial list of references that provide background information regarding IMRT are listed below.

1. ICRU Report 50: Prescribing, Recording, and Reporting Photon Beam Therapy. Bethesda, MD: International Commission on Radiation Units and Measurements, 1993.
2. ICRU Report 62: Prescribing, Recording, and Reporting Photon Beam Therapy (Supplement to ICRU Report 50). Bethesda, MD: International Commission on Radiation Units and Measurements, 1999.
3. Radiation Therapy Treatment Optimization, Seminars in Radiation Oncology Vol. 9(l): T. Rockwell Mackie - guest editor, W.B. Saunders Company, Philadelphia, PA, 1999.
4. IMRT Collaborative Working Group: Intensity modulated radiation therapy: current status and issues of interest. Int. J. Radiat. Oncol. Biol. Phys. 51:880-914, 2001
5. 3-D Conformal and Intensity Modulated Radiation Therapy. Edited by J.A. Purdy, W.H. Grant III, J.R. Palta, E.B. Butler, and C.A. Perez, Advanced Medical Physics Publishing, Madison, WI. (2001)
6. Intensity Modulated Radiation Therapy for Head and Neck Cancer. Edited by KSC_Chao and G Ozyigit, Lippincott Williams & Wilkins, Philadelphia, PA. (2003).

III. Credentialing Requirements for Participating Institutions

A. The following items are required before you can enter cases on each ATC supported protocols allowing IMRT:

1. Submit a completed IMRT Facility Questionnaire specific to the IMRT protocol

Image-guided Therapy Center
4511 Forest Park Ave., Suite 200
St. Louis, MO 63108

E-mail: itc@castor.wustl.edu
Phone: 314-747-5414
FAX: 314-747-5423

2. Contact the ITC (itc@castor.wustl.edu) and request an FTP account for digital data submission (unless your institution already has been issued a FTP account for a different protocol).

3. Submit and successfully complete a protocol specific Dry-Run test

4. A successful IMRT phantom experiment must be completed and the appropriate documents sent to the RPC and the digital data with the standard set of hard copy isodoses sent to the ITC. Contact Michael Gillin, Ph.D. at the M.D. Anderson Cancer Center or the Radiological Physics Center (713-745-8989) to arrange for the phantom to be sent to you.

Facility Questionnaire: A current copy of the Facility Questionnaire may only be obtained via the world-wide web at *http://itc.wustl.edu*. The Facility Questionnaire provides information regarding the training and experience of the IMRT team; IMRT treatment planning and treatment equipment; and in-house QA procedures.

1. IMRT Computer planning system: Documentation of IMRT system to be used. To participate in ATC supported protocols allowing IMRT, the institution's planning system must have the capability of digital data exchange with the ATC for all digital data required by the specific protocol. This digital data must comply with one of two possible formats:
   - RTOG Specification for Tape/Network Format for Exchange of Treatment planning Data, Version 3.20, or later; or
   - DICOM 3.0 in compliance with the ATC's DICOM 3.0 Conformance Statement.
2. IMRT Treatment Verification Procedures: Documentation of the IMRT planning and delivery process as well as the routine QA tests performed to insure proper functioning. The method used to conduct a check of the dose and monitor unit calculations performed by the IMRT planning system must be provided.

C. Dry Run (Benchmark) Test: A complete patient data set as specified by the treatment protocol is to be submitted to the ATC to demonstrate compliance with 3D technical requirements (see Dry Run Guidelines at http://itc.wustl.edu/). **A separate dry run test MUST be performed for each IMRT planning system used.**

1. No port films are required for the Dry Run test, as the patient's treatment is not required to be per protocol. However, if you plan on submitting your treatment verification images in digital format, you must prove that you have a compliant method of submitting these images as part of the Dry Run test.
2. **NOTE:** There is no requirement that the patient whose data is used for the Dry Run test be treated according to the protocol. This test set can be from a data set for a patient who was previously seen and/or treated (in some other fashion). The only requirement is that the CT scan be close to protocol compliant and the tumor/target volumes and critical normal structure contours be defined in compliance with the protocol and that protocol compliant treatment plans be generated and the appropriate data submitted to the ATC. Any protocol immobilization device requirement is waived for this test data set. All patient identifying data for the Dry Run test data must be removed before submission to protect patient confidentiality.

D. IMRT Phantom Dosimetry Test: A TLD dosimetry - treatment plan verification phantom experiment may be required by the specific protocol. **A separate phantom dosimetry test may be required for each IMRT planning system used. A separate phantom dosimetry test MUST be repeated if the delivery system is changed.**

IV. Protocol Requirements

A. Protocols permitting IMRT treatment delivery must be written using the nomenclature defined in the NCI IMRT Working Group Report (IMRT Collaborative Working Group: Intensity modulated radiation therapy: current status and issues of interest. Int. J. Radiat. Oncol. Biol. Phys. 51:880-914, 2001) and the International Commission on Radiation Units and Measurements (ICRU) Reports 50 and 62 for specifying the volumes of known tumor, i.e., Gross Tumor Volume (GTV), the volumes of suspected microscopic spread, i.e., Clinical Target Volume (CTV), and the marginal volumes necessary to account for setup variations and organ and patient motion, i.e., Planning Target Volume (PTV). Report 62 introduced the concept of the Planning Organ at Risk Volume (PRV), in which a margin is added around the critical structure to compensate for that organ's geometric uncertainties. The PRV

margin around the critical structure is analogous to the PTV margin around the CTV. The use of PRV concept is even more important for those cases involving IMRT because of the increased sensitivity of this type treatment to geometric uncertainties.

B. The protocol must provide a clear definition of the GTV, CTV, and the PTV.

C. The protocol must provide a clear definition of the prescription dose and dose heterogeneity allowed throughout the PTV.

D. The protocol must require that a volumetric treatment planning CT study be used to define the GTV.

E. The protocol must clearly define the organs-at-risk that are required to be contoured and provide clear guidelines for contouring each organ-at-risk defined in the study. Dose constraints for each organ-at-risk in the irradiated volume must be defined. This should include a reasonable definition of major and minor deviation for each item of interest.

F. The protocol must require that specific procedures be in place to insure correct, reproducible positioning of the patient. As a minimum, orthogonal (AP and lateral) DRRs and corresponding orthogonal portal images (film or electronic) are to be required.

G. The treatment machine monitor units generated using the IMRT planning system must be independently checked prior to the patient's first treatment. Measurements can suffice for a check as long as the plan's fluence distributions can be recomputed for a phantom geometry.

V. Protocol Data and Quality Assessment Parameters

A. Patient Data Submission: The following information is to be submitted to the ATC for each protocol patient at times specified in the protocol:

1. T2 Form: Digital Patient Information Submission Form (obtain from ATC website).

2. IMRT digital dosimetry and imaging data.
   a. Protocol compliant images (e.g. CT or MRI scan series);

   b. Protocol compliant contours using required standard names (standard structure names can be found on the ATC website) for all GTV, CTV and PTVs, and for all specified critical normal structures. They must be contoured on all slices in which each structure exists or as defined by the protocol and includes skin on ALL CT cuts;

   c. Volumetric 3-D dose distribution (**with** heterogeneity corrections) data in absolute dose for each fraction group used to deliver a protocol compliant dose. Note, a Fraction Group represents the beams and doses for a concurrently treated set of beams;

   d. DVH's computed **with** heterogeneity correction for the total dose of all dose distributions submitted for item c (summed fraction groups from item c) for all PTVs and all critical normal structures (excluding Unspecified Tissue);

   e. Any corrections to previously submitted digital data should be discussed with the ATC prior to such submission.

3. Color hardcopy isodose distribution for the axial, sagittal and coronal planes through the isocenter for the total dose plan must be submitted. If sagittal and coronal hard copy can not be generated, five axial distributions may be substituted for them (two cuts which are 2 slices superior and inferior of the superior and inferior slices containing the high dose PTV, the superior and inferior cuts containing the high dose PTV, and one through the center of the boost PTV. These dose distributions must include:
   a. A reasonable number of isodose lines should be shown which can be used to determine that the digital dose and anatomy data are properly aligned relative to each other. The prescription dose for the high-dose PTV should be displayed. If the hard copy isodose lines are in percentage, the conversion factor to convert them to absolute dose (Gy or cGy) for all delivered fractions must be indicated.

b. The above isodoses shall be superimposed over the treatment planning CT images or reconstructed planes of the planning CT images and should be in color.

4. Treatment prescription and verification images:

   a. DMLC and SMLC IMRT treatments require:

      - As specified in protocol

   b. Serial tomotherapy treatments require:

      - As specified in protocol

VI. QA Review

A. Quality Assurance of the CT Scan Data and Digital Planning Data Format

1. ATC personnel will review the CT scan data set to ensure protocol compliance with regard to both inter-slice spacing as well as the superior/inferior extents of the scan region.
2. ATC personnel will review the format of the digital treatment planning data submitted for compliance with the appropriate data exchange specification version. Deviations from compliance will be noted and, depending upon the severity of the deviation, may require a complete resubmission of the digital data set.

B. Quality Assurance of Target Volumes and Organs at Risk Volumes

1. The ATC will facilitate the review of the GTV, CTV, and PTV contours by the study chair or his/her designate on the first 5 cases submitted by each institution. After the institution has demonstrated compliance with the protocol, future cases may be spot checked only.

2. The ATC will facilitate the review of all designated critical structures contours by the study chair or his/her designate on the first 5 cases submitted by each institution. After the institution has demonstrated compliance with the protocol, future cases may be spot checked only.

C. Quality Assurance of Dose Distribution

1. ATC personnel will display, and compare with hardcopies, isodose distributions for the planes submitted to verify correct interpretation and conversion of the digital patient and dose data.
2. ATC personnel will calculate DVH's for the sum of all dose distributions submitted (each submitted distribution is for one set of concurrently treated beams) and may compare them with the digitally submitted dose-volume histograms for the PTV, designated critical structures, and unspecified tissue.
   a. There should be reasonable agreement between an individual participating institution’s DVH computations and those of the ATC. Therefore, any discrepancy between the submitting institution's DVHs and those computed by the ATC in excess of $\pm$5% (or 3 cc for small structures) in total volume or $\pm$5% (relative to the absolute structure volume) of the volume calculated to be at or above the protocol specified tolerance dose for the particular structure will need to be resolved prior to successfully completing the Dry Run Test.

D. **Dose QA Score Assignment:** Each protocol must have established criteria for evaluating the submitted treatment plan. The criteria will be published on the ATC website. An overall score will be assigned to each plan. The items involved in the scoring are the coverage and overdose of each PTV and the level of specified organ(s)-at-risk sparing. ***The largest variation encountered (None, Minor or Major) will be the overall score assigned to the plan.*** No credentialing plan (dry run) will be approved that results in a Major Variation. Plans with No Variation or Minor Variations will be approved (assuming no other significant areas of protocol non-compliance).

1. **Compliance with Prescription Dose Coverage:** A (1) No variation, (2) Minor variation (marginal coverage), and (3) Major variation (miss) criteria similar to that posted for RTOG H-0022 will be used to evaluate plan compliance.

2. **Compliance with Dose Heterogeneity**: A (1) No variation, (2) Minor variation, and (3) Major variation criteria similar to that posted for RTOG H-0022 will be used to evaluate plan compliance.

   The maximum point dose (to a volume of specified cc's) to organs at risk (and unspecified tissue) outside the $PTV_{High\ Dose}$ volume should not exceed the protocol specified allowable dose. The treating physician must carefully consider the tolerance dose/volume to each organ at risk and unspecified tissue.

Last modified: 06/19/2003 17:30:44

Appendix VIIB (9/18/03) (9/17/04)

IMRT Facility Questionnaire

Please type this form.

The following items are required before you can enter cases on each RTOG IMRT protocol supported by the Image-Guided Therapy QA Center (ITC):

1. Submit this completed Facility Questionnaire for the IMRT protocol to the:

   Image-guided Therapy Center
   4511 Forest Park Ave., Suite 200
   St. Louis, MO 63108

   E-mail: itc@castor.wustl.edu
   Phone: 314-747-5414
   FAX: 314-747-5423

2. Contact the ITC (itc@castor.wustl.edu) and request an FTP account for digital data submission

3. A successful IMRT phantom experiment must be completed and the appropriate documents sent to the RPC and the digital data with the standard set of hard copy isodoses sent to the ITC. Contact Michael Gillin, Ph.D. at the M.D. Anderson Cancer Center or the Radiological Physics Center (713-745-8989) to arrange for the phantom to be sent to you.

RTOG Protocol #:________________________________**RTOG Institution #:**__________________________

Institution Name:____

If Affiliate, Name of Member Institution:____

Date Questionnaire Submitted:_____/_____/_______

Physicist:___________________________________e-mail:______________________________________
Address:____

____

____

______

Telephone:________________________ Fax:________________________

Research Associate:________________________e-mail:________________________

Telephone:________________________

Fax:________________________

Dosimetrist:________________________e-mail:________________________

Telephone:________________________

Fax:________________________

Responsible Radiation Oncologist(s) ________________________

Telephone:________________________

e-mail:________________________

1. a. What treatment machine(s) do you use for IMRT treatments? ________________________

b. Photon energy(s)? ________________________

2 a. What form of IMRT do you use?

- ☐ SMLC (step and shoot)
- ☐ Serial tomotherapy (MIMiC)
- ☐ DMLC (sliding window)
- ☐ other ________________________

b. MLC/device used to deliver IMRT: vendor ____________

_____ (#) leaves with ____ cm leaf width at isocenter

_____ Nomos MIMiC in _______1cm mode _______2cm mode

Other : ________________________

3. What is your IMRT planning system? ________________________ Version No. ________________

4. Is your treatment planning system capable of transferring a patient's beams to a QA phantom for verification purposes?

☐ yes ☐ no

If no, how do you verify the dose distribution ________________________

____

5. What sites do you treat with IMRT?

- ☐ head and neck
- ☐ prostate
- ☐ other (please specify) ________________________

6. If you treat head and neck (H&N) patients with IMRT:

a. The total number of H&N patients treated with IMRT at your institution is _____

b. Number of H&N patients treated with IMRT in the past 12 months at your institution _____

c. The usual fraction size is ______ cGy and the usual number of fractions is ______

d. The usual beam energy is ____ MV number of fractions is ______

e. How are your H&N patients immobilized for IMRT?

☐ head-cup and mask ☐ talon

☐ foam-immobilization mold and mask ☐ other ________________________

A bite block is routinely used ☐ yes ☐ no

f. What PTV margins do you usually use for H&N IMRT patients? ______mm

g. To what isodose line are IMRT treatments for H&N patients commonly prescribed (relative to maximum dose)?

☐95% ☐90% ☐ 85% ☐80% ☐other _______

h. How do you verify field positioning relative to the patient's anatomy?

☐ orthogonal films

☐ beam films using a jaw setting that encloses all segments

☐ other (please be specific) ____

____

i. How frequently is position verification performed for H&N patients?

☐first treatment only ☐weekly ☐other _______

j. How do you verify that the field intensity patterns are delivered as planned?

____

____

____

7. If you treat prostate patients with IMRT:

a. The total number of prostate patients treated with IMRT at your institution is _____

b. Number of prostate patients treated with IMRT at your institution in past 12 months is _____

c. The usual fraction size is ______ cGy and the usual number of fractions is ______

d. The usual beam energy is _____ MV number of fractions is ______

e. How are your prostate patients immobilized for IMRT?

☐ knee sponge only ☐ thermoplastic cast

☐ knee sponge and foot holder ☐ foam-immobilization mold

☐ other ____

f. What PTV margins do you usually use for prostate patients? ______mm

g. To what isodose line are IMRT treatments for prostate patients commonly prescribed (relative to maximum dose)?

☐95% ☐90% ☐ 85% ☐80% ☐other _______

h. How do you verify field positioning relative to the patient's anatomy?

☐ orthogonal films

☐ beam films using a jaw setting that encloses all segments

☐ other (please be specific) ____

____

i. How frequently is position verification performed for prostate patients?

☐first treatment only ☐weekly ☐other _______

j. How do you verify that the field intensity patterns are delivered as planned?

____
____
____

8. Other than prostate or H&N, what site do you most commonly treat with IMRT? __________________ _______
____

a. The total number of patients treated to this site with IMRT at your institution is _____

b. The number of these patients treated with IMRT at your institution in past 12 months is _____

c. The usual fraction size is ______ cGy and the usual number of fractions is ______

d. The usual beam energy is ____MVnumber of fractions is ______

e. How are patients immobilized for these treatments? ____
____

f. What PTV margins do you usually use for this site? ______mm

g. To what isodose line are IMRT treatments for these patients commonly prescribed (relative to maximum dose)?

☐95% ☐90% ☐ 85%☐80% ☐other _______

h. How do you verify field positioning relative to the patient's anatomy?

☐ orthogonal films

☐ beam films using a jaw setting that encloses all segments

☐ other (please be specific) ____
____

i. How frequently is position verification performed for these patients?

☐first treatment only ☐weekly ☐other _______

j. How do you verify that the field intensity patterns are delivered as planned?

____
____
____

9. How do you verify that the treatment unit delivers the planned dose for individual patients?

a. <u>Absolute dose</u>

☐ point(s) measurement with

☐ion chamber (chamber size ____cc) ☐diode ☐ TLD

☐XV film ☐EDR2 film ☐ radiochromic film

☐Other: ________________________________

These absolute dose measurements are routinely performed for

☐ every field for every patient

☐ cumulative fields (i.e. total treatment) for every patient

☐ monthly as part of routine Quality Assurance

☐ only on special occasions

specify: ____

b. <u>Relative dose</u>

☐ isodose distribution with

☐XV film ☐EDR2 film ☐ radiochromic film ☐Gel dosimetry

☐other_________

Relative dose is routinely measured in _____ (#) axial planes

& in _____ (#) sagittal planes

& in ______(#)coronal planes

These relative dose measurements are routinely performed for

☐ every field for every patient

☐ cumulative fields (i.e. total treatment) for every patient

☐ monthly as part of routine Quality Assurance

☐ only on special occasions

specify: ____

c. <u>Type of QA phantom</u>:

☐ anthropomorphic phantom Vendor: ________________________________

☐ geometric phantom: ________________(material)

shape: ☐square ☐ cylinder ☐ other ____

size of phantom _____cm X _____cm X ___ cm

d. <u>For this measurement</u>

☐ the patient's beams are transferred to the QA phantom by the planning system.

☐ the patient's beams are not transferred to the QA phantom in software, but an anthropomorphic phantom is used to simulate approximate patient geometry for dose measurements.

e. The fields are delivered to the QA phantom and measured

☐ for individual fields delivered in the geometry of the treatment

☐ for cumulative fields (i.e. total treatment) delivered in the geometry of the treatment

☐ for individual fields delivered from one gantry angle (e.g. 0 or 180 degrees)

☐ for cumulative fields (i.e. total treatment) delivered from one gantry angle (e.g. 0 or 180 degrees)

f. What agreement between planned and measured doses for individual patients is considered acceptable at your institution?

For absolute dose in target volume (high dose) region ______________________________

For absolute dose in critical normal tissue region ______________________________

For absolute dose in low dose region ______________________________

For relative dose in high dose gradient region ______________________________

For relative dose in low dose gradient region

in high dose region (target) ______________________________

in low dose region ______________________________

g, Are your monitor unit calculations checked by an independent program?

☐ no ☐ yes Vendor: ______________________________

10. Are your IMRT treatments monitored by a record and verify system?

☐ no ☐ yes What system? ______________________________

11. Treatment Machine Calibration

a. Calibration Protocol: ☐ TG-21 ☐ TG-51 Other: ________________

Frequency of calibration checks: ______________________________

____

Physics Division
Quality Assurance Review Center
825 Chalkstone Ave.
Providence, RI 02908-4735
Phone: (401) 456-6500
FAX: (401) 456-6550
Email: Physics@QARC.org

Radiological Physics Center
1515 Holcombe Blvd.
Box 547
Houston, TX 77030
Phone: (713)745-8989
FAX: (713) 794-1364
Email: RPC@mdanderson.org

RTOG
1818 Market Street
Suite 1600
Philadelphia PA 19103
Phone: (215)574-3189
FAX: (215) 923-1737
Email: bmartin@phila.acr.org
RTOG Members Only

NOTE: A change in IMRT planning system (but not version number) from that listed here or a change in IMRT technique (i.e. step and shoot, sliding window, tomotherapy) requires submission of a new benchmark.

APPENDIX VIII (7/10/07)

Specimen Plug Kit* and Instructions

The Specimen Plug Kit contains a shipping tube and a dermal needle. **Note**: **Sites should not dispose of the Plug Kit.** Sites should ship the Plug Kit to the RTOG Tissue Bank to be used again.

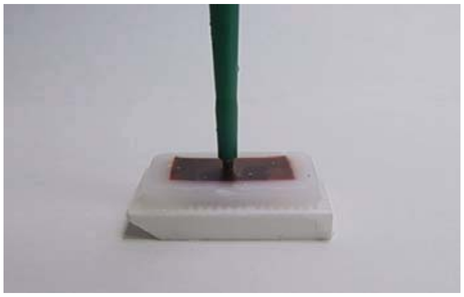

Step 1
Place the dermal needle on the paraffin block over the selected tumor area. (*Ask a Pathologist to select area with tumor.*) Push the needle into the paraffin block. Twist the needle once around to separate the plug from the block. Then pull the needle out of the block. The needle should be filled with tissue sample.

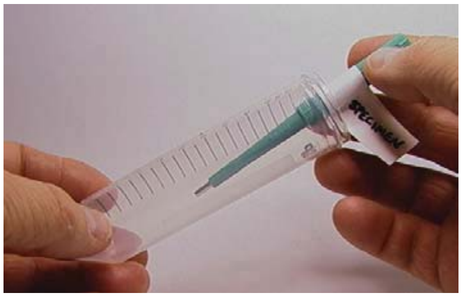

Step 2
Label dermal needle with the pathology accession number, RTOG study and case numbers. **Do not try to remove specimen from needle.**

Use a separate dermal needle for every specimen. **Do not mix specimens.** Call or e-mail the RTOG Tissue Bank for questions or for additional specimen Plug Kits.

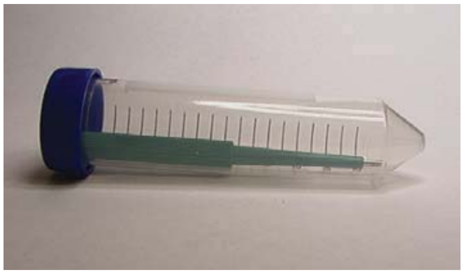

Step 3
Once specimen needle is labeled, place it in the shipping tube and mail to the address below.

The RTOG Tissue Bank will remove the specimen from the needle and embed it in a cassette, labeled with the specimen ID.

***NOTE**: If an institution is uncomfortable obtaining the plug but wants to retain the tissue block, the institution should send the entire block to the RTOG Tissue Bank. The Tissue Bank will sample a plug from the block and will return the remaining block to the institution. Institutions should indicate their request to perform the plug procedure and to return the block on the submission form.

Ship: Specimen Plug Kit, specimen in dermal needle, and all paper work to the address below:

LDS Hospital
RTOG Tissue Bank, 1st Floor North
8th Avenue and C Street
Salt Lake City, UT 84143

For questions or if you need additional Plug Kits please E-mail the RTOG Tissue Bank at:
RTOG@intermountainmail.org
Phone: (801) 408-5626
Fax: (801) 408-5020

APPENDIX IX (7/10/07)

BLOOD COLLECTION KIT INSTRUCTIONS

Instructions for use of serum, plasma, or buffy coat collection kit (collected as required by protocol):

This kit includes:
- Ten (10) 1 ml cryovials
- Biohazard bags
- Absorbent shipping material
- Styrofoam container (inner)
- Cardboard shipping (outer) box
- Pre-paid shipping label(s)

Serum (if requested):

- ❑ Using four (4) or more 1 ml cryovials, label them with the RTOG study and case number, collection date and time, and clearly mark cryovials "serum".

Process:
1. Allow one red top tube to clot for 30 minutes at room temperature.
2. Spin in a standard clinical centrifuge at ~2500 RPM for 10 minutes at room temperature.
3. Aliquot a minimum of 0.5 ml serum (optimal 1ml) into each cryovial labeled with RTOG study and case numbers, collection date/time, time point collected, and clearly mark specimen as "serum".
4. Place cryovials into biohazard bag and immediately freeze at –70 to –80° Celsius.
5. Store serum at –70 to –80° Celsius until ready to ship.
6. Ship on dry ice.

PLEASE MAKE SURE THAT EVERY SPECIMEN IS LABELED.

Plasma (If requested):

- ❑ Using three (3) or more 1 ml cryovials, label them with the RTOG study and case number, collection date and time, and clearly mark cryovials "plasma".

Process:
1. After collection, invert tube(s) multiple times to ensure adequate mixing of EDTA.
2. Centrifuge specimen(s) within one hour of collection in a standard clinical centrifuge at ~2500 RPM for 10 minutes at room temperature.
3. If the interval between specimen collection and processing is anticipated to be greater than one hour, keep specimen on ice until centrifuging is performed.
4. Carefully pipette and aliquot a minimum of 0.5 ml plasma (optimal 1 ml) into each cryovial labeled with RTOG study and case numbers, collection date/time, time point collected and clearly mark specimen as "plasma".
5. Place cryovials into biohazard bag and immediately freeze at –70 to –80° Celsius.
6. Store plasma at –70 to –80° Celsius until ready to ship.
7. Ship on dry ice.

PLEASE MAKE SURE THAT EVERY SPECIMEN IS LABELED.

Buffy coat (if requested):

For a visual explanation of Buffy coat, please refer to diagram below.

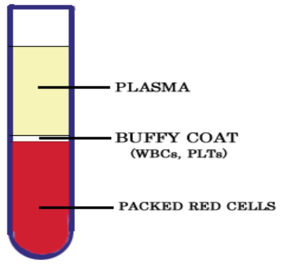

APPENDIX IX (continued)

- Using one (1) or more 1 ml cryovials, label them with the RTOG study and case number, collection date and time, and clearly mark cryovial(s) “buffy coat”.

Process:

1. Centrifuge EDTA (purple top) tube within one hour of collection in a standard clinical centrifuge at ~2500 RPM for 10 minutes at room temperature.
2. If the interval between specimen collection and processing is anticipated to be greater than one hour, keep specimen on ice until centrifuging is performed.
3. Carefully remove plasma close to the buffy coat and set plasma aside (*can be used to send plasma samples – see above instructions*).
4. Remove the buffy coat cells carefully and place into cryovials labeled “buffy coat” (*it is okay if a few packed red cells are inadvertently collected in the process*). Clearly mark the tubes with date/time of collection and time point collected.
5. Place cryovials into biohazard bag and freeze immediately at -70 to -80° Celsius.
6. Store buffy coat samples frozen (-70 to -80° Celsius) until ready to ship.
7. Ship on dry ice.

PLEASE MAKE SURE THAT EVERY SPECIMEN IS LABELED.

Shipping/Mailing:

- Ship specimens overnight **Monday-Wednesday** to prevent thawing due to delivery delays. Saturday and holiday deliveries will not be accepted.
- Include all RTOG paperwork in a sealed plastic and tape to the outside top of the Styrofoam box.
- Wrap frozen specimens of same type (i.e., all serum together, plasma together and buffy coats together) in absorbent shipping material and place each specimen type in a separate biohazard bag. Place specimen bags into the Styrofoam cooler and fill with dry ice (4-5lbs/2-2.5kg minimum). Ship ambient specimens in a separate envelope/cooler. Place Styrofoam coolers into outer cardboard box, and attach shipping label to outer cardboard box.
- *Multiple cases may be shipped in the same cooler, but make sure each one is in a separate bag.*

For questions regarding collection, shipping or to order a Blood Collection Kit, please Email RTOG@intermountainmail.org or contact the RTOG Tissue Bank by phone (801) 408-5626 or Fax at (801) 408-5020.
