# Supplementary material for: Conditional Risks of Biochemical Failure and Prostate Cancer–Specific Death in Patients Undergoing External Beam Radiotherapy: A Secondary Analysis of 2 Randomized Clinical Trials
Source: JAMA Netw Open. 2023 Sep 26;6(9):e2335069. doi: 10.1001/jamanetworkopen.2023.35069 (PMC10523164; doi:10.1001/jamanetworkopen.2023.35069)
Supplement: Supplement 2. — Trial Protocol. RTOG 0415 [file jamanetwopen-e2335069-s002.pdf]

RADIATION THERAPY ONCOLOGY GROUP

RTOG 0415

A PHASE III RANDOMIZED STUDY OF HYPOFRACTIONATED 3D-CRT/IMRT VERSUS CONVENTIONALLY FRACTIONATED 3D-CRT/IMRT IN PATIENTS WITH FAVORABLE-RISK PROSTATE CANCER

Study Chairs (9/20/07) (7/9/09)

Pathology
Mahul B. Amin, MD
Department of Pathology and Laboratory Medicine
Cedars-Sinai Medical Center
8700 Beverly Blvd., Suite 8728
Los Angeles, CA 90048
310-423-6631
FAX: 310-423-0170
aminm@cshs.org

Radiation Oncology
W. Robert Lee, MD, MS
Department of Radiation Oncology
Duke University
DUMC Box 3085
Durham, NC 27710
919-668-7342
FAX: 919-668-7345
lee00255@mc.duke.edu

Outcomes (Quality of Life and Utilities)
Deborah Watkins Bruner, RN, PhD
University of Pennsylvania
Nursing Education Building, Room 352
420 Guardian Drive
Philadelphia, PA 19104-6096
215-746-2356
FAX: 215-573-7496
wbruner@nursing.upenn.edu

Physics and Quality Assurance
Daniel Low, PhD
Washington University
Department of Radiation Oncology
Washington University School of Medicine
660 S. Euclid Avenue, Campus Box 8224
St. Louis, MO 63110
314-362-2636
FAX: 314-362-8521
low@wustl.edu

SWOG Co-Chair
Gregory P. Swanson, MD
UTHSC San Antonio
Department of Radiation Oncology & Urology
7703 Floyd Curl Drive - MC 7889
San Antonio, TX 78229-3900
210-616-5648
FAX: 210-949-5085
gswanson@ctrc.net

Activation Date: April 18, 2006
Closed Date: December 11, 2009
Version Date: July 9, 2009
Includes Amendments 1-3
(Broadcast: 8/6/09)
Update Date: January 8, 2008

Senior Statistician
Daniel Hunt, PhD
Radiation Therapy Oncology Group/ACR
1818 Market Street, Suite 1600
Philadelphia, PA 19103
215-940-8825
FAX: 215-928-0153
dhunt@acr-arrs.org

RTOG Headquarters/Statistical Center
215-574-3189
1-800-227-5463, ext. 4189

The following Cooperative Groups have endorsed this trial: SWOG. SWOG members will enroll patients to this study and submit data via the Cancer Trials Support Unit (CTSU). (9/20/07)

INSTITUTION MUST BE CREDENTIALED PRIOR TO ENROLLMENT (See Section 5.0)

This protocol was designed and developed by the Radiation Therapy Oncology Group (RTOG) of the American College of Radiology (ACR). It is intended to be used only in conjunction with institution-specific IRB approval for study entry. No other use or reproduction is authorized by RTOG nor does RTOG assume any responsibility for unauthorized use of this protocol.

This study is supported by the NCI Cancer Trials Support Unit (CTSU) [1/24/07]

Institutions not aligned with the RTOG will participate through the CTSU mechanism as outlined below and detailed in the CTSU logistical appendix.

- The **study protocol and all related forms and documents** must be downloaded from the protocol-specific Web page of the CTSU Member Web site located at http://members.ctsu.org
- Send completed **site registration documents** to the CTSU Regulatory Office. Refer to the CTSU logistical appendix for specific instructions and documents to be submitted.
- **Patient enrollments** will be conducted by the CTSU. Refer to the CTSU logistical appendix for specific instructions and forms to be submitted.
- Data management will be performed by the RTOG*.* **Case report forms** (with the exception of patient enrollment forms)**, clinical reports, and transmittals** must be sent to RTOG Headquarters unless otherwise directed by the protocol. Do not send study data or case report forms to CTSU Data Operations.
- **Data query and delinquency reports** will be sent directly to the enrolling site by the RTOG. Please send query responses and delinquent data to the RTOG and do not copy the CTSU Data Operations. Each site should have a designated CTSU Administrator and Data Administrator and must keep their CTEP AMS account contact information current. This will ensure timely communication between the clinical site and RTOG Headquarters.

INDEX

Schema

Eligibility Checklist

1.0 Introduction

2.0 Objectives

3.0 Patient Selection

4.0 Additional Pretreatment Evaluations/Management

5.0 Registration Procedures

6.0 Radiation Therapy

7.0 Drug Therapy

8.0 Surgery

9.0 Other Therapy

10.0 Tissue/Specimen Submission

11.0 Patient Assessments

12.0 Data Collection

13.0 Statistical Considerations

References

Appendix I - Sample Consent Form
Appendix II - Performance Status Scoring
Appendix III - Staging System
Appendix IV - Specimen Plug Kit and Instructions (9/20/07)
Appendix V - Blood Collection Kit Instructions (9/20/07)
Appendix VI - Cancer Trials Support Unit (CTSU) Participation Procedures (9/20/07)

RADIATION THERAPY ONCOLOGY GROUP

RTOG 0415

A PHASE III RANDOMIZED STUDY OF HYPOFRACTIONATED 3D-CRT/IMRT VERSUS CONVENTIONALLY FRACTIONATED 3D-CRT/IMRT IN PATIENTS WITH FAVORABLE-RISK PROSTATE CANCER

SCHEMA

**S T R A T I F Y**	<u>**Gleason Score**</u><br>1. Gleason 2-4<br>2. Gleason 5-6<br><br><u>**PSA**</u><br>1. < 4 ng/mL<br>2. 4- < 10 ng/mL<br><br><u>**Radiation Modality**</u><br>1. 3D-CRT<br>2. IMRT	**R A N D O M I Z E**	<u>**Arm 1**</u> ***(Minimum PTV prescription)***<br>3D-CRT or IMRT: 73.8 Gy in 41 fractions<br><br><u>**Arm 2**</u> ***(Minimum PTV prescription)***<br>3D-CRT or IMRT: 70 Gy in 28 fractions

Treatment is prescribed as a minimum to the planning target volume (PTV) to be delivered at a rate of 1.8 or 2.5 Gy/daily fraction. The PTV includes with margin a clinical target volume that encompasses the prostate only.

<u>**Patient Population**</u>**:** (See Section 3.0 for Eligibility*)*

- Histologically confirmed prostate adenocarcinoma within 180 days prior to randomization
- Clinical stage T1-2c according to the AJCC 6th edition
- Pretreatment PSA <10 ng/mL
- Gleason score must be <7
- No radical surgery or cryosurgery for prostate cancer
- No prior or planned androgen deprivation or bilateral orchiectomy

Required Sample Size: 1067

Institution # ________
RTOG 0415
Case # ________

ELIGIBILITY CHECKLIST (4/18/06)
(*page 1 of 3)*

_______(Y) 1. Is there histologically confirmed prostate adenocarcinoma within the past 180 days?

_______(Y) 2. Was there a history/physical with digital rectal examination of the prostate within 8 weeks prior to randomization?

_______(2-6) 3. What is the combined Gleason score?

______(Y) 4. Was the PSA done within 180 days prior to randomization?

____ (N) Was the PSA done within 10 days after prostate biopsy?

______ (< 10) 5. What is the PSA level?

______(T1-T2c) 6. What is the T stage (6th edition AJCC, see Appendix III)?

_______(N) 7. Is there evidence of nodal metastases?

_______(N) 8. Is there evidence of distant metastases?

_______(0-1) 9. What is the Zubrod Performance Status?

_______(N) 10. Has the patient had prior pelvic radiation, prostate brachytherapy, bilateral orchiectomy, or cytotoxic chemotherapy for prostate cancer?

_______(N) 11. Has the patient had prior radical surgery or cryosurgery for prostate carcinoma?

_______(N) 12. Has the patient had any previous hormonal therapy (LHRH agonists, anti-androgens, estrogens)?

_______(Y/N) 13. Has the patient received finasteride?

____ (Y) If yes, was it discontinued at least 30 days prior to randomization?

____ (Y) Was the PSA done at least 30 days after stopping finasteride?

_______(Y/N) 14. Has the patient received dutasteride?

____ (Y) If yes, was it discontinued at least 90 days prior to randomization?

____ (Y) Was the PSA done at least 90 days after stopping dutasteride?

_______(N) 15. Has the patient had previous or concurrent invasive cancer or lymphomatous/hematogenous malignancy within the past 5 years?

_______(Y) 16. Were pretreatment evaluations completed per Section 3.1 of the protocol?

_______(N) 17. Does the patient have any of the following severe, active comorbidities?

- Unstable angina and/or congestive heart failure requiring hospitalization within the last 6 months
- Transmural myocardial infarction within the last 6 months
- Acute bacterial or fungal infection requiring intravenous antibiotics at the time of registration
- Chronic obstructive pulmonary disease exacerbation or other respiratory illness requiring hospitalization or precluding study therapy at the time of registration
- Hepatic insufficiency resulting in clinical jaundice and/or coagulation defects; note, however, that laboratory tests for liver function and coagulation parameters are not required for entry into this protocol.
- Acquired immune deficiency syndrome (AIDS) based upon current CDC definition; note, however, that HIV testing is not required for entry into this protocol.

The following questions will be asked at Study Registration:

_______________ 1. Name of institutional person registering this case?

_______________(Y) 2. Has the Eligibility Checklist (above) been completed?

_______________(Y) 3. Is the patient eligible for this study?

_______________ 4. Date the study-specific Consent Form was signed? (must be prior to study entry)

_______________ 5. Patient's Initials (First Middle Last) [If no middle initial, use hyphen]

_______________ 6. Verifying Physician

_______________ 7. Patient's ID Number

_______________ 8. Date of Birth

_______________ 9. Race

_______________ 10. Ethnic Category (Hispanic or Latino; Not Hispanic or Latino; Unknown)

_______________ 11. Gender *(This question has been inactivated for this study)*

_______________ 12. Patient's Country of Residence

_______________ 13. Zip Code (U.S. Residents)

<u>ELIGIBILITY CHECKLIST</u> (4/18/06)(7/9/09)

_______________ 14. Patient's Insurance Status

_______________ 15. Will any component of the patient's care be given at a military or VA facility?

_______________ 16. Calendar Base Date

_______________ 17. Randomization Date: This date will be populated automatically.

____________(Y/N) 18. Tissue/blood kept for cancer research?

____________(Y/N) 19. Tissue/blood kept for medical research?

____________(Y/N) 20. Allow contact for future research?

_______________ 21. Specify Gleason Score (2-4 vs. 5-6)

_______________ 22. Specify PSA level (< 4 ng/mL vs. 4-< 10 ng/mL)

_______________ 23. Specify Radiation Modality (3D-CRT <u>or</u> IMRT)

____________(Y/N) 24. Did the patient agree to participate in the Quality of Life component of the study?

____________ If no, please specify the reason from the following:

1. Patient refused due to illness
2. Patient refused for other reason: specify _____________
3. Not approved by institutional IRB
4. Tool not available in patient's language
5. Other reason: specify_________________

The Eligibility Checklist must be completed in its entirety prior to web registration. The completed, signed, and dated Checklist used at study entry must be retained in the patient's study file and will be evaluated during an institutional NCI/RTOG audit.

Completed by _________________________________ Date ____________________

1.0 INTRODUCTION

1.1 Rationale for Hypofractionation

The optimal radiation schedule for the curative treatment of prostate cancer remains unknown.[1] Prostate cancer patients receiving external beam radiation therapy (EBRT) (teletherapy) typically are treated 5 days per week for 7-8 weeks.[2] Based on recent data some clinicians have increased the total dose of radiation by adding more treatment fractions. It is now the standard at some centers to treat men for 9-10 consecutive weeks.[3] This prolongation of treatment time increases health care costs and is less convenient for patients. In the past 5 years, a growing body of preclinical and clinical evidence has been published that suggests that a long treatment regimen with several (> 35) small (1.8-2 Gy) fractions may not represent the optimal schedule.[4-7]

Radiation-induced death for mammalian cells is classically described according to the linear quadratic equation (LQE). According to this formalism the survival rate of a given cell will depend on the overall radiation dose, the dose per fraction, and the overall treatment time. In this model, the dose-response of tumors and normal tissues to fractionated irradiation can be described according to the alpha-beta ratio **(α/ß)**. The alpha-beta ratio parameter is an indication of the fractionation sensitivity of a particular cell type. In general the alpha-beta ratio is high (≥ 10 Gy) for early-responding normal tissues (skin, mucosa) and most tumors and low (< 5 Gy) for late-responding normal tissues (spinal cord, bone). One implication of different alpha-beta ratios for tumor cells and normal tissue is that it may be possible to increase the therapeutic ratio by using unconventional fractionation schedules.

In the past 5 years several reports have been published that suggest that the alpha-beta ratio for prostate cancer is very low.[4-7] Most reports indicate that the alpha-beta ratio is between 1 and 3. If this hypothesis is in fact true, then **hypofractionated** regimens (less frequent, larger fractions) may be more efficacious and less costly. The primary goal of this non-inferiority trial is to determine if the results obtained with a hypofractionated regimen (70 Gy/28 fractions over 5.6 weeks) are not inferior to the results of a conventionally fractionated regimen (73.8/41 fractions over 8.2 weeks) in men with favorable risk prostate cancer.

1.2 Calculation of Biologically Effective Doses (BED)

To facilitate comparison between different fractionation schedules discussed in this protocol biologically effective doses (BED) will be calculated and the isoeffect model using the LQE will be used.

For the purposes of calculating the BED and isoeffective doses in this protocol two assumptions will be made: 1) complete repair occurs between fractions, and 2) there is no time factor. The calculated BED depends on the assumed alpha-beta ratio. The convention used in this protocol is to indicate the alpha-beta ratio used in the BED calculation by a subscript; e.g. $BED_{1.5}$ indicates that the BED provided is calculated assuming an alpha-beta ratio of 1.5. For example, the calculated BED for the two arms in this study are: Control arm, 73.8 Gy/41 fractions BED_{10} 87.1 Gy BED_3 118.1 Gy $BED_{1.5}$ 162.4 Gy; Experimental arm, 70 Gy/28 fractions BED_{10} 87.5 Gy BED_3 128.3 Gy $BED_{1.5}$ 186.7 Gy.

1.3 Phase I/II Trials of Hypofractionated Regimens for Prostate Cancer

The hypofractionated regimen chosen for this trial was originally described by Kupelian. Kupelian recently reported the 5-year freedom from biochemical recurrence (FFBR) and morbidity in the first 100 patients treated in this manner.[8] In this series men were treated with intensity-modulated radiation therapy (IMRT), and daily prostate localization was performed with a transabdominal ultrasound system. Patients were treated to 70 Gy delivered in 28 fractions (2.5 Gy/fraction). Patients were treated in 1998, and the median follow-up was 66 months. Fifty-one patients (51%) received androgen deprivation therapy for a period not greater than 6 months. The PTV-CTV margin was 4 mm posteriorly and 5-8 mm elsewhere. The ASTRO Consensus Definition (ACD)[9] and the RTOG Phoenix definition (nadir + 2 ng/mL) were used to report FFBR. Results were reported according to prognostic groups. The RTOG Morbidity System was used to report GI and GU morbidity.

The estimated rate of FFBR 5 years following treatment was 85% according to the ACD and 88% according to the RTOG Phoenix definition. This biochemical result was similar to a group of patients treated contemporaneously with 3D-CRT to 78 Gy/39 fractions. In the 100 men treated with hypofractionated IMRT, the rate of combined grade 2/3 late rectal morbidity was 11% at 5 years. This low level of reported morbidity may be explained by two factors: the daily target

localization allowed for very tight CTV-PTV margins, and the use of IMRT resulted in decreased volumes of normal tissue receiving high doses.

1.4 Randomized Trials of Hypofractionated Regimens

To date the preliminary results from two randomized trials examining fractionation schedules for prostate cancer have been published. The Australian trial compares 64 Gy/32 fractions (conventional schedule) to 55 Gy/20 fractions (hypofractionated schedule) in men with favorable-risk T1-2 prostate cancer.[10] The primary endpoint of this trial is morbidity. The sample size of 220 men (110 each arm) was determined to detect a difference in the frequency of mild late radiation morbidity of 20% (40% vs. 20%) with 90% power. Efficacy was a secondary endpoint. The first 120 consecutive men are included in the interim analysis. The median follow-up is 43.5 months (range 23-62 months). Two-dimensional EBRT was used in each arm; no 3D or IMRT was used. Three- or four-field techniques were used with 6-23 MV photons. Morbidity was measured with the LENT-SOMA questionnaires. GI morbidity measured with these questionnaires emphasizes six symptoms (stool frequency, stool consistency, rectal pain, mucus discharge, urgency of defecation, and rectal bleeding). GU morbidity measures four symptoms (urinary frequency, urgency, dysuria, and hematuria). Treatment efficacy was determined clinically and biochemically. PSA nadir and three consecutive rises were examined to estimate efficacy.

Of the ten symptoms measured, only the prevalence of rectal bleeding was different between the treatment arms. The prevalence of rectal bleeding 2 years following treatment was 42% in the hypofractionated arm (BED_3 105.4 Gy) and 27% in the conventionally fractionated (BED_3 106.6 Gy) arm ($p < 0.05$). The prevalence of rectal bleeding is somewhat higher than expected and may be the result of the two-dimensional methods employed. If only those patients with moderate to severe bleeding are considered there is no difference between the treatment arms (20% vs 14%, $p > 0.05$). The authors also reported on treatment efficacy. There was no difference in the nadir PSA and the PSA levels 2 years following treatment according to treatment arm. Using the ACD, the 4-year estimate of freedom from biochemical failure was 85.5% in the conventionally fractionated arm (BED_{10} 76.8 Gy; $BED_{1.5}$ 149.3) and 86.2% in the hypofractionated arm (BED_{10} 70.1 Gy; $BED_{1.5}$ 155.8).

Preliminary results of a randomized trial from Canada have been recently published. [11] The Canadian trial compares 66 Gy/33 fractions (Long arm) to 52.5 Gy/20 fractions (Short arm) in men with low- and intermediate-risk prostate cancer. The dose was prescribed to the isocenter and the prostate/seminal vesicle to block margin was 15 mm (could be reduced to 10 mm posteriorly at the discretion of the investigator). Four-field arrangement was required unless a prosthetic hip mandated a three-field approach. Most patients were treated with CT information, but IMRT was not performed.

In this trial the 5 year rate of failure (biochemical or clinical) is higher in the Short arm compared to the Long arm (59.95% vs. 52.95%; HR 1.18 [0.99-1.41], $p < 0.05$). At first glance this would appear to suggest that hypofractionated regimens are inferior compared to a conventionally fractionated regimen, but the two arms were not designed to be isoeffective. In fact, the biologically effective dose of the Short arm is consistently less than the Long arm until the alpha-beta ratio reaches a value of < 1 (See Table). The results of the Canadian trial, therefore, are not inconsistent with an alpha-beta ratio for prostate cancer of 1.5. At a median follow-up of 5.7 years there is no difference in 5-year actuarial rate of late grade 3+ GI/GU toxicity between the two arms.

Canadian Hypofractionation Study	BED_{10}	BED_3	$BED_{1.5}$
Long Arm			
66 Gy/33 fractions	79.2	110	154
Short Arm			
52.5 Gy/20 fractions	66.3	98.4	144

1.5 Trial Justification

If the hypofractionated arm is not found to be inferior, there will be two important implications. First, the standard of care for EBRT in men with favorable-risk prostate cancer will change from a 7- to 8-week regimen to a 5.5-week regimen, with significant savings in health care resources

and increased patient convenience. Secondly, a more lasting result will be the validation of clinical research endeavors that attempt to exploit the low alpha-beta ratio of prostate cancer. In short, if the hypofractionated regimen is not inferior then the paradigm for external radiation of prostate cancer will change to more abbreviated radiation schedules.

Further, if noninferiority is demonstrated between arms, it will be important to capture and report both early and late toxicities and patient reported quality of life (QOL) outcomes. Risks and benefits can be summed in a quality-adjusted survival analysis. Furthermore, if the gain in outcomes favors hypofractionation resource savings can be assessed in future cost-utility analyses.

Randomized hypofractionation trials with slightly different eligibility criteria are ongoing in Canada and at Fox Chase Cancer Center. The NCI-Canada trial is designed for patients with intermediate-risk disease, and the doses on each arm are slightly higher (P. Warde, personal communication January 2004). The Fox Chase trial includes men with intermediate- and high-risk disease and allows for androgen deprivation in men with unfavorable features.[12] The proposed trial is the only trial of hypofractionation for men with favorable-risk disease.

1.6 Patient Selection

This trial is restricted to those patients with favorable-risk prostate cancer (T1-2, and PSA < 10 ng/mL, and Gleason score 2-6). This population of patients is growing, as PSA screening continues and men with advanced tumors have already been identified. The risk of seminal vesicle and lymph node involvement in eligible patients is low enough to exclude these structures from the treatment volume, making a hypofractionated regimen more appealing.

1.7 Dose Selection

The control arm on this trial is similar to the experimental arm in a recent randomized dose-escalation study. In the dose-escalation study reported by Pollack, 78 Gy delivered to the isocenter was found to be superior to 70 Gy delivered to the isocenter in patients with T1-3 prostate cancer.[13] The control arm of this study delivers 73.8 Gy to the PTV; this results in an isocenter dose of approximately 78 Gy. In favorable-risk patients 73.8 Gy/41 fractions is felt to represent a strong standard. The RTOG has a large experience with morbidity at this dose (94-06) using 3D-CRT and the morbidity is acceptable.

The dose and fractionation chosen for the experimental arm is identical to that reported by Kupelian. The RTOG has not completed any multi-institutional phase I/II trials with hypofractionated regimens. The RTOG feels that the experiences reported by Kupelian[8] are generalizable to our membership, with important caveats described below.

The Kupelian regimen appears well tolerated provided daily target localization, narrow treatment margins, and conformal methods are used. Unlike any previous RTOG trial of prostate cancer this study will require daily target localization. Additionally, narrow margins and highly conformal techniques are mandated.

Because of the growing availability of IMRT and the desire to potentially further reduce normal tissue radiation dose volumes, the RTOG GU, Medical Physics, and Image-Guided Radiation Therapy committees have agreed that IMRT is an appropriate modality to be used on this clinical trial. The study will require a *minimum* dose prescription to the PTV with Arm 1 patients receiving 73.8 Gy in 41 x 1.8 Gy fractions and Arm 2 patients receiving 70 Gy in 28 x 2.5 Gy fractions.

1.8 Collection of Tissue for Tissue Banking

The RTOG has been collecting pretreatment diagnostic tissue from all of the prostate cancer protocols over the last 10 years. A number of histologic, cell kinetic/proliferation, and molecular markers are under investigation, with several showing promise for the stratification of patients in future trials. This large randomized study presents an excellent opportunity for the collection of diagnostic biopsy specimens that will be assayed for various cytogenetic or gene expression abnormalities. Correlating these findings with clinical outcome in a group of men treated with radiation alone may help increase our understanding of radiation sensitivity or resistance.

Retrospective analyses of several tissue biomarkers will also be performed. The biomarkers currently under study include Ki67, p53, bcl-2, bax, p21, pRb, p16, COX-2, EGFR, and VEGF.[14,15] All of these markers show promise for providing prognostic information that compliments the standard clinical parameters of PSA, Gleason score, and stage. Since diagnostic tissue will be

limited, a final selection of the most promising markers will be made upon the completion of the ongoing studies involving the completed protocols 86-10, 92-02, and 94-13. Approximately 10 years will be required for the protocol to mature and by that time, a clearer definition of the markers to be studied will be evident. The goal will be to analyze approximately 5-10 biomarkers from the pretreatment diagnostic material.

1.9 Single Nucleotide Polymorphisms (SNPS) and Normal Tissue Toxicity (9/20/07)

RT produces its biological effects mainly through the generation of short lived but highly reactive DNA radicals that evolve into stable/long-lived DNA lesions such as DSBs[16] or through interactions with the plasma membrane[17], leading to cell death. The total number of gene products currently known to be involved in determining cellular radiosensitivity is well over 100 and growing.[18] Several groups have reported analysis of genetic variants of individual candidate genes potentially implicated in normal tissue radiosensitivity.[19,20] A more powerful search approach, in the post-genome era, would be to screen patients for a large number of genes that could impact on radiosensitivity. Variations in the sequence of the human genome can comprise repeating sequences such as variable number of tandem repeats (VNTRs), short tandem repeats (STRs) and SNPs.[21] Although the human genome is ~99.9% identical among individuals, the ~0.1% variations (the vast majority of which are SNPs) tend to be heritable and stable.[22] It is postulated that these variations in the genome explain phenotypic differences between individuals and may also serve as a genetic blueprint for susceptibility to disease and cellular responses to pharmacologic agents.[23,24] SNP-types associated with a higher risk of radiation-induced normal tissue toxicity would comprise a predictive molecular signature of radiation injury, and would have broad applicability in patient selection for radical radiotherapy.

Several groups have reported preliminary results in their analysis of the association between candidate SNPs and late toxicity after RT for breast cancer.[25-30] An association between TGFB1 -509T and +869C alleles and fibrosis was found by Quarmby et al, while Andreassen et al found TGFB1 position -509 and codon 10 to be associated with fibrosis. The latter study also found associations between other DNA damage-related SNPs (SOD2 (codon 16), XRCC3 (codon 241), XRCC1 (codon 399)) and clinical late toxicity. Recently, in a different breast cancer patient cohort, Andreassen et al[29] found statistically significant associations between the TGFB1 codon 10 Pro allele (P=0.005) as well as the TGFB1 position -509 T allele (P=0.018) and increased risk of late breast fibrosis as indicated by breast appearance. The functional significance of either the TGFB1 codon 10 Pro allele or the TGFB1 position -509 T allele is currently unclear. However, recently Andreassen et al[31] failed to replicate these earlier associations in a study where DNA was obtained from formalin fixed paraffin embedded tissue samples in a different cohort of breast cancer patients. In order to avoid false positive associations, SNP-association studies should be validated in larger, well-defined cohorts of homogeneously treated patients.

The correlation of SNPs and pelvic normal tissue toxicity was reported by De Ruyck et al.[32,33] They examined SNPs in XRCC1, XRCC3, TGFB1 position -509, TGFB1 codon 10 and OGG1. Patients with three or more risk alleles in XRCC1 and XRCC3 had a significantly increased risk of developing late pelvic GI/GU toxicity (odds ratio 10.10, p = 0.001). Damaraju et al[34] analysed 53 SNPs in BRCA1, BRCA2, ESR1, XRCC1, XRCC2, XRCC3, NBS1, RAD51, RAD52, LIGIV, HAP1, ATM, BCL2, TGFβ-1, MSH6, XPD (ERCC2), XPF (ERCC4), GRL, CYP1A1, CYP2C19, CYP3A5, CYP2D6, CYP11B2, and CYP17 genes from a cohort of 83 men who had undergone 3-dimensional conformal RT for prostate cancer. Significant univariate associations with late rectal or bladder toxicity (grade 2+) were found for XRCC3 A>G 5' UTR NT 4541, LIGIV T>C Asp568Asp, MLH1 C>T, Val219Ile, CYP2D6*4 G>A splicing defect, mean rectal and bladder dose, dose to 30% of rectum or bladder, and age <60 years. In a Cox multivariate analysis, significant associations with toxicity were found for LIGIV T>C, Asp568Asp; XPD G>A, Asp711Asp; CYP2D6*4 G>A, splicing defect; mean bladder dose >60 Gy, and dose to 30% of rectal volume >75 Gy. These data suggest an association between candidate SNPs and late pelvic radiation toxicity.

1.9.1 Proposal for Banking of Buffy Coat Specimens for SNP Analysis

In order to search for a genomic signature correlated with a higher propensity to normal tissue radiation damage, it is appropriate to propose a broad-based genetic (SNP) analysis for candidate genes. The working hypothesis is that toxicity (rectum and/or bladder in the case of pelvic sites; skin and subcutaneous tissue in the case of breast) will be correlated to a patient's genetic makeup measured as SNPs in a select group of candidate genes. The criteria for selecting SNPs should be based on published evidence for the various genes implicated or

previously demonstrated to be involved in RT-induced tissue damage and repair pathways. Genomic DNA for SNP analysis can be most effectively isolated from buffy coat leukocytes using standard procedures. Banking of buffy coat leukocytes can be performed at any time in the patient's trajectory, whether before, during or after treatment.

1.10 Health-Related Quality of Life (HRQOL) (9/20/07)

In a noninferiority study where traditional prostate cancer outcomes of disease-free survival, progression, and overall survival are hypothesized to be similar, the outcomes of toxicity, health related quality of life (HRQOL) and resources gain an importance.

These later outcomes will play a significant role in patient, clinician and possibly even policy interpretations of the results of this study. As just one example, in a study to identify what factors men consider important when choosing treatment for localized prostate cancer, the advantages many men cited for choosing radiotherapy over radical prostatectomy were: evidence in favor of EBRT, short duration of therapy for brachytherapy, and less incontinence. The most frequently patient cited disadvantage of EBRT was the long duration of therapy.[35] One potential outcome of this study would be an evidence-based rationale for shorter duration of therapy. Therapy that has no more toxicity for a course of 5.6 weeks compared to 8.2 weeks would have obvious implications for patient decision-making and resource savings.

The previous statement would be true only if there is no increase in toxicity on the experimental arm. While toxicity will be scored using standard CTCAE criteria, it has been well documented that for more subjective parameters (e.g., sexual function, fatigue, anxiety or depression, etc.) patient-reported outcomes are more reflective of the patient experience and sometimes identify even more objective symptoms than CTCAE documents.[36-38] To supplement CTCAE and address HRQOL this trial will compare the treatment arms for differences in prostate cancer HRQOL outcomes (as measured by change over time in the Expanded Prostate Cancer Index Composite [EPIC]. EPIC is a prostate cancer HRQOL instrument that measures a broad spectrum of urinary, bowel, and sexual symptoms related to radiotherapy.[39] The patient-completed EPIC companion questionnaire, the Utilization of Sexual Medications/Devices, will be collected to provide a context for interpreting the sexual domain score of the EPIC questionnaire.

Further, the assessment of the primary endpoint, disease-free survival, may have significant implications for HRQOL. There is some evidence that biochemical recurrence, in and of itself, leads to increased anxiety and reduced QOL in men following therapy for prostate cancer.[40-42] A therapy that increases disease-free survival can be expected to reduce the utilization of salvage androgen deprivation therapy, reduce anxiety in men following treatment, and lead to increased QOL in men who do not experience biochemical recurrence. Therefore, anxiety and depression will be measured with the Hopkins Symptom Checklist (HSCL-25).

Finally, almost every incremental improvement in therapy comes at a cost. The cost is both financial and experienced in terms of QOL. Measurement of primary outcomes such as disease-free survival as well as the most important aspects of human functioning and QOL will permit a summary equation allowing for differences in QOL, clinical outcomes, and cost to be incorporated into one equation. This equation is the Quality Adjusted Life Year (QALY) and a study-specific modification, the Quality Adjusted Disease-Free Survival Year (QADFSY). The QALY has been modified in a similar manner for different treatments where survival is not the primary outcome. Much of the work in modifying the QALY began in ophthalmology, where sight-years, not life-years, is the outcome of interest. Examples of modifications to the QALY have included incremental cost per vision-year gained to assess the cost-effectiveness of photodynamic therapy with verteporfin for age-related macular degeneration,[43] costs per sight-year saved with screening for diabetic retinopathy,[44] cost-utility analysis for treatments of retinal detachment associated with severe proliferative vitreoretinopathy,[45] and the cost-utility of cataract surgery.[46] However, the QALY has been used in other studies where survival is not the primary outcome of interest, such as the cost-effectiveness of memantine in the treatment of patients with moderately severe to severe cognitive impairment from Alzheimer's[47] and cochlear implantation for patients unable to gain effective speech recognition with hearing aids.[48] We will report the quality-adjusted outcome of this study in both QALYs and QADFSYs. QALYs and QADFSYs are calculated by adjusting (weighting) outcomes of survival and FFBR by HRQOL as measured with the EQ-5D.

The EQ-5D instrument is intended to complement other forms of QOL measures, and it has been developed to generate a *generic* cardinal index of health, thus giving it considerable potential for use in economic evaluation. The EQ-5D has been used across numerous disease sites including cancer. For example, the EQ-5D mean score for 95 patients with non-small cell lung cancer (93% male, mean age 62 years) was 0.58 (SD 0.32) as measured by the questionnaire and 0.58 (SD 0.20) as measured by the visual analogue scale (VAS) version.[49] The EQ-5D has been used to assess QALYs and the economic value of prostate cancer screening,[50] as well as treatment of pain related to prostate cancer metastasis.[51] Further, the EQ-5D was used in a recent study to estimate the economic value of the welfare loss due to prostate cancer pain by estimating the extent to which pain affects HRQOL among patients with prostate cancer. Health status and economic outcomes were modeled among a well-defined population of 200,000 Swedish prostate cancer patients. Health utility ratings (using the EQ-5D) were obtained from a subset of 1,156 of the prostate cancer patients. A descriptive model showed that optimal treatment that would reduce pain to zero during the whole episode of disease would add on average 0.85 QALYs to every man with prostate cancer; the economic value of this welfare loss due to prostate cancer pain was approximately $121,240,000 per year.[52] If the primary hypothesis is supported we will report a cost-utility analysis using $U.S. in the numerator and QALYs and QADFSYs in the denominator. We will model costs using Medicare reimbursement and measure utilities with the brief 5-item EQ-5D.

2.0 OBJECTIVES

2.1 Primary Objective

To determine if hypofractionated 3D-CRT/IMRT (70 Gy in 28 fractions over 5.6 weeks) will result in disease-free survival that is no worse than DFS following conventionally fractionated 3D-CRT/IMRT (73.8 Gy in 41 fractions over 8.2 weeks) in patients treated for favorable-risk prostate cancer.

2.2 Secondary Objectives (9/20/07)(7/9/09)

2.2.1 To determine if hypofractionated 3D-CRT/IMRT will result in local progression, disease-specific survival, freedom from biochemical recurrence (FFBR), and overall survival that are no worse than that observed following conventionally fractionated 3D-CRT/IMRT in patients treated for favorable-risk prostate cancer.

2.2.2 To determine the incidence of GI and GU toxicity in patients treated with each of the regimens described above.

2.2.3 To prospectively collect diagnostic biopsy samples for future biomarker analyses

2.2.4 To assess the degree, duration, and significant differences of disease-specific HRQOL decrements among treatment arms using EPIC.

2.2.5 To assess whether anxiety/depression is decreased with therapy that improves disease-free survival as measured by the HSCL-25

2.2.6 To assess whether incremental gain in disease-free survival outweighs decrements in the generic domains of HRQOL (i.e., mobility, self care, usual activities, pain/discomfort, and anxiety/depression). This aim is reported as a Quality Adjusted Life Year (QALY), and for this study the Quality Adjusted Disease-Free Survival Year (QADFS). The QALY and QADFS-Year will be compared between treatment arms and to the literature.

2.2.7 To conduct a cost-utility analysis only if the primary objective is supported.

2.2.8 To collect paraffin-embedded tissue block, serum, plasma, and buffy coat cells for future translational research analyses.

3.0 PATIENT SELECTION

3.1 Conditions for Patient Eligibility (9/20/07)

3.1.1 Histologically confirmed prostate adenocarcinoma within 180 days of randomization

3.1.2 History/physical examination with digital rectal examination of the prostate within 8 weeks prior to registration

3.1.3 Histological evaluation of prostate biopsy with assignment of a Gleason score to the biopsy material; Gleason scores will be divided into 2-4 (well differentiated) and 5-6 (moderately differentiated) for stratification

3.1.4 Clinical stage T1-2c (AJCC 6th edition)

3.1.5 PSA < 10 ng/mL within 180 days prior to registration. PSA should not be obtained for at least 10 days after prostate biopsy. (Every effort should be made to obtain all serum PSA values

obtained in the 1 year prior to treatment to allow for calculation of PSA kinetics) The type of PSA assay (e.g., Abbott) should be recorded on the data forms.

3.1.5.1 For those patients who used finasteride and are not excluded per Section 3.2.7, PSA should not be obtained until 30 days after stopping finasteride.

3.1.5.2 For those patients who used dutasteride and are not excluded per Section 3.2.8, PSA should not be obtained until 90 days after stopping dutasteride.

3.1.6 Zubrod performance status 0-1

3.1.7 Age ≥ 18

3.1.8 Patient must sign study specific informed consent prior to randomization.

3.2 Conditions for Patient Ineligibility (4/18/06)

3.2.1 Prior or concurrent invasive malignancy (except non-melanomatous skin cancer) or lymphomatous/hematogenous malignancy unless continually disease free for a minimum of 5 years. (For example, carcinoma *in situ* of the bladder or oral cavity is permissible)

3.2.2 Evidence of distant metastases

3.2.3 Regional lymph node involvement

3.2.4 Previous radical surgery (prostatectomy) or cryosurgery for prostate cancer

3.2.5 Previous pelvic irradiation, prostate brachytherapy, or bilateral orchiectomy

3.2.6 Previous hormonal therapy, such as LHRH agonists (e.g. goserelin, leuprolide), anti-androgens (e.g., flutamide, bicalutamide), estrogens (e.g., DES), or surgical castration (bilateral orchiectomy)

3.2.7 Use of finasteride within 30 days prior to randomization. PSA should not be obtained prior to 30 days after stopping finasteride

3.2.8 Use of dutasteride within 90 days prior to randomization. PSA should not be obtained prior to 90 days after stopping dutasteride

3.2.9 Previous or concurrent cytotoxic chemotherapy for prostate cancer

3.2.10 Severe, active comorbidity, defined as follows:

3.2.10.1 Unstable angina and/or congestive heart failure requiring hospitalization within the last 6 months

3.2.10.2 Transmural myocardial infarction within the last 6 months

3.2.10.3 Acute bacterial or fungal infection requiring intravenous antibiotics at the time of registration

3.2.10.4 Chronic obstructive pulmonary disease exacerbation or other respiratory illness requiring hospitalization or precluding study therapy at the time of registration

3.2.10.5 Hepatic insufficiency resulting in clinical jaundice and/or coagulation defects; note, however, that laboratory tests for liver function and coagulation parameters are not required for entry into this protocol. (Patients on Coumadin or other blood thinning agents are eligible for this study.)

3.2.10.6 Acquired immune deficiency syndrome (AIDS) based upon current CDC definition; note, however, that HIV testing is not required for entry into this protocol. The need to exclude patients with AIDS from this protocol is necessary because the treatments involved in this protocol may be significantly immunosuppressive. Protocol-specific requirements may also exclude immunocompromised patients.

4.0 PRETREATMENT EVALUATIONS/MANAGEMENT (9/20/07)

Note: This section lists baseline evaluations needed before the initiation of protocol treatment that do not affect eligibility.

4.1 QOL Evaluations (For patients who consent to this component of the study) [7/9/09]

4.1.2 EPIC, HSCL-25, EQ5D, and the Utilization of Sexual Medications/Devices

4.2 Highly Recommended Evaluations/Management

4.2.1 Urethrogram at the time of simulation or CT scan for treatment planning (See Section 6.3.2)

4.2.2 Baseline testosterone

4.2.3 Baseline alkaline phosphatase

5.0 REGISTRATION PROCEDURES

5.1 Pre-Registration Requirements

5.1.1 *Pre-Registration Requirements for IMRT Treatment Approach* **(7/9/09)**

In order to utilize IMRT, the institution must have met technology requirements and have provided baseline physics information. Instructions for completing these requirements or determining if they already have been met are available on the Radiological Physics Center

(RPC) web site. Visit http://rpc.mdanderson.org/rpc and select “Credentialing” and “Credentialing Status Inquiry”.

An IMRT phantom study with the RPC must be successfully completed (if the institution has not previously met this IMRT credentialing requirement). Instructions for requesting and irradiating the phantom are available on the RPC web site at http://rpc.mdanderson.org/rpc/; select “Credentialing” and “RTOG”. Upon review and successful completion of the phantom irradiation, the RPC will notify both the registering institution and RTOG Headquarters that the institution has completed this requirement. Subsequently, RTOG Headquarters will notify the institution that the site can enroll patients on the study.

The institution or investigator must complete a new IMRT Facility Questionnaire and send it to RTOG for review prior to entering any cases, and/or set up an SFTP account for digital data submission, both of which are available on the Image-Guided Center (ITC) web site at http://atc.wustl.edu. Upon review and successful completion of the “Dry-Run” QA test, the ITC will notify both the registering institution and RTOG Headquarters that the institution has successfully completed this requirement. RTOG Headquarters will notify the institution when all requirements have been met and the institution is eligible to enter patients onto this study.

5.1.2 *Pre-Registration Requirements for 3D-CRT Treatment Approach* **(7/9/09)**

5.1.2.1 Only institutions that have met the technology requirements and that have provided the baseline physics information that are described in 3D-CRT Quality Assurance Guidelines may enter patients to this study.

5.1.2.1.1 The new Facility Questionnaire (one per institution, available on the ATC website at http://atc.wustl.edu) is to be sent to RTOG for review prior to entering any cases. Upon review and successful completion of a “Dry-Run” QA test, the ITC will notify both the registering institution and RTOG Headquarters that the institution has successfully completed this requirement. RTOG Headquarters will notify the institution when all requirements have been met and the institution is eligible to enter patients onto this study. Institutions that have previously enrolled patients on 3DCRT trials of this same disease site may enroll patients on this study without further credentia**ling.**

5.1.3 Regulatory Pre-Registration Requirements **(7/9/09)**

5.1.3.1 **U.S. and Canadian institutions** must fax copies of the documentation below to the CTSU Regulatory Office (215-569-0206), along with the completed CTSU-IRB/REB Certification Form, http://www.rtog.org/pdf_file2.html?pdf_document=CTSU-IRBCertifForm.pdf, prior to registration of the institution’s first case:

- IRB/REB approval letter;
- IRB/REB approved consent (English and native language versions*)
  ***Note**: Institutions must provide certification of consent translation to RTOG Headquarters
- IRB/REB assurance number

5.1.3.2 *Pre-Registration Requirements FOR CANADIAN INSTITUTIONS*

5.1.3.2.1 Prior to clinical trial commencement, Canadian institutions must complete and fax to the CTSU Regulatory Office (215-569-0206) Health Canada’s Therapeutic Products Directorates’ Clinical Trial Site Information Form, Qualified Investigator Undertaking Form, and Research Ethics Board Attestation Form.

5.1.3.3 *Pre-Registration Requirements FOR NON-CANADIAN INTERNATIONAL INSTITUTIONS*

5.1.3.3.1 **For institutions that do not have an approved LOI for this protocol:**
International sites must receive written approval of submitted LOI forms from RTOG Headquarters prior to submitting documents to their local ethics committee for approval. See http://www.rtog.org/pdf_forms.html?members/forms=Intl_LOI_Form.doc

5.1.3.3.2 **For institutions that have an approved LOI for this protocol:**
All requirements indicated in your LOI Approval Notification must be fulfilled prior to enrolling patients to this study.

5.2 Registration

Patients can be registered only after eligibility criteria are met.

Institutions must have an RTOG user name and password to register patients on the RTOG web site. To get a user name and password:

- The Investigator must have completed Human Subjects Training and been issued a certificate (Training is available via http://cme.cancer.gov/clinicaltrials/learning/humanparticipant-protections.asp).
- The institution must complete the Password Authorization Form at www.rtog.org/members/webreg.html (bottom right corner of the screen), and fax it to 215-923-1737. RTOG Headquarters requires 3-4 days to process requests and issue user names/passwords to institutions.

An institution can register the patient by logging onto the RTOG web site (www.rtog.org), going to “Data Center Login" and selecting the link for new patient registrations. The system triggers a program to verify that all regulatory requirements (OHRP assurance, IRB approval) have been met by the institution. The registration screens begin by asking for the date on which the eligibility checklist was completed, the identification of the person who completed the checklist, whether the patient was found to be eligible on the basis of the checklist, and the date the study-specific informed consent form was signed.

Once the system has verified that the patient is eligible and that the institution has met regulatory requirements, it assigns a patient-specific case number. The system then moves to a screen that confirms that the patient has been successfully enrolled. This screen can be printed so that the registering site will have a copy of the registration for the patient’s record. Two e-mails are generated and sent to the registering site: the Confirmation of Eligibility and the patient-specific calendar. The system creates a case file in the study’s database at the DMC (Data Management Center) and generates a data submission calendar listing all data forms, images, and reports and the dates on which they are due.

If the patient is ineligible or the institution has not met regulatory requirements, the system switches to a screen that includes a brief explanation for the failure to register the patient. This screen can be printed.

(7/9/09) Institutions can contact RTOG web support for assistance with web registration: websupport@acr-arrs.org **or** 800-227-5463 ext. 4189 **or** 215-574-3189.

In the event that the RTOG web registration site is not accessible, participating sites can register a patient by calling RTOG Headquarters, at (215) 574-3191, Monday through Friday, 8:30 a.m. to 5:00 p.m. ET. The registrar will ask for the site’s user name and password. This information is required to assure that mechanisms usually triggered by web registration (e.g., drug shipment, confirmation of registration, and patient-specific calendar) will occur.

6.0 RADIATION THERAPY (7/9/09)

[IMRT is allowed for institutions credentialed by the RTOG for IMRT.]

NOTE: Protocol treatment must begin within 6 weeks after registration.

6.1 Technical Factors

6.1.1 Megavoltage equipment is required with effective photon energies ≥ 6 MV.

6.2 Localization, Simulation and Immobilization

6.2.1 A urethrogram is recommended, but not required, to establish the most inferior portion of the prostate. If the urethrogram is not done with the planning CT scan, then an AP simulation radiograph with urethrogram, if performed, can be submitted with the planning CT.

6.2.2 A treatment planning CT scan will be required to define tumor, clinical, and planning target volumes and the critical normal structures (See Section 6.3). The treatment planning CT will be acquired with the patient set up in the same position as for daily treatments. Each patient will be positioned in the supine position. The CT scan of the pelvis should start at or above the iliac crest down to the perineum. All tissues to be irradiated must be included in CT scan. CT scan thickness should be ≤ 0.5 cm through the region that contains the target volumes (i.e., from the bottom of the sacroiliac joints down to the penile urethra). The regions above and below the target volume region may be scanned with slice thickness ≤ 1.0 cm.

It is advised that extreme bladder or rectal filling not be present at the time of the planning CT scan. A distended bladder or rectum can introduce a systematic patient positioning error that may increase the probability of missing the CTV. An enema before the planning CT scan and

use of a hollow (robnel) catheter to evacuate flatus will empty the rectum, thereby allowing a narrow posterior PTV margin (~5 mm) to account mainly for set up errors.

The GTV, CTV, and PTV (see Section 6.3), and normal tissues must be outlined on all CT slices in which the structures exist. For patients receiving forward planned 3D-CRT, beam's eye view display must be used to design beam aperture.

6.2.3 Daily target localization (fiducial markers, transabdominal ultrasound or other) is required for this protocol.

6.3 Treatment Planning/Target Volumes

6.3.1 The definition of volumes will be in accordance with the ICRU Report #50: Prescribing, Recording, and Reporting Photon Beam Therapy.

6.3.2 The Gross Tumor Volume **(GTV)** is defined by the physician as all known disease as defined by the planning CT, urethrogram, and clinical information. The GTV for the purposes of this protocol is the prostate only. If a urethrogram is used, the GTV will encompass a volume inferiorly 5-10 mm superior to the tip of the dye and no less than the entire prostate. Prostate dimensions should be defined as visualized on CT scan.

6.3.3 The Clinical Target Volume **(CTV)** is the GTV plus areas considered to contain microscopic disease, delineated by the treating physician, and is defined as the GTV (prostate) in this protocol.

6.3.4 The Planning Target Volume **(PTV)** will provide a margin around the CTV to compensate for the variability of treatment set up and internal organ motion. A minimum of 4 mm around the CTV is required to define the PTV. Superior and inferior margins (capping) should be 4-10 mm depending on the thickness and spacing of the planning CT scan. Careful consideration should be made when defining the 4-10 mm margin in three dimensions.

6.3.5 Treatment will be given only to the PTV using three-dimensional conformal fields shaped to exclude as much of the bladder and rectum as possible. Field arrangements will be determined by 3D planning to produce the optimal conformal plan in accordance with volume definitions. The treatment plan used for each patient will be based on an analysis of the volumetric dose including dose-volume histogram (DVH) analyses of the PTV and critical normal structures.

6.3.6 Critical Normal Structures **(4/18/06) (9/20/07)**

Custom shielding or multileaf collimation must be used in conjunction with conformal planning to restrict the dose to the normal structures. Dose-volume histograms (DVHs) must be generated for all critical normal structures and the unspecified tissues (see Section 6.3.10). Portions of the bladder and rectum will, by necessity, receive the full dose to the PTV; however, careful 3D planning must be performed to ensure that the volume of the bladder and rectum receiving the full dose is kept to a minimum.

Based upon a review of patient dosimetry on dose level 3 of RTOG 94-06, the following normal tissue guidelines should be followed:

Arm 1

Normal organ limit	No more than 15% volume receives dose that exceeds	No more than 25% volume receives dose that exceeds	No more than 35% volume receives dose that exceeds	No more than 50% volume receives dose that exceeds
Bladder Constraint	80 Gy	75 Gy	70 Gy	65 Gy
Rectum Constraint	75 Gy	70 Gy	65 Gy	60 Gy
Penile Bulb	Mean dose less than or equal to 52.5 Gy			

Arm 2 (Assumes alpha-beta for rectum bladder is 3)

Normal organ limit	No more than 15% volume receives dose that exceeds	No more than 25% volume receives dose that exceeds	No more than 35% volume receives dose that exceeds	No more than 50% volume receives dose that exceeds
Bladder Constraint	79 Gy	74 Gy	69 Gy	64 Gy

Rectum Constraint	74 Gy	69 Gy	64 Gy	59 Gy
Penile Bulb	Mean dose less than or equal to 51 Gy			

Fisch has reported a lower incidence of erectile dysfunction in patients who received a mean dose of 52.5 Gy or less to the penile bulb on RTOG 94-06(18). This dose value represents a treatment planning guideline and not a clinical study constraint. Care should be taken not to shield the penile bulb at the expense of adequate coverage of the PTV in this study.

6.3.7 The prescription dose is the minimum dose to the PTV (defined in Section 6.5.1). The maximum dose to the PTV should not exceed the prescription dose by more than 7% (inhomogeneity ≤ 7%) and will be scored as no variation: ≤ 7%; minor variation: > 7 to ≤ 10%; major variation: > 10%. It is expected that IMRT may result in more heterogeneity in dose coverage than forward planned 3D-CRT. Minor variations as described are acceptable.

6.3.8 Forward Planned 3D-CRT or IMRT

Prescription dose to the PTV shall be according to the following dose schema delivered in 1.8 Gy or 2.5 Gy minimum dose fractions. All fields treated once daily, 5 fractions per week.

ARM 1: 73.8 Gy in 41 fractions. No more than 2% of the PTV may receive less than 73.8 Gy.

ARM 2: 70 Gy in 28 fractions. No more than 2% of the PTV may receive less than 70 Gy.

Dose Goal (Prescription)	Minimum PTV dose (encompassing ≥ 98% of PTV)	Minimum CTV dose (encompassing ≥ 100% of CTV)	Maximum dose to PTV (No variation)	Maximum PTV dose to PTV (Minor variation)	Maximum PTV dose to PTV (Major variation)
ARM 1	73.8 Gy	73.8 Gy	79 Gy	81.2 Gy	>81.2 Gy
ARM 2	70 Gy	70 Gy	74.9 Gy	77 Gy	>77 Gy

[1] The maximum dose must not be within an "Organ at Risk" such as the Rectum, Bladder, or Penile Bulb

GTV = Prostate CTV = Prostate PTV = CTV + 0.4-1.0 cm

6.3.9 The reported doses shall include the dose to the ICRU Reference Point as well as the maximum point dose, minimum point dose, and mean dose to PTV. The ICRU Reference Points are to be located in the central part of the PTV and, secondly, on or near the central axis of the beams. Typically these points should be located on the beam axes or at the intersection of the beam axes.

6.3.10 Critical Normal Structures

The normal tissue volume to be contoured will include bladder, rectum, bilateral femora (to the level of ischial tuberosity), penile bulb, and skin. The normal tissues will be contoured and considered as solid organs. The bladder should be contoured from its base to the dome, and the rectum from the anus (at the level of the ischial tuberosities) for a length of 15 cm or to the rectosigmoid flexure. This generally is below the bottom of the sacroiliac joints. The tissue within the skin and outside all other critical normal structures and PTV's is designated as unspecified tissue. See the ATC Web site to view examples of target and normal tissue contours.

The following table summarizes the naming of organs for submission of data to the ITC

Standard Name	Description
**BLADDER**	Bladder
**CTV**	Clinical Target Volume (Prostate)
**FEMUR_LT**	Left Femur
**FEMUR_RT**	Right Femur
**GTV**	Gross Tumor Volume (Prostate)
**PENILE_BULB**	Penile Bulb
**PTV**	Planning Target Volume
**RECTUM**	Rectum

**SKIN**	External patient contour
**SEM_VES**	Seminal Vesicles

6.4 Documentation Requirements

6.4.1 The ITC will facilitate the review of GTV, CTV, PTV, and designated organs at risk (critical structures) on, as a minimum, the first five cases submitted by each institution. After an institution has demonstrated compliance with the protocol, future cases will receive ongoing remote review.

6.4.2 The institution will archive treatment prescription and verification images for later review by the study chair if requested. At least one port film or pretreatment alignment film per field along with the digital reconstructed radiographs (DRRs) from the treatment planning program or, alternatively, a simulation verification radiograph shall be acquired and kept for evaluation if requested except where geometrically impractical.

6.4.3 The ITC will display, and compare with hard copies, isodose distributions for the axial, and coronal planes (or multiple axial planes as outlined in QA Guidelines*)* through the planning target volume to verify correct digital submission and conversion.

6.4.4 The ITC will compare the submitted DVHs for the PTV, designated critical structures, and unspecified tissues with DVHs calculated by the ITC.

6.5 Compliance Criteria

6.5.1 Protocol Deviation

- No variation (total coverage); Prescription isodose surface covers ≥ 98% of the PTV and prescription isodose surface covers 100% of the CTV.
- Minor variation (marginal coverage); Prescription isodose surface coverage between ≥ 95% to < 98% of the PTV and prescription isodose surface covers 100% of the CTV.
- Major variation (miss); Prescription isodose surface coverage < 95% of the PTV or isodose covers < 100% of the CTV.

6.5.2 Dose Heterogeneity

Maximum dose to the PTV volume should not exceed the prescription dose by more than 7% (no variation: ≤ 7%; minor variation: > 7 to ≤ 10%; major variation: > 10%). The maximum point dose to critical normal structures outside the PTV including the unspecified tissue should not exceed the prescription dose. The treating physician must carefully consider the tolerance dose/volume to each critical normal structure and unspecified tissue.

6.6 R.T. Quality Assurance Reviews

The ITC will facilitate the review of GTV, CTV, PTV and designated organs at risk on the first five cases submitted by each institution (unless previously submitted on RTOG 94-06). After an institution has demonstrated compliance with the protocol, future cases will be randomly selected for review. These reviews will be ongoing and performed remotely. The final cases will be reviewed within 3 months after this study has reached the target accrual or as soon as complete data for all cases enrolled have been received, whichever occurs first.

6.7 Radiation Toxicity

6.7.1 All patients will be seen weekly by their radiation oncologist during radiation therapy. Any observations regarding radiation reactions will be recorded and should include attention toward the following potential side effects:

6.7.1.1 Small bowel or rectal irritation manifesting as abdominal cramping, diarrhea, rectal urgency, proctitis, or hematochezia

6.7.1.2 Bladder complications including urinary frequency/urgency, dysuria, hematuria, urinary tract infection, and incontinence

6.7.1.3 Radiation dermatitis

6.7.2 Clinical discretion may be exercised to treat side effects from radiation therapy. Rectal side effects such as diarrhea may be treated with diphenoxylate or loperamide. Bladder or rectal spasms can be treated with anticholinergic agents or tolterodine. Bladder irritation can be managed with phenazopyridine. Erectile dysfunction can be treated with phosphodiesterase (PDE) inhibitors (sildenafil).

6.8 (7/9/09) Radiation Therapy Adverse Event Reporting

6.8.1 Adverse Events (AEs) and Serious Adverse Events (SAEs) Reporting Requirements (4/18/06)(7/9/09)

Adverse events (AEs) and serious adverse events (SAEs) will be reported to the Cancer Therapy Evaluation Program (CTEP) via the Adverse Event Expedited Reporting System

(AdEERS) application AND to the Radiation Therapy Oncology Group (RTOG) as directed in this section.

Definition of an AE: Any unfavorable and unintended sign (including an abnormal laboratory finding), symptom, or disease temporally associated with the use of a medical treatment or procedure regardless of whether it is considered related to the medical treatment or procedure (attribution of unrelated, unlikely, possible, probable, or definite). [CTEP, NCI Guidelines: Adverse Event Reporting Requirements. January 2005; http://ctep.cancer.gov/reporting/adeers.html]

Definition of an SAE: Any adverse experience occurring at any dose that results in any of the following outcomes:

- Death;
- A life-threatening adverse experience;
- Inpatient hospitalization or prolongation of existing hospitalization;
- A persistent or significant disability/incapacity;
- A congenital anomaly/birth defect.

Important medical events that do not result in death, are not life threatening, or do not require hospitalization may be considered an SAE experience, when, based upon medical judgment, they may jeopardize the patient and may require medical or surgical intervention to prevent one of the outcomes listed in the definition. Any pregnancy occurring on study must be reported via AdEERS as a medically significant event.

SAEs (more than 30 days after last treatment) attributed to the protocol treatment (possible, probable, or definite) should be reported via AdEERS.

Note: All deaths on study require both routine and expedited reporting regardless of causality. Attribution to treatment or other cause must be provided. "On study" is defined as during or within 30 days of completing protocol treatment.

AdEERS REPORTING REQUIREMENTS

AdEERS provides a radiation therapy (RT)-only pathway for events experienced involving RT only, both with and without a drug component arm. Events that occur on the RT-only arm of a study with a drug component must be reported for purposes of comparison. Events that occur on an RT-only study without a drug component also must be reported. Events involving RT-only must be reported via the AdEERS RT-only pathway.

This study will utilize the Common Terminology Criteria for Adverse Events (CTCAE) version 3.0, MedDRA version 6.0 for grading of all adverse events. A copy of the CTCAE v3.0 can be downloaded from the Cancer Therapy Evaluation Program (CTEP) home page (http://ctep.info.nih.gov) or the RTOG web site (http://www.rtog.org/members/toxicity/main.html).

Adverse Events (AEs) and Serious Adverse Events (SAEs) that meet the criteria defined above experienced by patients accrued to this protocol must be reported to CTEP as indicated in the following tables using the AdEERS application. AdEERS can be accessed via the CTEP web site (https://webapps.ctep.nci.nih.gov/openapps/plsql/gadeers_main$.startup). Use the patient's case number without any leading zeros as the patient ID when reporting via AdEERS. In order to ensure consistent data capture, AEs and SAEs reported using AdEERS **must also be reported to RTOG on the AE case report form (see Section 12.1).** In addition, sites must submit CRFs in a timely manner after AdEERS submissions.

Certain SAEs as outlined below will require the use of the 24 Hour AdEERS Notification:

- **Phase II & III Studies: All unexpected potentially related SAEs**
- **Phase I Studies: All unexpected hospitalizations and all grade 4 and 5 SAEs regardless of relationship**

Any event that meets the above outlined criteria for an SAE but is assessed by the AdEERS System as "expedited reporting NOT required" must still be reported for safety reasons. Sites must bypass the "NOT Required" assessment and complete and submit the report. The AdEERS System allows submission of all reports regardless of the results of the assessment.

CRITERIA FOR AdEERS REPORTING REQUIREMENTS FOR ADVERSE EVENTS AND SERIOUS ADVERSE EVENTS THAT OCCUR WITHIN 30 DAYS OF THE DATE OF THE LAST PROTOCOL TREATMENT

	**3**		**3**		**4 & 5**	**4 & 5**
	**Unexpected**		**Expected**		**Unexpected**	**Expected**
	**With Hospitalization**	**Without Hospitalization**	**With Hospitalization**	**Without Hospitalization**		
**Unrelated Unlikely**	10 Calendar Days	Not Required	10 Calendar Days	Not Required	10 Calendar Days	10 Calendar Days
**Possible Probable Definite**	10 Calendar Days	10 Calendar Days	10 Calendar Days	Not Required	24 Hour: 5 Calendar Days	10 Calendar Days

CRITERIA FOR AdEERS REPORTING REQUIREMENTS FOR ADVERSE EVENTS AND SERIOUS ADVERSE EVENTS THAT OCCUR > 30 DAYS AFTER THE DATE OF THE LAST PROTOCOL TREATMENT

	**3**		**3**		**4 & 5**	**4 & 5**
	**Unexpected**		**Expected**		**Unexpected**	**Expected**
	**With Hospitalization**	**Without Hospitalization**	**With Hospitalization**	**Without Hospitalization**		
**Unrelated Unlikely**	Not required	Not required	Not required	Not Required	Not required	Not required
**Possible Probable Definite**	10 Calendar Days	Not required	Not required	Not Required	24 Hour: 5 Calendar Days	10 Calendar Days

- Expedited AE reporting timelines defined:
  - "24 hours; 5 calendar days" – The investigator must initially report the AE via AdEERS within <u>24 hours</u> of learning of the event followed by a complete AdEERS report within <u>5 calendar days</u> of the initial 24-hour report.
  - "10 calendar days" - A complete AdEERS report on the AE must be submitted within <u>10 calendar days</u> of the investigator learning of the event.
- Any medical event equivalent to CTCAE grade 3, 4, or 5 that precipitates hospitalization (or prolongation of existing hospitalization) must be reported regardless of attribution and designation as expected or unexpected with the exception of any events identified as protocol-specific expedited adverse event reporting exclusions.
- Any event that results in persistent or significant disabilities/incapacities, congenital anomalies, or birth defects must be reported via AdEERS if the event occurs following protocol treatment or procedure.
- Use the NCI protocol number and the protocol-specific patient ID assigned during trial registration on all reports.

<u>RTOG REPORTING REQUIREMENTS</u> (9/20/07)
AdEERS provides a radiation therapy (RT)-only pathway for events experienced involving RT only, both with and without a drug component arm. Events that occur on the RT-only arm of a study with a drug component must be reported for purposes of comparison. Events that occur on an RT-only study without a drug component also must be reported. Events involving RT-only must be reported via the AdEERS RT-only pathway.

This study will utilize the Common Terminology Criteria for Adverse Events (CTCAE) version 3.0 for grading of all adverse events. A copy of the CTCAE v3.0 can be downloaded from the Cancer Therapy Evaluation Program (CTEP) home page (http://ctep.info.nih.gov) or the RTOG web site (http://www.rtog.org/members/toxicity/main.html).

Adverse Events (AEs) and Serious Adverse Events (SAEs) that meet the criteria defined above experienced by patients accrued to this protocol must be reported via AdEERS. SAEs must be reported within 24 hours of discovery of the event. Contact the CTEP Help Desk if assistance is required.

All supporting source documentation being faxed to NCI, must be properly labeled with the RTOG study/case numbers and the date of the adverse event and must be faxed to the RTOG dedicated AE/SAE FAX, 215-717-0990, before the 5- or 10-calendar-day deadline. All forms submitted to RTOG Headquarters also must include the RTOG study/ case numbers; non-RTOG intergroup study and case numbers must be included, when applicable. AdEERS Reports are forwarded to RTOG electronically via the AdEERS system. Use the patient's case number as the patient ID when reporting via AdEERS.

Any late death (more than 30 days after last treatment) attributed to the protocol treatment (possible, probable or definite) should be reported via AdEERS within 24 hours of discovery. An expedited report, if applicable, will be required within 5 or 10 calendar days.

6.8.2 Acute Myeloid Leukemia (AML) or Myelodysplastic Syndrome (MDS)

AML or MDS that is diagnosed during or subsequent to treatment in patients on NCI/CTEP-sponsored clinical trials must be reported using the **NCI/CTEP Secondary AML/MDS Report Form** available at http://ctep.cancer.gov/forms/index.html. The report must include the time from original diagnosis to development of AML/MDS, characterization such as FAB subtype, cytogenetics, etc., and protocol identification (RTOG study/case numbers). This form will take the place of a report via the AdEERS system and **must be faxed to the Investigational Drug Branch, FAX 301-230-0159, and mailed to RTOG Headquarters (address below) within 30 days of AML/MDS diagnosis.**

RTOG Headquarters
AML/MDS Report
1818 Market Street, Suite 1600
Philadelphia, PA 19103

7.0 DRUG THERAPY

Not applicable to this study

8.0 SURGERY

Not applicable to this study

9.0 OTHER THERAPY

9.1 Neoadjuvant or Adjuvant Hormone Therapy

Neoadjuvant or adjuvant hormone therapy is NOT allowed on this randomized trial. The eligibility criteria for this study were chosen to exclude those patients that benefit from the use of hormone therapy in conjunction with radiation therapy. This trial is seeking to measure the effects of two fractionation schedules of radiation therapy on cancer control and toxicity. Non-protocol use of hormone therapy prior to protocol treatment will confound the effects related to the study question.

9.2 Subsequent Disease Progression

Treatment of patients who have failed by criteria described in Sections 11.4 (Criteria for Biochemical Recurrence), 11.5 (Criteria for Local Recurrence) or 11.6 (Criteria for Nonlocal Recurrence) may receive additional medical or surgical therapies. The selection of these therapies will be left to the discretion of the treating physician. Treatments may include local salvage surgery or brachytherapy in pathologically confirmed, isolated local failures. If salvage local therapy is not available or not medically appropriate, patients with local failure may be observed or treated with salvage hormone therapy (LHRH agonists, LHRH antagonists, castration, anti-androgens, or combinations of these) or other systemic treatments

(chemotherapy, other new agents). Patients with biochemical relapse or other nonlocal failures may be observed or treated with salvage hormone therapy or other systemic treatments.

10.0 TISSUE/SPECIMEN SUBMISSION (See Section 10.3.4 for a summary table) (9/20/07)(7/9/09)

Patients must be offered the opportunity to participate in the correlative components of the study, such as tissue/specimen submission. If the patient consents to participate in the tissue/specimen component of the study, the site is required to submit the patient's specimens as specified in Section 10.0 of the protocol. **Note**: Sites are not permitted to delete the tissue/specimen component from the protocol or from the sample consent.

10.1 General Information (7/9/09)

The RTOG Biospecimen Resource at the University of California San Francisco acquires and maintains high quality specimens from RTOG trials. Tissue from each block is preserved through careful block storage and processing. The RTOG encourages participants in protocol studies to consent to the banking of their tissue. In this study, tissue will be submitted to the RTOG Biospecimen Resource for the purpose of tissue banking. Translational research studies integrate the newest research findings into current protocols to investigate important biologic questions. The RTOG Biospecimen Resource also collects tissue for central review of pathology. Central review of tissue can be for eligibility and/or analysis.

In this study, tissue will be submitted to the RTOG Biospecimen Resource for the purpose of central review of pathology (required) and tissue banking for biomarker studies (optional but blocks or cores are strongly encouraged as outlined below).

10.2 Specimen Collection for Central Pathology Review (required)

The following materials must be supplied for central pathology review:

10.2.1 One H&E stained slide

10.2.2 A Pathology Report documenting that the submitted tissue specimen contains tumor. The report must include the RTOG protocol number and patient's case number. The patient's name and/or other identifying information should be removed from the report. The surgical pathology numbers and information must NOT be removed from the report.

10.2.3 A Specimen Transmittal Form clearly stating that tissue is being submitted for the RTOG Biospecimen Resource. The form must include the RTOG protocol number and patient's case number.

10.3 Specimen Collection for Tissue Banking for Biomarker Studies (optional but (1) blocks or cores and (2) serum, plasma, and buffy coat cells are strongly encouraged as outlined below) (9/20/07)

For patients who have consented to the tissue component of this study (See "About Using Tissue for Research" portion of Appendix I)

10.3.1 Biomarker studies are being done on all RTOG prostatic cancer protocols using the original diagnostic material. The emphasis has been on proliferation markers (e.g., Ki67), apoptotic pathway markers (e.g., p53, bcl-2, bax), and angiogenesis markers (e.g., COX-2, VEGF) [See Section 1.8]. These markers have shown promise in predicting prostate cancer patient outcome after radiotherapy. A final decision on which markers will be studied awaits the results of completed RTOG prostate cancer trials that have reached maturity (e.g., 86-10, 92-02, 94-13). The trial described here will not be ready for biomarker analysis for several years. The goal is to measure approximately 5-10 biomarkers using the archived pathologic material. Because genomic DNA for SNP analysis can be most effectively isolated from buffy coat leukocytes, these specimens will also be banked.

10.3.2 Sites may submit the following specimens: **(7/9/09)**

10.3.2.1 OPTIONAL BUT STRONGLY ENCOURAGED:

- A paraffin-embedded tissue block of the tumor (preferred); if the block cannot be obtained, the tissue to be submitted is dependent on specimen type as follows:
  - **If the specimen is a prostate needle core biopsy and the block cannot be obtained**, then 10-15 unstained slides (please used charged or "Plus" slides) from the block should be submitted;
  - **If the specimen is a TURP and the block cannot be obtained**, then either a 2-mm diameter core of tumor tissue, punched from the tissue block containing the tumor using a skin punch (preferred) and submitted in a plastic tube labeled with the surgical pathology number (see Appendix IV for punch kit instructions) OR 10-15

unstained slides (please use charged or "Plus" slides) from the block should be submitted.

- Tissue block, punch, or unstained slides must be clearly labeled with the pathology identification number that corresponds to the Pathology Report.

10.3.2.2 OPTIONAL BUT STRONGLY ENCOURAGED: Serum, plasma, and buffy coat cells (see Appendix V)

10.3.2.3 OPTIONAL: Fresh, frozen tissue (see Appendix IV)

10.3.3 Specimens submitted for tissue banking must be accompanied by the following: **(7/9/09)**

10.3.3.1 **For tissue blocks or fresh, frozen tissue** (see Appendix IV for the tissue collection kit and instructions)**:**

- A Pathology Report documenting that the submitted paraffin tissue block specimen contains tumor. The report must include the RTOG protocol number and patient's case number. The patient's name and/or other identifying information should be removed from the report. The surgical pathology numbers and information must NOT be removed from the report.
- A Specimen Transmittal Form clearly stating that tissue is being submitted for the RTOG Biospecimen Resource; if for translational research, this should be stated on the form. The form must include the RTOG protocol number and patient's case number.

10.3.3.2 **For serum, plasma, or buffy coat cells** (see Appendix V for the blood collection kits and instructions): **(7/9/09)**

- A Specimen Transmittal Form documenting the date of collection of the serum, the RTOG protocol number, the patient's case number, and method of storage (for example, stored at -80° C).

10.3.4 Specimen Collection Summary (9/20/07) (7/9/09)

**Specimens Strongly Encouraged for Submission**			
**Specimens taken from patient:**	**Specimens collected when:**	**Submitted as:**	**Shipped:**
A paraffin-embedded tissue block (preferred) of the primary tumor taken before initiation of treatment<br>Or if needle core biopsy: 10-15 unstained slides<br>Or if TURP: a 2 mm diameter core of tissue, punched from the tissue block with a skin punch or 10-15 unstained slides	Pretreatment	Paraffin-embedded tissue block or punch biopsy or unstained slides	Block or punch or unstained slides sent ambient
5-10 mL of whole blood (red-top) centrifuge for serum	Pretreatment	Serum samples into four (4) 1 mL cryovials	Serum sent frozen on dry ice via overnight carrier
5-10 mL of anticoagulated blood (EDTA) centrifuge for plasma	Pretreatment	Plasma samples into three (3) 1 mL cryovials	Plasma sent frozen on dry ice via overnight carrier
5-10 mL of anticoagulated blood (EDTA) centrifuge for buffy coat	Pretreatment	Buffy coat samples into three (3) 1 mL cryovials	Buffy coat sent frozen on dry ice via overnight carrier
5-10 mL of anticoagulated blood (EDTA) centrifuge for buffy coat	Mid RT treatment*	Buffy coat samples into three (3) 1 mL cryovials	Buffy coat sent frozen on dry ice via overnight carrier
5-10 mL of anticoagulated blood (EDTA) centrifuge for buffy coat	**Next follow-up treatment visit	Buffy coat samples into three (3) 1 mL cryovials	Buffy coat sent frozen on dry ice via overnight carrier

*During Week 4 for Arm 1 patients; during Week 3 for Arm 2 patients.

**For patients enrolled on study prior to Amendment 2 (previous protocol Version Date January 24, 2007) and who have signed the consent form for blood banking.

Optional Specimens for Submission (7/9/09)			
**Specimens taken from patient:**	**Specimens collected when:**	**Submitted as:**	**Shipped:**
A 5 mm^3 surgical sample from tumor	Pretreatment	1 sample of fresh, flash frozen tumor taken at surgery	Fresh tissue sent frozen on dry ice via overnight carrier

10.4 **(4/18/06) (9/20/07)(7/9/09)** Submit materials for central review and tissue banking to:

U. S. Postal Service Mailing Address: <u>For Non-frozen Specimens Only</u>
RTOG Biospecimen Resource
University of California San Francisco
Campus Box 1800
1657 Scott Street, Room 223
San Francisco, CA 94143-1800

Courier Address (FedEx, UPS, etc.): <u>For Frozen Specimens</u>
RTOG Biospecimen Resource
University of California San Francisco
1657 Scott Street, Room 223
San Francisco, CA 94115

Questions: 415-476-RTOG (7864)/FAX 415-476-5271; RTOG@ucsf.edu

10.5 Reimbursement

10.5.1 Only as specimens are requested by the protocol, or as defined for tissue banking, RTOG will reimburse submitting institutions $300 per case for fresh or flash frozen tissue or buffy coat specimens; $200 per case for a block or core of material; and $100 per case for serum or plasma. After confirmation from the RTOG Biospecimen Resource that appropriate materials have been received, RTOG Administration will prepare the proper paperwork and send a check to the institution. Pathology payment cycles are run twice a year in January and July and will appear on the institution's summary report with the institution's regular case reimbursement.

10.6 Confidentiality/Storage

(See RTOG Patient Tissue Consent Frequently Asked Questions http://www.rtog.org/tissuebank/tissuefaq.html for further details*)*

10.6.1 Upon receipt, the specimen is labeled with the RTOG protocol number and the patient's case number only. The RTOG Biospecimen Resource database only includes the following information: the number of specimens received, the date the specimens were received, documentation of material sent to a qualified investigator, type of material sent, and the date the specimens were sent to the investigator. No clinical information is kept in the database.

10.6.2 Specimens for tissue banking will be stored for an indefinite period of time. If at any time the patient withdraws consent to store and use specimens, the material will be returned to the institution that submitted it.

11.0 PATIENT ASSESSMENTS

11.1 Study Parameters (7/9/09)

**Assessments**	**Pre-Entry**	**Weekly During RT**	**Follow-Up (months)**							
			**3**	**6**	**9**	**12**	**15**	**18**	**21**	**24**
History, physical exam	X	X	X	X	X	X	X	X	X	X[c]
Zubrod performance status	X	X	X	X	X	X	X	X	X	X[c]
Prostate biopsy with Gleason score	X									X[e]
PSA	X[a]		X	X	X	X	X	X	X	X[c]
Digital rectal examination	X		X	X	X	X	X	X	X	X[c]
Urethrogram	X[b]									
Toxicity evaluation		X	X	X	X	X	X	X	X	X[c]
EPIC Questionnaire[h]	X			X		X				X[d]
EQ5D Questionnaire[h]	X			X		X				X[d]
HSCL Questionnaire[h]	X			X		X				X[d]
Utilization of Sexual Medications/Devices[h]	X			X		X				X[d]
Bone scan										X[f]
Testosterone	X[g]									
Alkaline phosphatase	X[g]									

a. PSA must be done within 180 days prior to randomization and prior to prostate biopsy or at least 10 days after prostate biopsy
b. Strongly encouraged at the time of simulation or CT scan for treatment planning
c. Follow-up will continue every 6 months for the next 3 years, then annually thereafter
d. Repeat at year 5 visit
e. Per Section 11.3.4
f. Per Section 11.3.3
g. Strongly encouraged
h. For patients who consent to this component of the study

11.2 Evaluation During Treatment

11.2.1 Patients will be seen and evaluated at least weekly during radiation therapy with documentation of tolerance, including acute reactions

11.3 Evaluation Following Treatment

11.3.1 At each visit (See Section 11.1) the patient will have an interval history, complete physical examination (including digital rectal examination) and assessment of specific GU and GI toxicity

11.3.2 **(7/9/09)** PSA will be drawn at each follow-up visit: every 3 months in years 1 & 2; then every 6 months in years 3, 4, and 5; then annually. The type of PSA assay (e.g., Abbott) should be recorded on the data forms.

11.3.3 A bone scan will be performed as clinically indicated: e.g., if the patient develops a PSA recurrence with a rapid doubling time (< 6 months) or if the patient develops symptoms suggesting the presence of metastatic disease

11.3.4 A needle biopsy is encouraged — from the site of original tumor within the prostate and/or other site of original tumor identified by the transrectal ultrasound, as indicated for rising PSA or clinical failure (see Sections 11.5.1 and 11.6.1)

11.4 Criteria for Biochemical Recurrence

11.4.1 Biochemical (PSA) recurrence is defined according to the proposed new Radiation Therapy Oncology Group/American Society for Therapeutic Radiology and Oncology (RTOG-ASTRO) criteria also known as the RTOG Phoenix definition: an increase of the PSA level at least 2 ng/mL greater than the minimum level reached after therapy (lowest PSA+ 2 criterion) (H.Sandler, personal communication, December 2005). All PSA levels done during a follow-up interval will be recorded on the data forms.

11.5 Criteria for Local Recurrence

11.5.1 Clinical criteria for local recurrence are progression (increase in palpable abnormality) at any time, failure of regression of the palpable tumor by 2 years, and redevelopment of a palpable

abnormality after complete disappearance of previous abnormalities. Needle biopsy is recommended. The presence of palpable disease must be recorded on the data collection forms for initial and follow-up evaluations of the patient.

11.5.2 Histologic criteria for local recurrence are presence of prostatic carcinoma upon biopsy and positive biopsy of the palpably normal prostate more than 2 years after the start of treatment

11.6 Criteria for Nonlocal Recurrence

11.6.1 Distant metastasis will be documented if clinical or bone scan evidence is demonstrated. Ultrasound evaluation of the prostate with needle biopsy as indicated by the findings is recommended at the time distant metastasis is reported.

11.6.2 Regional metastasis will be documented if there is radiographic evidence (CT or MRI) of lymphadenectomy and histologic confirmation.

11.7 Other Response Parameters

11.7.1 Disease-Free Survival: Disease-free survival will be measured from the date of randomization to the date of documentation of recurrence or until the date of death. This endpoint includes all measures of disease including physical exam, PSA, bone scans, CT/MRI, and biopsies.

11.7.2 Time to Local Progression: The time to progression will be measured from the date of randomization to the date of documented local progression. Patients who have a normal exam and no evidence of having a PSA recurrence will be considered controlled locally. Patients with a residual abnormality or a PSA failure shall undergo biopsy to distinguish between local and distant failures. If their exam is normal or if they are post orchiectomy, they will be censored at the last point in time they were considered locally controlled and considered "not evaluable" for further assessment of local control.

11.7.3 Time to Distant and/or Regional Failure: The time to distant or regional failure will be measured from the date of randomization to the date of documented regional nodal recurrence or distant disease relapse. Patients with evidence of biochemical failure, but a negative prostate biopsy, will be considered as distant or regional failure only.

11.7.4 Disease-Specific Survival: Disease-specific survival duration will be measured from the date of randomization to the date of death due to prostate cancer. Causes of death may require review by the study chair or their designee. Death due to prostate cancer will be defined as:

11.7.4.1 Primary cause of death certified as due to prostate cancer

11.7.4.2 Death in association with any of the following conditions:

- Further clinical tumor progression occurring after initiation of "salvage" anti-tumor (e.g., (androgen suppression) therapy
- A rise (that exceeds 1.0 ng/mL) in the serum PSA level on at least two consecutive occasions that occurs during or after "salvage" androgen suppression therapy
- Disease progression in the absence of any anti-tumor therapy

11.7.4.3 Death from a complication of therapy, irrespective of disease status.

11.7.5 Freedom from Biochemical (PSA) Recurrence (FFBR): The time to PSA failure will be measured from the date of randomization to the date of a rise by 2 ng/mL or more above the nadir PSA. Nadir PSA is defined as the lowest PSA value after randomization and before the call date PSA. That is, the time of failure will be the date of the first PSA that is 2 ng/mL or more above the lowest prior post-randomization PSA value.

11.7.6 Overall Survival: Survival duration will be measured from the date of randomization to the date of death from any cause. A post-mortem examination will be performed whenever possible and a copy of the final post-mortem report will be sent to RTOG Headquarters.

11.8 Quality of Life (QOL) (9/20/07) (7/9/09)

Note: Patients must be offered the opportunity to participate in the correlative components of the study, such as quality of life assessment. If the patient consents to participate in the quality of life (QOL) component of the study, sites are required to administer the baseline EPIC, HSCL-25, EQ5D, and the Utilization of Sexual Medications/Devices prior to the start of protocol treatment.

11.8.1 Prostate cancer-specific HRQOL as measured by the Expanded Prostate Index Composite (EPIC): Instrument development was based on advice from an expert panel and prostate cancer patients, which led to expanding the 20-item University of California-Los Angeles Prostate Cancer Index (UCLA-PCI) to the 50-item Expanded Prostate Index Composite (EPIC). Summary and subscale scores were derived by content and factor analyses. Test-retest reliability and internal consistency were high for EPIC urinary, bowel, sexual, and hormonal domain summary scores (each $r \geq 0.80$ and Cronbach's alpha ≥ 0.82) and for most domain-specific subscales. Correlations between function and bother subscales within domains were high $r > 0.60$). Correlations between different primary domains were consistently lower, indicating that these

domains assess distinct HRQOL components. EPIC domains had weak to modest correlations with the Medical Outcomes Study 12-item Short-Form Health Survey (SF-12), indicating rationale for their concurrent use. Moderate agreement was observed between EPIC domains relevant to the Functional Assessment of Cancer Therapy Prostate module (FACT-P) and the American Urological Association Symptom Index (AUA-SI), providing criterion validity without excessive overlap.[53] EPIC is a robust prostate cancer HRQOL instrument that measures a broad spectrum of symptoms; however, to decrease patient burden we will only use the domains most pertinent to this study: urinary, bowel, and sexual. The domains were validated separately; since each domain will be used intact there is no threat to validity. This reduces patient burden from 50 to 25 items.

11.8.2 The Utilization of Sexual Medications/Devices, developed as a companion questionnaire to the EPIC,[54] (Personal communication Dr. Martin Sanda 2/25/05) will be administered to assess utilization of medications and devices for erectile dysfunction and effectiveness of such interventions. The patient-completed Utilization of Sexual Medications/Devices will be collected to provide a context for interpreting the sexual domain score of the EPIC questionnaire.

11.8.3 Anxiety and depression as measured by the Hopkins Symptom Checklist (HSCL-25): The 25-item version[55] of the Hopkins Symptom Checklist (HSCL) will be used as a baseline and follow-up measure of anxiety and depressive symptoms.[55-57] The measure is closely related to the Brief Symptom Inventory[58] and is widely used as screening instruments among cancer patients. Using a cutoff of 44 and above for caseness, Hough and colleagues[55] found that the HSCL-25 was comparable or superior to the Center for Epidemiological Studies–Depression Scale in detecting psychiatric disorder. The HSCL-25 has demonstrated reliability (Cronbach's alpha >.90) and validity across a variety of general and medical populations.[59]

11.8.4 Utility as measured by the EQ-5D: The EQ-5D is a method for obtaining valuations (utilities) of HRQOL to be used as an adjustment to survival and in the cost-utility analysis. Developed in 1987, the EQ-5D is used by investigators and the pharmaceutical industry throughout the United States, Europe, and Asia. It is one of only several measures recommended for use in cost-effectiveness analyses by the Washington Panel on Cost Effectiveness in Health and Medicine.[60] The EQ-5D has now been translated into most major languages, with the EuroQol Group closely monitoring the translation process. The EQ-5D instrument is intended to complement other forms of QOL measures, and it has been *purposefully* developed to generate a *generic* cardinal index of health, thus giving it considerable potential for future use in economic evaluation. The argument by some that a generic measure does not capture some of the disease- or treatment-specific concerns of a given study misses the point. Utilities and their use in quality adjusted survival analyses and ecomonic analyses assist to inform macro (health policy, payor) decision making, not micro (individual) decision making. The findings from the disease-specific QOL instruments and treatment-related side effect QOL instruments described above will help inform individual decision making. The role of the EQ-5D is to measure HRQOL at a macro level, in the same metric as it has been measured across numerous diseases, including cancer. This instrument gives us the ability to compare across and within diseases the "big picture" of what the experts who developed the EQ-5D considered the primary health states of interest to humans: mobility, self care, usual activities, pain/discomfort, and anxiety/depression. Further, there is no standardized measure to assess and compare disease-specific utilities across or within diseases. Unlike the EQ-5D, the actual content of standard gamble (SG) and time trade-off (TTO) methods vary widely among studies and are subject to wide variations in amount and type of information presented, message framing, and visual aids, making replication of utilities with the SG or TTO extremely difficult. Therefore, using the EQ-5D, an exploratory aim is to evaluate the utility of the treatment arms. We will assess the value added of the summary score known as a Quality Adjusted Life Year (QALY), and for this study the Quality Adjusted DFS Year, that combines benefits of DFS and decrements of QOL. If (and only if) the hypothesis is substantiated we will use the quality adjusted survival in a cost-utility analysis to assess cost-benefit and compare the results to other widely accepted cancer and non-cancer therapies (see Table below).

Example of Common Medical Interventions Ranked by Incremental Cost-Effectiveness $US / Life Year Gained

Intervention	Incremental Cost-Effectiveness ($US) [61]
Liver transplantation compared with medical management	237,000
Mammography, age < 50 yrs	232,000
Dialysis compared with medical management	50,000

Drug therapy for moderate hypertension	32,600
Mammography screening for breast cancer in patients aged 50-75 yrs	20,000-50,000
ABMT compared with salvage CT for Hodgkin's recurrent after MOPP-ABV Induction CT and standard RT on RTOG trials for Non-Small Cell	21,100
Carcinoma of the Lung	7,500 - 18,500 [62]

The EQ-5D is a two-part self-assessment questionnaire that takes approximately 5 minutes to complete. [63] The first part consists of 5 items covering 5 dimensions including: mobility, self care, usual activities, pain/discomfort, and anxiety/depression. Each dimension can be graded on 3 levels including: 1-no problems, 2-moderate problems, and 3-extreme problems. Health states are defined by the combination of the leveled responses to the 5 dimensions, generating 243 (3^5) health states to which unconsciousness and death are added.[64] The second part is a visual analogue scale (VAS) valuing current health state, measured on a 20-cm 10-point interval scale. Worst imaginable health state is scored as 0 at the bottom of the scale, and best imaginable health state is scored as 100 at the top. Both the 5-item index score and the VAS score are transformed into a utility score between 0 "Worst health state" and 1 "Best health state." Either the index score or the VAS score can be used in the quality adjusted survival analysis depending on the health state(s) of interest.[65] For this study we will plan to report both the multidimensional and the VAS utilities for comparative purposes between standardized HRQOL and current health state (but will only use the multidimensional utilities for the cost-utility analysis).

Quality-adjusted survival and freedom from progression can be defined in the same manner, by the weighted sum of different time episodes added up to a total quality-adjusted life-year or freedom from progression–year [**U**= sum of quality ($\mathbf{q_i}$) of health states **K** times the duration ($\mathbf{s_i}$) spent in each health state.[66]

$$\text{Quality-Adjusted Survival} = \sum_{i=1}^{k} q_i \, s_i$$

12.0 DATA COLLECTION

Data should be submitted to:

RTOG
1818 Market Street, Suite 1600
Philadelphia, PA 19103

Patients will be identified by initials only (first middle last); if there is no middle initial, a hyphen will be used (first-last). Last names will be identified by the first letter of the last name.

12.1 Summary of Data Submission (4/18/06)

Item	Due
Demographic Form **(A5)**	Within 2 weeks of study entry
Initial Evaluation Form **(I1)**	
Pathology Report **(P1)**	
Slides/Blocks **(P2)**	
HRQOL	
▪ EPIC **(FA)**	
▪ Utilization of Sexual Medications/Devices **(SA)**	
▪ HSCL-25 **(HP)**	
▪ EQ-5D **(QF)**	
Radiotherapy Form **(T1)** **(copy to RTOG HQ and ITC)**	Within 1 week from end of RT
Adverse Event Form **(AE)** **(if corresponding T1 indicates an**	

adverse event)

Follow-Up Form **(F1)**<br>Adverse Event Form **(AE)**<br>**(if corresponding F1 indicates an adverse event)**	3, 6, 9, 12 months in year 1; q 3 months in year 2; q 6 months x 3 years, then annually; also at progression/relapse and at death
HRQOL<br>▪ EPIC **(FA)**<br>▪ Utilization of Sexual Medications/Devices **(SA)**<br>▪ HSCL-25 **(HP)**<br>▪ EQ-5D **(QF)**	6, 12, 24 months; 5 years
Autopsy Report **(D3)**	As applicable

12.2 Summary of Dosimetry Digital Data Submission (Submit to ITC; see Section 12.2.1)

Item	Due
**Preliminary Dosimetry Information**<br>▪ Digital Data Submission Information Form **(DDSI)** **(submitted online at http://atc.wustl.edu)**<br>▪ CT data, critical normal structures, all GTV, CTV and PTV contours<br>▪ Digital beam geometry for initial and boost beam sets<br>▪ Doses for initial and boost sets of concurrently treated beams<br>▪ Digital DVH data for all required critical normal structures, GTV, CTV, and PTVs for total dose plan<br>▪ Hard copy or JPEG color isodose distributions for total dose plan as described in the QA Guidelines	Within 1 week of start of RT
**Final Dosimetry Information (1/8/08)**<br>▪ Copy of Radiotherapy Form **(T1)**<br>▪ Daily Treatment Record **(T5)** (Copy to HQ and ITC)<br>▪ Modified digital patient data as required through consultation with Image Guided Therapy QA Center	Within 1 week of RT end

12.2.1 *Digital Data Submission to ITC (4/18/06)*

Digital data submission may be accomplished using media or the Internet.

For network submission: The FTP account assigned to the submitting institution by the ITC shall be used, and e-mail identifying the data set(s) being submitted shall be sent to:

itc@castor.wustl.edu

For media submission: Please contact the ITC about acceptable media types and formats.

Hardcopies accompanying digital data should be sent by mail or Federal Express and should be addressed to:

Image-Guided Therapy Center (ITC)
ATTN: Roxana Haynes
4511 Forest Park, Suite 200
St. Louis, MO 63108
314-747-5415
FAX 314-747-5423

13.0 STATISTICAL CONSIDERATIONS (9/20/07)

13.1 Study Endpoints

13.1.1 Primary Endpoint

- Disease-free survival: Disease-free failure events include local progression, distant progression, biochemical failure defined by the RTOG Phoenix definition, and death from any cause

13.1.2 Secondary Endpoints **(7/9/09)**

- Local progression: See Section 11.5
- Disease-specific survival: See Section 11.7.4
- Freedom from biochemical recurrence (FFBR): See Section 11.7.5
- Overall survival: See Section 11.7.6
- Incidence of GU and GI acute and late toxicity: See Section 13.4.3
- Statistical modeling of genomic biomarkers
- Comparison of disease-specific HRQOL change in EPIC; the Utilization of Sexual Medications/Devices supplements the EPIC
- Assessment of anxiety and depression change using the HSCL-25
- Assessment of trade-off between disease-free survival and quality of life
- Evaluation and comparison of the cost-utility of each treatment arm using EQ-5D if the primary endpoint supports the primary hypothesis
- To collect paraffin-embedded tissue block, serum, plasma, and buffy coat cells for future translational research analyses

13.2 Sample Size

13.2.1 Stratification and Randomization

Patients will be stratified before randomization with respect to Gleason score (2-4 vs. 5-6), PSA (0-< 4 vs. 4-< 10 ng/mL), and radiation modality (3D-CRT vs. IMRT). The treatment allocation scheme described by Zelen[67] will be used because it balances patient factors other than institution. Patients will be randomized to the hypofractionated 3D-CRT/IMRT arm (70 Gy /28 fractions over 5.6 weeks) or to the conventionally fractionated 3D-CRT/IMRT arm (73.8 Gy /41 fractions over 8.2 weeks).

13.2.2 Sample Size Derivation

The sample size calculation addresses the specific primary hypothesis that the disease-free survival rate in the hypofractionated 3D-CRT/IMRT (Arm 2) will not be worse than in the conventionally fractionated 3D-CRT/IMRT (Arm 1). Patients with favorable-risk prostate cancer are eligible for this trial and are characterized by a combined Gleason Score up to 6, a PSA less than 10 ng/mL and palpable tumor stages of T1 through T2c. Kupelian[68] showed that patients treated with hypofractionated 3D-CRT/IMRT had a 5-year FFBR rate per the RTOG Phoenix definition of 88%, 5-year local and distant failure rates of 4%, and a 5-year overall survival rate of 88%. Considering the patient population in the current study and based on the results of Kupelian,[68] we estimate the 5-year disease-free survival rate for Arm 1 to be 85%, which translates to a yearly hazard rate of 0.033. The study is designed to show that hypofractionated 3D-CRT will not be worse than conventionally fractionated 3D-CRT/IMRT in 5-year disease-free survival (i.e., non-inferiority testing). The sample size is estimated based on Schoenfeld's sample size formula.[69] This formula is used to calculate the sample size when the log rank test is used. We assume that the disease-free survival function follows an exponential distribution for each arm. Accrual to the study is assumed to be uniformly distributed. The null hypothesis (H_0) of this test is that the hazard rate of Arm 2 (λ_2) is worse than the hazard rate of Arm 1 (λ_1). The alternative hypothesis (H_A) is that the hazard rate of Arm 2 is not worse than the hazard rate of Arm 1.

$$H_0:\ \delta \geq \delta_0 \quad \text{vs.} \quad H_A:\ \delta < \delta_0$$

where $\delta = -\ln(\lambda_1/\lambda_2)$ and δ_0 is a non-inferiority margin. The sub-patient population group of ACR 9509 that is similar to the patient population in this study (Gleason score ≤ 6, PSA < 10 ng/mL, and T-stage T1b-T2a) shows a 15.3% difference of 5-year disease-free survival rate. The non-inferiority margin will be less than half this difference, or < 7.65%. Based on this result, a clinically meaningful and conservative difference in the disease-free survival rate is projected to be 7%, which translates to a non-inferiority margin of $\delta_0 = 0.424$. Three interim analyses and a final analysis are planned for early rejection of both the null hypothesis and the alternative hypothesis. The efficacy testing is based on one of Lan and DeMat's alpha spending functions[70] that behaves similarly to the O'Brien-Fleming boundary.[71] The futility testing is based on the Freidlin and Korn[72] method. The number of events required adjusted for this group sequential analysis is 238, so a sample size of 960 patients will be accrued to

achieve the desired 90% statistical power and one-sided significance level of 0.025. We project a study duration of about 11 years (10.8 years), with a 4.5-year accrual period and a uniform accrual rate of 20 patients per month. Guarding against an ineligibility or lack-of-data rate of up to 10%, **the final targeted accrual for this study will be 1067 patients.**

13.3 Patient Accrual

Based on patient accrual in previous RTOG randomized prostate studies, there will be relatively few entries during the initial 6 months while institutions are obtaining IRB approval. After this initial period of negligible accrual, patient accrual is projected to be 20 cases per month. The basis for this projection is RTOG 9408, which has a similar patient population and accrued at a monthly rate of 26 cases. We anticipate that the accrual rate for this study will be less than that of RTOG 9408 due to the popularity of brachytherapy in this group of patients. We expect to complete accrual in 4.5 years. The total duration of the study is expected to be 11 (10.8) years from the time the first patient is entered to the final analysis. If the average monthly accrual rate between 12 and 18 months after activation is below 5 cases per month, the study will be re-evaluated for its feasibility. If the study is continued after 18 months with fewer than 5 cases per month and then if at 24 months after study activation the average monthly accrual between 19 and 24 months is less than 5 patients per month, the study statistician will recommend to the RTOG DMC that the study be terminated. The participation of non-RTOG institutions through CTSU is expected to follow a similar pattern as seen in RTOG.

13.4 Analysis Plan

All eligible patients randomized will be included in the comparison of treatment arms (intent-to-treat analysis).

13.4.1 Primary Endpoint

The primary endpoint, 5-year disease-free survival, is measured from the date of randomization to the date of one of the following events: local progression, distant progression, biochemical failure defined by the RTOG Phoenix definition, or death from any cause. We assume that the distribution of disease-free survival for each arm is an exponential distribution. The survival distribution of disease-free survival will be estimated by the Kaplan-Meier method.[5473] We want to show that the hazard rate of Arm 2 (λ_2) will not be worse than that of Arm 1 (λ_1) in the disease-free survival distribution (i.e., non-inferiority test). The null hypothesis (H_0) and alternative hypothesis (H_A) of this non-inferiority test are:

$$H_0:\ \delta \geq 0.424 \quad \text{vs.} \quad H_A:\ \delta < 0.424$$

where $\delta = -\ln(\lambda_1/\lambda_2)$. The sub-patient population group of ACR 9509 that is similar to the patient population in this study (Gleason score ≤ 6, PSA < 10 ng/mL and T-stage T1b-T2a) shows a 15.3% difference in 5-year disease-free survival rate. Based on this result, a clinically meaningful and conservative difference in 5-year disease-free survival rate is projected to be 7%, a rate that is less than half of the observed difference in 9509 (i.e., < 7.65%)and that translates to a non-inferiority margin 0.424. This hypothesis will be tested using a log-rank test statistic at a significance level $\alpha = 0.025$. In addition, the Cox regression model[74] will be used to compare the treatment differences; PSA, Gleason score, radiation modality, race, and age (as appropriate) will be adjusted for in this model. Both unadjusted and adjusted hazard ratios and their respective 95% confidence interval will be computed.

13.4.2 Secondary Endpoints Related to Time to Failure

We assume that the distribution of failure times of secondary endpoints related to time to failure for each arm is an exponential distribution. In a trial of local radiation therapy, disease-specific survival, local progression, and FFBR provide relevant measures of the treatment effect. However, the treatment effect on other types of failure may impact the observable measures of local failure, and other competing risks may dilute the sensitivity of local failure.[75] We will use the cause-specific hazard rate (the instantaneous rate of cause-specific failure in the presence of competing failure types as a function of time) approach to consider the competing events. Freidlin and Korn[75,76] show that the cause-specific hazard rate approach is better than other approaches (e.g., the survival distribution of the time to first failure, cumulative incidence method, etc.) in most of cases. The log-rank test[76,77] on times to the specific type of failure will be used to test secondary endpoints related to time to failure (local progression, disease-specific survival, and FFBR).

We want to show that the hazard rate of Arm 2 (λ_{L2}) will not be worse than that of Arm 1 (λ_{L1}) in the local progression survival distribution. The time of local progression is measured from the date of randomization to the date of documented local progression. The null hypothesis (H_0) and alternative hypothesis (H_A) of this non-inferiority test are:

$$H_0: \delta_L \geq 0.245 \quad \text{vs.} \quad H_A: \delta_L < 0.245$$

Where $\delta_L = -\ln(\lambda_{L1}/\lambda_{L2})$. A conservative and clinically meaningful non-inferiority margin is 0.245, which is translated from a 5% difference with an 80% local progression failure rate in Arm 1. These estimates are based on the results of ACR 9509. We will use the log-rank test[76,77] with a significance level of 0.025 at the final analysis.

We want to show that the hazard rate of Arm 2 (λ_{D2}) will not be worse than that of Arm 1 (λ_{D1}) in the distribution of time to disease-specific survival. Disease-specific survival time is measured from the date of randomization to the date to the events, as defined in Section 11.7.4. The null hypothesis (H_0) and alternative hypothesis (H_A) of this non-inferiority test are:

$$H_0: \delta_D \geq 0.720 \quad \text{vs.} \quad H_A: \delta_D < 0.720$$

where $\delta_D = -\ln(\lambda_{D1}/\lambda_{D2})$. A conservative and clinically meaningful non-inferiority margin is $\delta_{D,0}$ = 0.720, which is translated from a 5% difference with 95% disease-specific survival in Arm 1 These estimates are based on the results of RTOG 9406 and ACR 9509. We will use the log-rank test[76,77] with a significance level of 0.025 at the final analysis.

We want to show that the hazard rate of Arm 2 (λ_{L2}) will not be worse than that of Arm 1 (λ_{L1}) in the 5-year FFBR survival distribution. FFBR is measured from the date of randomization to the time of PSA is greater than the nadir + 2 ng/mL. The null hypothesis (H_0) and alternative hypothesis (H_1) of this non-inferiority test are:

$$H_0: \delta \geq 0.511 \quad \text{vs.} \quad H_1: \delta < 0.511$$

where $\delta = -\ln(\lambda_1/\lambda_2)$. A conservative and clinically meaningful non-inferiority margin is 0.511, which is translated from a 7% difference with an 88% FFBR rate in Arm 1. These estimates are based on the results of Kupelian[68] and ACR 9509. We will use the log-rank test[76,77] with a significance level of 0.025 at the final analysis.

We want to test whether the hazard rate in the overall survival distribution in Arm 2 (λ_{O2}) is no worse than that of Arm 1 (λ_{O1}). Overall survival time is measured from the date of randomization to the date of documented death due to any cause. The overall survival distribution will be estimated by the Kaplan-Meier method.[73] The null (H_0) and alternative (H_A) hypotheses are:

$$H_0: \delta_{os} \geq 0.433_0 \quad \text{vs.} \quad H_A: \delta_{os} < 0.433$$

where $\delta_{os} = -\ln(\lambda_{O1}/\lambda_{O2})$. The non-inferiority margin $\delta_{os,0}$= 0.433 , which is translated from a 5% difference in overall survival with 90% overall survival in Arm 1, will be tested. We will use the log-rank test[76,77] with a significance level of 0.025 at the final analysis.

In addition, the Cox regression model will be used to compare the treatment differences for each survival distribution of secondary endpoints that are related to time to failure. Both unadjusted and adjusted hazard ratios and the respective 95% confidence interval will be computed. PSA, Gleason score, radiation modality, race, and age (as appropriate) will be adjusted for in this analysis.

13.4.3 Incidence of GU and GI Acute and Late Adverse Events

Adverse events are scored according to CTCAE version 3.0. An acute adverse event will be defined as an adverse event occurring less than or equal to 90 days from the completion of RT. A multivariate logistic regression will be used to model the distribution of acute adverse events for each arm. Both unadjusted and adjusted odds ratios and the respective 95% confidence interval will be computed. PSA, Gleason score, radiation modality, race, and age (as appropriate) will be adjusted for in this analysis. A late adverse event will be defined as an adverse event occurring more than 90 days from the completion of RT. The time to late adverse events will be measured from the time that protocol treatment is completed (i.e., the completion of radiation) to the time of the worst late adverse event. If no such late adverse event is observed until the time of the analysis, the patient will be censored at the time of the analysis. The distribution of time to late adverse events (observed severities of adverse events

over time) will be estimated using the Kaplan-Meier method using a two-sided log-rank test with a significance level of 0.05.

A multivariate Cox regression model will be used to compare the treatment differences for time to late adverse events between the two arms. Both unadjusted and adjusted hazard ratios and the respective 95% confidence interval will be computed. PSA, Gleason score, radiation modality, race, and age (as appropriate) will be adjusted for in this analysis.

13.4.4 Statistical Modeling of Genomic and Proteomic Biomarkers

At the time of data maturity of this study, we will propose specific details of the markers to be investigated. We will address the assays that will be used and will provide a list of specific correlative aims along with appropriate statistical considerations. The following is a general guideline for the statistical consideration for this analysis.

A genomic or proteomic biomarker will be categorized into either overexpressed or underexpressed. The biomarkers have shown promise in complementing the standard clinical parameters of PSA, Gleason score, and stage in prior RTOG (or other) analyses at the time of the analysis will be considered. While these markers have been selected based on prior analyses, it is likely that some other markers and/or methods will be investigated when the proposed trial matures. The patients with genomic and proteomic biomarker will be compared with the patients without a value for that biomarker to determine if there are any differences with respect to distribution of baseline variables (Gleason score, PSA, radiation modality). The number of events needed to obtain 1- β statistical power will be calculated based on Schoenfeld.[78]

$$n_d = (Z_\beta + Z_{1-\alpha})^2/[(\log(1/\Lambda))^2 P_0 P_1]$$

Where P_i = The proportion of patients allocated to group i. i=0,1
$\Lambda = \lambda_0/\lambda_1$ (>1)
n_d = The number of events (failure)
$Z_{1-\alpha}$ = The normal for the significance level α

The favorable group denotes a group with a better survival rate and the unfavorable group denotes the adverse group. The following hypotheses are equivalent to the following hypotheses under the assumption of the exponential survival distribution with hazard rate parameter λ. λ_1 is the hazard rate of the favorable group and λ_0 is the hazard rate of the unfavorable group.

$$H_0: \lambda_1 \leq \lambda_0 \text{ vs. } H_A: \lambda_1 > \lambda_0$$

Tests will be performed to see if one group is statistically significantly better than the other in the survival functions for the primary endpoint and secondary endpoints that are related to time to failure (local progression, disease-specific survival, FFBR, and overall survival). However, the selection of the cut-off point for the determination of the value to decide favorable and unfavorable risk groups for each biomarker is not established. If the hypothesized cut-off points do not yield statistical significance, other cut-off points may be evaluated. Therefore, various cut-off points are evaluated for their statistical significance. To correct the problem from the multiple testing, the Bonferroni correction will be used. In the univariate analysis, the log-rank test will be used to test for the survival differences between the favorable and unfavorable groups. The survival functions for these groups will be estimated by the Kaplan-Meier method. The multivariate analysis will be performed using the Cox proportional hazards model for both groups. Potential covariates evaluated for the multivariate models are assigned treatment and baseline variables, such as Gleason score (2-4 vs. 5-6), PSA (0-< 4 vs. 4-< 10), and radiation modality (3D-CRT vs. IMRT). A stepwise procedure will be used to develop the base model for each outcome endpoint prior to evaluating the prognostic impact of the biomarkers. This approach will be employed to account for as much variation as possible for each outcome before it is tested. It is entirely possible that factors shown to be prognostic in other published series may not be found prognostic here.

13.4.5 Analysis for Endpoints Related to HRQOL (Collected for patients who consent to this component of the study)

We will use four instruments to measure QOL: the Expanded Prostate Cancer Index Composite (EPIC), the Hopkins Symptom Checklist (HSCL-25), the Utilization of Sexual Medications/Devices, and EQ-5D. Protocol eligible patients will be included in the QOL analysis only if they have provided baseline and at least one subsequent measurement. All QOL

instruments (EPIC, HSCL-25, the Utilization of Sexual Medications/Devices, and EQ-5D) will be collected on all cases participating in the trial.

The EPIC, HSCL-25, the Utilization of Sexual Medications/Devices, and EQ-5D will be collected at pretreatment (baseline) and at 6, 12, 24 months, and 5 years after therapy starts. Patient self-assessment of symptoms will be performed using three primary EPIC scales: urinary, bowel, and sexual symptoms. The HSCL-25 has 25 items and is scored by a four-point likert scale (1-not at all, 2-a little, 3-quite a bit, and 4-extremely). A higher score means a worse mood or depression. The Utilization of Sexual Medications/Devices is designed to assess the use of erectile aids among patients treated for prostate cancer. This instrument is used to complement the sexual symptom domain in the EPIC. The EQ-5D is a two-part self-assessment questionnaire. The first part consists of five items covering five dimensions (mobility, self care, usual activities, pain/discomfort, and anxiety/depression). Each dimension is measured by a three-point likert scale (1-no problems, 2-moderate problems and 3-extreme problems). The second part is a visual analog scale (VAS) valuing the current health state measured by a 100-point scale with a 10-point interval. (0-worst imaginable health state, 100-best imaginable health state). We will transform the five-item index score and VAS score into a utility score between 0 (Worst health state) and 1 (Best health state) for comparative purposes.

For all QOL analyses we will conduct a comparison between the two treatment arms with a significance level of 0.05 and a two-sided test. To address the non-ignorable missing data caused by censoring survival time, the data analysis will also be done with patients who have not died.

The required sample size per treatment arm when we use 1 domain is 64 with 80% statistical power and 86 with 90% statistical power, respectively, based on an effect size of 0.5 according to the EPIC website. The required sample size per treatment arm when we use 4 domains is 91 with 80% statistical power and 116 with 90% statistical power, respectively, based on an effect size of 0.5. Therefore, there will be sufficient statistical power to detect a difference of 0.5 in three domain scores of HRQOL measurements in the EPIC instrument among the treatment arms. Because the participation rate in QOL assessments will be less than 100%, the expected sample size for the QOL analysis must be adjusted according to the participation rate. The Table below shows adjusted sample sizes for a range of participation rates.

Adjusted sample size per treatment with number of different domains in EPIC

Participation rate	80% power		90% power	
Number of domains	1 domain	4 domains	1 domain	4 domains
100%	64	91	86	116
90%	72	102	96	129
80%	80	114	108	145
70%	92	130	123	166
60%	107	152	144	194

* The sample size is calculated by dividing the sample size at 100% by participation rate

To inspect the missing data mechanism, we will use at least a graphical method. A missing completely at random (MCAR) mechanism exists when missing values are randomly distributed across all observations. A missing at random (MAR) mechanism exists when values are not randomly distributed across all observations, rather than one or more sub-samples.

If the cause of missing data is MCAR, listwise deletion (complete case analysis) will be done. If the MAR assumption is supported by the data, then an imputation method such as multiple imputation will be applied to impute missing data.

If the MAR assumption is not supported by the data, then adjusting for covariates (such as the baseline QOL score) might reduce the conditional association between outcomes and missing values. If missing data patterns look similar when stratified by such covariate(s), then an analysis that adjusts for such covariate(s) will be conducted and an imputation method such as multiple imputation will be applied. If approximate conditional independence cannot be obtained with any set of covariates, then MNAR (missing not at random) must be addressed

by an explicit model for the missing data mechanism[79] and then an imputation method such as multiple imputation will be applied. All results from the imputed analysis using the multiple imputation will be compared to the complete case analysis results to assess any potential biases.

We will describe the distributions of QOL data collection patterns over all collection points in each treatment arm. Longitudinal data analysis, specifically the general linear mixed-effect model,[80] will be performed to describe the change trend of the EPIC, HSCL-25 and EQ-5D scores over time across the two treatments. The primary objective in the HRQOL analysis is to determine the QOL differences. The response will be the change of measurement from baseline for each measurement. z- test statistics will be used to test the null hypothesis that responses are the same across the two treatment arms versus the alternative hypothesis that they are different. To maintain the overall significance level for testing six HRQOL instruments, the Bonerroni-adjusted significance level is 0.05/6 = 0.0083. The model will include the baseline and stratification variables (Gleason score, PSA, and radiation modality).

To examine trade-offs between the survival time and QOL, we will combine them for each patient into two single measurements: QALY and QADFSY. If (and only if) the primary endpoint hypothesis is substantiated, we will conduct a cost-utility analysis. The cost-utility analysis will not be done until after the primary endpoint results are published. QALY and QADFSY are defined by the weighted sum of different time episodes added up to a total quality-adjusted survival time and a total quality-adjusted disease-free survival time, respectively.

These health state-based methods of quality-adjusted survival analysis are known as Q-TWiST, the quality-adjusted time without symptoms and toxicity method.

$$\text{Q-TWiST} = \sum_{i=1}^{k} q_i \, s_i$$

where q_i is the quality (the utility coefficient) of health state i , s_i is the duration spent in each health state, and k is the number of health states. We will use Glasziou's multiple health-state (Q-TWiST) models[81] to use the repeated measures of EQ-5D. Because Glasziou's method incorporates longitudinal QOL data into an analysis of quality-adjusted survival, the health-stated model must be constructed on the following assumptions:

A1) QOL is independent from treatment
A2) A health state is independent from previous states
A3) Proportionality of quality-adjusted duration and duration of the actual state of a health state

Assumption A1 can be checked by plotting QOL over time according to treatment, and the t-test can be used to compare the mean QOL scores of each treatment arm. Assumption A2 can be checked by comparing the QOL for patient groups in a given health state where the groups are defined by duration of previous health state experience using a regression model. Suitable checks for assumption A3 at minimum would be a simple plot. If data does not support these assumptions, we will use a method which uses the longitudinal QOL data directly.

The Medicare reimbursement in dollars/QALY and the Medicare reimbursement in dollars/QALDFSY will be calculated as a function of the monetary cost per relative value of each health state and its duration. Cost-utility will be analyzed at two time points: at 12 months post-therapy and at 5 years follow-up. We will use the five-item utility score in EQ-5D for the cost-utility analysis. We will use the z-test to test the hypothesis that the cost-utility in the two treatment arms is the same with significance level of 0.05. We will compare the cost-utility using the Medicare reimbursement in dollars/QALY and the Medicare reimbursement in dollars/QALDFSY between the two treatment arms after adjusting for the baseline and stratification variables.

13.4.6 Group Sequential Testing for Early Termination and Reporting of Efficacy and Futility

A group sequential test with three planned interim analyses and a final analysis will be performed. The null hypothesis (H_0) and alternative hypothesis (H_A) of the primary endpoint are:

$$H_0: \ \delta \geq 0.424 \qquad \text{vs.} \qquad H_A: \ \delta < 0.424$$

where $\delta = -\ln(\lambda_1 / \lambda_2)$. At each planned interim analysis, the p-value from the log-rank test assessing treatment efficacy or futility with respect to the primary endpoint will be compared to

the nominal significance level. Lan-DeMets's alpha-spending function[70] was chosen for the efficacy test because, in practice, the information accumulated at each time point may not be equally spaced. We chose the alpha spending function that behaves like the O'Brien-Fleming boundary.[71] The null hypothesis (H_0) of the primary endpoint is that the hazard rate of Arm 2 (λ_2) will be worse than that of Arm 1 (λ_1) in the disease-free survival distribution. At each planned interim analysis, we will test the null hypothesis (H_0) for the primary endpoint with the nominal significance level boundary presented in the Table below. If the computed p-value is less than or equal to the nominal significance level boundary (α_{n1}), then we will consider stopping the trial in favor of H_A. If we stop the trial, then we conclude that the 5-year disease-free survival of Arm 2 will not be worse than that of Arm 1 (reject the null hypothesis, H_0).

Efficacy Boundaries for the Planned Interim Analysis

Information Time	Number of Biochemical Failures	Stop and Reject H_0 if p-value $\le \alpha_{n1}$
0.25	60	< 0.0001 (-4.333)
0.5	120	0.0015 (-2.963)
0.75	179	0.0092 (-2.359)
1.0	238	0.022 (-2.0141)

* The nominal significance levels are calculated at $\delta = 0.424$
* ( ) are for the **z-scale critical boundary for each** nominal significance level.

The alternative hypothesis (H_A) of the primary endpoint is that the hazard rate of Arm 2 (λ_2) will not be worse than that of Arm 1 (λ_1) in the disease-free survival distribution. For the futility testing boundary, we will use a less aggressive boundary, Rule C in Freidlin and Korn,[72] than the power family group sequential tests. The alternative hypothesis (at $\delta = 0$) will be tested at 0.005 level at each interim analysis. If the computed p-value is less than 0.005 (the futility nominal significance level) then we will consider stopping the trial in favor of H_0. If we stop the trial, then we will conclude that that the 5-year disease-free survival of Arm 2 will be worse than that of Arm 1 (not reject H_o). Otherwise, we will continue the trial.

The responsible statistician will recommend to the RTOG DMC that the randomization be discontinued, if applicable, and the study be considered for early publication. Before making such a recommendation, the accrual rate, treatment compliance, safety of the treatments, and the importance of the study are taken into consideration along with the p-value. The RTOG DMC will then make a recommendation about the trial to the RTOG group chair.

13.4.7 Stopping Rules for Excessive Adverse Events

Based on our experience in RTOG 9406, we estimated approximately ≤ 5% of the men experienced a grade 3^+ adverse event. For this study, a rate of 5% grade 3^+ GU and GI adverse events (p_t) according to the CTCAE version 3.0 within 24 months of the start of radiation therapy is considered acceptable for each arm. A rate of 20% is considered unacceptable. The null hypothesis (H_0) is that this radiation therapy is not tolerable versus the alternative hypothesis (H_A) that this radiation therapy is tolerable. The following hypothesis will be tested using Fleming's Multiple Testing Procedure,[82] with a significance level of 0.035 and 90% statistical power.

$$H_0: p_t \ge 0.2 \text{ vs. } H_A: p_t \le 0.05$$

We are more concerned with a false negative decision (i.e., failing to detect the increase in toxicity if it exists) than we are with a false positive decision (i.e., deciding one treatment arm is more toxic, when in fact it is not). The stopping and continuation rules in the Table below will be applied in three stages to the first analyzable 45 cases randomized to each arm who received at least some treatment. Analyzable patients are defined as eligible patients who received at least some treatment. If at any stage, we reject the null hypothesis and show that the grade 3^+ GU and GI adverse event rate may not be greater than or equal to 20%, we would conclude that this treatment regimen is "tolerable" and continue accrual to the study. If we reject the alternative hypothesis at any stage, claiming that the grade 3^+ GU and GI adverse event rate may be not be less than or equal to 5%, we would temporarily close the study to accrual, gather the relevant source data on the cases with grade 3^+ GU and GI adverse events, prepare a statistical report summarizing the adverse event findings, and present the report to the radiation and medical oncology study chairs for review. The study chairs will review all source documentation on the analyzed cases with adverse events and the statistical report summarizing the findings as soon as possible. Following the study chairs' review of the

data, a conference call will be scheduled with the study chairs, statistician, and RTOG group chair to discuss the findings and make a recommendation about the study. Once a recommendation is made, the responsible statistician will present the statistical report along with the recommendation to the RTOG Data Monitoring Committee (DMC) for the Committee's consideration. The RTOG DMC will then make a recommendation about the course of action and future of the study. If at the first or second stage either of the stopping rules is not met, we will continue accrual and monitoring for grade 3^+ GU and GI adverse events. If we continue until the last stage, then we will either conclude "tolerability" or not.

Stopping and Continuation Rules for Grade 3^+ GU/GI Adverse Events

Number of Analyzable Patients *	Reject H_0 $p_t \geq 0.2$ and continue	Reject $H_A : p_t \leq 0.05$ and stop
15	≤ 2	≥ 3
30	≤ 3	≥ 4
45	≤ 4	≥ 5

* Analyzable patients are defined as eligible patients who received at least some treatment.
- The second and third column contains the number of men who experience grade 3^+ GU/GI Adverse Events.

13.4.8 Interim Report to Monitor Study Progress

Interim reports with descriptive statistics will be prepared twice per year until the initial paper reporting the treatment results has been submitted. In general, the interim reports will contain information about the patient accrual rate with a projected completion date for the accrual phase; compliance rate of treatment delivery with the distributions of important prognostic baseline variables; and the frequencies and severity of the adverse event by treatment arm. The interim reports will not contain the results from the treatment comparisons with respect to the primary endpoint, disease-free survival, or secondary endpoints.

13.4.9 Analysis for Reporting Initial Treatment Results

The primary hypothesis of this study is that the hypofractionated 3D-CRT/IMRT method is no worse than the conventionally fractionated 3D-CRT/IMRT method for 5-year disease-free survival. The final analysis reporting the treatment results will be carried out after 238 disease-free events have been observed unless the criteria for early stopping are met. The disease-free survival difference between the control arm and the experimental arm will be tested using the log-rank statistic[76,77] at a significance level of 0.025 given that the three interim analyses are carried out as described in the Section 13.4.6. The final analysis will include tabulation of all cases entered and those excluded from the analyses with the reasons for such given; the distribution of the important prognostic baseline variables; and observed results with respect to the primary and secondary endpoints. All eligible patients randomized will be included in the comparison and will be grouped by assigned treatment in the analysis (intent-to-treat analysis). In addition, exploratory analyses of treatment comparisons of local progression, disease-free survival, FFBR, and overall survival will be tested using the Cox proportional hazard model that includes age, race, clinical tumor stage, and the stratification factors (PSA, Gleason score, and radiation modality). Also, where feasible, treatment comparisons with respect to the primary endpoint (disease-free survival) and secondary endpoints (local progression, disease-free survival, FFBR, and overall survival) will be compared within each ethnic category.

13.4.10 CDUS Tracking (4/18/06)

This study will be monitored by the Clinical Data Update System (CDUS) version 3.0. Cumulative CDUS data will be submitted quarterly by electronic means. Reports are due January 31, April 30, July 31, and October 31.

13.5 Gender and Minorities (4/18/06)

In conformance with the National Institutes of Health (NIH) Revitalization Act of 1993 with regard to inclusion of women and racial/ethnic minorities in clinical research, we have also considered the possible interaction between race and treatment. Based on the accrual statistics from RTOG 9408, we project that 81% of the men in the study will be white, 15% black or African American, 3% Hispanic, 0.5% Asian, 0.3% Pacific Islander, and 0.2% American Indian or Alaskan Native. Planned gender and minorities accrual is listed below. The distribution of cases by race (black or African American vs. non-black or non-African American) and treatment arm for the recently completed RTOG prostate trials 92-02 and 94-13 is shown below. There was no statistical

evidence to support a difference in treatment outcome and race in either study. Thus, we do not expect to see any evidence of a treatment difference between the two arms in the black or African American population in the current study. We will, however, include the race variable in all regressions including the Cox models.

Planned Gender and Minority Inclusion

Ethnic Category	Sex/Gender		
	**Females**	**Males**	**Total**
Hispanic or Latino	N/A	32	32
Not Hispanic or Latino	N/A	1035	1035
**Ethnic Category: Total of all subjects**	N/A	1067	1067
Racial Category			
American Indian or Alaskan Native	N/A	2	2
Asian	N/A	5	5
Black or African American	N/A	160	160
Native Hawaiian or other Pacific Islander	N/A	3	3
White	N/A	897	897
Racial Category: Total of all subjects		1067	1067

Distribution of Race and Treatment Arm in RTOG Studies 94-13 and 92-02

Study	Treatment Arms*	Sample Size	Race	
			**African American**	**Non-African American**
**RTOG 94-13**				
**Radiation Field**	**WP RT**	**641**	**153 (24%)**	**488 (76%)**
	**PO RT**	**638**	**176 (28%)**	**462 (72%)**
**Hormone Timing**	**NHT**	**635**	**159 (25%)**	**476 (75%)**
	**AHT**	**644**	**170 (26%)**	**474 (74%)**
**RTOG 92-02**				
	**STAD**	**761**	**92 (12%)**	**669 (88%)**
	**LTAD**	**753**	**105 (14%)**	**648 (86%)**

*Treatment arms for: RTOG 94-13: WP RT = Whole Pelvis RT (Radiation Therapy)+Boost and TAS (Total Androgen Suppression); PO RT = Prostate Only RT and TAS; NHT= Neoadjuvant TAS and RT; and AHT = Adjuvant TAS and RT and RTOG 92-02: STAD = Short-term TAS (4 months) and RT; and LTAD = Long-term TAS (28 months)

REFERENCES (9/20/07)

1. Brenner DJ, Hall EJ. Fractionation and protraction for radiotherapy of prostate carcinoma. Int J Radiat Oncol Biol Phys 1999; 43:1095-1101.

2. Zietman A, Moughan J, Owen J, Hanks G. The Patterns of Care Survey of radiation therapy in localized prostate cancer: similarities between the practice nationally and in minority-rich areas. Int J Radiat Oncol Biol Phys 2001; 50:75-80.

3. Zelefsky MJ, Fuks Z, Hunt M, et al. High-dose intensity modulated radiation therapy for prostate cancer: early toxicity and biochemical outcome in 772 patients. Int J Radiat Oncol Biol Phys 2002; 53:1111-1116.

4. Fowler JF, Ritter MA, Chappell RJ, Brenner DJ. What hypofractionated protocols should be tested for prostate cancer? Int J Radiat Oncol Biol Phys 2003; 56:1093-1104.

5. Brenner DJ, Martinez AA, Edmundson GK, et al. Direct evidence that prostate tumors show high sensitivity to fractionation (low alpha/beta ratio), similar to late-responding normal tissue. Int J Radiat Oncol Biol Phys 2002; 52:6-13.

6. D'Souza WD, Thames HD. Is the alpha/beta ratio for prostate cancer low? Int J Radiat Oncol Biol Phys 2001; 51:1-3.

7. King CR, Fowler JF. A simple analytic derivation suggests that prostate cancer alpha/beta ratio is low. Int J Radiat Oncol Biol Phys 2001; 51:213-214.

8. Kupelian PA, Thakkar W, Khuntia D, et al. Hypofractionated intensity-modulated radiotherapy (70 gy at 2.5 gy per fraction) for localized prostate cancer: Long term outcomes. Int J Radiat Oncol Biol Phys 2005; Sep 15 [Epub ahead of print]

9. Consensus statement: guidelines for PSA following radiation therapy. American Society for Therapeutic Radiology and Oncology Consensus Panel. Int J Radiat Oncol Biol Phys 1997; 37:1035-1041.

10. Yeoh EE, Fraser RJ, McGowan RE, et al. Evidence for efficacy without increased toxicity of hypofractionated radiotherapy for prostate carcinoma: early results of a phase III randomized trial. Int J Radiat Oncol Biol Phys 2003; 55:943-955.

11. Lukka H, Hayter C, Julian JA, et al. Randomized trial comparing two fractionation schedules for patients with localized prostate cancer. J Clin Oncol 2005; 23:6132-6138.

12. Pollack A, Hanlon A, Horwitz EM, et al. Radiation therapy dose escalation for prostate cancer: a rationale for IMRT. World J Urol 2003; 21:200-208.

13. Pollack A, Zagars GK, Starkschall G, et al. Prostate cancer radiation dose response: results of the M. D. Anderson phase III randomized trial. Int J Radiat Oncol Biol Phys 2002; 53:1097-1105.

14. Pollack A, Cowen D, Troncoso P, et al. Molecular markers of outcome after radiotherapy in patients with prostate carcinoma: Ki-67, bcl-2, bax, and bcl-x. Cancer 2003; 97:1630-1638.

15. Grignon DJ, Caplan R, Sarkar FH, et al. p53 status and prognosis of locally advanced prostatic adenocarcinoma: a study based on RTOG 8610. J Natl Cancer Inst 1997; 89:158-170.

16. Leadon SA Repair of DNA damage produced by ionizing radiation: a minireview. Semin. Radiat. Oncol. 6, 295-305, 1996.

17. Haimovitz-Friedman A Radiation-induced signal transduction and stress response. Radiat. Res. 150, S102, 1998.

18. Murray D, Begg AD. DNA repair genes and radiosensitivity. In: LC Panasci and MA Alaoui-Jamali (eds.), DNA Repair in Cancer Therapy, Humana Press, Totawa, NJ, pp. 211-256, 2004.

19. Green H et al. Variation in the manganese superoxide dismutase gene (SOD2) is not a major cause of radiotherapy complications in breast cancer patients. Radiother Oncol 63, 213-216, 2002.
20. Severin DM et al. Novel DNA sequence variants in the hHR21 DNA repair gene in radiosensitive cancer patients. Int. J. Radiat. Oncol. Biol. Phys. 50, 1323-1331, 2001.

21. Brookes AJ. The essence of SNPs. Gene, 234, 177-186, 1999.

22. Stoneking M. Single nucleotide polymorphisms. From the evolutionary past. Nature 409, 821-822, 2001.

23. Damaraju S et al. Genomic approaches to clinical drug resistance. In: B. Anderson and D. Murray, (eds.) Clinically Relevant Resistance in Cancer Chemotherapy. pp.347-372, 2002.

24. Riley JH et al. The use of single nucleotide polymorphisms in the isolation of common disease genes. Pharmacogenomics 1, 39-47, 2000.

25. Quarmby S, et al. Association of transforming growth factor beta-1 single nucleotide polymorphisms with radiation-induced damage to normal tissues in breast cancer patients. Int J Radiat Biol. 79(2):137-43, 2003.

26. Andreassen CN et al. Prediction of normal tissue radiosensitivity from polymorphisms in candidate genes. Radiother Oncol. 69(2):127-35, 2003.

27. Andreassen CN et al. TGFβ1 polymorphisms are associated with risk of late normal tissue complications in the breast after radiotherapy for early breast cancer. Radiother Oncol. 75:18-21, 2005.

28. Cesaretti JA, Stock RG, Lehrer S, Atencio DA, Bernstein JL, Stone NN, Wallenstein S, Green S, Loeb K, Kollmeier M, Smith M, Rosenstein BS. ATM sequence variants are predictive of adverse radiotherapy response among patients treated for prostate cancer. Int J Radiat Oncol Biol Phys. 2005 Jan 1;61(1):196-202.

29. Andreassen CN, Alsner J, Overgaard J, Herskind C, Haviland J, Owen R, Homewood J, Bliss J, Yarnold J. TGFB1 polymorphisms are associated with risk of late normal tissue complications in the breast after radiotherapy for early breast cancer. Radiother Oncol. 2005 Apr;75(1):18-21.

30. Andreassen CN, Overgaard J, Alsner J, Overgaard M, Herskind C, Cesaretti JA, Atencio DP, Green S, Formenti SC, Stock RG, Rosenstein BS. ATM sequence variants and risk of radiation-induced subcutaneous fibrosis after postmastectomy radiotherapy. Int J Radiat Oncol Biol Phys. 2006 Mar 1;64(3):776-83.

31. Andreassen CN, Alsner J, Overgaard M, Sorensen FB, Overgaard J. Risk of radiation-induced subcutaneous fibrosis in relation to single nucleotide polymorphisms in TGFB1, SOD2, XRCC1, XRCC3, APEX and ATM--a study based on DNA from formalin fixed paraffin embedded tissue samples. Int J Radiat Biol. 2006 Aug;82(8):577-86.

32. De Ruyck K, Van Eijkeren M, Claes K, Morthier R, De Paepe A, Vral A, De Ridder L, Thierens H. Radiation-induced damage to normal tissues after radiotherapy in patients treated for gynecologic tumors: association with single nucleotide polymorphisms in XRCC1, XRCC3, and OGG1 genes and in vitro chromosomal radiosensitivity in lymphocytes. Int J Radiat Oncol Biol Phys. 2005 Jul 15;62(4):1140-9.

33. De Ruyck K, Van Eijkeren M, Claes K, Bacher K, Vral A, De Neve W, Thierens H. TGFbeta1 polymorphisms and late clinical radiosensitivity in patients treated for gynecologic tumors. Int J Radiat Oncol Biol Phys. 2006 Jul 15;65(4):1240-8.

34. Damaraju S, Murray D, Dufour J, Carandang D, Myrehaug S, Fallone G, Field C, Greiner R, Hanson J, Cass CE, Parliament M. Association of DNA repair and steroid metabolism gene polymorphisms with clinical late toxicity in patients treated with conformal radiotherapy for prostate cancer. Clin Cancer Res. 2006 Apr 15;12(8):2545-54.

35. Holmboe ES, Concato J. Treatment decisions for localized prostate cancer: asking men what's important. J Gen Intern Med 2000; 15:694-701.

36. Parliament MB, Danjoux CE, Clayton T. Is cancer treatment toxicity accurately reported? Int J Radiat Oncol Biol Phys 1985; 11:603-608.

37. Watkins-Bruner D, Scott C, Lawton C, et al. RTOG's first quality of life study--RTOG 90-20: a phase II trial of external beam radiation with etanidazole for locally advanced prostate cancer. Int J Radiat Oncol Biol Phys 1995; 33:901-906.

38. Movsas B, Scott C, Langer C, et al. Randomized trial of amifostine in locally advanced non-small-cell lung cancer patients receiving chemotherapy and hyperfractionated radiation: radiation therapy oncology group trial 98-01. J Clin Oncol 2005; 23:2145-2154.

39. van Andel G, Kurth KH. The impact of androgen deprivation therapy on health related quality of life in asymptomatic men with lymph node positive prostate cancer. Eur Urol 2003; 44:209-214

40. Clark JA, Bokhour BG, Inui TS, Silliman RA, Talcott JA. Measuring patients' perceptions of the outcomes of treatment for early prostate cancer. Med Care 2003; 41:923-936.

41. Roth AJ, Rosenfeld B, Kornblith AB, et al. The memorial anxiety scale for prostate cancer: validation of a new scale to measure anxiety in men with prostate cancer. Cancer 2003; 97:2910-2918.

42. Wei JT, Dunn RL, Sandler HM, et al. Comprehensive comparison of health-related quality of life after contemporary therapies for localized prostate cancer. J Clin Oncol 2002; 20:557-566.

43. Smith DH, Fenn P, Drummond M. Cost effectiveness of photodynamic therapy with verteporfin for age related macular degeneration: the UK case. Br J Ophthalmol 2004; 88:1107-1112.

44. Maberley D, Walker H, Koushik A, Cruess A. Screening for diabetic retinopathy in James Bay, Ontario: a cost-effectiveness analysis. CMAJ 2003; 168:160-164.

45. Brown GC, Brown MM, Sharma S, Busbee B, Landy J. A cost-utility analysis of interventions for severe proliferative vitreoretinopathy. Am J Ophthalmol 2002; 133:365-372.

46. Kobelt G, Lundstrom M, Stenevi U. Cost-effectiveness of cataract surgery. Method to assess cost-effectiveness using registry data. J Cataract Refract Surg 2002; 28:1742-1749.

47. Jonsson L. Cost-effectiveness of memantine for moderate to severe Alzheimer's disease in Sweden. Am J Geriatr Pharmacother 2005; 3:77-86.

48. Palmer CS, Niparko JK, Wyatt JR, Rothman M, de Lissovoy GA. Prospective study of the cost-utility of the multichannel cochlear implant. Arch Otolaryngol Head Neck Surg 1999; 125:1221-1228.

49. Trippoli S, Vaiani M, Lucioni C, Messori A. Quality of life and utility in patients with non-small cell lung cancer. Quality-of-life Study Group of the Master 2 Project in Pharmacoeconomics. Pharmacoeconomics 2001; 19:855-863.

50. Essink-Bot ML, de Koning HJ, Nijs HG, et al. Short-term effects of population-based screening for prostate cancer on health-related quality of life. J Natl Cancer Inst 1998; 90:925-931.

51. Sandblom G, Carlsson P, Sennfalt K, Varenhorst E. A population-based study of pain and quality of life during the year before death in men with prostate cancer. Br J Cancer 2004; 90:1163-1168.

52. Sennfalt K, Carlsson P, Sandblom G, Varenhorst E. The estimated economic value of the welfare loss due to prostate cancer pain in a defined population. Acta Oncol 2004; 43:290-296.

53. Wei JT, Dunn RL, Litwin MS, Sandler HM, Sanda MG. Links Development and validation of the expanded prostate cancer index composite (EPIC) for comprehensive assessment of health-related quality of life in men with prostate cancer. Urology 2000; 56:899-905.

54. Miller DC, Wei JT, Dunn RL, et al. Differences in patient-reported utilization of medications or devices for erectile dysfunction among long-term prostate cancer treatment survivors. Submitted 2005.

55. Hough R, Landsverk J, Stone J, et al. Comparison of psychiatric screening questionnaires for primary care patients. Final report for NIMH Contract No. 278-81-0036 (DB). 15 June 1995; 22 (12): 913-921.

56. Hesbacher P, Rickels K, Downing RW, Stepansky P. Assessment of psychiatric illness severity by family physicians. Social Science & Medicine 1978; 12(1-A):45-47.

57. Hesbacher PT, Rickels K, Morris RJ, Newman H, Rosenfeld H. Psychiatric illness in family practice. J Clin Psychiat 1980; 41:6-10.

58. Derogatis LR, Melisaratos N. The brief symptom inventory: an introductory report. Psychol Med 1983; 13:595-605.

59. Bolton P. Cross-cultural validity and reliability testing of a standard psychiatric assessment instrument without a gold standard. J Nerv Ment Dis 2001; 189:238-242.

60. Rabin R, de Charro F. EQ-5D: a measure of health status from the EuroQol Group. Ann Med 2001; 33:337-343.

61. Hillner BE, Smith TJ. Cost-effectiveness analysis of three regimens using vinorelbine (Navelbine) for non-small cell lung cancer. Semin Oncol 1996; 23(2 Suppl 5):25-30.

62. Konski A, Scott C, Movsas B, et al. Cost-utility analysis of various treatments for non-small cell carcinoma of the lung. 2000; The Second European Conference on the Economics of Cancer, Brussels (Abstract).

63. Schulz MW, Chen J, Woo HH, et al. A comparison of techniques for eliciting patient preferences in patients with benign prostatic hyperplasia. J Urol 2002; 168:155-159.

64. Badia X, Herdman M, Kind P.The influence of ill-health experience on the valuation of health. Pharmacoeconomics 1998; 13:687-696.

65. Wu AW, Jacobson KD, Frick DL, et al. Validity and responsiveness of the EQ5D as a measure of health-related quality of life in people enrolled in an AIDS clinical trial. Qual Life Res 2002; 11:273-282.

66. Glasziou PP, Simes RJ, Gelber RD. Quality adjusted survival analysis. Stat Med 1990; 9:1256-1276.

67. Zelen M. The randomization and stratification of patients to clinical trials. J Chron Dis 1974; 27:365-375.

68. Kupelian PA, Elshaikh M, Reddy CA, et al. Comparison of the efficacy of local therapies for localized prostate cancer in the prostate-specific antigen era: a large single-institution experience with radical prostatectomy and external-beam radiotherapy. J Clin Oncol 2002; 20:3376-3385.

69. Schoenfeld D. Sample-size formula for the proportional-hazards regression model. Biometrics 1983; 39:499-503.

70. Lan KKG, DeMets ER. Discrete sequential boundaries for clinical trials. Biometrika 1983; 70:659-663.

71. O'Brien PC, Fleming TR. A multiple testing procedure for clinical trials. Biometrics 1979; 35:549-556.

72. Freidlin B, Korn EL. A comment on futility monitoring. Controlled Clinical Trials 2002; 23:355-366.

73. Kaplan EL, Meier P. Nonparameteric estimation from incomplete observations. J Amer Statist Assoc 1958; 53:457-481.

74. Cox DR. Regression models and life tables. J Royal Stat Soc 1972; (Series B) 34:187-229.

75. Freidlin B, Korn EL. Testing treatments effects in the presence of competing risks. Stat Med 2004; 24:1703-1712.

76. Mantel N. Evaluation of survival data and two new rank order statistics arising in its consideration. Cancer Chemother Rep 1966; 50:163-170.

77. Kim K, Tsiatis AA. Study duration for clinical trials with survival response and early stopping rule. Biometrics 1990; 46:81-92.

78. Schoenfeld D. The asymptotic properties of nonparametric test for comparing survival distributions. Biometrika 1981; 68:316-319.

79. Donaldson GW, Moinpour CM. Learning to live with missing quality of life data in advanced-stage disease trials. J Clin Oncol 2005; 23:1-5.

80. Verbeke G, Molenberghs G. Linear Mixed Models for Longitudinal Data. 2000; New York: Springer-Verlag, Inc.

81. Glasziou PP, Cole BF, Gelber RD, et al. Quality adjusted survival analysis with repeated quality of life measures. Stat Med 1998; 17:1215-1229.

82. Fleming T. One-sample multiple testing procedure for phase II clinical trials. Biometrics 1982; 38:143-151.

APPENDIX I (4/18/06)(9/20/07)(12/3/07)

Informed Consent Template for Cancer Treatment Trials (English Language)

RTOG 0415

A PHASE III RANDOMIZED STUDY OF HYPOFRACTIONATED 3D-CRT/IMRT VERSUS CONVENTIONALLY FRACTIONATED 3D-CRT/IMRT IN PATIENTS WITH FAVORABLE-RISK PROSTATE CANCER

This is a clinical trial, a type of research study. Your study doctor will explain the clinical trial to you. Clinical trials include only people who choose to take part. Please take your time to make your decision about taking part. You may discuss your decision with your friends and family. You can also discuss it with your health care team. If you have any questions, you can ask your study doctor for more explanation.

You are being asked to take part in this study because you have prostate cancer and your doctor has recommended external beam radiation therapy.

Why is this study being done?

One of the standard treatment options for your stage and type of prostate cancer is external beam radiation therapy. More recent radiation therapy planning methods with three-dimensional therapy or intensity modulated radiation therapy (IMRT) allow safer delivery of higher than conventional daily doses of radiation. The purpose of this study is to compare the effects (good and bad) on you and your cancer of the standard dose of radiation therapy (41 treatments over 8 weeks) with a higher daily dose (experimental) of radiation (28 treatments over 5 and a half weeks) to see if the effects of the treatments are similar.

How many people will take part in the study?

About 1067 people will take part in this study.

What will happen if I take part in this research study?

Before you begin the study … (12/3/07) (7/9/09)

You will need to have the following exams, tests or procedures to find out if you can be in the study. These exams, tests or procedures are part of regular cancer care and may be done even if you do not join the study. If you have had some of them recently, they may not need to be repeated.

- History and physical exam, including a digital rectal exam (DRE) and an assessment of your ability to carry out activities of daily living (which will include questions such as whether you are able to feed, bathe, and dress yourself)

- A biopsy of your prostate to determine your Gleason score (a value that helps determine the stage of your prostate cancer) Your doctor will send some of the tumor tissue obtained in the biopsy of your prostate to a central office. There, a pathologist will confirm your type of tumor. This tissue submission for review is required for this study.
- A blood test to determine your PSA (a value that helps determine the stage of your prostate cancer). About 2 teaspoons of blood will be drawn from a vein or, if you have one, a catheter. The study doctor may also test your testosterone and alkaline phosphatase levels.

<u>During the study …</u> (12/3/07)

If the exams, tests and procedures show that you can be in the study, and you choose to take part, then you will need the following tests and procedures. They are part of regular cancer care.

- History and physical exam, including an assessment of your ability to carry out activities of daily living *(Weekly during radiation treatment)*

You will need this assessment to see how the study is affecting your body.

- Assessment of any side effects you may be experiencing from the treatment *(Weekly during radiation treatment)*

You will be "randomized" into one of the study groups described below. Randomization means that you are put into a group by chance. Neither you nor your study doctor can choose the group you will be in. You will have an equal chance of being placed in either group.

<u>If you are in group 1 (often called "Arm A") *...*</u>
You will receive the standard daily dose of three-dimensional radiation or IMRT. You will receive radiation therapy once daily, 5 days a week, Monday through Friday, for a total of 41 treatments. Each radiation treatment will take 15-30 minutes.

<u>If you are in group 2 (often called "Arm B")…</u>
You will receive a higher daily dose of three-dimensional radiation or IMRT. You will receive radiation therapy once daily, 5 days a week, Monday through Friday, for a total of 28 treatments. Each radiation treatment will take 15-30 minutes.

<u>When you are finished receiving radiation*...*</u>
You will need these tests and procedures:

- History and physical exam, including a digital rectal exam (DRE) and an assessment of your ability to carry out activities of daily living *(Every 3 months for the first 2 years following the start of radiation, every 6 months for the next 3 years, and then annually)*
- Assessment of any side effects you may be experiencing from the treatment *(Every 3 months for the first 2 years following the start of radiation, every 6 months for the next 3 years, and then annually)*
- If your disease progresses, your study doctor may request a needle biopsy of your prostate to microscopically evaluate response to treatment

How long will I be in the study?

You will receive radiation treatments for either 5 and a half or 8 weeks. After you are finished receiving radiation, the study doctor will ask you to visit the office for follow-up exams every 3 months for the first 2 years following the start of radiation, then every 6 months for the next 3 years. After that, the study doctors would like to keep track of your medical condition indefinitely by seeing you for follow-up exams every year.

Can I stop being in the study? (12/3/07)

Yes. You can decide to stop at any time. Tell the study doctor if you are thinking about stopping or decide to stop. He or she will tell you how to stop safely.

It is important to tell the study doctor if you are thinking about stopping so any risks from the radiation can be evaluated by him/her. Another reason to tell your study doctor that you are thinking about stopping is to discuss what follow-up care and testing could be most helpful for you.

The study doctor may decide to take you off this study at any time if he/she believes it is in your best interest; if you do not follow the study rules; or if the study is stopped.

What side effects or risks can I expect from being in the study?

You may have side effects while on the study. Everyone taking part in the study will be watched carefully for any side effects. However, researchers don't know all the side effects that may happen. Side effects may be mild or very serious. Your health care team may give you medicines to help lessen side effects. Many side effects go away soon after you stop taking the radiation. In some cases, side effects can be serious, long lasting, or may never go away. In addition, some of the side effects may be life threatening and, in rare instances, may cause death.

You should talk to your study doctor about any side effects that you have while taking part in the study.

Risks and side effects related to the radiation include those which are:

Likely

- Tanning, redness, or darkening of skin in treatment area
- Rash, itching or peeling of skin
- Temporary hair loss in the treatment area
- Temporary fatigue, nausea or diarrhea
- Abdominal cramps
- Bladder irritation with a stinging sensation
- Frequency or urgency of urination
- Rectal irritation with more frequent bowel movements
- Mild rectal bleeding that does not require treatment

Less Likely

- Urinary obstruction requiring the placement of a temporary urinary catheter

Rare but Serious (7/9/09)

- Injury to the bladder, urethra, bowel, or other tissues in the pelvis or abdomen
- Intestinal obstruction
- Inability to achieve an erection (inability of the penis to become hard)
- Rectal bleeding that requires medication or surgery to stop

Reproductive Risks

You should not father a baby while on this study because the radiation can affect an unborn baby. It is important you understand that you need to use birth control while on this study. Check with your study doctor about what kind of birth control methods to use and how long to use them. Some methods might not be approved for use in this study.

For more information about risks and side effects, ask your study doctor.

Are there benefits to taking part in the study?

Taking part in this study may or may not make your health better. It is not known whether the higher daily dose of three-dimensional radiation therapy or IMRT is equivalent to the standard daily dose. We do know that the information from this study will help researchers learn more about these different doses as a treatment for prostate cancer. This information could help future patients with prostate cancer.

What other choices do I have if I do not take part in this study? (4/18/06)

Your other choices may include:

- Getting treatment or care for your cancer without being in a study; this could include the following options, either alone or in combination with each other:
  - External (non–three-dimensional) radiation therapy
  - Internal radiation (seed implants or brachytherapy)
  - Three-dimensional radiation therapy or IMRT similar to the therapy described in this study
  - Surgery
  - Hormone therapy
- Taking part in another study
- Getting no treatment (With this choice, your tumor could continue to grow and your disease could spread)

Talk to your study doctor about your choices before you decide if you will take part in this study.

Will my medical information be kept private?

We will do our best to make sure that the personal information in your medical record will be kept private. However, we cannot guarantee total privacy. Your personal information may be given out if required by law. If information from this study is published or presented at scientific meetings, your name and other personal information will not be used.

Organizations that may look at and/or copy your medical records for research, quality assurance, and data analysis include:

- The Radiation Therapy Oncology Group
- The National Cancer Institute (NCI) and other government agencies involved in keeping research safe for people, like the Central Institutional Review Board (CIRB) and the Food and Drug Administration (FDA)
- The Cancer Trials Support Unit (CTSU), a research group sponsored by the National Cancer Institute (NCI) to provide greater access to cancer trials [for CTSU participants only]
- A Data Monitoring Committee (DMC) that regularly meets to monitor safety and other data related to this study

What are the costs of taking part in this study?

You and/or your health plan/insurance company will need to pay for some or all of the costs of treating your cancer in this study. Some health plans will not pay these costs for people taking part in studies. Check with your health plan or insurance company to find out what they will pay for. Taking part in this study may or may not cost your insurance company more (or less) than the cost of getting regular cancer treatment.

You will not be paid for taking part in this study.

For more information on clinical trials and insurance coverage, you can visit the National Cancer Institute's Web site at http://www.cancer.gov/clinicaltrials/understanding/insurance-coverage. You can print a copy of the "Clinical Trials and Insurance Coverage" information from this Web site.

Another way to get the information is to call 1-800-4-CANCER (1-800-422-6237) and ask them to send you a free copy.

What happens if I am injured because I took part in this study?

It is important that you tell your study doctor, ___________________ *[investigator's name(s)],* if you feel that you have been injured because of taking part in this study. You can tell the study doctor in person or call him/her at ___________________ *[telephone number].*

You will get medical treatment if you are injured as a result of taking part in this study. You and/or your health plan will be charged for this treatment. The study will not pay for medical treatment.

What are my rights if I take part in this study?

Taking part in this study is your choice. You may choose either to take part or not to take part in the study. If you decide to take part in this study, you may leave the study at any time. No matter what decision you make, there will be no penalty to you and you will not lose any of your regular benefits. Leaving the study will not affect your medical care. You can still get your medical care from our institution.

We will tell you about new information or changes in the study that may affect your health or your willingness to continue in the study.

In the case of injury resulting from this study, you do not lose any of your legal rights to seek payment by signing this form.

Who can answer my questions about the study?

You can talk to your study doctor about any questions or concerns you have about this study. Contact your study doctor __________________ *[name(s)]* at __________________ *[telephone number].*

For questions about your rights while taking part in this study, call the ________________________ *[name of center]* Institutional Review Board (a group of people who review the research to protect your rights) at __________________ *(telephone number). [Note to Local Investigator: Contact information for patient representatives or other individuals in a local institution who are not on the IRB or research team but take calls regarding clinical trial questions can be listed here.]*

*You may also call the Operations Office of the NCI Central Institutional Review Board (CIRB) at 888-657-3711 (from the continental US only). *[*Only applies to sites using the CIRB.]*

Please note: This section of the informed consent form is about additional research that is being done with people who are taking part in the main study. You may take part in this additional research if you want to. You can still be a part of the main study even if you say 'no' to taking part in this additional research.

You can say "yes" or "no" to each of the following studies. Below, please mark your choice for each study.

<u>Consent Form for Quality of Life Study</u> (9/20/07)

We want to know your view of how your life has been affected by cancer and its treatment. This "Quality of Life" study looks at how you are feeling physically and emotionally during your cancer treatment. It also looks at how you are able to carry out your day-to-day activities.

This information will help doctors better understand how patients feel during treatments and what effects the treatments are having. In the future, this information may help patients and doctors as they decide which treatments to use to treat cancer.

You will be asked to complete four questionnaires at the following time points: immediately before you enroll in the study, at 6, 12, and 24 months following the start of your radiation treatment, and at 5 years following the start of your radiation treatment. It takes about 25-30 minutes to fill out the questionnaires.

If any questions make you feel uncomfortable, you may skip those questions and not give an answer.

If you decide to take part in this study, the only thing you will be asked to do is fill out the four questionnaires. You may change your mind about completing the questionnaires at any time, and you may chose to discontinue answering the questionnaires altogether at any time.

No matter what you decide to do, it will not affect your care or your participation in the main part of the study.

Just like in the main study, we will do our best to make sure that your personal information will be kept private. You will not be paid for taking part in this study.

Please circle your answer.

I choose to take part in the Quality of Life study. I agree to fill out the four Quality of Life questionnaires.

Yes **No**

<u>**Consent Form for Use of Tissue and Blood for Research**</u>

About Using Tissue and Blood for Research (9/20/07)
You have had a biopsy (or surgery) to see if you have cancer. Your doctor has removed some of your tissue to do some tests. The results of these tests will be given to you by your doctor and will be used to plan your care.

We would like to keep some of the tissue that is left over from your biopsy for future research. If you agree, this tissue will be kept and may be used in research to learn more about cancer and other diseases. Please read the information sheet called "How is Tissue Used for Research" to learn more about tissue research. This information sheet is available to all at the following web site: http://www.rtog.org/tissue%20for%20research_patient.pdf

(7/9/09) In addition, you will have blood tests before you start treatment and during treatment. We would like to keep about four teaspoons of blood for future research as well. If you agree, this blood will be kept and may be used in research to learn more about cancer and other diseases. One specific test will analyze whether your blood contains certain genes and if the side effects you had on radiation are related to these genes. We will then try to see if these genes can help us learn about why some people get worse side effects than others.

Your tissue and blood may be helpful for research. The research that may be done with your tissue and blood is not designed specifically to help you. It might help people who have cancer and other diseases in the future.

Reports about research done with your tissue and blood will not be given to you or your doctor. These reports will not be put in your health record. The research will not have an effect on your care.

Things to Think About
The choice to let us keep the left over tissue and blood for future research is up to you. No matter what you decide to do, it will not affect your care or your participation in the main part of the study.

If you decide now that your tissue and blood can be kept for research, you can change your mind at any time. Just contact us and let us know that you do not want us to use your tissue and blood. Then any tissue or blood that remains will no longer be used for research; remaining tissue will be returned to the institution that submitted it and remaining blood will be destroyed.

In the future, people who do research may need to know more about your health. While the study doctor/institution may give them reports about your health, the study doctor/institution will not give them your name, address, phone number, or any other information that will let the researchers know who you are.

Sometimes tissue and blood are used for genetic research (about diseases that are passed on in families). Even if your tissue and blood are used for this kind of research, the results will not be put in your health records.

Your tissue and blood will be used only for research and will not be sold. The research done with your tissue and blood may help to develop new products in the future. You will not be paid for taking part in this study.

Benefits
The benefits of research using tissue and blood include learning more about what causes cancer and other diseases, how to prevent them, and how to treat them.

Risks
The greatest risk to you is the release of information from your health records. We will do our best to make sure that your personal information will be kept private. The chance that this information will be given to someone else is very small.

Making Your Choice (9/20/07)
Please read each sentence below and think about your choice. After reading each sentence, circle "Yes" or "No". If you have any questions, please talk to your doctor or nurse or call our research review board at *[IRB's phone number]*.

No matter what you decide to do, it will not affect your care.

1. My tissue/blood may be kept for use in research to learn about, prevent, or treat cancer.

Yes **No**

2. My tissue/blood may be kept for use in research to learn about, prevent or treat other health problems (for example: diabetes, Alzheimer's disease, or heart disease).

Yes **No**

3. My blood may be kept for use in future research to learn about the correlation between genes and radiation side effects.

Yes **No**

4. Someone may contact me in the future to ask me to take part in more research.

Yes **No**

Where can I get more information?

You may call the National Cancer Institute's Cancer Information Service at:

1-800-4-CANCER (1-800-422-6237) or **TTY: 1-800-332-8615**

You may also visit the NCI Web site at http://www.cancer.gov/

- **For NCI's clinical trials information, go to http://www.cancer.gov/clinicaltrials/**
- **For NCI's general information about cancer, go to http://cancer.gov/cancerinfo/**

You will get a copy of this form. If you want more information about this study, ask your study doctor.

I have been given a copy of all _____ *[insert total of number of pages]* **pages of this form. I have read it or it has been read to me. I understand the information and have had my questions answered. I agree to take part in this study.**

Participant ________________________________

Date _____________________________________

APPENDIX II (7/9/09)

ZUBROD PERFORMANCE SCALE

0	Fully active, able to carry on all predisease activities without restriction
1	Restricted in physically strenuous activity but ambulatory and able to carry work of a light or sedentary nature. For example, light housework, office work
2	Ambulatory and capable of all self-care but unable to carry out any work activities. Up and about more than 50% of waking hours
3	Capable of only limited self-care, confined to bed or chair 50% or more of waking hours
4	Completely disabled. Cannot carry on self-care. Totally confined to bed or
5	Death

APPENDIX III

AJCC STAGING SYSTEM
PROSTATE, 6th Edition

DEFINITION OF TNM

Primary Tumor, Clinical (T)

TX Primary tumor cannot be assessed

T0 No evidence of primary tumor

T1 Clinically inapparent tumor not palpable or visible by imaging
- T1a Tumor incidental histologic finding in 5% or less of tissue resected
- T1b Tumor incidental histologic finding in more than 5% of tissue resected
- T1c Tumor identified by needle biopsy *(e.g., because of elevated PSA)*

T2 Tumor confined with prostate*
- T2a Tumor involves less than ½ of one lobe
- T2b Tumors involves greater than ½ of one lobe but < 2 lobes
- T2c Tumor involves both lobes

T3 Tumor extends through prostate capsule**
- T3a Extracapsular extension *(unilateral or bilateral)*
- T3b Tumor involves the seminal vesicle*(s)*

T4 Tumor is fixed or invades adjacent structures other than the seminal vesicles: bladder neck, external sphincter, rectum, levator muscles and/or pelvic wall

*Note: Tumor found in one or both lobes by needle biopsy, but not palpable or reliably visible by imaging, is classified as T1c

**Note: Invasion into the prostatic apex or into *(but not beyond)* the prostatic capsule is not classified as T3, but as T2.

Regional Lymph Nodes (N)

Clinical

NX Regional lymph nodes cannot be assessed
N0 No regional lymph node metastasis
N1 Metastasis in regional lymph node or nodes

Pathologic

pNX Regional nodes not sampled
pN0 No positive regional nodes
pN1 Metastases in regional node(s)

Primary Tumor, Pathologic (pT)

pT2*** Organ confined
- pT2a Unilateral
- pT2b Bilateral

pT3 Extraprostatic extension
- pT3a Extraprostatic extension
- pT3b Seminal vesicle invasion

pT4 Invasion of bladder, rectum

***Note: There is no pathologic T1 classification

APPENDIX III ***(continued)***

AJCC STAGING SYSTEM
PROSTATE, 6th Edition

Distant Metastasis* (M)***

MX	Presence of distant metastasis cannot be assessed	
M0	No distant metastasisM1	Distant metastasis
	M1a	Non regional lymph node*(s)*
	M1b	Bone*(s)*
	M1c	Other site*(s)*

****Note: When more than one site of metastasis is present, the most advanced category is used. pM1c is most advanced

Histopathologic Grade (G)

GX	Grade cannot be assessed
G1	Well-differentiated *(slight anaplasia)*
G2	Moderately differentiated *(moderate anaplasia)*
G3-4	Poorly undifferentiated or undifferentiated *(marked anaplasia)*

Stage Grouping

Stage I	T1a	N0	M0	G1
Stage II	T1a	N0	M0	G2, G3-4
	T1b	N0	M0	Any G
	T1c	N0	M0	Any G
	T1	N0	N0	Any G
	T2	N0	M0	Any G
Stage III	T3	N0	M0	Any G
Stage IV	T4	N0	M0	Any G
	Any T	N1,	M0	Any G
	Any T	Any N	M1	Any G

APPENDIX IV (9/20/07) (7/9/09)

Specimen Plug Kit* and Instructions

The Specimen Plug Kit contains a shipping tube and a dermal needle. **Note**: **Sites should not dispose of the Plug Kit.** Sites should ship the Plug Kit to the RTOG Biospecimen Resource to be used again.

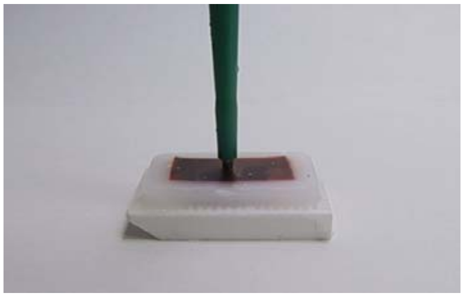

Step 1
Place the dermal needle on the paraffin block over the selected tumor area. (*Ask a Pathologist to select area with tumor.*) Push the needle into the paraffin block. Twist the needle once around to separate the plug from the block. Then pull the needle out of the block. The needle should be filled with tissue sample.

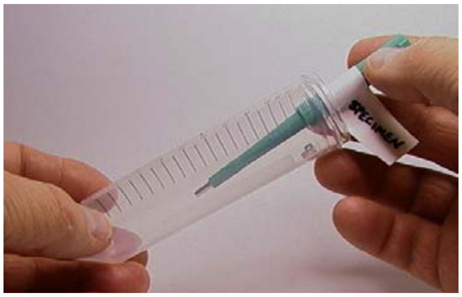

Step 2
Label dermal needle with the pathology accession number, RTOG study and case numbers. **Do not try to remove specimen from needle.**

Use a separate dermal needle for every specimen. **Do not mix specimens.** Call or e-mail the RTOG Biospecimen Resource for questions or for additional specimen Plug Kits.

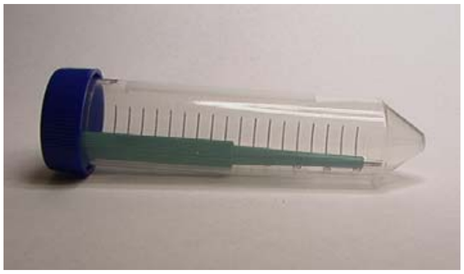

Step 3
Once specimen needle is labeled, place it in the shipping tube and mail to the address below.

The RTOG Biospecimen Resource will remove the specimen from the needle and embed it in a cassette, labeled with the specimen ID.

***NOTE**: If an institution is uncomfortable obtaining the plug but wants to retain the tissue block, the institution should send the entire block to the RTOG Biospecimen Resource. The Biospecimen Resource will sample a plug from the block and will return the remaining block to the institution. Institutions should indicate their request to perform the plug procedure and to return the block on the submission form.

Paraffin Blocks: All specimens should be fixed in 10% buffered formalin. The method of fixation is dependent on feasibility at the local institution. Immersion of the serially sliced sections in formalin is acceptable provided that slices are no more than 1 cm in thickness. Specimens are to be placed in adequate-sized containers with a 10-fold excess of fresh (non-bloody formalin). Whatever method is chosen, good penetration of tissue by fixative is essential. After overnight fixation, the specimen is to be carefully dissected, and the tissue blocks are to be removed from the specimen for embedding in paraffin, orienting the specimen **on edge**.

Fresh, Flash Frozen Tissue: Harvested directly by punch biopsy or from the surgical specimen and snap frozen in liquid nitrogen without preservatives. After biopsy, evenly cut tissue into 5 mm^3 sections. Use forceps to place each piece into separate 5 ml cryovial. Place the cryovials into liquid nitrogen. Once frozen, place all of the cryovials into biohazard bag and label bag. Store specimens frozen until ready to mail.

Ship: Specimen Plug Kit, specimen in dermal needle, and all paper work as follows:

<u>APPENDIX IV</u> (Continued)

U. S. Postal Service Mailing Address: <u>For Non-frozen Specimens Only</u>
RTOG Biospecimen Resource
University of California San Francisco
Campus Box 1800
1657 Scott Street, Room 223
San Francisco, CA 94143-1800

Courier Address (FedEx, UPS, etc.): <u>For Frozen Specimens</u>
RTOG Biospecimen Resource
University of California San Francisco
1657 Scott Street, Room 223
San Francisco, CA 94115

Questions: 415-476-RTOG (7864)/FAX 415-476-5271; RTOG@ucsf.edu

APPENDIX V (9/20/07) (7/9/09)

BLOOD COLLECTION KIT INSTRUCTIONS

Instructions for use of serum, plasma, or buffy coat collection kit (collected as required by protocol):

This kit includes:
- Ten (10) 1 ml cryovials
- Biohazard bags
- Absorbent shipping material
- Styrofoam container (inner)
- Cardboard shipping (outer) box
- Pre-paid shipping label(s)

Serum (if requested):
- ❑ Using four (4) or more 1 ml cryovials, label them with the RTOG study and case number, collection date and time, and clearly mark cryovials "serum".

Process:
1. Allow one red top tube to clot for 30 minutes at room temperature.
2. Spin in a standard clinical centrifuge at ~2500 RPM for 10 minutes at room temperature.
3. Aliquot a minimum of 0.5 ml serum (optimal 1ml) into each cryovial labeled with RTOG study and case numbers, collection date/time, time point collected, and clearly mark specimen as "serum".
4. Place cryovials into biohazard bag and immediately freeze at –70 to –80° Celsius.
5. Store serum at –70 to –80° Celsius until ready to ship.
6. Ship on dry ice.

PLEASE MAKE SURE THAT EVERY SPECIMEN IS LABELED.

Plasma (If requested):
- ❑ Using three (3) or more 1 ml cryovials, label them with the RTOG study and case number, collection date and time, and clearly mark cryovials "plasma".

Process:
1. After collection, invert tube(s) multiple times to ensure adequate mixing of EDTA.
2. Centrifuge specimen(s) within one hour of collection in a standard clinical centrifuge at ~2500 RPM for 10 minutes at room temperature.
3. If the interval between specimen collection and processing is anticipated to be greater than one hour, keep specimen on ice until centrifuging is performed.
4. Carefully pipette and aliquot a minimum of 0.5 ml plasma (optimal 1 ml) into each cryovial labeled with RTOG study and case numbers, collection date/time, time point collected and clearly mark specimen as "plasma".
5. Place cryovials into biohazard bag and immediately freeze at –70 to –80° Celsius.
6. Store plasma at –70 to –80° Celsius until ready to ship.
7. Ship on dry ice.

PLEASE MAKE SURE THAT EVERY SPECIMEN IS LABELED.

Buffy coat (if requested):

For a visual explanation of Buffy coat, please refer to diagram below.

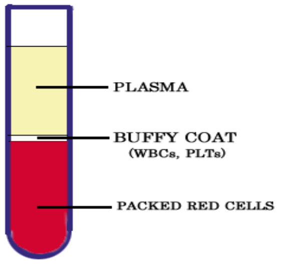

- ❑ Using one (1) or more 1 ml cryovials, label them with the RTOG study and case number, collection date and time, and clearly mark cryovial(s) "buffy coat".

Process:
1. Centrifuge EDTA (purple top) tube within one hour of collection in a standard clinical centrifuge at ~2500 RPM for 10 minutes at room temperature.
2. If the interval between specimen collection and processing is anticipated to be greater than one hour, keep specimen on ice until centrifuging is performed.
3. Carefully remove plasma close to the buffy coat and set plasma aside (*can be used to send plasma samples – see above instructions*).
4. Remove the buffy coat cells carefully and place into cryovials labeled "buffy coat" (*it is okay if a few packed red cells are inadvertently collected in the process*). Clearly mark the tubes with date/time of collection and time point collected.
5. Place cryovials into biohazard bag and freeze immediately at -70 to -80° Celsius.
6. Store buffy coat samples frozen (-70 to -80° Celsius) until ready to ship.
7. Ship on dry ice.

PLEASE MAKE SURE THAT EVERY SPECIMEN IS LABELED.

Shipping/Mailing:
- ❑ Ship specimens overnight **Monday-Wednesday** to prevent thawing due to delivery delays. Saturday and holiday deliveries will not be accepted.
- ❑ Include all RTOG paperwork in a sealed plastic and tape to the outside top of the Styrofoam box.
- ❑ Wrap frozen specimens of same type (i.e., all serum together, plasma together and buffy coats together) in absorbent shipping material and place each specimen type in a separate biohazard bag. Place specimen bags into the Styrofoam cooler and fill with dry ice (4-5lbs/2-2.5kg minimum). Ship ambient specimens in a separate envelope/cooler. Place Styrofoam coolers into outer cardboard box, and attach shipping label to outer cardboard box.
- ❑ *Multiple cases may be shipped in the same cooler, but make sure each one is in a separate bag.*

For questions regarding collection, shipping or to order a Blood Collection Kit, contact the RTOG Biospecimen Resource at 415-476-RTOG (7864)/FAX 415-476-5271; RTOG@ucsf.edu

APPENDIX VI (1/24/07) (9/20/07) (1/8/08)

CTSU LOGISTICS

ADDRESS AND CONTACT INFORMATION FOR RTOG-0415

To submit site registration documents:	For patient enrollments:	Submit study data directly to the RTOG unless otherwise specified in the protocol:
CTSU Regulatory Office<br>1818 Market Street, Suite 1100<br>Philadelphia, PA 19103<br>Phone - 1-888-823-5923<br>Fax – 215-569-0206	CTSU Patient Registration<br>Voice Mail – 1-888-462-3009<br>Fax – 1-888-691-8039<br>Hours: 9:00 AM – 5:30 PM Eastern Time, Monday – Friday (excluding holidays)<br><br>[For CTSU patient enrollments that must be completed within approximately one hour, or other extenuating circumstances, call 301-704-2376. Please use the 1-888-462-3009 number for ALL other CTSU patient enrollments.]	RTOG Headquarters<br>1818 Market Street, Suite 1600<br>Philadelphia, PA 19103<br><br>Please do not submit study data or forms to CTSU Data Operations. Do not copy the CTSU on data submissions.
**For patient eligibility questions:**<br>Contact the RTOG Research Associate for Protocol, Data Management section at 215-574-3214.<br>**For treatment-related questions:**<br>Correspond by e-mail (preferred) or by phone with the study chair designated on the protocol cover page.		
**For questions unrelated to patient eligibility, treatment, or data submission** contact the CTSU Help Desk by phone or e-mail:<br>CTSU General Information Line – 1-888-823-5923, or ctsucontact@westat.com. All calls and correspondence will be triaged to the appropriate CTSU representative.		
**The CTSU Public Web site** is located at: www.ctsu.org<br><br>**The CTSU Registered Member Web site** is located at: http://members.ctsu.org		

CANCER TRIALS SUPPORT UNIT (CTSU) PARTICIPATION PROCEDURES

REGISTRATION/RANDOMIZATION

Prior to the recruitment of a patient for this study, investigators must be registered members of the CTSU. Each investigator must have an NCI investigator number and must maintain an "active" investigator registration status through the annual submission of a complete investigator registration packet (FDA Form 1572 with original signature, current CV, Supplemental Investigator Data Form with signature, and Financial Disclosure Form with original signature) to the Pharmaceutical Management Branch, CTEP, DCTD, NCI. These forms are available on the CTSU registered member Web site or by calling the PMB at 301-496-5725 Monday through Friday between 8:30 a.m. and 4:30 p.m. Eastern time.

Each CTSU investigator or group of investigators at a clinical site must obtain IRB approval for this protocol and submit IRB approval and supporting documentation to the CTSU Regulatory Office before they can enroll patients. Study centers can check the status of their registration packets by querying the Regulatory Support System (RSS) site registration status page of the CTSU member web site at http://members.ctsu.org

All forms and documents associated with this study can be downloaded from the RTOG-0415 Web page on the CTSU registered member Web site (http://members.ctsu.org). Patients can be registered only after pre-treatment evaluation is complete, all eligibility criteria have been met, and the study site is listed as 'approved' in the CTSU RSS.

Requirements for RTOG-0415 site registration:

- **All patients MUST be treated with either 3DCRT or IMRT on this trial and all institutions must be pre-credentialed.** Credentialing requirements for 3DCRT and IMRT Treatment Approach are outlined in Section 5.1 of the protocol and on the Advanced Technology Consortium (ATC) web site at http://atc.wustl.edu. Submission of digital data to the Image-Guided Therapy Center (ITC) requires advanced request for an FTP account with the ITC (itc@castor.wustl.edu). The ITC will notify the registering institution when that institution is eligible to enter patients on study. The status of the credentialing review will be reflected on the RSS Site Registration Status screen http://members.ctsu.org/rss/

- CTSU IRB Certification
- CTSU IRB/Regulatory Approval Transmittal Sheet
- CTSU RT Facilities Inventory Form

Note: Per NCI policy all institutions that participate on protocols with a radiation therapy component must participate in the Radiological Physics Center (RPC) monitoring program. For sites enrolling through the CTSU an RT Facilities Inventory From must be on file with CTSU. If this form has been previously submitted to CTSU it does not need to be resubmitted unless updates have occurred at the RT facility.

Pre-study requirements for patient enrollment on RTOG-0415

- Patient must meet all inclusion criteria, and no exclusion criteria should apply.
- Patient has signed and dated all applicable consents and authorization forms.
- All baseline laboratory tests and prestudy evaluations performed within the time period specified in the protocol.
- Baseline QOL forms completed prior to treatment start.

CTSU Procedures for Patient Enrollment

1. Contact the CTSU Patient Registration Office by calling 1-888-462-3009. Leave a voicemail to alert the CTSU Patient Registrar that an enrollment is forthcoming. For immediate registration needs, e.g. within one hour, call the registrar cell phone at 1-301-704-2376.

2. Complete the following forms:

- CTSU Patient Enrollment Transmittal Form
- RTOG-0415 Eligibility Checklist

3. Fax these forms to the CTSU Patient Registrar at 1-888-691-8039 between the hours of 9:00 a.m. and 5:30 p.m., Mon-Fri, Eastern Time (excluding holidays); however, please be aware that RTOG registration hours end at 4:30 pm Eastern Time. The CTSU registrar will check the investigator and site information to ensure that all regulatory requirements have been met. The registrar will also check that forms are complete and follow-up with the site to resolve any discrepancies.

4. Once investigator eligibility is confirmed and enrollment documents are reviewed for compliance, the CTSU registrar will contact the RTOG within the confines of RTOG's registration hours to obtain assignment of a treatment arm and assignment of a unique patient ID (to be used on all future forms and correspondence). The CTSU registrar will confirm registration by fax.

Study treatment must begin within 6 weeks of patient registration.

APPENDIX VI (Continued)

DATA SUBMISSION AND RECONCILIATION

1. All case report forms (CRFs) and transmittals associated with this study must be downloaded from the RTOG-0415 web page located on the CTSU registered member Web site (http://members.ctsu.org). Sites must use the current form versions and adhere to the instructions and submission schedule outlined in the protocol.

2. Submit all completed CRFs (with the exception of patient enrollment forms), clinical reports, and transmittals to RTOG Headquarters unless an alternate location is specified in the protocol. Do not send study data to the CTSU. See the Special Materials or Substudies section below for submission of dosimetry data.

3. The RTOG Headquarters will send query notices and delinquency reports to the site for reconciliation. Please send query responses and delinquent data to the RTOG and do not copy CTSU Data Operations. Each clinical site should have a designated CTSU Administrator and Data Administrator and must keep their CTEP AMS account contact information current. This will ensure timely communication between the clinical site and the RTOG.

4. Please affix the RTOG study/case label to all source documentation and redact the patient's name.

SPECIAL MATERIALS OR SUBSTUDIES

Radiation Therapy (section 6.0)
Dosimetry data for 3DCRT and IMRT must be submitted to the Image-Guided Therapy Center (ITC), either by digital transmission using the ITC-assigned FTP account or tape submission (contact ITC for acceptable tape types and format). Hard copy materials accompanying digital data should also be sent directly to the ITC. See section 12.2 for a complete inventory of dosimetry items to be submitted.

(7/9/09) Specimen Submission (section 10.0)
1. **Tumor tissue for central pathology review is required**. A pathology report and RTOG Specimen Transmittal Form must accompany specimens in order for the case to be considered evaluable by the RTOG Biospecimen Resource at the University of California San Francisco .

2. With patient's consent, tumor tissue and blood will be collected for biomarkers research and banking. Submit specimens, pathology report, and RTOG Specimen Transmittal Form to the RTOG Biospecimen Resource at the University of California San Francisco .

3. See protocol section 10.0 for detailed instructions on collection, preparation, and shipment of samples.
All reports must include the protocol number and patient's case number (or RTOG label attached). Surgical pathology numbers and information must not be removed from the report; however, the patient's name and/or other identifying information should be redacted. Do not send specimens, forms, reports, or transmittals to the CTSU.

4. CTSU clinical sites qualify for specimen reimbursement in the amounts stated in section 10.5 of the protocol. Payments will be made in accordance with RTOG's pathology payment cycle and forwarded to the enrolling sites by the Cooperative Group credited with the accrual.

SERIOUS ADVERSE EVENT (SAE) REPORTING

1. CTSU sites must comply with the expectations of their local Institutional Review Board (IRB) regarding documentation and submission of adverse events. Local IRBs must be informed of all reportable serious adverse reactions.

2. CTSU sites will assess and report adverse events according to the guidelines and timelines specified in the protocol. You may navigate to the CTEP Adverse Event Expedited Report System (AdEERS) from either the Adverse Events tab of the CTSU member homepage (http://members.ctsu.org) or by selecting Adverse Event Reporting Forms from the document center drop down list on the RTOG-0415 web page.

APPENDIX VI (Continued)

3. Do not send adverse event reports to the CTSU.

4. Secondary AML/MDS/ALL reporting: Report occurrence of secondary AML, MDS, or ALL via the NCI/CTEP AML-MDS Report Form in lieu of AdEERS. Submit the completed form and supporting documentation as outlined in the protocol.

DRUG PROCUREMENT
Not applicable to this study.

REGULATORY AND MONITORING
Study Audit

To assure compliance with Federal regulatory requirements [CFR 21 parts 50, 54, 56, 312, 314 and HHS 45 CFR 46] and National Cancer Institute (NCI)/ Cancer Therapy Evaluation Program (CTEP) Clinical Trials Monitoring Branch (CTMB) guidelines for the conduct of clinical trials and study data validity, all protocols approved by NCI/CTEP that have patient enrollment through the CTSU are subject to audit.

Responsibility for assignment of the audit will be determined by the site's primary affiliation with a Cooperative Group or CTSU. For Group-aligned sites, the audit of a patient registered through CTSU will become the responsibility of the Group receiving credit for the enrollment. For CTSU Independent Clinical Research Sites (CICRS), the CTSU will coordinate the entire audit process.

For patients enrolled through the CTSU, you may request the accrual be credited to any Group for which you have an affiliation provided that Group has an active clinical trials program for the primary disease type being addressed by the protocol. Per capita reimbursement will be issued by the credited Group provided they have endorsed the trial, or by the CTSU if the Group has not endorsed the trial.

Details on audit evaluation components, site selection, patient case selection, materials to be reviewed, site preparation, on-site procedures for review and assessment, and results reporting and follow-up are available for download from the CTSU Operations Manual located on the CTSU Member Web site.

Health Insurance Portability and Accountability Act of 1996 (HIPAA)

The HIPAA Privacy Rule establishes the conditions under which protected health information may be used or disclosed by covered entities for research purposes. Research is defined in the Privacy Rule referenced in HHS 45 CFR 164.501. Templated language addressing NCI-U.S. HIPAA guidelines are provided in the HIPAA Authorization Form located on the CTSU website.

The HIPAA Privacy Rule does not affect participants from outside the United States. Authorization to release Protected Health Information is NOT required from patients enrolled in clinical trials at non-US sites.

Clinical Data Update System (CDUS) Monitoring

This study will be monitored by the Clinical Data Update System (CDUS) Version 3.0. Cumulative CDUS data will be submitted quarterly to CTEP by electronic means. The sponsoring Group fulfills this reporting obligation by electronically transmitting to CTEP the CDUS data collected from the study-specific case report forms.
