# Supplementary material for: Conditional Risks of Biochemical Failure and Prostate Cancer–Specific Death in Patients Undergoing External Beam Radiotherapy: A Secondary Analysis of 2 Randomized Clinical Trials
Source: JAMA Netw Open. 2023 Sep 26;6(9):e2335069. doi: 10.1001/jamanetworkopen.2023.35069 (PMC10523164; doi:10.1001/jamanetworkopen.2023.35069)
Supplement: Supplement 3. — Data Sharing Statement [file jamanetwopen-e2335069-s003.pdf]

Data Sharing Statement

Alexander. Conditional Risks of Biochemical Failure and Prostate Cancer–Specific Death in Patients Undergoing External Beam Radiotherapy. *JAMA Netw Open*. Published September 26, 2023. doi:10.1001/jamanetworkopen.2023.35069

Data
Data available: Yes
Data types: Deidentified participant data
How to access data: https://nctn-data-archive.nci.nih.gov/
When available: With publication

Supporting Documents
Document types: None

Additional Information
Who can access the data: researchers whose proposed use of the data has been approved
Types of analyses: For any specified purpose
Mechanisms of data availability: After signed agreement
Any additional restrictions: None
